# Tofacitinib blocks IFN-regulated biomarker genes in skin fibroblasts and keratinocytes in a systemic sclerosis trial

**DOI:** 10.1172/jci.insight.159566

**Published:** 2022-09-08

**Authors:** Dinesh Khanna, Cristina Padilla, Lam C. Tsoi, Vivek Nagaraja, Puja P. Khanna, Tracy Tabib, J. Michelle Kahlenberg, Amber Young, Suiyuan Huang, Johann E. Gudjonsson, David A. Fox, Robert Lafyatis

**Affiliations:** 1 Division of Rheumatology, Department of Internal Medicine, and; 2 University of Michigan Scleroderma Program, University of Michigan, Ann Arbor, Michigan, USA.; 3 Division of Rheumatology, University of Pittsburgh, Pittsburgh, Pennsylvania, USA.; 4 Department of Dermatology, University of Michigan, Ann Arbor, Michigan, USA.; 5 VA Medical Center, Ann Arbor, Michigan, USA.; 6 Department of Biostatistics, University of Michigan, Ann Arbor, Michigan, USA.

**Keywords:** Clinical Trials, Rheumatology, Skin

## Abstract

**BACKGROUND:**

Systemic sclerosis (SSc) is an autoimmune, connective tissue disease characterized by vasculopathy and fibrosis of the skin and internal organs.

**METHODS:**

We randomized 15 participants with early diffuse cutaneous SSc to tofacitinib 5 mg twice a day or matching placebo in a phase I/II double-blind, placebo-controlled trial. The primary outcome measure was safety and tolerability at or before week 24. To understand the changes in gene expression associated with tofacitinib treatment in each skin cell population, we compared single-cell gene expression in punch skin biopsies obtained at baseline and 6 weeks following the initiation of treatment.

**RESULTS:**

Tofacitinib was well tolerated; no participants experienced grade 3 or higher adverse events before or at week 24. Trends in efficacy outcome measures favored tofacitnib. Baseline gene expression in fibroblast and keratinocyte subpopulations indicated IFN-activated gene expression. Tofacitinib inhibited IFN-regulated gene expression in SFRP2/DPP4 fibroblasts (progenitors of myofibroblasts) and in MYOC/CCL19, representing adventitial fibroblasts (*P* < 0.05), as well as in the basal and keratinized layers of the epidermis. Gene expression in macrophages and DCs indicated inhibition of STAT3 by tofacitinib (*P* < 0.05). No clinically meaningful inhibition of T cells and endothelial cells in the skin tissue was observed.

**CONCLUSION:**

These results indicate that mesenchymal and epithelial cells of a target organ in SSc, not the infiltrating lymphocytes, may be the primary focus for therapeutic effects of a Janus kinase inhibitor.

**TRIAL REGISTRATION:**

ClinicalTrials.gov NCT03274076.

**FUNDING:**

Pfizer, NIH/National Institute of Arthritis and Musculoskeletal and Skin Diseases (NIAMS) R01 AR070470, NIH/NIAMS K24 AR063120, Taubman Medical Research Institute and NIH P30 AR075043, and NIH/NIAMS K01 AR072129.

## Introduction

Systemic sclerosis (SSc, scleroderma) is an autoimmune, connective tissue disease characterized by fibrosis in the skin and internal organs and vasculopathy (1). It has the highest case fatality in rheumatic diseases, and one subclassification of this disease, diffuse cutaneous SSc (dcSSc), has a 10-year mortality rate of 50% (1). There are no licensed treatments for SSc, and currently, disease management is focused on organ-specific complications.

The pathogenesis of SSc includes an interplay between autoimmunity, vascular dysfunction, and ensuing fibrosis. T cells play a key role in the pathogenesis of early SSc that can lead to endothelial apoptosis, the production of autoantibodies, and the eventual onset of fibrosis (2–4). Innate immune mechanisms, including type I IFNs, are important in SSc (5). Polymorphisms in IFN-regulatory factors confer increased risk of SSc, and IFN excess is evident in blood and skin of a large percentage of patients with SSc.

Tofacitinib is a potent, selective inhibitor of the Janus kinase (JAK) family of proteins with a high degree of selectivity against other kinases in the human genome (6). Tofacitinib blocks JAKs in the JAK/signal transducer and activator of transcription (STAT) pathway, preferentially JAK1 and JAK3, affecting signaling for IFN-α, IFN-β, IL-6, IL‑7, IL‑10, IL-12, IL-15, IL-21, and IL-23 (7). Tofacitinib blocks IL-2, IL-4, IL-6, IL-7, IL-15, and IL-21 in T cells and IL-6, IFN-α, and IFN-γ signaling in monocytes (8). In murine arthritis, tofacitinib reduces levels of plasma IL-6 and CXCL10 (IFN-γ–induced protein 10), while in rheumatoid arthritis (RA), it lowered IL-6 in one clinical trial but decreased CXCL10 in another.

In the cellular environment, where JAKs signal in pairs, tofacitinib preferentially inhibits phosphorylation of STAT, preventing dimerization and translocation of STAT into the nucleus. Because of selective inhibition of JAK1 and JAK3 by tofacitinib, signaling through common γ chain–containing receptors for several cytokines, including IL‑2, ‑4, ‑7, ‑9, ‑15, and ‑21, is blocked. These cytokines are integral to lymphocyte activation, proliferation, and function, and inhibition of their signaling results in modulation of multiple aspects of the immune response. In addition, inhibition of JAK1 causes attenuation of signaling by additional proinflammatory cytokines, such as IL‑6 and IFN-γ, and affects type I IFN signaling. The effect of JAK inhibition affects not only lymphocyte populations but also myeloid, vascular, and fibroblast populations (9). Specifically, a study by Migita et al. highlighted key roles for the effect of JAK/STAT inhibition on fibroblasts (10).

Bioinformatic analysis showed that IL-6/JAK/STAT3 gene signatures were aberrant in SSc biopsies in 4 independent cohorts (11). The results were confirmed by JAK and STAT3 phosphorylation in skin and lung biopsies from patients with SSc. Treatment of mice with tofacitinib not only prevented bleomycin-induced skin and lung fibrosis (12) but also reduced skin fibrosis in TSK1/+ mice (11). In another mouse model of scleroderma-related lung fibrosis, JAK inhibition prevented the upregulation of M1 and M2 markers with improvement in skin and pulmonary involvement (13). Based on these observations, we conducted a phase I/II trial to evaluate daily oral tofacitinib versus placebo in dcSSc in a 24-week randomized controlled trial. The primary objective was to assess safety and tolerability of tofacitinib, and our secondary objectives were to assess for efficacy on clinical outcome measures and to assess the effect on skin tissue single-cell RNA transcription after treatment with tofacitinib.

## Results

Of 17 participants who gave consent, 15 were randomized at 2 centers between September 2017 and October 2018 (Supplemental Figure 1; supplemental material available online with this article; https://doi.org/10.1172/jci.insight.159566DS1). Ten (100%) in the tofacitinib group and 4 (80%) in the placebo group completed the 24-week trial. At week 24, all participants were included in the modified intent to treat and safety analyses. Fourteen of 15 participants decided to continue in the open-label extension. The demographic and baseline disease characteristics were balanced between the treatment groups (Supplemental Table 1).

Tofacitinib was well tolerated — no participants experienced grade 3 or higher Common Terminology Criteria for Adverse Events (CTCAE) v 4.03 adverse events (AEs) at or before week 24 (predefined primary end point). There were 13 and 10 grade 2 or higher AEs in the tofacitinib and placebo groups, respectively, with 5 AEs of special interest in the tofacitinib arm (4 infections requiring treatment and 1 laboratory abnormality) and no episodes of serious infections, cancer, thromboembolic events, herpes zoster, or gastrointestinal perforations (Supplemental Table 2).

For the efficacy endpoints, the median (25th–75th) change in modified Rodnan skin score (mRSS) was –5.5 (–6.0, –1.0; 0–51 range; a negative score denotes improvement in mRSS) in the tofacitinib group and –2.5 (–7.5, 2.5) in the placebo group, with a treatment difference of –3.0 (–12.0, 6.0, *P* = 0.47; Supplemental Figure 2 and Supplemental Tables 2 and 3). We also saw trends in improvement favoring tofacitinib in a composite endpoint, the American College of Rheumatology Composite Response Index in Systemic Sclerosis (ACR-CRISS), with a median (Q1, Q3) 0.30 (0.0, 1.0) in the tofacitinib group and 0.10 (0.0, 0.6) in the placebo group (Supplemental Table 3).

### Open-label extension.

There were 3 grade 3 AEs in the tofacitinib-to-tofacitinib group and in 1 participant in placebo-to-tofacitinib group (Supplemental Table 2). There was 1 herpes zoster reactivation and 1 serious infection (cytomegalovirus induced hepatitis) in the tofacitinib-to-tofacitinib group and 1 serious AE in the placebo-to-tofacitinib group with a thermal injury (diabetic foot ulcer). There was continued improvement in the mRSS in both groups [–12.5 (–15.5, –5.5) in the tofacitinib-to-tofacitinib and –9.0 (–11.0, –9.0) in the placebo-to-tofacitinib group] and other measures, such as ACR-CRISS.

### Single-cell gene expression in skin of tofacitinib-treated participants.

**Figure 1:**
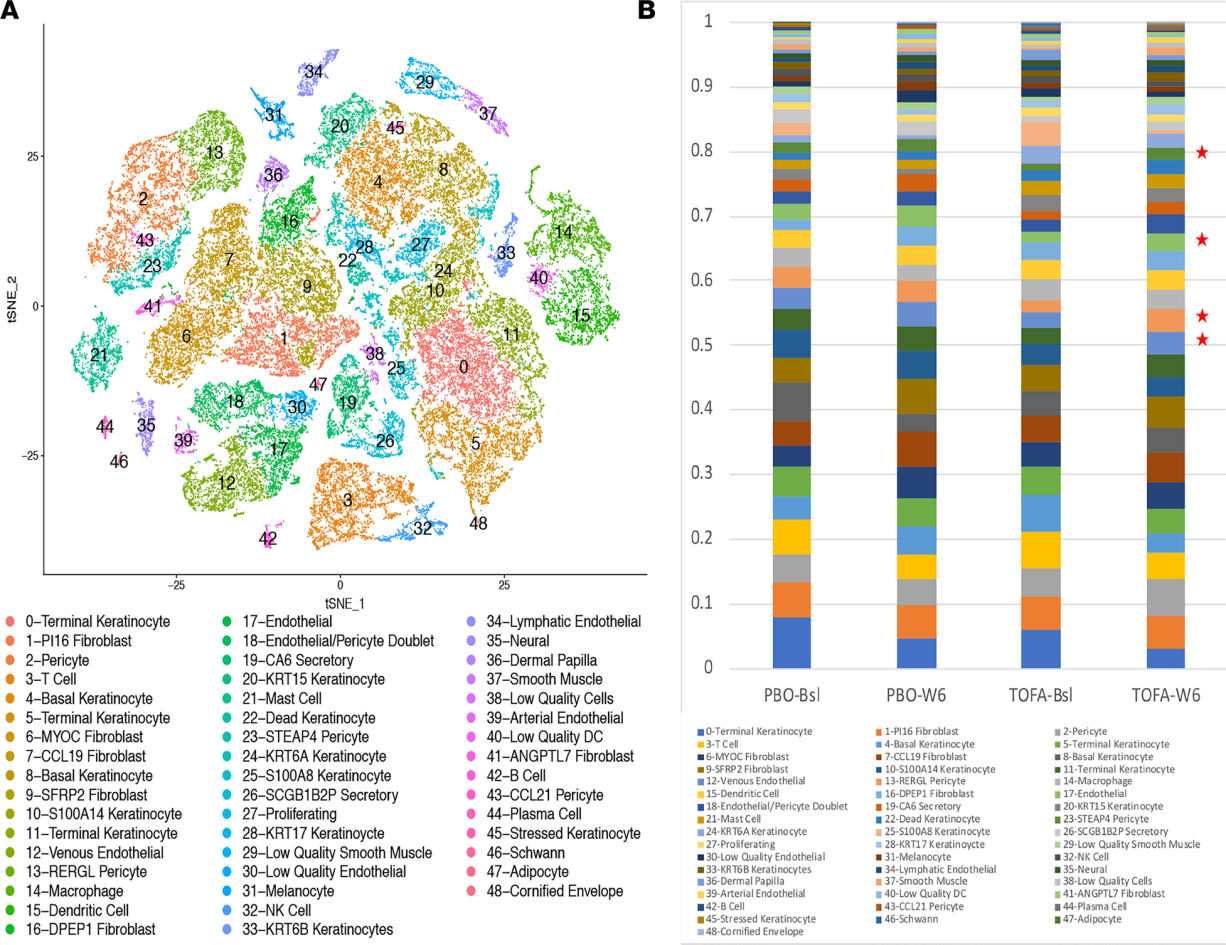
Transcriptomes and proportions of cell populations of study participants. Combined analysis of scRNA-Seq transcriptomes of skin from 15 participants with dcSSc at baseline and 6 weeks after treatment with tofacitinib (*n* = 10) or placebo (*n* = 5; **A**). Cell clusters (*n* = 49) are numbered with cell types based on known marker genes indicated to the right. The proportion of cells in each cluster by subgroups of participants: placebo-treated baseline (PBO-Bsl) and 6-week (PBO-W6) and tofacitinib-treated baseline (TOFA-Bsl) and 6-week (TOFA-W6) biopsies (**B**). Stars indicate pericyte and endothelial cell clusters showing increased proportions of cells after tofacitinib (*P* < 0.05 by paired 2-tailed *t* test corrected for multiple comparisons by Bonferroni’s method).

In order to understand the changes in gene expression associated with tofacitinib treatment in each skin cell population, we examined single-cell gene expression in baseline skin biopsies and again 6 weeks after initiating treatment. We digested the whole skin biopsies into single cells and analyzed transcriptomes using droplet-based single-cell RNA-Seq (scRNA-Seq), obtaining transcriptomes from approximately 2,000–3,000 cells/sample. After filtering out cells with low unique molecular identifier counts, we generated a t-distributed stochastic neighbor embedding plot from approximately ~1,500–2,500 cells/sample (mean 1,875 ± 489), combining the transcriptomes of all the participants entered into the study (Figure 1A). Cells from the placebo- and tofacitinib-treated participants were found in all clusters in biopsies at both baseline and week 6 and were initially analyzed without knowledge of treatment (Supplemental Figures 3 and 4). We identified 49 different cell clusters, including multiple subsets of keratinocytes and pericyte and endothelial cell populations, as well as discrete clusters of T cells, macrophages, DCs, NK cells, B cells, and plasma cells, easily identified by characteristic marker genes identified in the top differentially expressed genes/cluster (Supplemental Table 4).

We examined the proportion of each cell population, focusing on tofacitinib-treated participants in view of the small number of placebo-treated participants who had data at both baseline and week 6 (*n* = 4). This showed a significant increase in the proportion of cells in 2 pericyte and 2 endothelial cell populations after tofacitinib compared with baseline (Figure 1B and Supplemental Figure 5). A third, more common, population of pericytes also showed increased numbers of pericytes, though not reaching statistical significance after correction for multiple testing (Figure 1B). Thus, 5 of 7 pericyte and endothelial cell clusters associated with vascular biology showed increased proportions of cells after tofacitinib. The 2 other vascular cell populations of arterial endothelial cells and CCL21 pericytes represented very low proportions of the total cells.

### Baseline gene expression in fibroblast subpopulations indicates IFN-activated gene expression.

In recent studies, we found that 2 subpopulations of dermal fibroblasts undergo striking changes in gene expression in the skin from participants with dcSSc (14). Furthermore, we found that one of these populations, marked by expression of SFRP2 and DPP4 in healthy skin, appear to be the progenitors of myofibroblasts and show upregulated expression of genes that in bulk RNA skin gene expression correlate with degree of clinical skin thickness, as assessed by the mRSS. In our tofacitinib-treated data set, these cells were largely found in clusters 1 and 9 (Figure 1A). The second of these populations, marked by expression of MYOC and CCL19, represent adventitial fibroblasts and a morphologically distinct population of reticular fibroblasts. In our tofacitinib-treated data set, these cells were largely found in clusters 6 and 7 (Figure 1A). To further the understanding of how SSc affects dermal fibroblast populations, we examined pathways activated in these 2 populations, studying genes correlating with the mRSS in tofacitinib-treated baseline skin biopsies (15 SSc samples) and other SSc and healthy skin biopsies previously examined by scRNA-Seq (10 healthy skin and 12 SSc samples).

**Figure 2:**
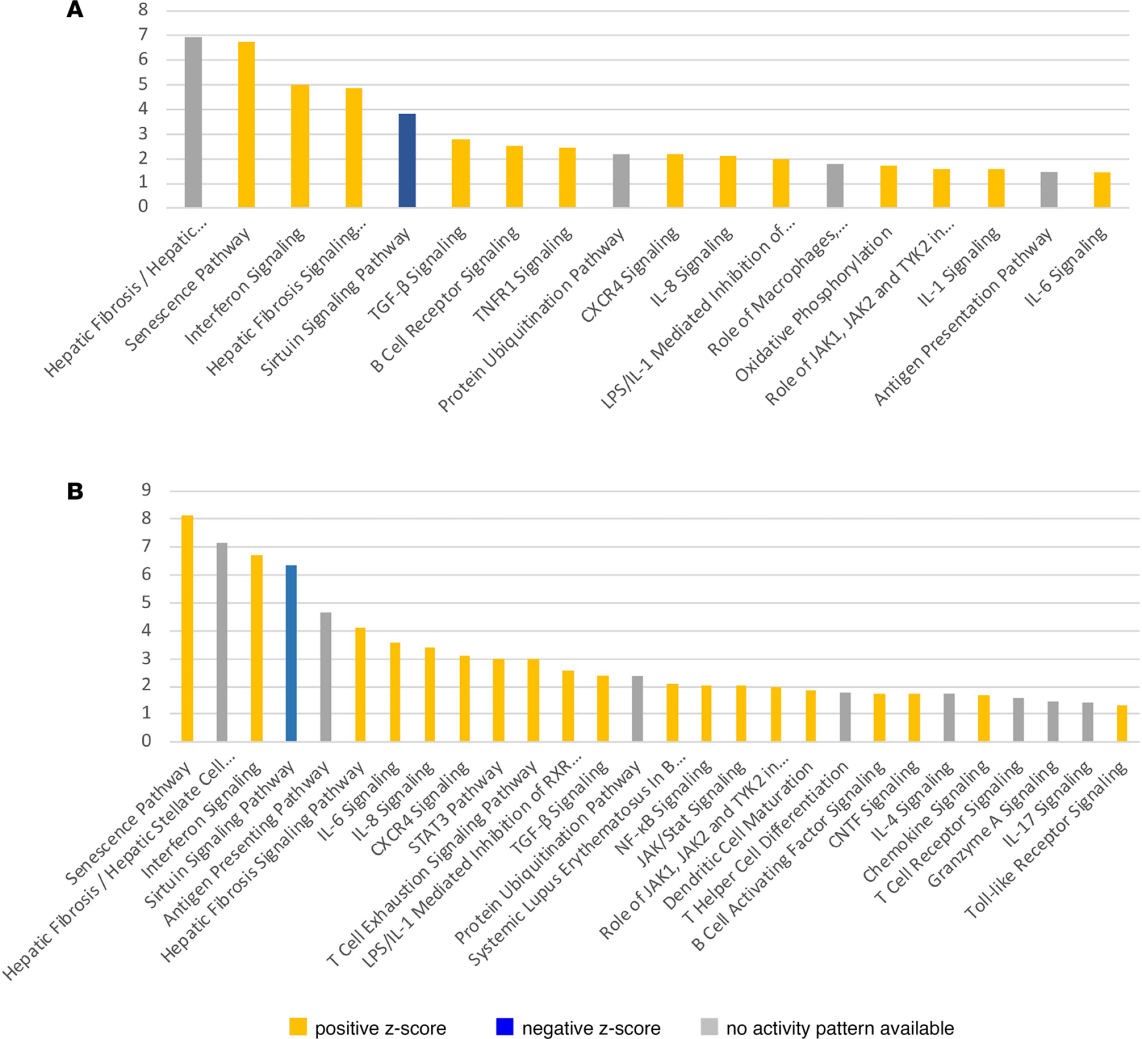
IPA of scRNA-Seq from fibroblast populations. Pathway analysis of fibroblast scRNA-Seq data from baseline study biopsies (*n* = 15), analyzed together with scRNA-Seq data from previously described dcSSc (*n* = 12) and healthy skin (*n* = 10). Selected pathways from clusters 1 and 9, clustered with analogous cells in previous studies, representing SFRP2/DPP4 fibroblasts (**A**) and clusters 6 and 7 clustered with analogous cells in previous studies representing MYOC/CCL19 fibroblasts (**B**). Genes correlating with baseline mRSS (uncorrected *P* < 0.05) were included in the pathway analysis. Only selected significant pathways (–log *P* < 1.4) are indicated. SFRP2/DPP4. Yellow bars indicate positive associations with IPA-expected direction of regulation; blue bars show negative associations with expected direction of regulation; and gray bars indicate no expected direction of regulation.

Ingenuity Pathway Analysis (IPA; QIAGEN) of SFRP2/DPP4 fibroblast gene expression with a focus on components that positively correlated with the mRSS revealed several prominent pathways: hepatic fibrosis/hepatic stellate cell activation, senescence, interferon signaling, sirtuin signaling, and TGF-β signaling, as well as several other pathways, including protein ubiquitination, oxidative phosphorylation, IL-1, IL-6, and role of JAK1, JAK2 and TYK2 in interferon signaling (JAK) (Figure 2A and Supplemental Table 5). Consistent with these pathways, we have previously noted upregulation of TGF-β and IFN pathways, in microarray-assessed gene expression in whole SSc skin biopsies (15, 16). The IFN pathway genes that positively correlated with the mRSS included STAT1 (Supplemental Table 5), and IFN is known to upregulate expression of STAT1 mRNA via STAT1 phosphorylation (17). The JAK pathway indicated that both STAT1 and STAT2 expression correlated with the mRSS (Table 1 and Supplemental Table 5).

TGF-β pathway genes included 2 of the 3 TGF-β isoforms: TGF-β1, TGF-β3, and both TGF-β receptors, TGF-βR1 and TGF-βR2, all correlated highly with the mRSS; TGF-β2 did not correlate with the mRSS (Table 1). Examining previous bulk skin mRNA gene expression, we noted that TGF-β3 correlated highly and TGF-β1 weakly but positively with the mRSS, while TGF-β2 correlated negatively with the mRSS (Supplemental Figure 6) (14). TGF-β3 expression correlated most highly with the mRSS in SFRP2/DPP4 fibroblasts (*R* = 0.68), which include myofibroblasts (Supplemental Table 6).

Several other pathways showed inconsistent pathway regulation, (i.e., positive correlations with gene expression expected to decrease or negative correlations with gene expression expected to increase) and are hereafter referred to as mixed pathways (Supplemental Table 5). The senescence and sirtuin signaling pathways were mixed pathways, with 15/75 (20%) and 29/50 (58%) genes regulated in the opposite manner to that expected in an activation of the pathway. The mixed effects of the senescence signaling pathway in SFRP2/DPP4 fibroblasts were particularly reflected in cyclin dependent kinase inhibitor 2B (CDKN2B, p15INK4b) and TP53 (p53), genes inhibiting proliferation, and CDK1, CDK2, CDK4, and CDK6 (cyclin-dependent kinases), promoting cell division. IPA of MYOC/CCL19 fibroblast gene expression correlating with the mRSS revealed several prominent pathways: senescence, hepatic fibrosis signaling, IL-6 signaling, STAT3 signaling, TGF-β signaling, protein ubiquitination, JAK/Stat signaling, and role of JAK1, JAK2 and TYK2 in interferon signaling. IFN pathway genes included STAT1 and STAT2, as well as JAK2, a direct target of tofacitinib (Figure 2B). The TGF-β signaling pathway, for SFRP2/DPP4 fibroblasts, showed significant correlations between the mRSS and expression of TGFB1, TGFB3, TGFBR1, and TGFBR2 (Table 1).

The senescence signaling pathway was again regulated in MYOC/CCL19 fibroblasts, as in SFRP2/DPP4 fibroblasts, showing a mixed pattern, 16/69 (23%) genes regulated in the opposite-than-expected direction. However, expression of several key senescence pathway genes in these fibroblasts correlated highly with the mRSS, most notably, cyclin dependent kinase inhibitor 1A (CDKN1A, p21Cip1), CDKN2A (p16INK4A), CDKN2B, and TP53 without the coregulated expression of cyclin genes seen in SFRP2/DPP4 fibroblasts. P53, the TP53 gene product, links DNA damage to cell cycle arrest through p21, the gene product of CDKN1A (18).

### Tofacitinib inhibits IFN-regulated gene expression by SSc SFRP2/DPP4 fibroblasts.

**Figure 3:**
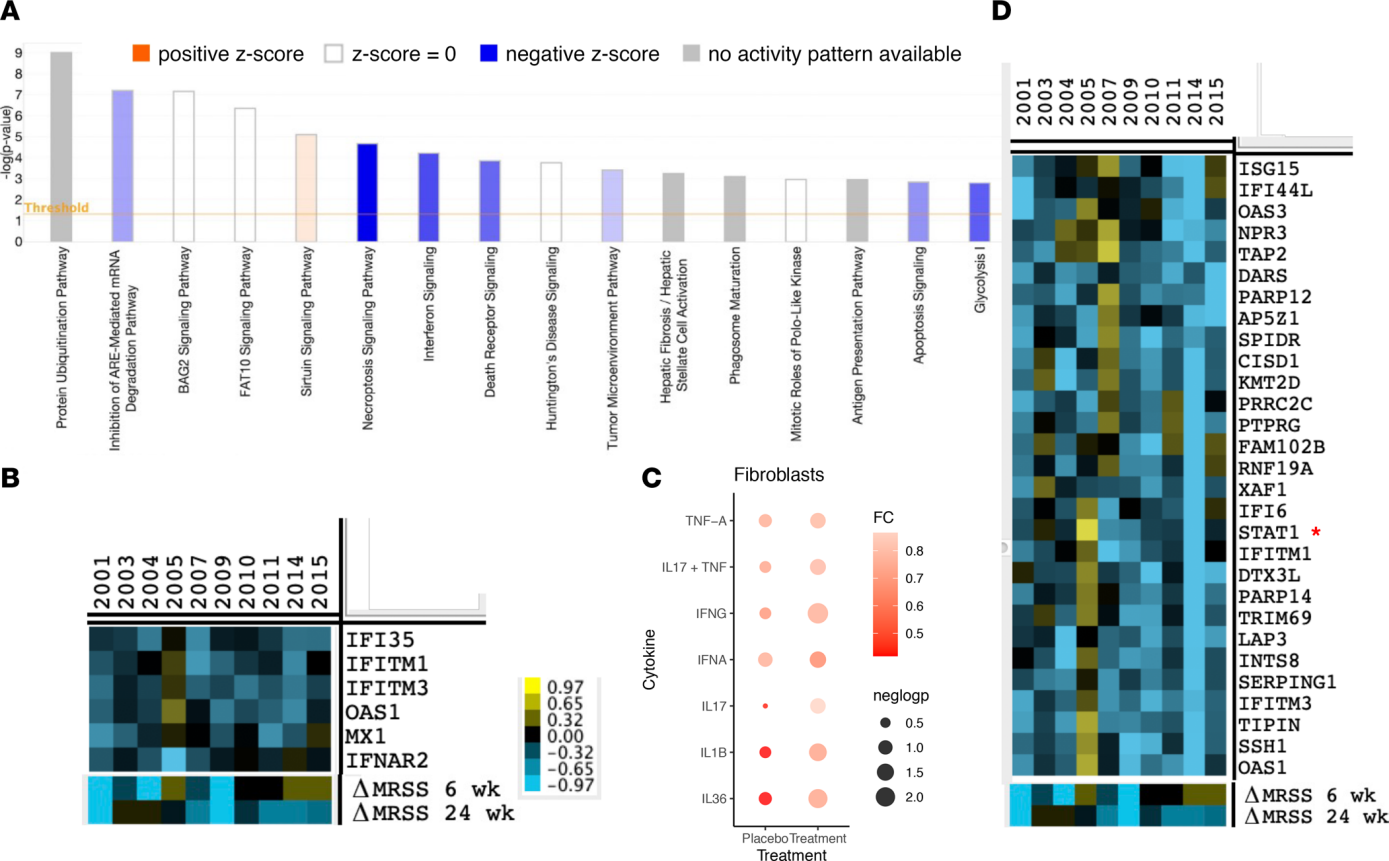
Genes and pathways changing in tofacitinib-treated patient SFRP2/DPP4 fibroblasts. Pathway analysis of scRNA-Seq data from tofacitinib-treated baseline compared with week 6 gene expression (*n* = 10) by SFRP2 fibroblasts (clusters 1 and 9; **A**). Average gene expression in these clusters (pseudo-bulk gene expression) showing decreased expression at week 6 compared with baseline were included in the IPA (uncorrected *P* < 0.05). Only selected significant pathways (–log *P* < 1.4) are indicated. IPA used right-sided Fisher’s exact test to calculate the significance scores (shown on the *y* axis). Blue bars indicate pathways downregulated (*z* score less than –2), orange bar upregulated (*z* score greater than 2), white bars without direction of regulation, and gray bars with no expected direction of regulation after tofacitinib compared to baseline. The intensity of the shading indicates the level of the *z* score. Heatmap of gene expression of the genes associated with the IFN pathway seen in **A** (**B**). Changes in inflammatory gene signatures at week 6 compared with baseline in the placebo and tofacitinib groups for SFRP2/DPP4 fibroblasts (**C**). Clustering of changes in pseudo-bulk gene expression in SFRP2/DPP4 fibroblasts at week 6 compared with baseline in tofacitinib-treated participants of all (filtered) genes (**D**), showing IFN-regulated genes clustering with STAT1 (indicated by a red star).

After establishing fibroblast gene expression pathways correlating with the mRSS at baseline in SSc, we examined pathways downregulated after tofacitinib treatment, examining the change in expression of genes at week 6 compared with baseline. We focused on the pathways described above correlating at baseline with the mRSS and, thus, more likely involved in promoting skin fibrosis. IPA of SFRP2/DPP4 fibroblasts showed that tofacitinib regulated multiple pathways, including protein ubiquitination, death receptor, glycolysis, necroptosis and sirtuin signaling pathways (Supplemental Table 6), but most notably, regulated the interferon signaling pathway (Figure 3A). IPA indicated 6 genes in the interferon response pathway decreased after tofacitinib treatment (Figure 3B). To determine the effect of tofacitinib on SFRP2/DDP4 fibroblasts, we examined the changes in cytokine signature burden; among the cytokines we examined, only the signature of IFNG was significantly (*P* ≤ 0.01) downregulated in the treatment group at week 6 (Figure 3C).

To look more broadly at the effect of tofacitinib on SFRP2/DPP4 fibroblast gene expression, we hierarchically clustered the difference between week 6 and baseline gene expression and detected a cluster of downregulated genes that included STAT1 (Figure 3D). Remarkably, expression of 18/30 genes in this cluster correlated with baseline mRSS (Supplemental Table 7), and this cluster showed 8 genes in the Gene Ontology (GO) term (19, 20) cellular response to type I IFN, of which 5 were not detected in the IPA (XAF1, IFI6, OAS3, STAT1, and ISG15). Finally, to examine possible more subtle relationships between tofacitinib treatment and gene expression, we hierarchically clustered SFRP2/DPP4 gene expression and samples by changes in the mRSS at 6 or 24 weeks (Supplemental Figure 7). Examining the genes most closely associated with the mRSS change at 24 weeks, revealed leucine rich repeat containing G protein-coupled receptor 5 (LGR5), a marker gene of SSc fibroblasts (21), IL-6; and PDGFRA (22, 23), implicated in SSc pathogenesis. LGR5 has recently been implicated as a marker gene. Examining the genes most closely associated with the mRSS change at week 6 did not reveal any pathways.

### Tofacitinib inhibits IFN-regulated gene expression by SSc MYOC/CCL19 fibroblasts.

**Figure 4:**
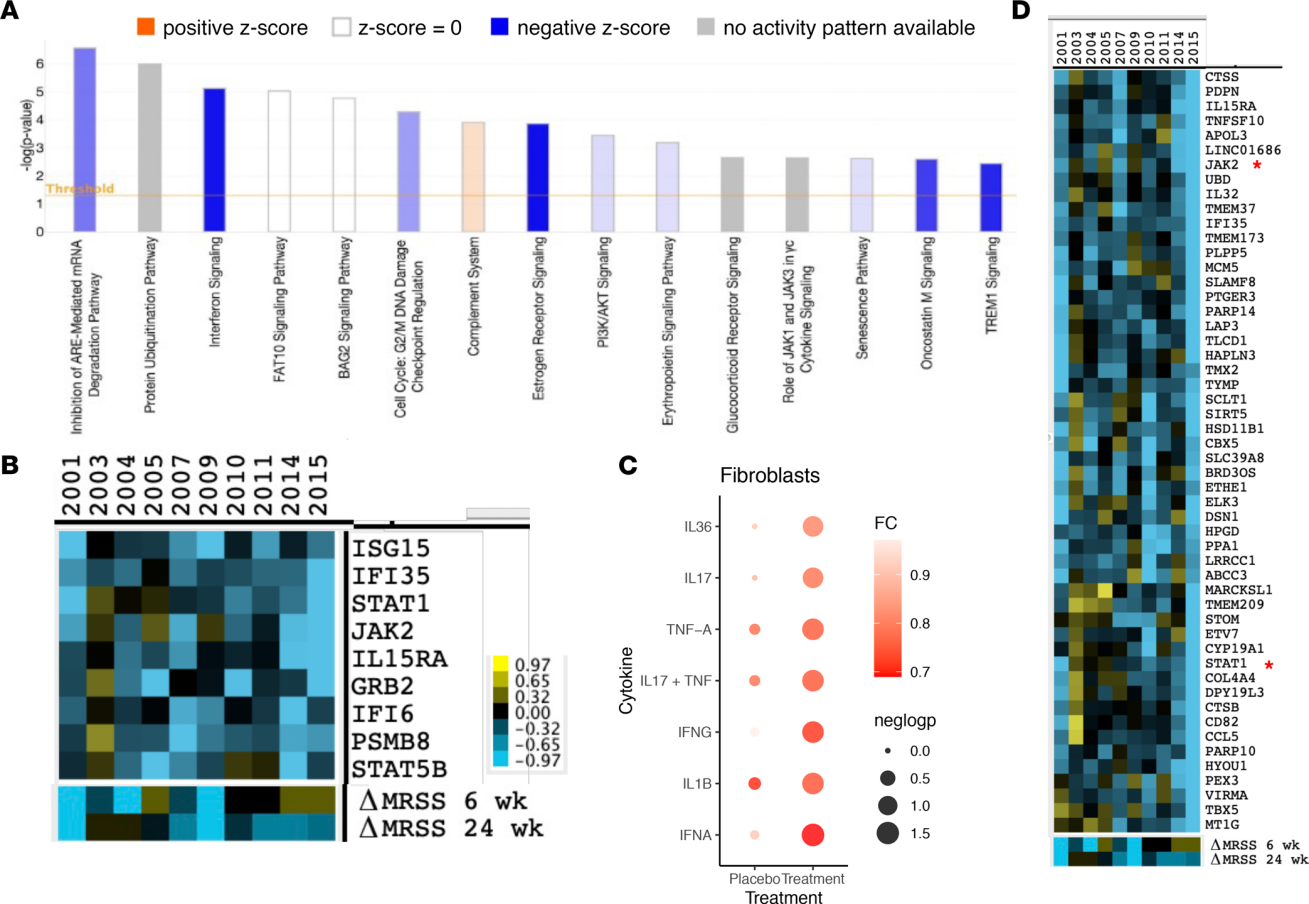
Genes and pathways changing in tofacitinib-treated patient CCL19/MYOC fibroblasts. Pathway analysis of scRNA-Seq data from tofacitinib-treated patients at week 6 compared with baseline gene expression (*n* = 10) by CCL19/MYOC fibroblasts (clusters 6 and 7; **A**). Average gene expression in these clusters (pseudo-bulk gene expression) showing decreased expression at week 6 compared with baseline were included in the IPA (uncorrected *P* < 0.05). Only selected significant pathways (–log *P* < 1.4) are indicated. IPA used right-sided Fisher’s exact test to calculate the significance scores (shown on the *y* axis). Blue bars indicate pathways downregulated (*z* score less than –2), orange bar upregulated (*z* score greater than 2), white bars without direction of regulation, and gray bars with no expected direction of regulation after tofacitinib compared to baseline. The intensity of the shading indicates the level of the *z* score. Heatmap of gene expression of the genes associated with the IFN pathway seen in **A** (**B**). Changes in inflammatory gene signatures at week 6 in the placebo and tofacitinib groups for CCL19/MYOC fibroblasts (**C**). Clustering of changes in pseudo-bulk gene expression in CCL19/MYOC fibroblasts at 6 weeks compared with baseline in tofacitinib-treated participants of all (filtered) genes (**D**), showing IFN-regulated genes clustering with JAK2 and STAT1 (indicated by red stars).

We next examined pathways downregulated after tofacitinib treatment, by IPA of MYOC/CCL19 fibroblasts, finding that tofacitinib regulated the interferon and role of JAK1 and JAK3 in cytokine signaling pathways, as well as multiple other pathways, including the protein ubiquitination pathway (Figure 4A). Eight genes in the interferon and JAK1/3 pathway decreased after tofacitinib treatment, including STAT1, STAT5B, and JAK2 (Figure 4B). When we investigated the changes of different cytokine signatures in the placebo and treatment groups at week 6, our results highlighted that the signatures of IFNA and IFNG were the most downregulated (*P* < 0.05); such an observation was absent in the placebo group (Figure 4C).

To look more broadly at the effect of tofacitinib on MYOC/CCL19 fibroblast gene expression, we hierarchically clustered the difference between week 6 and baseline gene expression (Figure 4D). Examining downregulated genes that clustered with STAT1 and JAK2 did not reveal a significant GO pathway. However, podoplanin, a marker for the early transition of SSc fibroblasts (24), and IL-32, a cytokine induced by IFNs (25, 26), were included in this cluster. These and most of the other genes in this cluster correlated highly with the mRSS (Supplemental Table 7). Finally, to examine possible more subtle relationships between tofacitinib treatment and gene expression, we hierarchically clustered MYOC/CCL19 gene expression and samples by changes in the mRSS at 6 or 24 weeks (Supplemental Figure 8). Examining the genes most closely associated with the mRSS change at 24 weeks revealed IFN-regulated genes. Examining the genes most closely associated with the mRSS change at week 6 did not reveal any pathways.

### Tofacitinib inhibits inflammatory responses in SSc epidermis.

**Figure 5:**
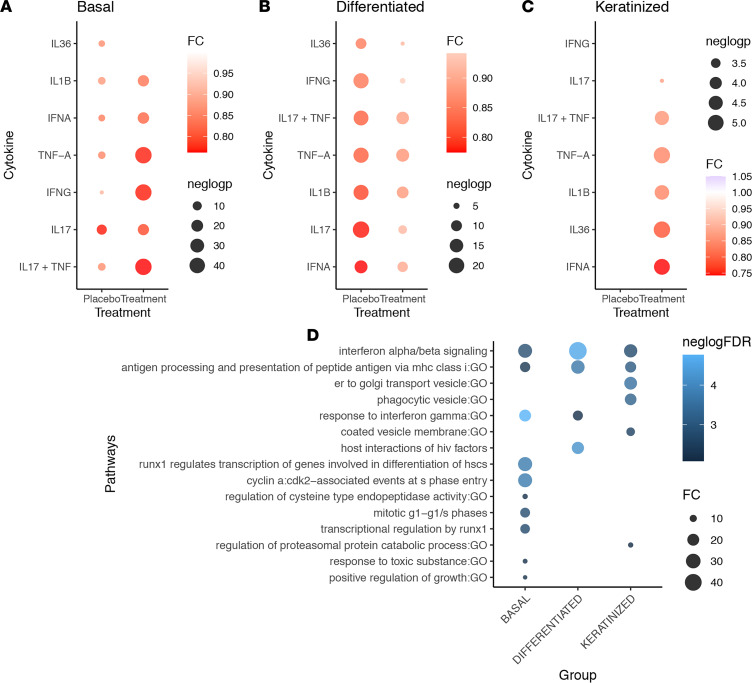
Reduction of type I and type I IFN and other inflammatory signatures by tofacitinib in epidermal keratinocytes. Changes in inflammatory signature by week 6 in the placebo and tofacitinib groups for basal (KRT14), differentiated (KRT10), and keratinized (FLG) epidermal keratinocytes (**A**–**C**). Fold change was computed using median value in baseline versus week 6 groups with *P* value calculated using Wilcoxon rank sum test. (**D**) Dot plot showing the most significantly enriched functions among genes only downregulated in the tofacitinib (but not placebo) group. Only significant results (i.e., FDR ≤ 1%) are shown.

To determine the effect of tofacitinib on SSc epidermal cells, we examined the changes in cytokine signature burden in keratinocyte subpopulations. A total of 17,736 keratinocytes were analyzed and subclustered into basal (KRT14), differentiated (KRT10), and keratinized (FLG) keratinocytes. For each cell, the cytokine signature response was calculated (i.e., type I and type II IFNs). The differences between week 6 and baseline gene expression were calculated for each cytokine signature response in both placebo and tofacitinib groups in each layer of the epidermis; basal, differentiated/spinous, and keratinized/granular layer (Figure 5, A–C). The most robust changes in inflammatory responses were seen in the basal layer and most prominently in the keratinized layer of the epidermis with prominent decrease in both type I and type II IFN responses in the tofacitinib group but not in the placebo (Figure 5D). To confirm these observations, we stratified on genes that were significantly downregulated at week 6 in the tofacitinib group but showed no changes in the placebo group. This approach showed significant enrichment for IFN signaling pathway in all 3 epidermal compartments and revealed changes related to antigen processing and presentation and in keratinocyte proliferation (basal layer) (Figure 5D).

### Gene expression in macrophages indicate tofacitinib inhibition of STAT3.

Examining SSc macrophage cell gene expression at baseline correlating with mRSS showed altered metabolic pathways: oxidative phosphorylation, mitochondrial dysfunction, sirtuin, and protein ubiquitination pathways; and showed dysregulated immune pathways: interferon, phagosome formation and maturation, Fcγ receptor-mediated phagocytosis in macrophages and monocytes, IL-8, TGF-β signaling, Th1, Th2, and Th17 pathways (Supplemental Figure 9A). The IFN pathway was characterized by positive correlations of BAX, IFI6, IFITM1, IFITM2, IFITM3, IRF1, IRF9, ISG15, MED14, MX1, and STAT1 with the mRSS. JAK/STAT signaling pathways were not found in the pathways correlating with the mRSS, but baseline macrophage expression of STAT1 and JAK3 correlated with the mRSS (Table 1). The oxidative phosphorylation, mitochondrial dysfunction, and glycolysis I pathways correlated strongly with the mRSS, suggesting metabolic reprogramming in the macrophage populations, which is consistent with a shift to an M2-like phenotype (27).

Changes in gene expression after tofacitinib in macrophages indicated multiple pathways associated with JAK/STAT activation, similar to those seen as correlating with the mRSS at baseline (Table 1). However, STAT3, but not STAT1, JAK1, or JAK3, was downregulated in macrophages after tofacitinib treatment (Supplemental Figure 9B). Further, none of the IFN-regulated genes upregulated in baseline macrophages were reduced after tofacitinib. However, clustering changes in gene expression comparing tofacitinib with baseline gene expression showed coregulation of STAT3 with PSMB5 (28), ARID5A (29), and CD274 (programmed cell death ligand 1, PD-L1) (30, 31), which are known downstream targets of STAT3 (Supplemental Figure 9B), suggesting that tofacitinib regulates the macrophage phenotype in skin through STAT3, but not by affecting genes dysregulated in SSc macrophages. Upregulated macrophage expression of PD-L1 in SSc skin is likely to have profound effects on SSc skin T cells.

To examine possible more subtle relationships between tofacitinib treatment and gene expression, we hierarchically clustered macrophage gene expression and samples by changes in the mRSS at 6 or 24 weeks (Supplemental Figure 10). Examining the genes most closely associated with the mRSS change at 6 and 24 weeks by GO did not reveal any pathways.

### Gene expression in DCs indicates tofacitinib inhibition of STAT3.

IPA of DC gene expression correlating with baseline mRSS strongly implicated dysregulation of metabolic signaling: oxidative phosphorylation, mitochondrial dysfunction, protein ubiquitination and senescence pathways; JAK/STAT signaling: interferon and STAT3 pathways; and immune functions: antigen presentation, NK signaling, crosstalk between DC and NK, IL-8, and IL-1 signaling were the immune pathways generally showing mixed patterns of regulation (Supplemental Figure 4 and Supplemental Table 5).

Comparing DC gene expression changes after tofacitinib by IPA revealed STAT1 and STAT3 changes in the JAK/STAT/IFN signaling pathways (Supplemental Table 6). However, genes associated with the baseline SSc DC IFN pathway were not downregulated, and genes clustering with STAT1 and STAT3 did not implicate IFN or other signaling pathways. Despite this, genes clustering had STAT3-associated functions — HTRA2, implicated in regulating STAT3 (32), and ADAR regulating editing and splicing of STAT3 (33) — suggesting that STAT3 activity was downregulated in DCs by tofacitinib (Supplemental Figure 4D). NK cell signaling and protein ubiquitination were the only other pathways both correlating with the baseline mRSS and changing after tofacitinib treatment. Relatively few genes were common to both lists, HSPA4 and PSMB8, making it unclear whether these pathways were being affected.

To examine possible more subtle relationships between tofacitinib treatment and gene expression, we hierarchically clustered DC gene expression and samples by changes in the mRSS at 6 or 24 weeks (Supplemental Figure 11). Examining the genes most closely associated with mRSS change at 6 and 24 weeks by GO did not reveal any pathways.

### T cells markers indicate senescence and exhaustion not reversed by tofacitinib.

Examining baseline SSc T cell expression correlating with mRSS showed senescence, interferon, protein ubiquitin, T cell exhaustion, CTLA4 signaling, JAK/Stat, oxidative phosphorylation, mitochondrial dysfunction, and Th1 and Th2 activation pathways. The IFN pathway showed positive correlations of BAX, IFI6, IFI35, IFITM1, IFITM2, IFNG, IRF1, ISG15, and STAT1 expression with the mRSS, as did STAT3, but these genes were not inhibited after tofacitinib treatment. Baseline T cell expression showed both senescence and exhaustion pathways as closely related processes (34). Notably, T cell expression of CDKN2A, EOMES, a transcription factor contributing to T cell exhaustion (35, 36), FOXP1, a transcription factor linked to T cell suppression (37), and LAG3, a checkpoint inhibitor (38), correlated with the mRSS. Of these genes, only expression of CDKN2A changed substantially after tofacitinib, downregulated in T cells from 8 of 10 participants after tofacitinib treatment. Although expression of the Th1 inflammatory cytokine, IFNG, correlated with baseline mRSS, its expression did not change significantly after tofacitinib.

To examine possible more subtle relationships between tofacitinib treatment and gene expression, we hierarchically clustered T cell gene expression and samples by changes in the mRSS at 6 or 24 weeks (Supplemental Figure 12). Examining the genes most closely associated with the mRSS change at 6 and 24 weeks by GO did not reveal any pathways.

### Increased expression of JAK/STAT/IFN-regulated, senescent, and oxidative phosphorylation pathways in endothelial cells unaffected by tofacitinib.

Examining SSc endothelial cell expression correlating with the mRSS showed oxidative phosphorylation, senescence, JAK/Stat, protein ubiquitin, and IFN signaling, with most of the other pathways showing mixed responses. The IFN pathway was characterized by positive correlations of BAK1, BAX, IFI6, IFI35, IFITM1, IFITM2, IFITM3, ISG15, MED14, STAT1, STAT2, and TYK2 with the mRSS. The oxidative phosphorylation pathway stood out, with 43 of 45 genes correlating strongly positively with the mRSS. Enhanced fatty acid oxidation profoundly changes endothelial phenotype, enabling vascular sprouting and endothelial cell proliferation (39). The senescence pathway was mixed with key senescence regulator TP53 increasing, but CDKN1A deceasing, with the mRSS. Despite strong correlations of endothelial cell expression of STAT1, STAT2, and TYK2 with the mRSS, none of the genes in the IFN, JAK/Stat, STAT3, oxidative phosphorylation, or other pathways correlating with the mRSS showed consistent change in gene expression after tofacitinib (not shown).

Finally, baseline expression of pericyte genes correlating with the mRSS implicated a wide array of pathways. Many were mixed pathways and others similar to those seen in other cell types, but particularly relevant to tofacitinib: interferon, STAT3, JAK/Stat, role of JAK2 in hormone-like cytokine, and role of JAK1, and JAK2 and TYK2 in interferon signaling pathways. Both STAT1 and STAT2 and JAK3 correlated positively with the mRSS. However, none of these pathways or other pathways regulated by JAK/STAT were found to be regulated by tofacitinib. Protein kinase A, protein ubiquitination, and senescence pathways were the only pathways identified in pericytes as correlating with the mRSS and inhibited by tofacitinib. In the senescence pathway, CDKN2A and TP53 both correlated strongly positively with the mRSS, but these key senescence genes were not changed after tofacitinib treatment. A subset of ubiquitin pathway genes, HSPA8, PSMB9, PSMB10, PSME2, and UBD, correlated with baseline mRSS and changed consistently with tofacitinib.

### Tofacitinib affects gene expression in the first step of SSc fibroblast differentiation into myofibroblasts.

**Figure 6:**
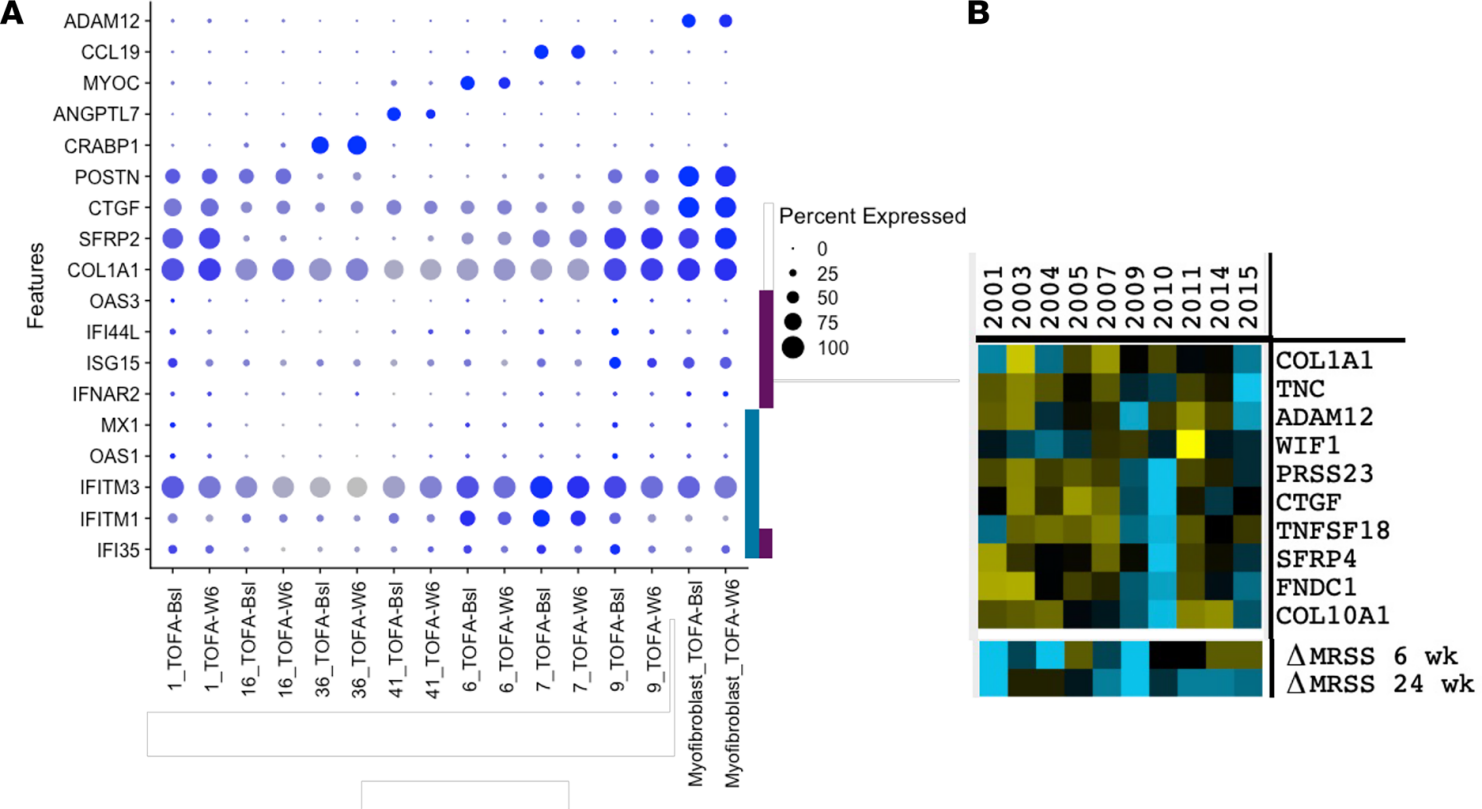
Downregulated expression of IFN-regulated genes after tofacitinib in fibroblast populations. Dot plots showing markers for the SFRP2 (cluster 1 and 9), MYOC (cluster 6), CCL19 (cluster 7), as well as CRABP1 (dermal papilla, cluster 36) and ANGPTL7 (cluster 41) fibroblasts. IFN-regulated genes (IFI35, IFITM1, IFITM3, OAS1, and MX1) decreased after tofacitinib in clusters 1/9 (indicated by aqua bar) and in clusters 6/7 (IFNAR2, ISG15, IFI44L, and OAS3), indicated by maroon bar), but not in myofibroblasts (**A**). The lack of effect of tofacitinib treatment on expression of genes associated with myofibroblast differentiation in individual participants (**B**).

We have recently shown that the process of fibroblast differentiation into myofibroblasts involves 2 steps (14). In the first step, SFRP2-expressing fibroblasts upregulate COL1A1, THBS1, PRSS23, and TNC; in the second step, they upregulate additionally SFRP4, ADAM12, TNFSF18, CTGF, FNDC1, COL10A1, and MATN3 (Figure 6). To better understand the effect of tofacitinib on fibroblast differentiation, we examined baseline IFN-regulated gene expression in fibroblast subpopulations in each of these steps. IFN-regulated genes showed increased expression in all fibroblast subsets, including the first step in fibroblast differentiation into myofibroblasts, but were unchanged in the second step in differentiation to myofibroblasts (Figure 6A). However, tofacitinib did not inhibit expression of other marker genes previously associated with step one or step two in myofibroblast differentiation (COL1A1, PRSS23, THBS2, TNC, SFRP4, ADAM12, TNFSF18, FNDC1, CTGF, or MATN3) (Figure 6B).

## Discussion

Drug development has been difficult in early SSc, partly due to heterogeneous disease course of skin and other organ involvement in early SSc, heterogeneity in molecular expression in the skin, and lack of in-depth work assessing the effect of pharmacologic targets on the pathobiology of SSc (40). In our current phase I/II trial, we show that tofacitinib was well tolerated in early SSc and trended toward improvement in efficacy outcomes. In addition, analysis of scRNA-Seq data presented here indicated that fibroblast and keratinocyte populations were the cell types most profoundly affected by tofacitinib, with minimal impact on T cells and endothelial cells.

Previous murine studies suggest that tofacitinib can affect fibrosis in murine skin by acting on lymphocytes (12). However, many studies have emphasized the importance of JAK/STAT signaling in fibroblasts. Fibroblast STATs are activated through a wide array of signals, including PDGF, IL-6, OSM, and IFNs, with IFN inducing STAT1 and STAT3 phosphorylation in rheumatoid synovial fibroblasts (41, 42). Amelioration of inflammatory arthritis by JAK inhibition may in part reflect effects on synovial fibroblasts. JAK3 is heavily phosphorylated in RA synovium and in synovial fibroblasts (10). Although TNF does not use a JAK/STAT signaling pathway, TNF induction of chemokine secretion was blocked by tofacitinib, a phenomenon attributed to an autocrine loop involving JAK/STAT-dependent type I IFN signaling critical to the TNF response (42). Tofacitinib also suppresses production of RANK ligand (critical for osteoclast activation in RA) by both T cells and synovial cells (43). The profibrotic effect of TGF-β, a critically important cytokine in SSc, was recently reported to occur in part through a JAK2-dependent pathway (44).

The importance of fibroblasts as mediators of fibrosis is well understood, but the role of fibroblast subsets in immune and fibrotic responses in rheumatic disease is emerging, particularly in recent scRNA-Seq studies of rheumatoid synovium and our studies of SSc skin and lungs (14, 45, 46). This scRNA-Seq approach enabled us to examine pathways disrupted in different SSc cell types, including subsets of fibroblasts, and to see which of these pathways were affected by tofacitinib.

IFN appears to be the main target of tofacitinib in SSc, both in keratinocytes and fibroblasts, including the 2 major subsets: SFRP2 fibroblasts, progenitors of myofibroblasts, and MYOC/CCL19 fibroblasts, which include adventitial fibroblasts. We have previously shown that patients with SSc show increased expression of IFN-regulated genes in PBMCs (47) and skin (15), the latter correlating with the mRSS. Other groups have confirmed these observations, making IFN and IFN-regulated genes an important target in SSc. The pattern of IFN gene expression, lacking CXCL9, which is highly regulated by IFN-γ in vitro (48), makes it more likely these genes are responding to type I IFNs, either IFN-β from fibroblasts or IFN-α possibly from plasmacytoid DCs upregulated in SSc skin (49). The genes in both fibroblast populations are coregulated with STAT1, a gene shown to autoregulate its expression, suggesting that tofacitinib is primarily blocking STAT1 in fibroblasts.

The contribution of keratinocytes to SSc pathogenesis is not fully clear, but studies have indicated that keratinocytes may promote fibroblast activation in a TGF-β–independent manner (50). Although the specific role of type I IFN in SSc epidermis is unknown, our data show that keratinocytes respond to the enriched IFN environment in SSc skin (51) and are sensitive markers of suppression of IFN signaling with tofacitinib.

In contrast to fibroblast populations, we found STAT3 was downregulated in myeloid cell populations. Like STAT1, STAT3 autoregulates its expression (51), implicating that tofacitinib inhibits STAT3 signaling in SSc myeloid cells. This is supported by genes coregulated with STAT3, genes known to be regulated by STAT3.

We observed many other pathways activated in SSc skin correlating with the mRSS at baseline but not affected by tofacitinib. These observations point to significant common features of SSc pathogenesis encompassing multiple cell types, such as increased protein ubiquitination (seen in all 8 cell types), oxidative phosphorylation (OxPhos), senescence, and IFN signaling (seen in 7 of 8 cell types). The changes in these pathways may well be linked even though the genes associated with each pathway are largely distinct. The ubiquitin and IFN pathways are linked through ISG15, a ubiquitin-like protein, and USP18, a deubiquitinating enzyme specific for ISG15, both upregulated in SFRP2/DPP4 and MYOC/CCL19 SSc fibroblast populations and downregulated in tofacitinib-treated SFRP2^+^ (but not CCL19^+^) fibroblasts (52). USP18 and ISG15 mRNA expression are upregulated through Jak1/Tyk and Stat1/Stat2 signaling. USP18 also regulates IFN signaling independently of ISG15.

Senescence contributes importantly to lung fibrosis (53) and is associated with mitochondrial dysfunction (54) but also with OxPhos (55). Senescent cells secrete proinflammatory and profibrotic molecules referred to as senescence-associated secretory phenotype (56–58). Altered cell metabolism associated with aging and cellular stress have been implicated in SSc pathogenesis (59, 60). SSc dermal fibroblasts show evidence indicative of cellular senescence (59). Metabolic reprogramming of fibroblasts also has been shown to be important in differentiation of lung myofibroblasts (61, 62). Glycolysis, fatty acid oxidation, and OxPhos are upregulated in alveolar macrophages from TGF-β and bleomycin murine fibrosis (63). Glycolysis appears to be the profibrotic metabolic pathway in both of these cell populations. Our data indicate that interconnected pathways linking senescence and OxPhos are acting across multiple cell types in SSc skin, promoting fibrosis.

The strengths of our study include in-depth scRNA-Seq in the skin tissue in a setting of a double-blind placebo-controlled trial providing insights into pathophysiology of SSc and mechanism of action of tofacitinib.

The limitations include the small sample size and lack of correction for multiple comparisons in the transcriptomic analysis.

In conclusion, we did not see effects of tofacitinib on genes associated with TGF-β or T cell signaling, but we did see an effect on IFN signaling. One of the potential reasons for the observed lack of impact of tofacitinib on immune pathways may be due to background immunosuppressive therapy. In view of the difficulty of finding effective treatment for SSc, combined therapies may be required to suppress altered SSc fibroblast differentiation. Thus, tofacitinib might be particularly useful in combination with an inhibitor of other fibroblast signals, such as TGF-β, or with T cell–targeted therapy, such as abatacept or romilkimab.

## Methods

### Study design.

This was a phase I/II, investigator-initiated, randomized, double-blind, placebo-controlled trial of tofacitinib (5 mg twice a day) versus placebo in 2:1 ratio in patients with dcSSc (ClinicalTrials.gov NCT03274076). dcSSc was defined as skin thickening, proximal as well as distal, to the elbows or knees with or without involvement of the face and neck at the time of study entry. Study participants were treated for 24 weeks on double-blind study medication and were offered an additional 6 months of open-label daily oral tofacitinib therapy. The sponsor, DK, received an Investigational New Drug exemption from the Food and Drug Administration.

### Study participation criteria.

Key inclusion criteria were adult participant, age 18 and older; classification of SSc, as defined using the 2013 American College of Rheumatology/European Union League Against Rheumatism classification of SSc (64), and dcSSc, as defined by LeRoy and Medsger Jr (65); disease duration of ≤60 months (defined as time from the first non−Raynaud phenomenon manifestation); and mRSS units ≥ 10 and ≤ 45 at screening. Varicella-zoster vaccination was provided, or the participant had received vaccination prior to screening. Stable-dose background immunosuppressive therapy, such as methotrexate ≤ 25 mg/w or mycophenolate mofetil ≤ 2 g/d, was allowed if on a stable dose for at least 12 weeks. Oral corticosteroids (≤10 mg/d of prednisone or equivalent) and NSAIDs were permitted if the patient was on a stable-dose regimen for ≥2 weeks. More details of the inclusion and exclusion criteria are listed in the study protocol (available from the corresponding author).

### Randomization and masking.

Eligible participants were randomized in a 2:1 ratio to either 5 mg twice a day of tofacitinib or matching placebo (provided by Pfizer Inc.). A randomization schedule using computer-generated block randomization with the random block sizes (known only by the statistician) was used to randomize patients. The study staff, including the research pharmacists, and participants were blinded to the treatment assigned.

### Procedures.

Participants were seen at regular intervals throughout the 24-week study period. Study assessments and their timing are summarized in the study protocol (available from the corresponding author). All participants who had not received the varicella-zoster vaccination prior to study participation followed the timeline indicated below considering whether or not they were on background immunosuppressive therapy. Participants on background therapy were asked to temporarily hold the therapies for 14 days, receive the varicella-zoster vaccination, and wait another 14 to restart the background medication, then 28 days later continued to randomization. The screening window was up to 65 days to ensure these steps were completed. Eligible participants were assessed at baseline; at week 6, 12, 18, and 24 during the double-blind phase; and at weeks 30, 36, and 48 during open-label extension. Patients who did not continue into the open-label period had follow-up via phone 30 days after their last dose.

### Outcomes.

The primary study endpoint was the proportion of participants who experienced grade 3 (severe) or higher AEs, as defined by the CTCAE v 4.03, that occurred at or before week 24. The secondary study endpoints included number of grade 2 (moderate) or grade 3 (severe) or higher AEs that occurred at or before weeks 12, 36, and 48; number of AEs of special interest at weeks 12, 24, 36, and 48; change in the mRSS at weeks 12, 24, 36, and 48; and provisional ACR-CRISS at weeks 12, 24, and 48.

The study was overseen by a Data and Safety Monitoring Committee (University of Michigan) that reviewed study conduct and safety outcomes approximately every 6 months.

### scRNA-Seq.

A skin biopsy (3 mm) of the involved forearm skin was performed on each participant, at baseline and at week 12. Biopsies were stored in RNA*later* (Thermo Fisher Scientific), and fresh skin tissue was transferred to the University of Pittsburgh on the same day. The scRNA-Seq data were submitted to Gene Expression Omnibus database, accession GSE209635.

### Gene-skin correlation.

Gene expression values obtained using scRNA-Seq were analyzed in different cell types: CCL19/MYOC (cluster 6 and 7) fibroblasts, SFRP2/DPP4^+^ (cluster 1 and 9) fibroblasts, macrophages, pericytes, T cells, DCs, and NK cells. Correlation between the mRSS and baseline gene expression values obtained prior to and after treatment in the placebo and only prior to treatment in the tofacitinib-treated groups were analyzed.

Pearson’s correlation coefficient (*R*) was calculated to determine which genes had the strongest relationship to the mRSS. The *P* value for each correlation was calculated from a 2-tailed Student’s *t* distribution to determine its statistical significance. Genes with *P* values less than 0.05 were included in the IPA.

### IPA.

QIAGEN IPA Core Analysis was performed using genes and their associated correlations with the mRSS for each cell population, using values of gene expression from all 15 baseline samples from the tofacitinib trial as well as samples previously reported from 12 patients with dcSSc and 10 controls (14). These gene sets were combined using the sctransform function in Seurat, which uses molecular anchors and canonical correlation analysis to integrate data sets (66). We used this combined data set to correlate gene expression values for each cell population with the associated mRSS, calculating the Pearson’s correlation coefficient (*R*). Genes in each cell population showing correlations with the mRSS with *P* values less than 0.05 were analyzed using the IPA Core Analysis, generating statistically significant pathways. Pathways derived with –log *P* values of 1.3 or greater (*P* < 0.05) were considered significant.

IPA was also used to determine pathways and their associated genes comparing week 6 to baseline gene expression in cell populations in tofacitinib-treated patients, using a paired *t* test with 2-tailed distribution. Genes with uncorrected *P* values less than 0.05 were included in an IPA Core Analysis. Pathways derived with –log *P* values of 1.3 (*P* < 0.05) or greater were considered significant. Certain pathways and associated genes of theoretical and analytical significance were then further analyzed by hierarchical clustering.

### Hierarchical clustering.

Hierarchical gene clustering was carried out, analyzing the difference between week 6 and baseline gene expression, hierarchically clustering samples showing at least 8 observations having absolute value greater than 0.01, using Cluster 3.0. Normalized expression was clustered by Euclidean distance and complete linkage and visualized by Java Treeview. GO analysis was carried out using the online Gene Ontology resource (http://geneontology.org/) (19, 20).

### Statistics.

This phase I/II study was sized primarily based on practical considerations rather than a desired power for a prespecified difference. The main analysis set for efficacy was the modified intention to treat (mITT) population, defined as all randomized participants who received at least 1 dose of study medication. The safety population, defined equivalently to the mITT set, was used for all safety analyses.

We analyzed baseline and demographic characteristics by treatment group for participants who entered the double-blinded period and open-label extension. We reported means and standard deviations for continuous variables and counts and percentages for categorical variables. As for safety outcome, we counted the number of treatment-emergent AEs during the double-blinded period and the open-label extension by body system. We calculated change from baseline in the following variables: mRSS, patient global assessment, physician global assessment, Health Assessment Questionnaire-Disability Index, forced vital capacity percent predicted, and calculated ACR-CRISS at week 24 (end of double-blinded period) and week 48 (end of open-label extension). Medians and IQRs were reported for these variables by treatment group. Group differences in medians were calculated, corresponding 95% CIs were obtained via bootstrapping, and corresponding *P* values were obtained via Wilcoxon rank sum test. We also fitted a linear mixed model for change in mRSS, adjusted for baseline mRSS, study week, treatment group, interaction of baseline mRSS and study week, and interaction of study week and treatment. We obtained least square means (LSM) and corresponding 95% CI at each time point by treatment group from the model and plotted these values in a figure. Most analyses were conducted in SAS (version 9.4), and LSM figure was plotted in R Studio.

### Study approval.

IRBs from the University of Michigan and University of Pittsburgh approved the study protocol (available from the corresponding author) before research commenced. The study was conducted in accordance with the Declaration of Helsinki and Good Clinical Practice. Written informed consent was received prior to the individual’s participation.

## Author contributions

DK contributed to design and acquisition and interpretation of data; wrote the first draft, and approved the final manuscript version. CP, LCT, VN, PPK, TT, JMK, and AY contributed to acquisition and interpretation of data, revised the manuscript for critically important intellectual content, and approved of the final manuscript version. SH contributed to analysis and interpretation of data, revised the manuscript for critically important intellectual content, and approved of the final manuscript version. JEG and DAF contributed to acquisition and interpretation of data, performed experiments, revised the manuscript for critically important intellectual content, and approved of the final manuscript version. RL contributed to acquisition and interpretation of data, performed experiments, wrote the first draft with DK, and approved of the final manuscript version.

## Supplementary Material

Supplemental data

ICMJE disclosure forms

Supplemental table 5

Supplemental table 6

Supplemental table 7

## Figures and Tables

**Table 1:**
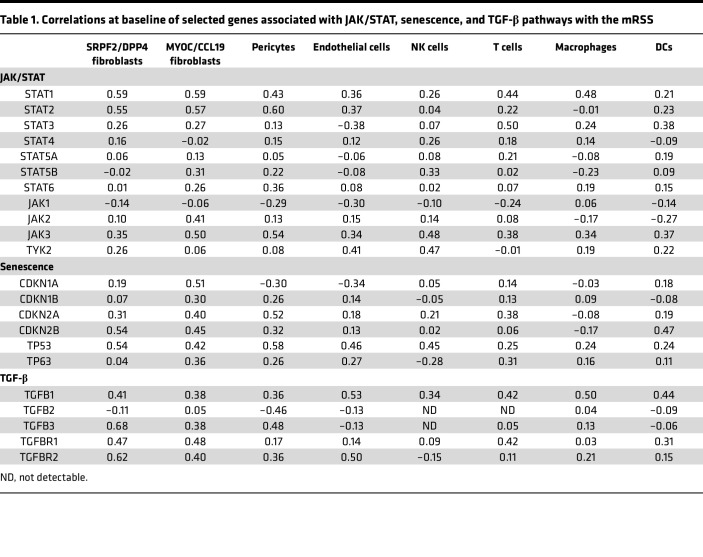
Correlations at baseline of selected genes associated with JAK/STAT, senescence, and TGF-β pathways with the mRSS

## Version 1 08/09/2022

In-Press Preview

## Version 2 09/08/2022

Electronic publication
