# Supplementary material for: Tofacitinib blocks IFN-regulated biomarker genes in skin fibroblasts and keratinocytes in a systemic sclerosis trial
Source: JCI Insight. 2022 Sep 8;7(17):e159566. doi: 10.1172/jci.insight.159566 (PMC9536259; doi:10.1172/jci.insight.159566)
Supplement: Supplemental data [file jciinsight-7-159566-s206.pdf]

Supplementary Materials

Fig. S1. CONSORT diagram for the clinical trial

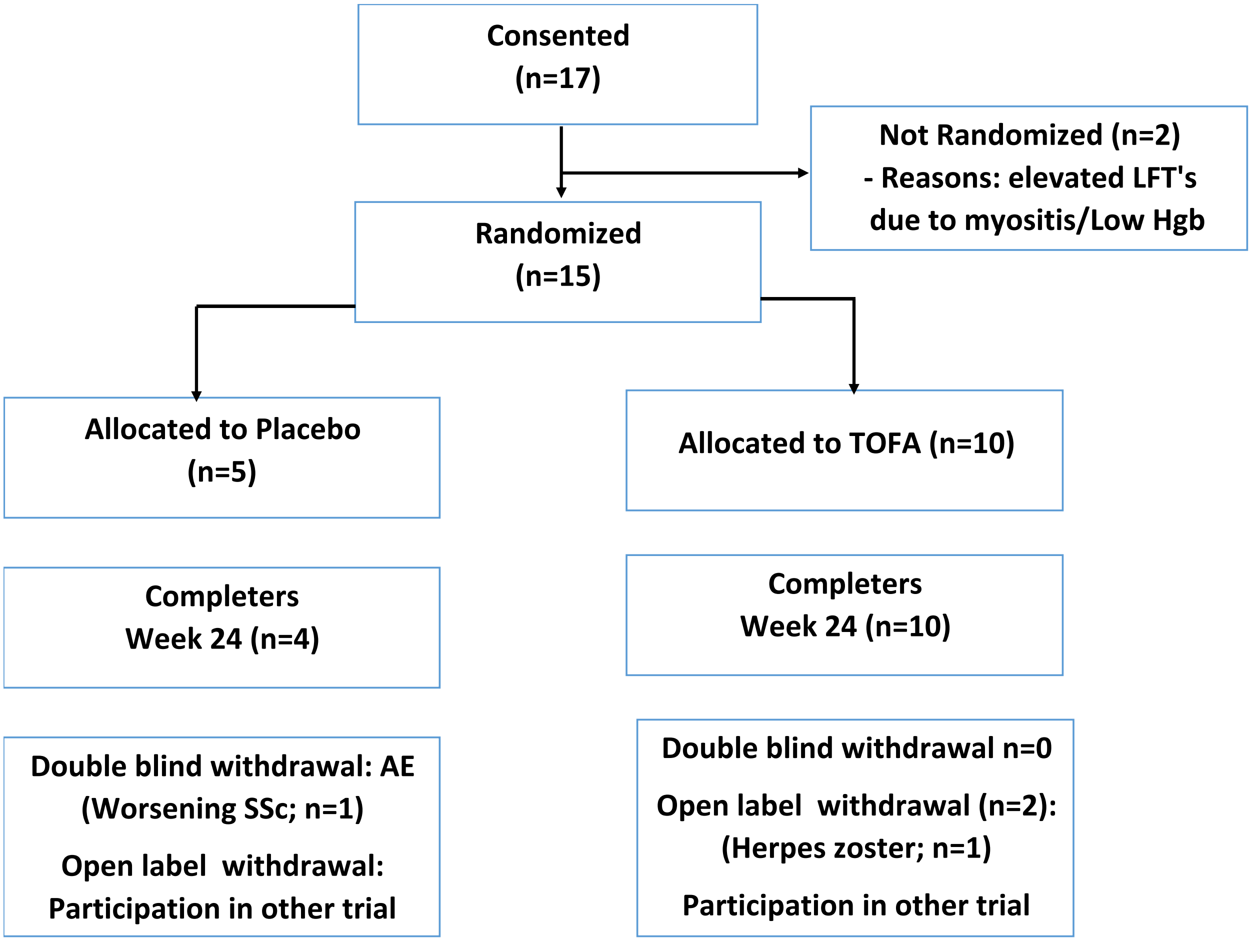

Fig. S2. Mean Trend Over Time: Change in the modified Rodnan skin score during the double blind and open label phases

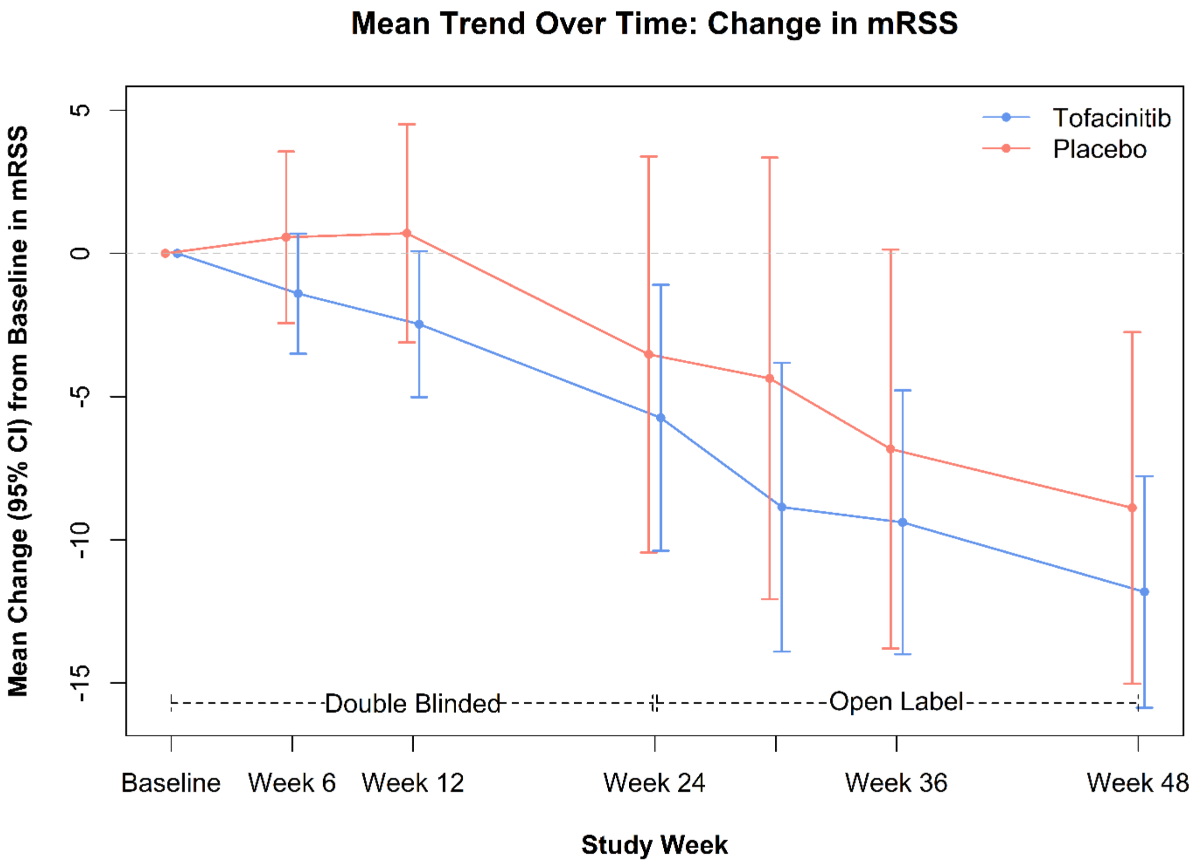

Fig. S3-T-SNE plot as in Figure 1, with cells colored by skin biopsy sample (Panel A), or by patient group (placebo patient baseline biopsies, PBO-Bsl; Placebo patient biospies at Week 6, PBO-W6; tofacitinib treated patient baseline biopsies, TOFA-Bsl; tofacitinib treated patient Wk 6 biopsies, TOFA-W6).

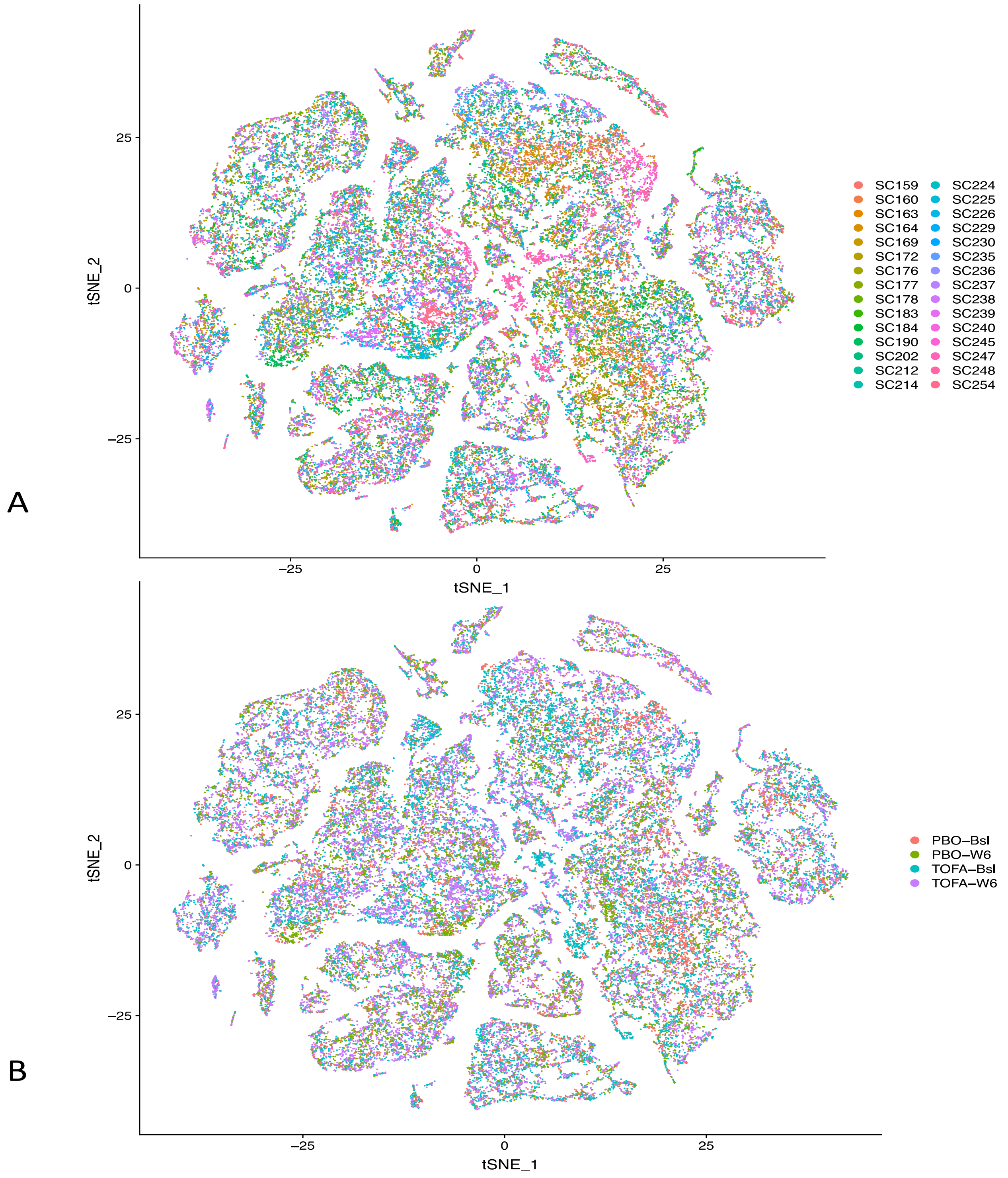

Fig. S4- Marker genes for each cell population, numbers on the x axis indicate cluster number as in Figure 1.

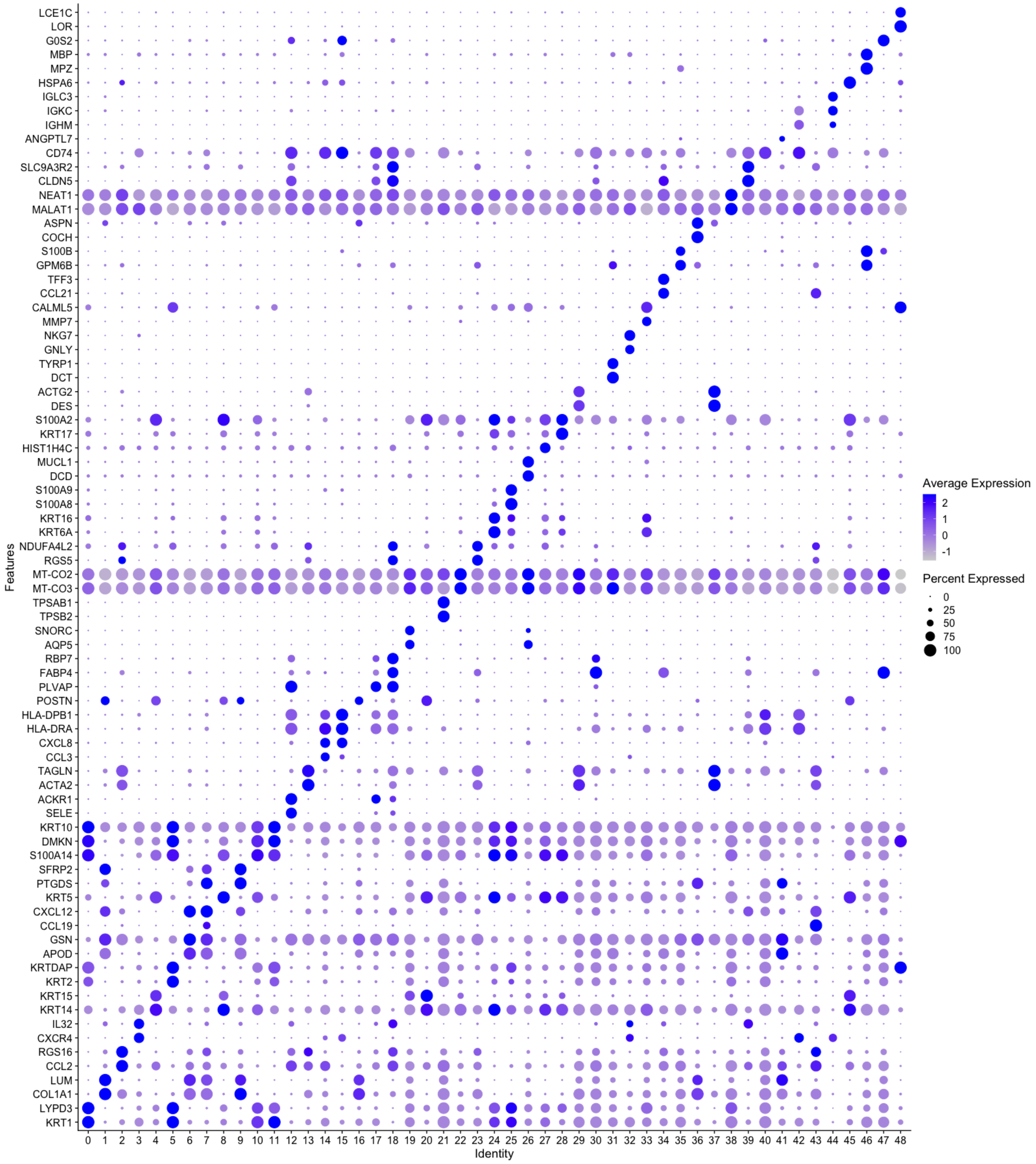

Cluster number	Average number of cells/cluster
0-Terminal Keratinocyte	103.5483871
1-PI16 Fibroblast	89.16129032
2-Pericyte	87.09677419
3-T Cell	82.32258065
4-Basal Keratinocyte	81.67741935
5-Terminal Keratinocyte	80.83870968
6-MYOC Fibroblast	77.67741935
7-CCL19 Fibroblast	77.06451613
8-Basal Keratinocyte	76.38709677
9-SFRP2 Fibroblast	74.74193548
10-S100A14 Keratinocyte	67.12903226
11-Terminal Keratinocyte	61.09677419
12-Venous Endothelial	56.96774194
13-RERGL Pericyte	54.16129032
14-Macrophage	54.03225806
15-Dendritic Cell	51.87096774
16-DPEP1 Fibroblast	47.09677419
17-Endothelial	42.5483871
18-Endothelial/Pericyte Doublet	42.19354839
19-CA6 Secretory	36.35483871
20-KRT15 Keratinocyte	35.48387097
21-Mast Cell	35.29032258
22-Dead Keratinocyte	30.96774194
23-STEAP4 Pericyte	29.83870968
24-KRT6A Keratinocyte	27.77419355
25-S100A8 Keratinocyte	27.74193548
26-SCGB1B2P Secretory	27.4516129
27-Proliferating	23.4516129
28-KRT17 Keratinoycte	22.4516129
29-Low Quality Smooth Muscle	20.67741935
30-Low Quality Endothelial	19.74193548
31-Melanocyte	18.96774194
32-NK Cell	18.51612903
33-KRT6B Keratinocytes	18.5483871
34-Lymphatic Endothelial	18.48387097
35-Neural	18.16129032
36-Dermal Papilla	15.90322581
37-Smooth Muscle	15.25806452
38-Low Quality Cells	14.22580645
39-Arterial Endothelial	11.4516129
40-Low Quality DC	11.38709677
41-ANGPTL7 Fibroblast	10.48387097
42-B Cell	8.064516129
43-CCL21 Pericyte	6.935483871
44-Plasma Cell	6.709677419
45-Stressed Keratinocyte	4.709677419
46-Schwann	3.677419355
47-Adipocyte	3.483870968
48-Cornified Envelope	2.322580645

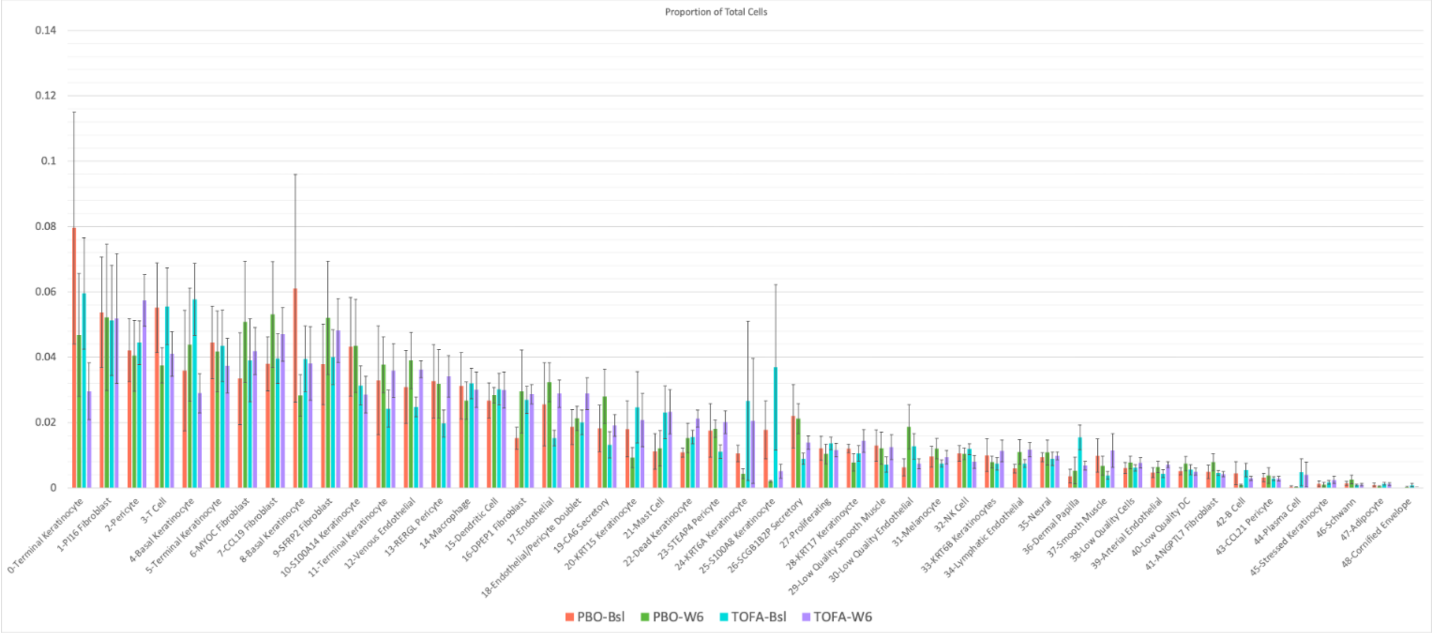

Figure S5. Proportions of cells in each cluster (panel A). Proportions of cells in each cluster by treatment group at baseline and week 6 skin biopsies (panel B). Error bars indicate standard deviation between samples in each group.

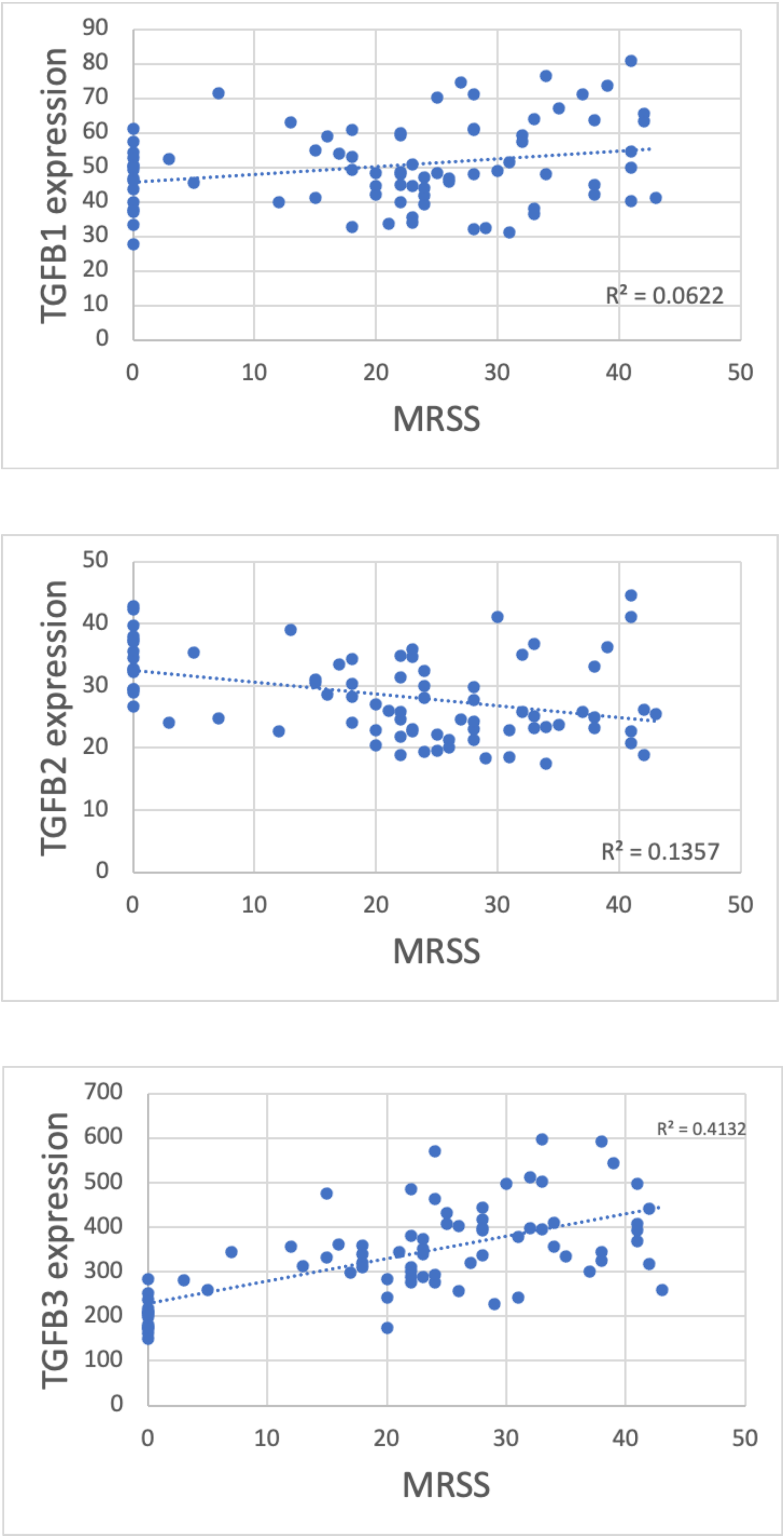

Fig. S6- Bulk skin gene expression from patients with dcSSc, correlations with TGFB isoform expression

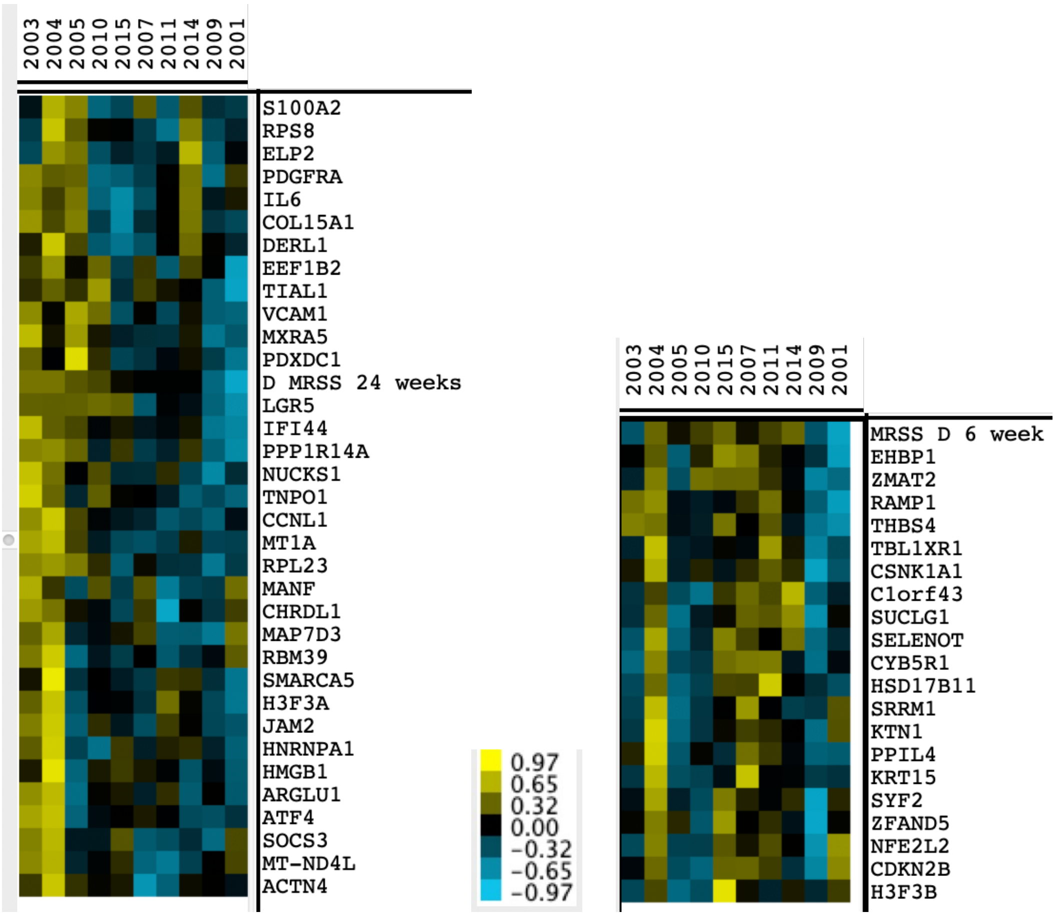

<u>Figure S7. Change in gene expression by cluster of SFRP2/DPP4 fibroblast genes, comparing week 6 to baseline.</u> Normalized change in MRSS is included in the clustering algorithm. Columns are arranged by change in MRSS at 24 weeks with increased (worsening) skin score (yellow) to the left and decreased (improved) skin score (blue) to the right. Genes are shown that cluster with change in MRSS at 24 weeks (D MRSS 24 weeks, left panel) or with change in MRSS at 6 weeks (D MRSS 6 weeks, right panel). Analysis of genes in clusters by Gene Ontology did not indicate and statistically significant Gene Ontology pathways.

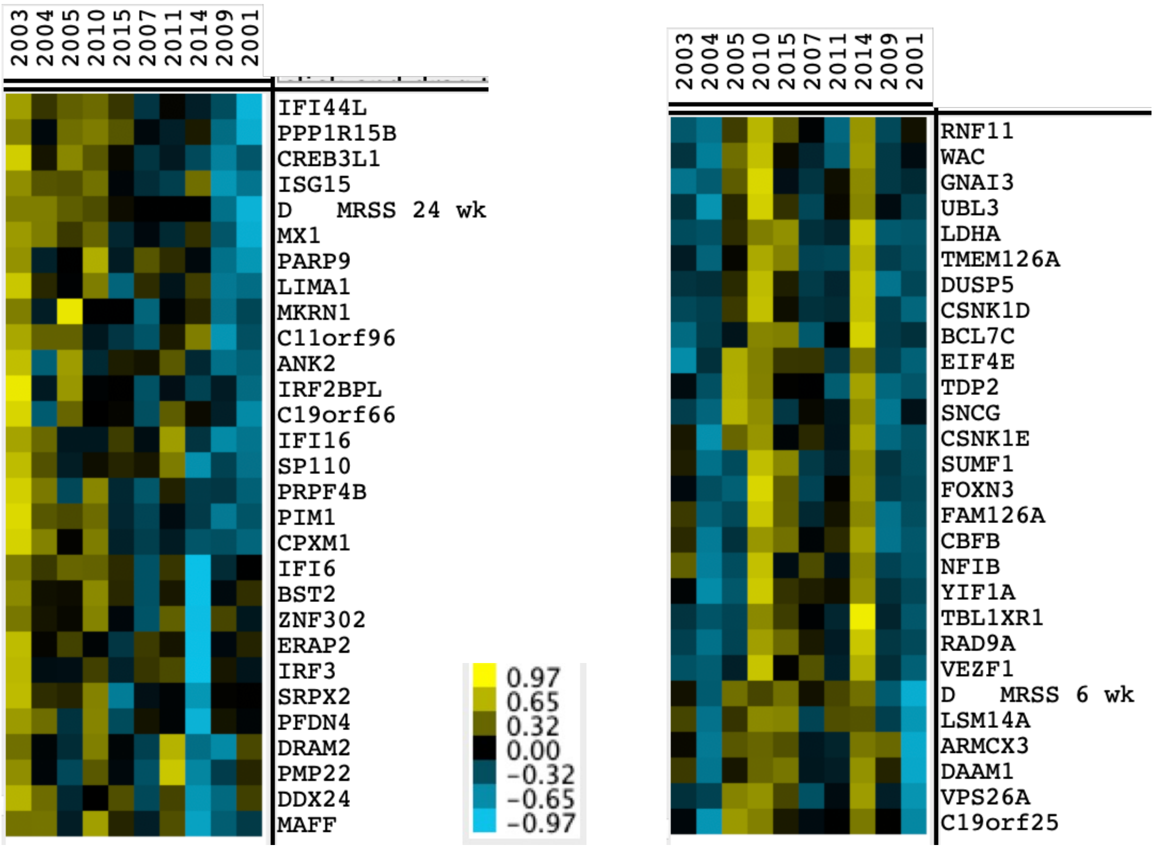

Figure S8. Change in gene expression by cluster of MYOC/CCL19 fibroblast genes, comparing week 6 to baseline. Normalized change in MRSS is included in the clustering algorithm. Columns are arranged by change in MRSS at 24 weeks with increased (worsening) skin score (yellow) to the left and decreased (improved) skin score (blue) to the right. Genes are shown that cluster with change in MRSS at 24 weeks (D MRSS 24 weeks, left panel) or with change in MRSS at 6 weeks (D MRSS 6 weeks, right panel). 24-week cluster genes indicated Gene Ontology pathways: Response to virus and response to type 1 interferon

Fig. S9-Ingenuity Pathway Analysis of scRNA-seq from myeloid populations. Pathway analysis of myeloid scRNA-seq data from baseline study biopsies (n-15), analyzed together with scRNA-seq data from previously described dcSSc (n-12) and healthy skin (n=10). Selected pathways from cluster 14, clustered with analogous cells in previous studies, representing macrophages (panel A) and cluster 15 clustered with analogous cells in previous studies representing DCs (panel C). Genes correlating with baseline MRSS (uncorrected p<0.05) were included in the pathway analysis. Only selected significant pathways (-log(p-value) <1.4) are indicated. Yellow bars indicate positive associations with IPA expected direction of regulation; blue bars show negative associations with expected direction of regulation; open bars indicate no associations with IPA expected direction of regulation; and grey bars indicate no expected direction of regulation. Clustering of changes in pseudo-bulk gene expression in macrophages (cluster 14, panel B) and dendritic cells (cluster 15, panel D) at 6-week compared to baseline in tofacitinib-treated patients of all (filtered) genes, showing co-regulated genes clustering with STAT3 (indicated by red stars).

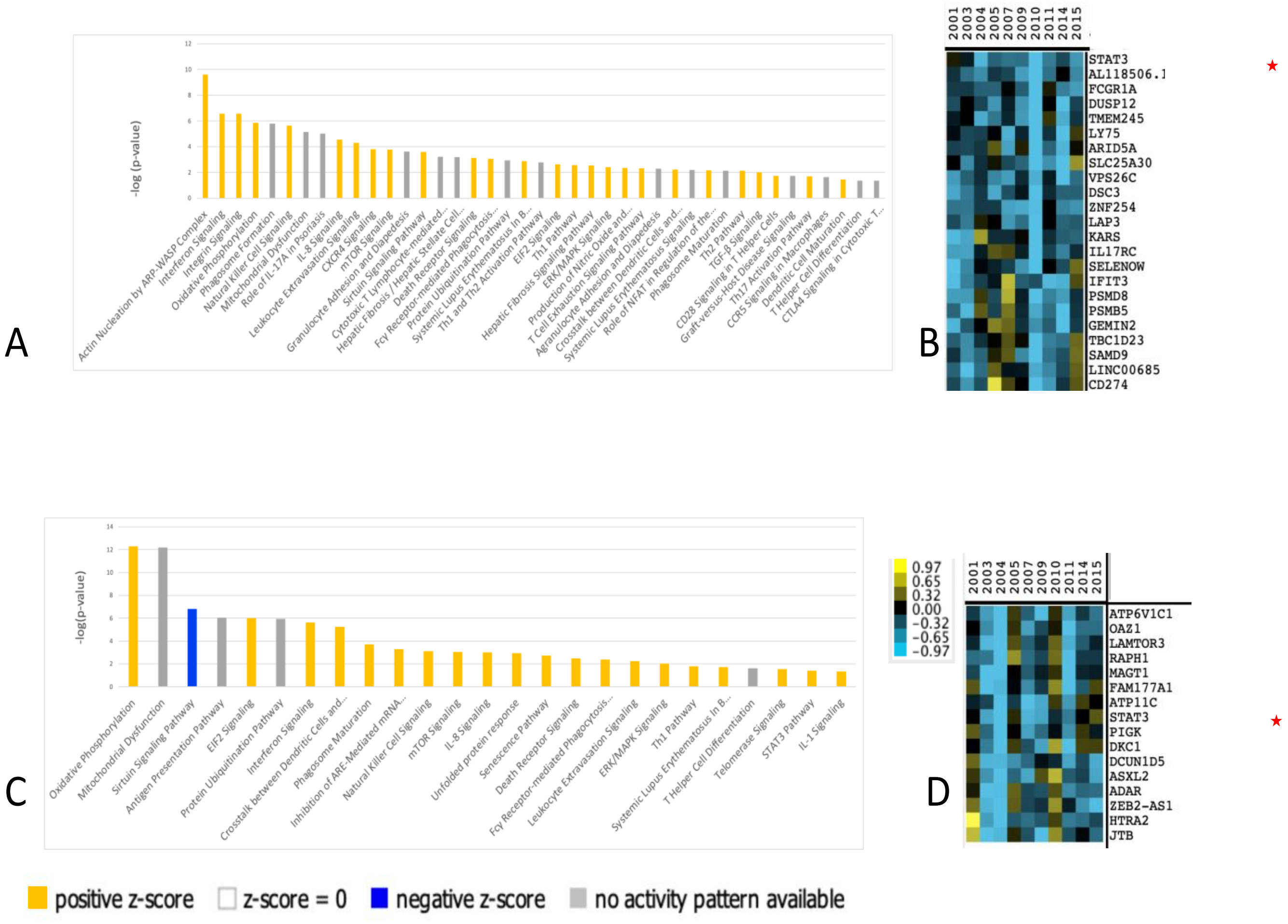

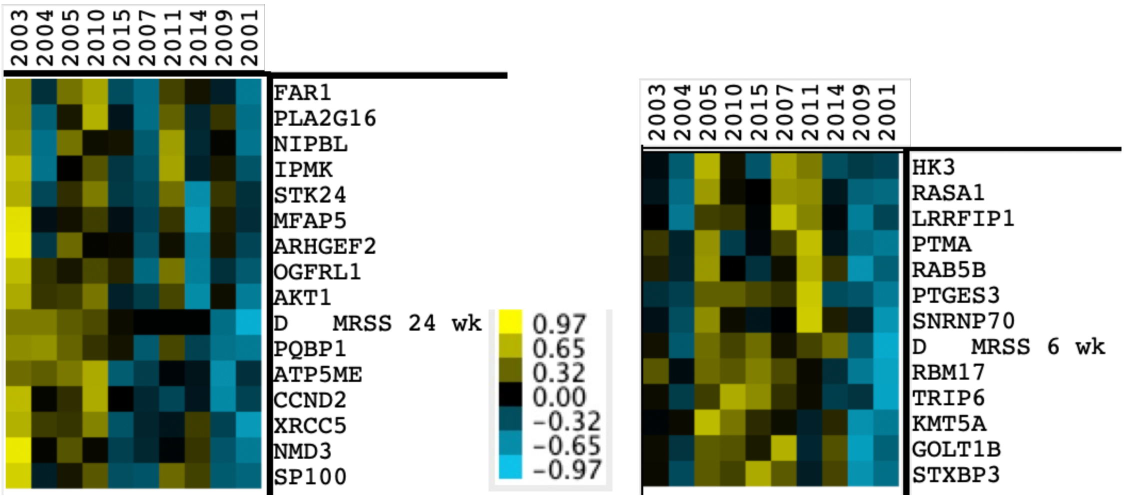

<u>Figure S10. Change in gene expression by cluster of macrophage genes, comparing week 6 to baseline.</u> Normalized change in MRSS is included in the clustering algorithm. Columns are arranged by change in MRSS at 24 weeks with increased (worsening) skin score (yellow) to the left and decreased (improved) skin score (blue) to the right. Genes are shown that cluster with change in MRSS at 24 weeks (D MRSS 24 weeks, left panel) or with change in MRSS at 6 weeks (D MRSS 6 weeks, right panel). Analysis of genes in clusters by Gene Otology did not indicate and statistically significant pathways.

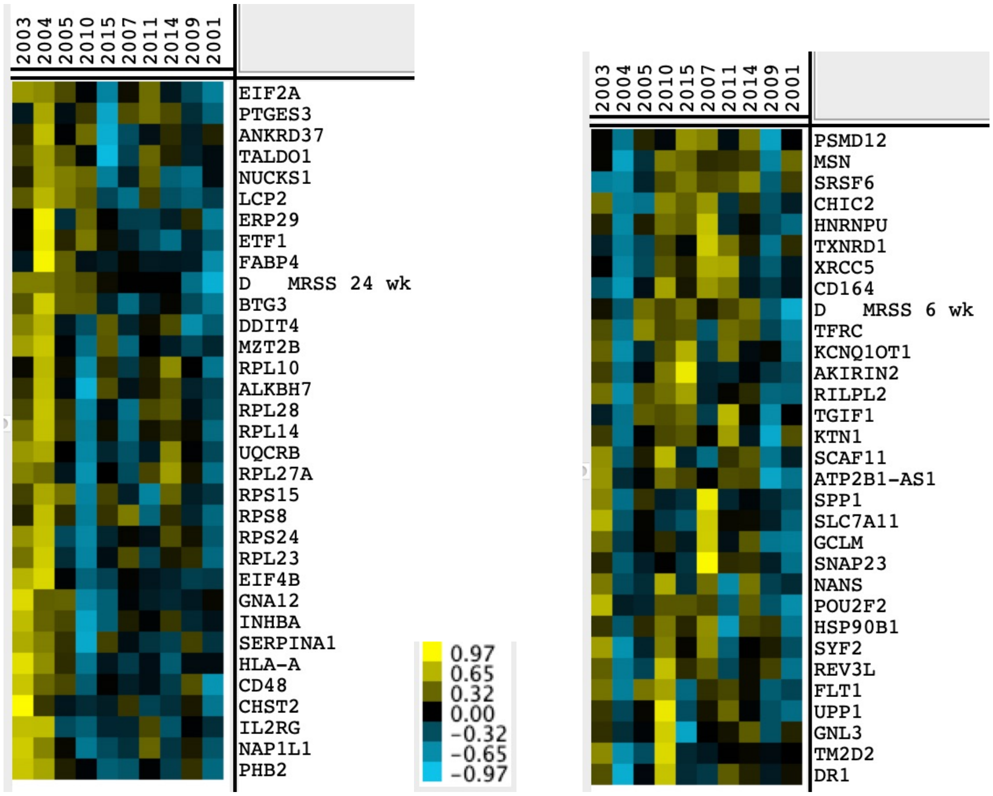

<u>Figure S11. Change in gene expression by cluster of dendritic cell genes, comparing week 6 to baseline.</u> Normalized change in MRSS is included in the clustering algorithm. Columns are arranged by change in MRSS at 24 weeks with increased (worsening) skin score (yellow) to the left and decreased (improved) skin score (blue) to the right. Genes are shown that cluster with change in MRSS at 24 weeks (D MRSS 24 weeks, left panel) or with change in MRSS at 6 weeks (D MRSS 6 weeks, right panel). Analysis of genes in clusters by Gene Otology did not indicate and statistically significant pathways.

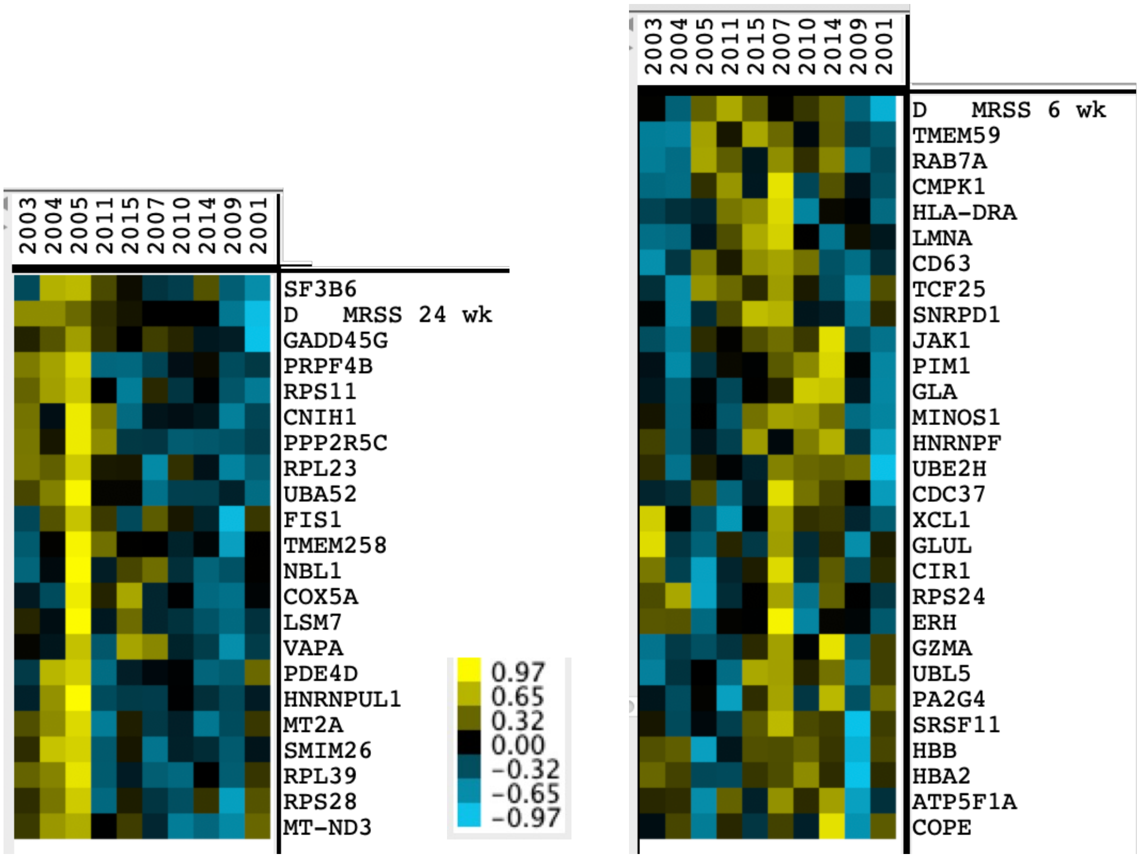

<u>Figure S12. Change in gene expression by cluster of T cell genes, comparing week 6 to baseline.</u> Normalized change in MRSS is included in the clustering algorithm. Columns are arranged by change in MRSS at 24 weeks with increased (worsening) skin score (yellow) to the left and decreased (improved) skin score (blue) to the right. Genes are shown that cluster with change in MRSS at 24 weeks (D MRSS 24 weeks, left panel) or with change in MRSS at 6 weeks (D MRSS 6 weeks, right panel). Analysis of genes in clusters by Gene Otology did not indicate and statistically significant pathways.

Supplementary Table 1: Baseline and Demographics Characteristics

	Double Blind Trial			Open Label Extension		
	**Overall (N=15)**	**Tofacitinib (N=10)**	**Placebo (N=5)**	**Overall (N=14)**	**Tofacitinib (N=10)**	**Placebo (N=4)**
Age, Years, mean (SD)	50.8 (14.0)	46.2 (13.9)	60.0 (9.7)	49.7 (13.9)	46.2 (13.9)	58.4 (10.5)
Female, N (%)	10.0 (66.7)	7.0 (70.0)	3.0 (60.0)	9 (64.3)	7.0 (70.0)	2.0 (50.0)
White, N (%)	14.0 (93.3)	10.0 (100.0)	4.0 (80.0)	13.0 (92.9)	10.0 (100.0)	3.0 (75.0)
Not Hispanic or Latino, N (%)	15.0 (100.0)	10.0 (100.0)	5.0 (100.0)	14.0 (100.0)	10.0 (100.0)	4.0 (100.0)
Disease Duration, Years*, mean (SD)	2.1 (1.2)	2.0 (1.3)	2.4 (1.2)	2.0 (1.2)	2.0 (1.3)	2.1 (1.1)
mRSS, mean (SD)	23.3 (8.4)	22.7 (9.3)	24.4 (6.9)	22.8 (8.5)	22.7 (9.3)	23.0 (7.1)
FVC% Predicted, mean (SD)	88.4 (16.6)	90.7 (16.5)	83.8 (17.5)	88.2 (17.2)	90.7 (16.5)	82.0 (19.6)
DLCO% Predicted, Uncorrected for Hgb, mean (SD)	85.6 (19.8)	82.4 (19.7)	92.0 (20.5)	86.2 (20.4)	82.4 (19.7)	95.8 (21.6)
Patient Global Assessment, mean (SD)[1]	4.3 (2.2)	3.8 (1.9)	5.4 (2.6)	4.3 (2.3)	3.8 (1.9)	5.5 (3.0)
HAQ-DI, mean (SD)[2]	1.0 (0.6)	1.1 (0.6)	0.8 (0.5)	0.9 (0.6)	1.1 (0.6)	0.6 (0.3)
Physician Global Assessment, mean (SD)[1]	5.3 (1.7)	5.6 (1.8)	4.8 (1.6)	5.3 (1.8)	5.6 (1.8)	4.5 (1.7)
Tendon Friction Rubs, N (%)	2.0 (13.3)	1.0 (10.0)	1.0 (20.0)	2.0 (14.3)	1.0 (10.0)	1.0 (25.0)
Large Joint Contractures, N (%)	5.0 (33.3)	5.0 (50.0)	0.0 (0.0)	5.0 (35.7)	5.0 (50.0)	0.0 (0.0)
Background Immunosuppressive, N (%)	13.0 (86.7)	9.0 (90.0)	4.0 (80.0)	12.0 (85.7)	9.0 (90.0)	3.0 (75.0)
Mycophenolate mofetil, N (%)	13.0 (86.7)	9.0 (90.0)	4.0 (80.0)	12.0 (85.7)	9.0 (90.0)	3.0 (75.0)
Mycophenolate mofetil in mg/day, mean (SD)	1500.2 (706.8)	1389.1 (781.3)	1750.0 (500.0)	1541.8 (721.3)	1389.1 (781.3)	2000.0 (0.0)
Methotrexate, mg/week,, N (%)	2.0 (13.3)	1.0 (10.0)	1.0 (20.0)	2.0 (14.2)	1.0 (10.0)	1.0 (25.0)
Methotrexate, mg/week, mean (SD)	25.0 (0.0)	25.0 (-)	25.0 (-)	25.0 (0.0)	25.0 (-)	25.0 (-)

Use of Prednisone, N (%)	4.0 (26.7)	2.0 (20.0)	2.0 (40.0)	3.0 (21.4)	2.0 (20.0)	1.0 (25.0)
Prednisone dose in mg/day, mean (SD)	8.8 (2.5)	7.5 (3.5)	10.0 (0.0)	8.3 (2.9)	7.5 (3.5)	10.0 (-)

*Disease onset was defined as first non-Raynaud's sign or symptoms; mRSS=modified Rodnan skin score; FVC=Forced vital capacity, DLCO =Diffusion capacity of carbon monoxide; [1] =theoretical range 0-10; [2]=theoretical range 0-3

Supplementary Table 2: Safety Outcomes during the double blind and open label extension in the RCT

	Double Blinded Trial		Open Label Extension	
	**Tofacitinib N=10**	**Placebo N=5**	**Tofacitinib → Tofacitinib N = 10**	**Placebo → Tofacitinib N= 4**
**Grade 3 or Higher AEs**	0	0	3	1
**Grade 2 of Higher AEs**[1]	13	10	18	4
**Treatment Emergent AEs**	20	14	21	7
Infections and Infestations	4	2	4	1
Gastrointestinal Disorders	4	1	3	0
General Disorders	1	4	0	0
Musculoskeletal and Connective Tissue Disorders	2	3	2	1
Skin and Subcutaneous Disorder	1	0	6	1
Investigations	2	1	3	2
Respiratory, Thoracic, and Mediastinal Disorders	2	1	1	0
Nervous System Disorders	2	0	2	0
Cardiac Disorders	1	1	0	1
Renal and Urinary Disorders	0	1	0	1
Ear and Labyrinth Disorders	1	0	0	0
**AESI**	5	0	6	1
Serious Infections	0	0	1	0
Infections Requiring Treatment	4	0	2	0
Otitis Externa	1	0	0	0
Urinary Tract Infection	2	0	0	0
Digital Ulcer Infection	1	0	1	0
CMV	0	0	1	0
Gastrointestinal Perforations	0	0	0	0
Herpes Zoster	0	0	1	0
Malignancies	0	0	0	0

Gastrointestinal Perforations	0	0	0	0
***Laboratories Abnormalities*[3]**	1	0	2	1
Hypercholesterolemia	0	0	1	0
Hypertriglyceridemia	0	0	1	0
HDL>50%[4]	1	0	0	1

AE= Adverse Event; AESI= Adverse Event of Special Interest; CMV= Cytomegalovirus; HDL= high-density lipoproteins
[1] Grade 2: Moderate; minimal, local or non-invasive intervention indicated; limiting age-appropriate instrumental ADL (activities of daily living)
[2] Two participants had UTI
[3] Additional lab values included per protocol as AEs of special interest of which did not occur during the study include: Hb $\leq$ 8 gm/dL, Hb drop by >2 gm/dL, AST/ALT >3 ULN, increase in serum creatinine >50%, Hy's law= ALT > 3x ULN + bilirubin > 2x ULN Lymphocyte count less than 500 cells/mm3, ANC less than 500 cells/mm3
[4] Reported as AE of special interest per protocol as tofacitinib can impact lipids but was not considered clinically significant.

Supplementary Table 3: Secondary Endpoints at Week 24 and Week 48 – Efficacy: Changes from Baseline

	Double Blind Trial (Week 24)				Open label Extension (Week 48)			
	Median (IQR)		Difference of median (95% CI) ¶	P-Value	Median (IQR)		Difference of median (95% CI) ¶	P-Value
	Tofacitinib (N = 10)	Placebo (N = 4)			Tofacitinib→ Tofacitinib (N = 8)	Placebo→ Tofacitinib (N = 3)		
**mRSS**	-5.5 (-6.0, -1.0)	-2.5 (-7.5, 2.5)	-3.0 (-12.0, 6.0)	0.47	-12.5 (-15.5, -5.5)	-9.0 (-11.0, -9.0)	-3.5 (-7.0, 5.0)	0.60
**Patient Global Assessment**	0.0 (-1.0, 1.0)	-1.5 (-5.0, -0.5)	1.5 (0.0, 8.0)	0.16	-0.5 (-1.5, 1.0)	0.0 (-8.0, 5.0)	-0.5 (-6.0, 8.0)	0.85
**Physician Global Assessment**	-1.5 (-3.0, -1.0)	0.5 (0.0, 1.0)	-2.0 (-3.0, 2.0)	0.03	-3.5 (-4.0, -1.0)	-1.0 (-2.0, 2.0)	-2.5 (-6.0, 1.0)	0.15
**HAQ-DI**	-0.1 (-0.3, 0.0)	0.13 (0, 0.1)	-0.2 (-0.4, 0.1)	0.30	-0.4 (-0.4, -0.1)	0.1 (-0.1, 0.3)	-0.5 (-0.6, 0.0)	0.07
**FVC % Predicted**	-0.5 (-2.0, 6.0)	5.0 (2.0, 9.5)	-5.5 (-14.0, 3.0)	0.14	-1.0 (-6.0, 1.5)	4.0 (-3.0, 15.0)	-5 (-17.0, 7.0)	0.92
**ACR CRISS**	0.3 (0.0, 1.0)	0.1 (0.0, 0.6)	0.2 (-0.5, 1.0)	0.84	1.0 (0.3, 1.0)	0.8 (0.5, 1.0)	0.2 (-0.5, 0.5)	0.92

*Negative is improvement for all outcome measures, except FVC% predicted
¶ 95% CI of difference of median was obtained via bootstrapping.

Supplementary Table 4: Top 50 genes/cluster

cluster	gene	avg_logFC	pct.1	pct.2	p_val	p_val_adj
0	KRT1	1.9721797	1	0.56	0	0
0	LYPD3	1.850546992	0.996	0.427	0	0
0	KRT10	1.729270499	1	0.847	0	0
0	SFN	1.690887964	1	0.67	0	0
0	SERPINB2	1.609799454	0.939	0.283	0	0
0	DMKN	1.478722092	1	0.59	0	0
0	AQP3	1.415986606	0.998	0.521	0	0
0	TACSTD2	1.350037576	0.996	0.516	0	0
0	DSP	1.308965821	0.983	0.463	0	0
0	KRTDAP	1.248193366	0.98	0.413	0	0
0	LGALS7B	1.210466661	0.991	0.507	0	0
0	PHLDA2	1.181818143	0.956	0.487	0	0
0	PERP	1.179131174	1	0.696	0	0
0	LY6D	1.173808315	0.997	0.471	0	0
0	S100A14	1.171144873	0.998	0.513	0	0
0	SBSN	1.171111544	0.941	0.257	0	0
0	SERPINB5	1.093049992	0.972	0.351	0	0
0	CLDN1	1.081912845	0.875	0.181	0	0
0	KLF5	1.080681255	0.985	0.456	0	0
0	DSC3	1.02554917	0.954	0.327	0	0
0	CLDN4	1.025491772	0.839	0.17	0	0
0	FGFBP1	0.997202261	0.727	0.157	0	0
0	TUBB2A	0.938320162	0.979	0.607	0	0
0	IRF6	0.935430441	0.898	0.2	0	0
0	TUBA4A	0.826132328	0.918	0.417	0	0
0	EMP2	0.809103678	0.936	0.543	0	0
0	HBEGF	0.788268529	0.713	0.219	0	0
0	EHF	0.777897794	0.786	0.146	0	0
0	CCL27	0.773184177	0.823	0.201	0	0
0	PKP1	0.771037033	0.869	0.209	0	0
0	SLC38A2	0.768709538	0.948	0.674	0	0
0	DSG1	0.765928829	0.832	0.175	0	0
0	KRT2	0.740697298	0.782	0.303	0	0
0	KLF4	0.735338597	0.979	0.728	0	0
0	EZR	0.729793725	0.96	0.513	0	0
0	LGALS7	0.726599185	0.603	0.247	0	0
0	BAZ1A	0.724494588	0.886	0.432	0	0
0	HCAR3	0.713847244	0.593	0.087	0	0
0	TUBA1C	0.713381445	0.931	0.564	0	0
0	DEFB1	0.70516673	0.801	0.166	0	0
0	EPHA2	0.703229294	0.771	0.18	0	0
0	ADRB2	0.691787991	0.766	0.219	0	0
0	KLK11	0.681565136	0.79	0.188	0	0
0	NXF1	0.675920772	0.8	0.266	0	0
0	SOX15	0.674582431	0.857	0.244	0	0
0	DYNLL1	0.669257776	0.997	0.946	0	0
0	ABHD5	0.669188715	0.814	0.287	0	0
0	CD55	0.664371336	0.852	0.461	0	0
0	DNAJA1	0.661836801	0.996	0.82	0	0
0	PLIN3	0.660561808	0.818	0.292	0	0
1	COL1A1	2.494484638	0.999	0.468	0	0
1	LUM	2.470142997	0.99	0.276	0	0
1	PI16	2.398787847	0.84	0.08	0	0
1	COL1A2	2.395930304	1	0.455	0	0
1	COL3A1	2.313007362	0.987	0.369	0	0

**1**	MMP2	2.295657587	0.997	0.222	0	0
**1**	COMP	2.278187714	0.585	0.062	0	0
**1**	FBLN1	2.275928895	0.979	0.281	0	0
**1**	ELN	2.270597391	0.931	0.13	0	0
**1**	DCN	2.241280591	0.999	0.542	0	0
**1**	SFRP2	2.23657602	0.919	0.131	0	0
**1**	CTHRC1	2.14006806	0.964	0.146	0	0
**1**	SPARC	2.094846179	0.997	0.58	0	0
**1**	WISP2	1.967734922	0.87	0.111	0	0
**1**	CTSK	1.966815892	0.973	0.179	0	0
**1**	CCDC80	1.89346588	0.988	0.314	0	0
**1**	MFAP5	1.893462828	0.869	0.087	0	0
**1**	FBLN2	1.785986336	0.971	0.188	0	0
**1**	FSTL1	1.784700341	0.982	0.232	0	0
**1**	DPT	1.771833536	0.916	0.139	0	0
**1**	CFD	1.750398537	0.918	0.463	0	0
**1**	POSTN	1.714477826	0.688	0.187	0	0
**1**	MFAP4	1.703140524	0.969	0.245	0	0
**1**	IGFBP4	1.669672589	0.98	0.461	0	0
**1**	COL6A2	1.616637099	1	0.483	0	0
**1**	BGN	1.603122288	0.807	0.315	0	0
**1**	CTGF	1.54839032	0.797	0.239	0	0
**1**	AEBP1	1.544983992	0.969	0.215	0	0
**1**	PLAC9	1.533499308	0.972	0.336	0	0
**1**	C1R	1.499934416	0.975	0.366	0	0
**1**	CXCL14	1.492440577	0.953	0.755	0	0
**1**	COL6A1	1.488339336	0.995	0.411	0	0
**1**	SERPINF1	1.480424092	0.964	0.361	0	0
**1**	FBN1	1.439424424	0.9	0.104	0	0
**1**	THY1	1.384947559	0.902	0.183	0	0
**1**	HTRA1	1.376671187	0.964	0.246	0	0
**1**	MXRA8	1.355976358	0.932	0.176	0	0
**1**	C1S	1.315712363	0.987	0.322	0	0
**1**	LOX	1.308321032	0.878	0.093	0	0
**1**	FN1	1.305568852	0.872	0.191	0	0
**1**	PCOLCE	1.300190598	0.947	0.294	0	0
**1**	S100A6	1.266964518	1	0.819	0	0
**1**	TIMP1	1.266713308	0.992	0.569	0	0
**1**	CD99	1.259055608	0.997	0.689	0	0
**1**	TIMP2	1.257112648	0.956	0.257	0	0
**1**	MGP	1.245660264	0.955	0.471	0	0
**1**	THBS2	1.244402591	0.865	0.131	0	0
**1**	LGALS1	1.223946633	1	0.658	0	0
**1**	C1QTNF3	1.223855103	0.659	0.021	0	0
**1**	COL6A3	1.200556785	0.904	0.18	0	0
**2**	CCL2	2.628897911	0.973	0.484	0	0
**2**	RGS16	2.330185935	0.955	0.285	0	0
**2**	C11orf96	2.219597803	0.926	0.161	0	0
**2**	IL6	2.103156323	0.734	0.108	0	0
**2**	GEM	1.901234639	0.864	0.178	0	0
**2**	MT1A	1.867012416	0.808	0.073	0	0
**2**	RGS5	1.764812914	0.582	0.069	0	0
**2**	CEBPD	1.707652687	0.986	0.813	0	0
**2**	HSPA6	1.672967849	0.388	0.124	0	0
**2**	CCL8	1.575239345	0.387	0.03	0	0
**2**	TAGLN	1.521202612	0.965	0.266	0	0
**2**	ID4	1.515167586	0.811	0.228	0	0
**2**	ADAMTS1	1.386601822	0.856	0.216	0	0
**2**	CRISPLD2	1.376030383	0.797	0.138	0	0
**2**	ADIRF	1.360034192	0.995	0.617	0	0
**2**	DDIT4	1.353870335	0.844	0.388	0	0
**2**	JUNB	1.347477541	0.999	0.96	0	0
**2**	ACTA2	1.304942552	0.854	0.156	0	0
**2**	SOCS3	1.285773186	0.952	0.581	0	0

**2**	CXCL2	1.277341507	0.523	0.193	0	0
**2**	GADD45B	1.273099563	0.985	0.705	0	0
**2**	TPM2	1.254411591	0.879	0.2	0	0
**2**	MYL9	1.235287862	0.957	0.438	0	0
**2**	ZFP36	1.214255037	0.994	0.893	0	0
**2**	GJA4	1.200763079	0.604	0.047	0	0
**2**	ZNF331	1.193953963	0.624	0.121	0	0
**2**	CDKN1A	1.152623858	0.968	0.738	0	0
**2**	IGFBP5	1.149773873	0.744	0.217	0	0
**2**	PPP1R15A	1.145511173	0.995	0.842	0	0
**2**	IER3	1.137740152	0.943	0.774	0	0
**2**	LBH	1.12917487	0.777	0.168	0	0
**2**	TINAGL1	1.128646308	0.857	0.124	0	0
**2**	RRAD	1.127415462	0.633	0.072	0	0
**2**	ATF3	1.108938187	0.911	0.542	0	0
**2**	NR4A1	1.092985563	0.893	0.474	0	0
**2**	IRF1	1.077124519	0.928	0.547	0	0
**2**	MT2A	1.061117261	0.949	0.797	0	0
**2**	CRYAB	1.031311901	0.714	0.435	0	0
**2**	CPE	1.026039324	0.902	0.226	0	0
**2**	ADAMTS4	1.00887127	0.605	0.087	0	0
**2**	MT1M	1.000852215	0.729	0.178	0	0
**2**	RERGL	0.992164878	0.448	0.033	0	0
**2**	NDUFA4L2	0.981254763	0.605	0.224	0	0
**2**	IGFBP7	0.97891793	1	0.632	0	0
**2**	MYC	0.956012572	0.927	0.694	0	0
**2**	ADRA2A	0.928439339	0.623	0.057	0	0
**2**	CALD1	0.912099848	0.964	0.563	0	0
**2**	PHLDA1	0.911699224	0.656	0.196	0	0
**2**	CSRNP1	0.886164389	0.748	0.27	0	0
**2**	CYCS	1.026872748	0.842	0.69	2.48E-289	5.90E-285
**3**	CXCR4	1.945957378	0.822	0.068	0	0
**3**	IL32	1.726271481	0.82	0.119	0	0
**3**	SRGN	1.660338349	0.914	0.162	0	0
**3**	CD52	1.574453432	0.783	0.039	0	0
**3**	CREM	1.567299558	0.781	0.189	0	0
**3**	LTB	1.468084389	0.628	0.018	0	0
**3**	DUSP2	1.464595834	0.785	0.165	0	0
**3**	KLRB1	1.319011537	0.319	0.009	0	0
**3**	CD69	1.293689362	0.575	0.021	0	0
**3**	SARAF	1.228559464	0.88	0.523	0	0
**3**	SAMSN1	1.199136927	0.639	0.035	0	0
**3**	TRAC	1.177242809	0.649	0.033	0	0
**3**	RGS1	1.161882269	0.443	0.038	0	0
**3**	ARHGDIB	1.137198353	0.771	0.248	0	0
**3**	BTG1	1.117889638	0.998	0.9	0	0
**3**	BIRC3	1.082859996	0.634	0.152	0	0
**3**	TRBC2	1.080243422	0.548	0.007	0	0
**3**	CCL5	1.076003937	0.332	0.017	0	0
**3**	RGCC	1.04842238	0.651	0.22	0	0
**3**	TRBC1	1.038520198	0.432	0.006	0	0
**3**	CD3D	1.022603216	0.614	0.003	0	0
**3**	CYTIP	1.002076549	0.573	0.038	0	0
**3**	CORO1A	0.984013445	0.629	0.046	0	0
**3**	DUSP4	0.971700568	0.43	0.072	0	0
**3**	IL7R	0.970639105	0.512	0.009	0	0
**3**	TMSB4X	0.962686217	1	0.993	0	0
**3**	ZFP36L2	0.962027585	0.965	0.84	0	0
**3**	PTPRC	0.961326496	0.639	0.035	0	0
**3**	CLEC2D	0.939780329	0.527	0.02	0	0
**3**	RHOH	0.921609435	0.548	0.014	0	0
**3**	LEPROTL1	0.910443436	0.58	0.119	0	0
**3**	LAPTM5	0.866320028	0.662	0.083	0	0
**3**	B2M	0.863313069	1	0.998	0	0

**3**	STK4	0.860986076	0.619	0.11	0	0
**3**	GPR183	0.844011369	0.544	0.068	0	0
**3**	RPS29	0.842797541	1	0.999	0	0
**3**	ACAP1	0.832132505	0.583	0.042	0	0
**3**	FXYD5	0.823399422	0.694	0.259	0	0
**3**	CD2	0.821229784	0.487	0.007	0	0
**3**	HCST	0.809206307	0.581	0.062	0	0
**3**	EZR	0.804303646	0.857	0.523	0	0
**3**	CD37	0.800053021	0.586	0.048	0	0
**3**	RPS27	0.789924433	1	1	0	0
**3**	NR4A2	0.766754962	0.617	0.251	0	0
**3**	CD3E	0.764850748	0.518	0.003	0	0
**3**	ZNF331	0.762300844	0.552	0.126	0	0
**3**	CD48	0.753935359	0.53	0.028	0	0
**3**	STK17B	0.751686456	0.538	0.122	0	0
**3**	ISG20	0.750058839	0.494	0.081	0	0
**3**	SPOCK2	0.748939863	0.468	0.006	0	0
**4**	KRT14	1.648690938	1	0.66	0	0
**4**	KRT15	1.591000869	0.928	0.153	0	0
**4**	DST	1.388283275	0.91	0.309	0	0
**4**	S100A2	1.328477235	0.981	0.361	0	0
**4**	AREG	1.213406429	0.672	0.195	0	0
**4**	KRT5	1.16855723	1	0.474	0	0
**4**	COL17A1	1.086465996	0.868	0.122	0	0
**4**	CCL27	0.821915638	0.655	0.217	0	0
**4**	SOX15	0.785246059	0.824	0.253	0	0
**4**	ERRFI1	0.78116934	0.711	0.267	0	0
**4**	SERPINB2	0.7580056	0.713	0.302	0	0
**4**	RPLP1	0.730798555	1	1	0	0
**4**	ADRB2	0.718197916	0.681	0.23	0	0
**4**	HOPX	0.710313226	0.871	0.37	0	0
**4**	SYT8	0.70154934	0.556	0.077	0	0
**4**	DNAJA1	0.697824609	0.96	0.824	0	0
**4**	KLF5	0.689747596	0.908	0.466	0	0
**4**	IL18	0.644031178	0.718	0.184	0	0
**4**	FGFBP1	0.628090996	0.562	0.172	0	0
**4**	HSPA2	0.627514215	0.748	0.357	0	0
**4**	ARL4A	0.603399912	0.695	0.279	0	0
**4**	CKS2	0.586938147	0.764	0.451	0	0
**4**	TGFBI	0.586006822	0.716	0.264	0	0
**4**	ATP1B3	0.580029377	0.945	0.632	0	0
**4**	PMAIP1	0.576823684	0.699	0.276	0	0
**4**	SFN	0.57304413	1	0.674	0	0
**4**	FBXW7	0.546919241	0.688	0.23	0	0
**4**	TOB1	0.545247017	0.765	0.429	0	0
**4**	ALDH3A1	0.54057311	0.563	0.107	0	0
**4**	AMD1	0.54042605	0.828	0.564	0	0
**4**	ZC3H12A	0.537845052	0.76	0.433	0	0
**4**	AQP3	0.534542332	0.958	0.529	0	0
**4**	ANKRD37	0.53357321	0.473	0.162	0	0
**4**	CXADR	0.530599713	0.708	0.232	0	0
**4**	TGIF1	0.530323256	0.72	0.366	0	0
**4**	DUSP14	0.517176098	0.586	0.185	0	0
**4**	MT1X	0.511160256	0.959	0.742	0	0
**4**	FAM213A	0.490734948	0.69	0.265	0	0
**4**	NPM1	0.488937694	0.996	0.952	0	0
**4**	POSTN	0.488057662	0.752	0.186	0	0
**4**	C19orf33	0.472433768	0.789	0.329	0	0
**4**	CBR1	0.47084275	0.719	0.348	0	0
**4**	TIPARP	0.466064569	0.61	0.225	0	0
**4**	TACSTD2	0.460989052	0.902	0.527	0	0
**4**	HSP90AA1	0.546022259	0.995	0.97	6.57E-295	1.56E-290
**4**	KLF4	0.473572069	0.92	0.734	1.80E-288	4.29E-284
**4**	H2AFZ	0.495993611	0.955	0.823	7.39E-286	1.76E-281

**4**	MYC	0.495912897	0.897	0.696	3.77E-259	8.96E-255
**4**	ARID5B	0.591231584	0.768	0.604	1.02E-204	2.43E-200
**4**	DDX5	0.482252127	0.967	0.934	1.89E-189	4.50E-185
**5**	KRT2	3.43048635	0.976	0.301	0	0
**5**	KRTDAP	2.704584157	0.999	0.419	0	0
**5**	SBSN	2.399388647	0.992	0.264	0	0
**5**	CALML5	2.368178091	0.845	0.148	0	0
**5**	KRT10	2.131580525	1	0.849	0	0
**5**	DMKN	2.028034623	1	0.596	0	0
**5**	SLURP1	1.972533363	0.473	0.022	0	0
**5**	SPRR1B	1.910948157	0.611	0.048	0	0
**5**	FABP5	1.897551024	0.982	0.517	0	0
**5**	LYPD3	1.863607981	0.989	0.435	0	0
**5**	CSTA	1.812916987	0.901	0.236	0	0
**5**	LGALS7B	1.579278324	0.996	0.513	0	0
**5**	KRT1	1.568828695	0.993	0.566	0	0
**5**	SFN	1.513497596	1	0.674	0	0
**5**	PERP	1.508377635	1	0.7	0	0
**5**	TMEM45A	1.488439962	0.849	0.275	0	0
**5**	SPINK5	1.436375023	0.675	0.127	0	0
**5**	TACSTD2	1.396858933	0.98	0.523	0	0
**5**	DSP	1.386318542	0.939	0.472	0	0
**5**	CLDN4	1.368638517	0.791	0.181	0	0
**5**	CALML3	1.312252444	0.812	0.155	0	0
**5**	PKP1	1.273528712	0.837	0.219	0	0
**5**	LY6D	1.25628503	0.984	0.479	0	0
**5**	SERPINB2	1.227742289	0.685	0.303	0	0
**5**	DSG1	1.213189831	0.804	0.185	0	0
**5**	PHLDA2	1.195692715	0.918	0.495	0	0
**5**	CTNNBIP1	1.190102954	0.866	0.254	0	0
**5**	IVL	1.152325785	0.529	0.031	0	0
**5**	CLDN1	1.147142142	0.767	0.195	0	0
**5**	DSC1	1.122811782	0.723	0.093	0	0
**5**	LGALS7	1.112582671	0.61	0.251	0	0
**5**	KLK11	1.085109551	0.837	0.194	0	0
**5**	TUBA4A	1.067574938	0.906	0.425	0	0
**5**	SERPINB5	1.063029706	0.909	0.362	0	0
**5**	JUP	1.061303254	0.85	0.316	0	0
**5**	SLPI	1.056407018	0.731	0.161	0	0
**5**	SERPINA12	1.023664391	0.405	0.032	0	0
**5**	SCEL	1.006779178	0.593	0.055	0	0
**5**	S100A14	1.003864909	0.995	0.519	0	0
**5**	SULT2B1	0.971492301	0.631	0.061	0	0
**5**	GLTP	0.969981176	0.795	0.235	0	0
**5**	DAPL1	0.969707148	0.83	0.249	0	0
**5**	EMP2	0.942197265	0.876	0.551	0	0
**5**	CLIC3	0.939146088	0.467	0.054	0	0
**5**	SDC1	0.935417808	0.756	0.211	0	0
**5**	TRIM29	0.92544599	0.797	0.259	0	0
**5**	AQP3	0.898462333	0.964	0.528	0	0
**5**	LGALSL	0.883523194	0.651	0.172	0	0
**5**	DEFB1	0.883003128	0.788	0.175	0	0
**5**	RAB25	0.881209309	0.771	0.17	0	0
**6**	APOD	2.614537313	0.991	0.344	0	0
**6**	GSN	2.539012038	1	0.732	0	0
**6**	CFD	2.511711285	0.993	0.463	0	0
**6**	MGP	2.397535339	0.998	0.472	0	0
**6**	PLA2G2A	2.308252841	0.684	0.039	0	0
**6**	CFH	1.813943248	0.968	0.19	0	0
**6**	IGFBP6	1.712808077	0.869	0.132	0	0
**6**	C3	1.671982173	0.904	0.086	0	0
**6**	CCDC80	1.564995146	0.993	0.318	0	0
**6**	CXCL12	1.544201794	0.982	0.296	0	0
**6**	DCN	1.532661736	0.999	0.545	0	0

**6**	C1S	1.431402467	0.991	0.326	0	0
**6**	SERPING1	1.430603559	0.991	0.405	0	0
**6**	SERPINF1	1.42298889	0.991	0.363	0	0
**6**	C1R	1.403035056	0.989	0.369	0	0
**6**	CLU	1.382381505	0.794	0.338	0	0
**6**	GPC3	1.370354996	0.821	0.066	0	0
**6**	EFEMP1	1.344282586	0.927	0.18	0	0
**6**	ITM2A	1.337364332	0.899	0.208	0	0
**6**	CXCL14	1.33672075	0.973	0.755	0	0
**6**	ADH1B	1.307191101	0.823	0.091	0	0
**6**	GPX3	1.295192276	0.747	0.166	0	0
**6**	MYOC	1.259592778	0.504	0.011	0	0
**6**	FBLN1	1.241422468	0.972	0.286	0	0
**6**	C7	1.199410325	0.583	0.037	0	0
**6**	THY1	1.15230411	0.842	0.191	0	0
**6**	SOD3	1.136079722	0.982	0.375	0	0
**6**	LUM	1.129435238	0.968	0.282	0	0
**6**	SRPX	1.11429259	0.832	0.151	0	0
**6**	TIMP1	1.086451851	0.952	0.574	0	0
**6**	APOE	1.085915822	0.888	0.654	0	0
**6**	WISP2	1.057708904	0.776	0.12	0	0
**6**	MFAP4	1.043067205	0.919	0.252	0	0
**6**	S100A13	1.042319532	0.958	0.404	0	0
**6**	NTRK2	1.037133333	0.825	0.174	0	0
**6**	PLAC9	1.030817516	0.96	0.34	0	0
**6**	FBLN2	1.001417339	0.924	0.196	0	0
**6**	COL6A2	0.993982017	0.998	0.486	0	0
**6**	PCOLCE	0.961050395	0.95	0.298	0	0
**6**	RARRES2	0.960080998	0.85	0.171	0	0
**6**	IGFBP3	0.947312877	0.623	0.098	0	0
**6**	CYGB	0.94550351	0.765	0.118	0	0
**6**	LGALS3BP	0.943755045	0.838	0.192	0	0
**6**	IGFBP4	0.933009435	0.947	0.466	0	0
**6**	MFAP5	0.928073927	0.705	0.099	0	0
**6**	LSP1	0.926165522	0.87	0.272	0	0
**6**	MGST1	0.920467035	0.739	0.109	0	0
**6**	SPON2	0.917740924	0.674	0.15	0	0
**6**	VIM	0.907827263	1	0.701	0	0
**6**	SELENOP	0.888907996	0.963	0.622	0	0
**7**	CCL19	2.507691313	0.567	0.034	0	0
**7**	CXCL12	2.334091646	0.993	0.296	0	0
**7**	PTGDS	2.232090718	0.937	0.24	0	0
**7**	APOE	2.048365704	0.928	0.652	0	0
**7**	C1S	1.607610573	0.985	0.326	0	0
**7**	CXCL14	1.579777225	0.984	0.755	0	0
**7**	CFH	1.495749474	0.953	0.191	0	0
**7**	C3	1.489182989	0.783	0.092	0	0
**7**	CCDC80	1.470283806	0.983	0.319	0	0
**7**	CFD	1.455575829	0.989	0.463	0	0
**7**	C1R	1.432962358	0.981	0.37	0	0
**7**	SERPING1	1.384295289	0.985	0.405	0	0
**7**	APOD	1.369861474	0.982	0.344	0	0
**7**	DCN	1.363335456	1	0.545	0	0
**7**	IGFBP7	1.337845763	0.999	0.634	0	0
**7**	SERPINF1	1.308734084	0.979	0.364	0	0
**7**	SOD3	1.287018272	0.97	0.376	0	0
**7**	MGP	1.244209756	0.961	0.474	0	0
**7**	PTN	1.17448257	0.759	0.125	0	0
**7**	FBLN1	1.17190776	0.917	0.288	0	0
**7**	RARRES2	1.111630039	0.848	0.171	0	0
**7**	GSN	1.084768677	0.993	0.732	0	0
**7**	COL6A2	1.081735947	0.991	0.487	0	0
**7**	THY1	1.052103328	0.836	0.191	0	0
**7**	TIMP1	1.048359734	0.979	0.573	0	0

**7**	CST3	1.034273139	1	0.789	0	0
**7**	NNMT	1.024829112	0.871	0.262	0	0
**7**	DPT	1.017026355	0.84	0.148	0	0
**7**	SFRP2	1.012795398	0.774	0.143	0	0
**7**	VCAN	1.01036942	0.807	0.135	0	0
**7**	S100A13	1.000288656	0.931	0.405	0	0
**7**	PCOLCE	0.997206945	0.944	0.298	0	0
**7**	C7	0.97438554	0.403	0.045	0	0
**7**	CLU	0.937947947	0.741	0.341	0	0
**7**	ADH1B	0.932767231	0.671	0.098	0	0
**7**	EFEMP1	0.927545422	0.799	0.186	0	0
**7**	TMEM176B	0.925167124	0.792	0.131	0	0
**7**	CTSK	0.897020961	0.91	0.188	0	0
**7**	SELENOM	0.896786523	0.957	0.469	0	0
**7**	IL11RA	0.894212166	0.756	0.132	0	0
**7**	CYGB	0.886259352	0.654	0.123	0	0
**7**	MFAP4	0.884217527	0.838	0.256	0	0
**7**	PLAC9	0.877946022	0.935	0.342	0	0
**7**	PLPP3	0.860803234	0.812	0.227	0	0
**7**	S100A6	0.85745797	1	0.82	0	0
**7**	VIM	0.844200461	1	0.701	0	0
**7**	FBLN2	0.823003212	0.864	0.199	0	0
**7**	IFITM3	0.822635588	0.998	0.752	0	0
**7**	MEG3	0.808328762	0.716	0.166	0	0
**7**	COL6A1	0.802259016	0.964	0.416	0	0
**8**	KRT14	2.375096239	1	0.661	0	0
**8**	KRT5	2.040354148	1	0.476	0	0
**8**	S100A2	1.654312808	0.989	0.363	0	0
**8**	COL17A1	1.458361408	0.919	0.122	0	0
**8**	DST	1.347181476	0.908	0.311	0	0
**8**	HOPX	0.965809482	0.94	0.368	0	0
**8**	AQP3	0.938553774	0.99	0.528	0	0
**8**	SYT8	0.853538014	0.644	0.075	0	0
**8**	KRT15	0.840467058	0.762	0.163	0	0
**8**	SOX15	0.800020666	0.834	0.254	0	0
**8**	FAM213A	0.780140503	0.822	0.261	0	0
**8**	MT1X	0.766107775	0.944	0.744	0	0
**8**	AREG	0.76529134	0.556	0.201	0	0
**8**	KLF5	0.757706213	0.919	0.467	0	0
**8**	SERPINB2	0.726692805	0.668	0.305	0	0
**8**	ERRFI1	0.715454694	0.669	0.271	0	0
**8**	PERP	0.696101194	0.999	0.701	0	0
**8**	CCL27	0.695638618	0.612	0.221	0	0
**8**	TGFBI	0.688142229	0.749	0.264	0	0
**8**	ATP1B3	0.684333545	0.959	0.633	0	0
**8**	IMPA2	0.668638142	0.786	0.232	0	0
**8**	FBXW7	0.666910118	0.743	0.229	0	0
**8**	SPINT2	0.658155373	0.898	0.377	0	0
**8**	SFN	0.657792876	1	0.675	0	0
**8**	IL18	0.630106234	0.696	0.186	0	0
**8**	C19orf33	0.627270562	0.833	0.328	0	0
**8**	PDLIM1	0.615374936	0.864	0.459	0	0
**8**	CXADR	0.606417677	0.749	0.232	0	0
**8**	FGFBP1	0.605953853	0.527	0.175	0	0
**8**	ADRB2	0.60087999	0.647	0.232	0	0
**8**	PKM	0.598072332	0.969	0.814	0	0
**8**	CBR1	0.588971488	0.775	0.347	0	0
**8**	TOB1	0.583794437	0.774	0.43	0	0
**8**	FXYD3	0.577326421	0.952	0.47	0	0
**8**	LGALS7	0.574149569	0.748	0.246	0	0
**8**	ALDH3A1	0.572260663	0.533	0.109	0	0
**8**	HSPA2	0.571973537	0.765	0.358	0	0
**8**	NDFIP2	0.535845035	0.723	0.235	0	0
**8**	FGFR3	0.531755184	0.64	0.153	0	0

**8**	ARL4A	0.531378004	0.667	0.281	0	0
**8**	CXCL14	0.528882483	0.994	0.755	0	0
**8**	PMAIP1	0.526170325	0.687	0.278	0	0
**8**	S100A14	0.524080391	0.948	0.523	0	0
**8**	DEGS1	0.522327871	0.864	0.496	0	0
**8**	SERPINB5	0.51985538	0.842	0.367	0	0
**8**	DUSP14	0.518619233	0.586	0.186	0	0
**8**	DNAJA1	0.631091106	0.919	0.826	9.88E-275	2.35E-270
**8**	HSP90AA1	0.54331514	0.991	0.97	5.92E-248	1.41E-243
**8**	MT1E	0.531942493	0.736	0.458	9.82E-236	2.34E-231
**8**	CKS2	0.526633232	0.695	0.455	1.35E-209	3.20E-205
**9**	PTGDS	2.629427691	0.974	0.239	0	0
**9**	SFRP2	2.555105522	0.965	0.136	0	0
**9**	COL1A1	2.48125612	1	0.472	0	0
**9**	COL3A1	2.329857121	0.996	0.373	0	0
**9**	COL1A2	2.311622744	1	0.46	0	0
**9**	COMP	2.224628281	0.709	0.061	0	0
**9**	COL6A1	2.150439892	1	0.415	0	0
**9**	COL6A2	1.989768633	1	0.487	0	0
**9**	SPARC	1.828772992	0.998	0.584	0	0
**9**	MMP2	1.770070909	0.974	0.23	0	0
**9**	DCN	1.659366902	0.991	0.546	0	0
**9**	CTSK	1.553272495	0.934	0.188	0	0
**9**	IGFBP4	1.540056401	0.958	0.466	0	0
**9**	APCDD1	1.42788231	0.82	0.169	0	0
**9**	LGALS1	1.395256356	1	0.661	0	0
**9**	HTRA1	1.389253643	0.945	0.253	0	0
**9**	PCOLCE	1.388159875	0.961	0.299	0	0
**9**	ELN	1.370653924	0.841	0.14	0	0
**9**	CTHRC1	1.35371438	0.812	0.159	0	0
**9**	TWIST2	1.340799482	0.902	0.125	0	0
**9**	C1R	1.339071515	0.968	0.371	0	0
**9**	FBLN1	1.316650221	0.87	0.291	0	0
**9**	COL6A3	1.289819184	0.915	0.186	0	0
**9**	NBL1	1.275428299	0.899	0.529	0	0
**9**	SERPINF1	1.248713716	0.962	0.366	0	0
**9**	C1S	1.239758414	0.966	0.328	0	0
**9**	WISP2	1.232369238	0.666	0.126	0	0
**9**	POSTN	1.22728652	0.57	0.196	0	0
**9**	LUM	1.210869944	0.891	0.286	0	0
**9**	COL5A1	1.202207001	0.86	0.108	0	0
**9**	MFAP4	1.194144903	0.915	0.254	0	0
**9**	AEBP1	1.191232194	0.919	0.224	0	0
**9**	FBLN2	1.175450645	0.92	0.197	0	0
**9**	S100A6	1.167292614	1	0.82	0	0
**9**	MEG3	1.165047457	0.905	0.158	0	0
**9**	FSTL1	1.164323538	0.906	0.241	0	0
**9**	TIMP2	1.154324068	0.924	0.264	0	0
**9**	PTN	1.152287521	0.743	0.127	0	0
**9**	THBS2	1.146794273	0.832	0.139	0	0
**9**	STC2	1.131037201	0.568	0.037	0	0
**9**	MXRA8	1.123466812	0.877	0.185	0	0
**9**	PLPP3	1.112605914	0.846	0.226	0	0
**9**	CLEC11A	1.099573289	0.834	0.163	0	0
**9**	MMP11	1.085912139	0.416	0.033	0	0
**9**	CXCL14	1.041623459	0.969	0.756	0	0
**9**	SOD3	1.034971903	0.943	0.378	0	0
**9**	PPIC	1.030697558	0.864	0.234	0	0
**9**	CFD	1.028088832	0.896	0.468	0	0
**9**	TIMP1	1.025606524	0.992	0.573	0	0
**9**	THY1	1.020223032	0.842	0.192	0	0
**10**	S100A14	1.066249857	0.994	0.523	0	0
**10**	DMKN	1.060263314	0.996	0.599	0	0
**10**	LY6D	1.048729284	0.991	0.483	0	0

**10**	MT1X	0.988720023	0.986	0.743	0	0
**10**	SFN	0.937523076	1	0.677	0	0
**10**	KRT5	0.913997598	0.89	0.483	0	0
**10**	KRT1	0.908361771	0.987	0.57	0	0
**10**	LGALS7B	0.86883037	0.976	0.518	0	0
**10**	KRT10	0.818181832	0.996	0.85	0	0
**10**	DSP	0.814236169	0.971	0.474	0	0
**10**	PERP	0.806948795	1	0.702	0	0
**10**	AQP3	0.71593941	0.982	0.531	0	0
**10**	DSC3	0.692882839	0.884	0.343	0	0
**10**	RPLP1	0.673184117	1	1	0	0
**10**	LGALS7	0.654505775	0.608	0.254	0	0
**10**	TACSTD2	0.641848983	0.941	0.528	0	0
**10**	KLF5	0.640710829	0.93	0.469	0	0
**10**	LYPD3	0.624847274	0.913	0.443	0	0
**10**	SOX15	0.599384526	0.765	0.26	0	0
**10**	RPS19	0.573820201	1	1	0	0
**10**	RPLP0	0.572627518	1	0.999	0	0
**10**	RPS18	0.572279228	1	1	0	0
**10**	FXYD3	0.567855769	0.943	0.473	0	0
**10**	SERPINB2	0.562373134	0.716	0.305	0	0
**10**	RHOV	0.554689266	0.678	0.178	0	0
**10**	RPS2	0.539921847	1	1	0	0
**10**	SERPINB5	0.52870134	0.838	0.369	0	0
**10**	EEF1B2	0.520377455	0.992	0.957	0	0
**10**	RPS8	0.516306785	1	1	0	0
**10**	RPS7	0.506782289	1	0.999	0	0
**10**	CCL27	0.504616366	0.656	0.221	0	0
**10**	HMGA1	0.502399775	0.713	0.291	0	0
**10**	RPL18	0.497467364	1	0.999	0	0
**10**	IRF6	0.491613218	0.665	0.224	0	0
**10**	TRIM29	0.489777312	0.767	0.264	0	0
**10**	PKP1	0.488449346	0.75	0.227	0	0
**10**	MT1G	0.483408695	0.443	0.09	0	0
**10**	RPL7A	0.475407704	1	0.999	0	0
**10**	RPS12	0.472965466	1	1	0	0
**10**	RPS3A	0.467355374	1	0.999	0	0
**10**	RPS6	0.464357725	1	1	0	0
**10**	TENT5B	0.464321841	0.618	0.22	0	0
**10**	DEFB1	0.461888849	0.665	0.184	0	0
**10**	FGFBP1	0.460817878	0.515	0.178	0	0
**10**	RPL18A	0.460618958	1	1	0	0
**10**	RPL10A	0.459807332	1	0.999	0	0
**10**	RPS10	0.457020158	1	0.994	0	0
**10**	PHLDA2	0.607423604	0.825	0.501	1.68E-302	4.00E-298
**10**	KRT14	0.619586842	0.884	0.668	1.65E-289	3.94E-285
**10**	MYC	0.479811543	0.904	0.697	7.37E-215	1.75E-210
**11**	KRT1	2.029778588	0.999	0.571	0	0
**11**	KRT10	1.804417889	1	0.85	0	0
**11**	DMKN	1.663715507	1	0.6	0	0
**11**	LY6D	1.339929858	0.997	0.484	0	0
**11**	KRTDAP	1.328774567	0.976	0.427	0	0
**11**	LGALS7B	1.324971796	0.995	0.519	0	0
**11**	PERP	1.200354399	0.999	0.703	0	0
**11**	LGALS7	1.194203878	0.776	0.25	0	0
**11**	DSP	1.060472113	0.963	0.476	0	0
**11**	MT1X	0.929056627	0.982	0.744	0	0
**11**	DEGS1	0.898081254	0.947	0.496	0	0
**11**	PKP1	0.894292202	0.852	0.226	0	0
**11**	DEFB1	0.838437711	0.827	0.18	0	0
**11**	JUP	0.811171627	0.896	0.321	0	0
**11**	FXYD3	0.810294598	0.962	0.474	0	0
**11**	DSC3	0.807109193	0.91	0.344	0	0
**11**	S100A14	0.795939223	0.991	0.525	0	0

**11**	CCL27	0.761132674	0.807	0.217	0	0
**11**	DSG1	0.735209206	0.814	0.192	0	0
**11**	SFN	0.730300749	0.999	0.678	0	0
**11**	RHOV	0.728413346	0.743	0.177	0	0
**11**	AQP3	0.680475411	0.981	0.533	0	0
**11**	NSG1	0.67564105	0.775	0.189	0	0
**11**	TRIM29	0.667559919	0.813	0.264	0	0
**11**	CTNNBIP1	0.647208236	0.8	0.263	0	0
**11**	KLK11	0.644563686	0.766	0.203	0	0
**11**	RPS18	0.632972912	1	1	0	0
**11**	CCND1	0.62938388	0.797	0.407	0	0
**11**	CSTA	0.61202543	0.807	0.247	0	0
**11**	GJA1	0.611790353	0.793	0.352	0	0
**11**	KCNK7	0.604066033	0.675	0.121	0	0
**11**	SBSN	0.603247573	0.882	0.276	0	0
**11**	S100A16	0.591935888	0.922	0.591	0	0
**11**	KRT2	0.585579314	0.757	0.316	0	0
**11**	RPLP1	0.580549212	1	1	0	0
**11**	ALDH2	0.579592969	0.797	0.413	0	0
**11**	CHP2	0.575583264	0.599	0.077	0	0
**11**	C19orf33	0.568016069	0.781	0.334	0	0
**11**	SDC1	0.567212312	0.723	0.219	0	0
**11**	GATA3	0.560667701	0.644	0.156	0	0
**11**	DAPL1	0.553448647	0.768	0.258	0	0
**11**	PKP3	0.55118319	0.732	0.22	0	0
**11**	RAB25	0.550940989	0.686	0.18	0	0
**11**	DBI	0.550277307	0.96	0.747	0	0
**11**	RPS19	0.54338758	1	1	0	0
**11**	NUPR1	0.542437505	0.937	0.677	0	0
**11**	OVOL1	0.514405259	0.558	0.177	0	0
**11**	GLTP	0.513449961	0.706	0.245	0	0
**11**	GATM	0.513269284	0.591	0.146	0	0
**11**	HES1	0.578920734	0.809	0.594	1.48E-167	3.52E-163
**12**	SELE	2.812319037	0.881	0.023	0	0
**12**	ACKR1	2.695230967	0.952	0.046	0	0
**12**	TM4SF1	2.667600623	0.999	0.274	0	0
**12**	IL6	2.417747032	0.871	0.114	0	0
**12**	AQP1	1.831170847	0.987	0.178	0	0
**12**	IFI27	1.768969797	0.999	0.461	0	0
**12**	PLVAP	1.748141838	0.971	0.056	0	0
**12**	SPARCL1	1.620922136	0.995	0.331	0	0
**12**	C2CD4B	1.612474323	0.799	0.027	0	0
**12**	G0S2	1.527314278	0.545	0.079	0	0
**12**	CD74	1.519176373	0.995	0.354	0	0
**12**	PECAM1	1.446149513	0.946	0.072	0	0
**12**	ICAM1	1.386324292	0.916	0.181	0	0
**12**	GNG11	1.360813885	0.975	0.246	0	0
**12**	PCAT19	1.351234555	0.923	0.055	0	0
**12**	NPDC1	1.341677909	0.93	0.166	0	0
**12**	RCAN1	1.323538247	0.83	0.119	0	0
**12**	SOCS3	1.286422556	0.922	0.589	0	0
**12**	STC1	1.27971246	0.487	0.016	0	0
**12**	CLDN5	1.27901333	0.83	0.067	0	0
**12**	CSF3	1.275658199	0.6	0.01	0	0
**12**	RAMP2	1.2707616	0.922	0.12	0	0
**12**	LMCD1	1.269314431	0.896	0.124	0	0
**12**	VWF	1.249534866	0.877	0.057	0	0
**12**	RAMP3	1.2441465	0.862	0.055	0	0
**12**	TMEM70	1.238345912	0.825	0.176	0	0
**12**	TSC22D1	1.235999099	0.882	0.519	0	0
**12**	HLA-DRB1	1.215394482	0.982	0.182	0	0
**12**	HLA-E	1.210661379	0.999	0.88	0	0
**12**	ECSCR	1.196672607	0.895	0.059	0	0
**12**	ADGRL4	1.161329784	0.896	0.05	0	0

**12**	ADAMTS9	1.149783406	0.757	0.058	0	0
**12**	IFIT1	1.149399189	0.572	0.06	0	0
**12**	CCL14	1.131431489	0.791	0.033	0	0
**12**	BCAM	1.128591951	0.951	0.189	0	0
**12**	A2M	1.119518108	0.91	0.176	0	0
**12**	NFKBIA	1.117502763	0.998	0.889	0	0
**12**	RND1	1.116409627	0.77	0.057	0	0
**12**	PDLIM1	1.115542841	0.955	0.46	0	0
**12**	ENG	1.112888142	0.872	0.133	0	0
**12**	CAV1	1.111128591	0.981	0.649	0	0
**12**	SDCBP	1.103003995	0.976	0.536	0	0
**12**	EMCN	1.089663483	0.877	0.081	0	0
**12**	CLU	1.083455382	0.873	0.341	0	0
**12**	CTGF	1.067192071	0.787	0.25	0	0
**12**	SOX17	1.066554659	0.608	0.029	0	0
**12**	ADAMTS1	1.050866311	0.804	0.229	0	0
**12**	CNKSR3	1.037758288	0.74	0.059	0	0
**12**	SPRY1	1.028365933	0.765	0.226	0	0
**12**	TSPAN7	1.020908686	0.825	0.052	0	0
**13**	ACTA2	2.677973679	0.999	0.165	0	0
**13**	TAGLN	2.538114748	1	0.278	0	0
**13**	MYL9	2.302821611	1	0.446	0	0
**13**	ADIRF	2.111703558	1	0.624	0	0
**13**	TPM2	2.105591867	0.994	0.209	0	0
**13**	RERGL	1.931366368	0.887	0.028	0	0
**13**	DSTN	1.767856407	0.999	0.916	0	0
**13**	PLN	1.766352482	0.878	0.038	0	0
**13**	MYH11	1.711271485	0.926	0.06	0	0
**13**	SNCG	1.494481991	0.836	0.122	0	0
**13**	BCAM	1.445275244	0.935	0.191	0	0
**13**	PPP1R14A	1.378551821	0.827	0.092	0	0
**13**	SPARCL1	1.372888246	0.971	0.333	0	0
**13**	TPM1	1.348917088	0.923	0.34	0	0
**13**	SORBS2	1.344946262	0.823	0.069	0	0
**13**	GADD45B	1.335851144	0.934	0.712	0	0
**13**	CSRP1	1.286998046	0.85	0.246	0	0
**13**	CALD1	1.246896193	0.984	0.57	0	0
**13**	LBH	1.241922578	0.865	0.177	0	0
**13**	MFGE8	1.24041151	0.93	0.351	0	0
**13**	RGS16	1.23993407	0.696	0.306	0	0
**13**	TINAGL1	1.228376884	0.897	0.137	0	0
**13**	IGFBP7	1.228296227	1	0.639	0	0
**13**	CRIP1	1.171769931	0.948	0.577	0	0
**13**	IGFBP5	1.146784596	0.869	0.223	0	0
**13**	NET1	1.136210466	0.78	0.157	0	0
**13**	C11orf96	1.097472934	0.828	0.178	0	0
**13**	CNN1	1.090852443	0.71	0.046	0	0
**13**	S100A4	1.079755405	0.993	0.668	0	0
**13**	FLNA	1.058021775	0.869	0.35	0	0
**13**	SOD3	1.045863808	0.957	0.384	0	0
**13**	CAVIN3	1.017434031	0.886	0.334	0	0
**13**	MCAM	1.001000036	0.773	0.106	0	0
**13**	ACTB	0.990930998	1	0.995	0	0
**13**	SELENOM	0.985484634	0.932	0.476	0	0
**13**	CCDC3	0.980460796	0.795	0.218	0	0
**13**	NDUFA4	0.960174897	0.987	0.874	0	0
**13**	CPE	0.939156551	0.895	0.239	0	0
**13**	CSRP2	0.91080527	0.73	0.203	0	0
**13**	CD151	0.904155931	0.846	0.43	0	0
**13**	SH3BGRL	0.902017669	0.893	0.494	0	0
**13**	LMOD1	0.893156336	0.689	0.065	0	0
**13**	MYLK	0.858403181	0.806	0.13	0	0
**13**	TSC22D1	0.856114774	0.896	0.519	0	0
**13**	CLU	0.853865301	0.838	0.343	0	0

**13**	RRAD	0.852377648	0.604	0.084	0	0
**13**	VIM	0.837564406	1	0.705	0	0
**13**	JUN	1.065365015	0.955	0.858	4.29E-269	1.02E-264
**13**	ATF3	0.990393482	0.741	0.554	1.66E-197	3.94E-193
**13**	JUNB	0.923130375	0.981	0.961	8.69E-192	2.07E-187
**14**	CCL3	3.057109268	0.7	0.026	0	0
**14**	CXCL8	2.776400337	0.789	0.087	0	0
**14**	RNASE1	2.731065487	0.852	0.085	0	0
**14**	C1QA	2.517137573	0.902	0.016	0	0
**14**	CXCL3	2.493605157	0.768	0.12	0	0
**14**	CCL4	2.434860484	0.603	0.027	0	0
**14**	FTL	2.350482026	1	0.97	0	0
**14**	CXCL2	2.162086665	0.815	0.191	0	0
**14**	CCL3L1	2.124775588	0.497	0.014	0	0
**14**	TYROBP	2.081390253	0.921	0.052	0	0
**14**	C1QB	1.971016476	0.794	0.01	0	0
**14**	HLA-DRA	1.919475946	0.954	0.221	0	0
**14**	IL1B	1.873075916	0.523	0.031	0	0
**14**	C1QC	1.852305037	0.8	0.01	0	0
**14**	GPR183	1.815653312	0.725	0.071	0	0
**14**	AIF1	1.774469156	0.858	0.036	0	0
**14**	SELENOP	1.743480639	0.951	0.627	0	0
**14**	CCL4L2	1.712372876	0.381	0.015	0	0
**14**	HMOX1	1.701518871	0.6	0.052	0	0
**14**	PLAUR	1.652951738	0.745	0.19	0	0
**14**	INSIG1	1.625769762	0.741	0.216	0	0
**14**	CXCL1	1.561510962	0.396	0.064	0	0
**14**	CCL13	1.520455703	0.418	0.012	0	0
**14**	MMP9	1.518062975	0.541	0.022	0	0
**14**	F13A1	1.512589758	0.675	0.042	0	0
**14**	SRGN	1.5080004	0.849	0.176	0	0
**14**	FCER1G	1.499377262	0.797	0.048	0	0
**14**	CD74	1.493648221	0.986	0.356	0	0
**14**	CTSB	1.487613605	0.784	0.306	0	0
**14**	CD68	1.487457702	0.727	0.091	0	0
**14**	HLA-DRB1	1.477415389	0.843	0.188	0	0
**14**	LGMN	1.443957831	0.692	0.17	0	0
**14**	FOLR2	1.416106619	0.71	0.006	0	0
**14**	CD83	1.40164922	0.736	0.107	0	0
**14**	FTH1	1.395379996	1	0.95	0	0
**14**	CST3	1.365564078	0.998	0.792	0	0
**14**	BCL2A1	1.329665893	0.464	0.035	0	0
**14**	HLA-DPA1	1.324828698	0.783	0.158	0	0
**14**	MS4A6A	1.236133274	0.671	0.025	0	0
**14**	PLEK	1.234900433	0.646	0.032	0	0
**14**	HLA-DPB1	1.227722375	0.788	0.176	0	0
**14**	CTSL	1.227588647	0.659	0.243	0	0
**14**	SAT1	1.22521329	0.998	0.787	0	0
**14**	MS4A7	1.156465439	0.647	0.011	0	0
**14**	CD14	1.148566397	0.577	0.057	0	0
**14**	CTSZ	1.121651948	0.692	0.191	0	0
**14**	IER3	1.105604594	0.98	0.776	0	0
**14**	LAPTM5	1.10328091	0.663	0.093	0	0
**14**	CTSC	1.101609868	0.662	0.225	0	0
**14**	CTSD	1.156265264	0.778	0.495	1.41E-265	3.35E-261
**15**	HLA-DRA	3.297387094	0.996	0.22	0	0
**15**	HLA-DPB1	3.242868501	0.984	0.172	0	0
**15**	LYZ	3.229776492	0.965	0.027	0	0
**15**	HLA-DRB1	3.098190061	0.987	0.184	0	0
**15**	HLA-DPA1	2.994071639	0.979	0.153	0	0
**15**	CD74	2.954300571	0.998	0.356	0	0
**15**	CXCL8	2.861779523	0.833	0.087	0	0
**15**	G0S2	2.828201695	0.745	0.075	0	0
**15**	HLA-DQA1	2.806684774	0.955	0.076	0	0

**15**	HLA-DQB1	2.743052044	0.962	0.115	0	0
**15**	IL1B	2.550244952	0.772	0.024	0	0
**15**	SRGN	2.344776928	0.947	0.174	0	0
**15**	GPR183	2.122127102	0.847	0.068	0	0
**15**	FCER1A	2.119045548	0.803	0.013	0	0
**15**	INSIG1	2.045886711	0.859	0.214	0	0
**15**	C15orf48	1.975722785	0.728	0.03	0	0
**15**	BCL2A1	1.899470622	0.798	0.026	0	0
**15**	TYROBP	1.880973817	0.94	0.053	0	0
**15**	CST3	1.853602613	0.997	0.792	0	0
**15**	SAT1	1.839121122	0.989	0.788	0	0
**15**	PLAUR	1.831938401	0.895	0.186	0	0
**15**	LST1	1.79832816	0.927	0.029	0	0
**15**	CD83	1.782532544	0.864	0.104	0	0
**15**	COTL1	1.755993406	0.948	0.164	0	0
**15**	CXCL3	1.727904853	0.505	0.128	0	0
**15**	FCER1G	1.688750527	0.893	0.047	0	0
**15**	HLA-DMA	1.592604607	0.923	0.103	0	0
**15**	AIF1	1.540011422	0.9	0.036	0	0
**15**	PLEK	1.537494536	0.842	0.027	0	0
**15**	CLEC10A	1.528415242	0.764	0.009	0	0
**15**	FTH1	1.459511885	1	0.95	0	0
**15**	CPVL	1.415797042	0.741	0.052	0	0
**15**	BIRC3	1.372117149	0.781	0.156	0	0
**15**	RGS2	1.362943523	0.741	0.182	0	0
**15**	REL	1.361847644	0.898	0.425	0	0
**15**	HLA-DRB5	1.353177853	0.273	0.038	0	0
**15**	CD1C	1.337959744	0.666	0.003	0	0
**15**	CCR7	1.29133497	0.422	0.018	0	0
**15**	SERPINB9	1.286042099	0.733	0.069	0	0
**15**	HLA-DMB	1.279460306	0.814	0.05	0	0
**15**	RGS1	1.270100393	0.573	0.042	0	0
**15**	LGALS2	1.253935085	0.709	0.004	0	0
**15**	CYBA	1.23291901	0.95	0.323	0	0
**15**	TMSB4X	1.228153614	1	0.993	0	0
**15**	NAMPT	1.205391297	0.877	0.455	0	0
**15**	DUSP2	1.199907988	0.797	0.176	0	0
**15**	CXCL2	1.190111592	0.573	0.199	0	0
**15**	EMP3	1.178459385	0.931	0.4	0	0
**15**	CCL3	1.120609773	0.348	0.037	0	0
**15**	ID2	1.133108692	0.836	0.596	2.81E-296	6.68E-292
**16**	POSTN	1.248631345	0.65	0.2	0	0
**16**	COL1A1	1.173927063	0.961	0.482	0	0
**16**	COL1A2	1.165962865	0.961	0.469	0	0
**16**	COL6A2	1.000339441	0.958	0.496	0	0
**16**	LUM	0.971908548	0.869	0.296	0	0
**16**	GPC3	0.963260415	0.508	0.087	0	0
**16**	MFAP4	0.962412568	0.782	0.267	0	0
**16**	HTRA1	0.960727541	0.746	0.269	0	0
**16**	TNMD	0.94718035	0.333	0.029	0	0
**16**	LGALS1	0.916727687	0.986	0.666	0	0
**16**	ASPN	0.900795006	0.51	0.109	0	0
**16**	COL6A1	0.870320187	0.9	0.427	0	0
**16**	MEG3	0.861717631	0.681	0.176	0	0
**16**	COL3A1	0.828679889	0.882	0.386	0	0
**16**	PTN	0.782991699	0.591	0.141	0	0
**16**	MFAP5	0.781750202	0.528	0.114	0	0
**16**	COL6A3	0.724336701	0.666	0.204	0	0
**16**	DPT	0.683592311	0.62	0.165	0	0
**16**	VCAN	0.636945131	0.565	0.153	0	0
**16**	COL11A1	0.62865461	0.29	0.013	0	0
**16**	TNN	0.602159482	0.313	0.012	0	0
**16**	F2R	0.596571732	0.499	0.139	0	0
**16**	MXRA8	0.592916604	0.666	0.201	0	0

**16**	TMEM119	0.548047127	0.412	0.094	0	0
**16**	MMP2	0.54773998	0.771	0.247	0	0
**16**	DCN	0.532042624	0.984	0.553	0	0
**16**	EDNRA	0.523187895	0.37	0.05	0	0
**16**	LAMC3	0.50353163	0.357	0.041	0	0
**16**	SPARC	0.942748645	0.935	0.592	2.48E-303	5.91E-299
**16**	PCOLCE	0.583386563	0.764	0.314	5.26E-292	1.25E-287
**16**	CD99	0.651234151	0.949	0.698	3.48E-290	8.29E-286
**16**	PRELP	0.613778291	0.444	0.13	4.06E-270	9.66E-266
**16**	11-Sep	0.471962379	0.48	0.172	2.42E-227	5.77E-223
**16**	PRRX1	0.477374157	0.555	0.215	2.08E-220	4.94E-216
**16**	DKK3	0.500912022	0.481	0.181	4.21E-215	1.00E-210
**16**	MDK	0.550947263	0.489	0.186	8.99E-209	2.14E-204
**16**	LAPTM4A	0.493950797	0.989	0.898	2.83E-199	6.74E-195
**16**	C6orf48	0.580028713	0.814	0.606	9.80E-199	2.33E-194
**16**	ZBTB20	0.55353145	0.602	0.297	9.00E-194	2.14E-189
**16**	SELENOP	0.486851514	0.882	0.63	1.14E-193	2.72E-189
**16**	VIM	0.588182575	0.988	0.707	1.40E-183	3.34E-179
**16**	S100A6	0.503844507	0.997	0.823	6.33E-177	1.51E-172
**16**	TCF4	0.521257409	0.633	0.329	1.56E-174	3.72E-170
**16**	PRSS23	0.509880318	0.472	0.188	4.59E-173	1.09E-168
**16**	SOD3	0.534966926	0.735	0.392	6.05E-172	1.44E-167
**16**	CTGF	0.525562419	0.533	0.26	1.11E-124	2.63E-120
**16**	BGN	0.695078526	0.586	0.332	1.31E-124	3.12E-120
**16**	IGFBP6	0.624421172	0.382	0.158	1.93E-112	4.59E-108
**16**	MRPS6	0.483999873	0.501	0.326	1.19E-74	2.83E-70
**16**	HES1	0.47283778	0.745	0.597	1.38E-48	3.28E-44
**17**	ACKR1	1.766103545	0.723	0.058	0	0
**17**	PLVAP	1.61176759	0.849	0.066	0	0
**17**	AQP1	1.583184894	0.885	0.187	0	0
**17**	GNG11	1.458884568	0.923	0.253	0	0
**17**	SPARCL1	1.454809771	0.951	0.338	0	0
**17**	IFI27	1.417851085	0.992	0.466	0	0
**17**	CD74	1.298284657	0.965	0.36	0	0
**17**	RAMP2	1.285995514	0.821	0.129	0	0
**17**	RAMP3	1.275758982	0.723	0.065	0	0
**17**	PECAM1	1.247736754	0.77	0.084	0	0
**17**	VWF	1.200007989	0.707	0.068	0	0
**17**	IFITM3	1.178027076	1	0.757	0	0
**17**	CLDN5	1.126455925	0.685	0.077	0	0
**17**	ENG	1.11578297	0.748	0.142	0	0
**17**	ECSCR	1.102680261	0.748	0.069	0	0
**17**	NPDC1	1.088309724	0.782	0.176	0	0
**17**	EGFL7	1.084506934	0.75	0.089	0	0
**17**	A2M	1.000925968	0.747	0.186	0	0
**17**	TMSB10	0.982875986	1	0.973	0	0
**17**	GIMAP7	0.972583833	0.642	0.07	0	0
**17**	EMCN	0.903102846	0.652	0.093	0	0
**17**	FKBP1A	0.901226444	0.939	0.684	0	0
**17**	CCL14	0.896230596	0.573	0.044	0	0
**17**	IGFBP7	0.88554233	0.999	0.641	0	0
**17**	PCAT19	0.87740258	0.674	0.068	0	0
**17**	BCAM	0.875588517	0.757	0.2	0	0
**17**	CRIP2	0.866092115	0.8	0.423	0	0
**17**	ADGRL4	0.864436331	0.633	0.063	0	0
**17**	CLEC14A	0.854485069	0.605	0.072	0	0
**17**	TSPAN7	0.848627472	0.602	0.063	0	0
**17**	IFITM2	0.831300886	0.935	0.553	0	0
**17**	TMSB4X	0.828101306	1	0.993	0	0
**17**	HSPG2	0.827196587	0.672	0.22	0	0
**17**	HLA-DRB1	0.824406327	0.866	0.191	0	0
**17**	CD34	0.8242484	0.639	0.138	0	0
**17**	HLA-E	0.808758533	0.998	0.881	0	0
**17**	RBP7	0.802318516	0.513	0.083	0	0

**17**	COL15A1	0.754603631	0.582	0.155	0	0
**17**	ESAM	0.729726855	0.609	0.111	0	0
**17**	RNASE1	0.726709016	0.717	0.094	0	0
**17**	MYL12A	0.718378327	0.985	0.835	0	0
**17**	TM4SF1	0.709593887	0.813	0.284	0	0
**17**	SNCG	0.708247433	0.625	0.132	0	0
**17**	TGFBR2	0.703463129	0.614	0.182	0	0
**17**	IFITM1	0.708480546	0.678	0.266	4.87E-300	1.16E-295
**17**	ADIRF	0.775977177	0.952	0.627	1.06E-258	2.51E-254
**17**	CCDC85B	0.747151365	0.763	0.516	2.92E-248	6.96E-244
**17**	ID1	0.950430961	0.72	0.4	6.01E-242	1.43E-237
**17**	TXNIP	0.819719315	0.79	0.523	2.59E-229	6.17E-225
**17**	CLU	0.997649062	0.613	0.351	2.06E-143	4.91E-139
**18**	FABP4	3.523489826	0.884	0.123	0	0
**18**	RBP7	2.583933355	0.914	0.074	0	0
**18**	CD36	2.207234099	0.843	0.068	0	0
**18**	GNG11	1.986585467	1	0.252	0	0
**18**	CLDN5	1.946970212	0.929	0.071	0	0
**18**	A2M	1.938782139	0.99	0.18	0	0
**18**	RGS5	1.887271304	0.789	0.078	0	0
**18**	AQP1	1.808798286	0.961	0.185	0	0
**18**	IFI27	1.758427389	0.999	0.466	0	0
**18**	PLVAP	1.712806231	0.92	0.065	0	0
**18**	SLC9A3R2	1.612989347	0.942	0.151	0	0
**18**	BST2	1.601024538	0.971	0.195	0	0
**18**	RAMP2	1.600069032	0.974	0.125	0	0
**18**	COL4A1	1.553630319	0.889	0.134	0	0
**18**	PECAM1	1.542484755	0.974	0.079	0	0
**18**	SPARCL1	1.538765089	0.999	0.337	0	0
**18**	EGFL7	1.501880732	0.9	0.085	0	0
**18**	VWF	1.440241399	0.929	0.063	0	0
**18**	COL4A2	1.408094694	0.909	0.152	0	0
**18**	TM4SF1	1.338722561	0.98	0.28	0	0
**18**	COL15A1	1.330883797	0.935	0.147	0	0
**18**	RAMP3	1.312034859	0.836	0.062	0	0
**18**	NDUFA4L2	1.308612952	0.771	0.23	0	0
**18**	CAV1	1.303855922	0.996	0.651	0	0
**18**	ADGRL4	1.262791533	0.922	0.057	0	0
**18**	IGFBP7	1.259254085	1	0.641	0	0
**18**	ESAM	1.245452731	0.948	0.103	0	0
**18**	ENG	1.214435251	0.933	0.138	0	0
**18**	MGLL	1.205628183	0.92	0.193	0	0
**18**	EPAS1	1.184580389	0.953	0.19	0	0
**18**	CD34	1.180888762	0.922	0.132	0	0
**18**	VAMP5	1.177230823	0.954	0.265	0	0
**18**	CRIP2	1.170026122	0.977	0.419	0	0
**18**	CD300LG	1.151461309	0.63	0.009	0	0
**18**	TINAGL1	1.137102264	0.935	0.141	0	0
**18**	ACKR1	1.131304114	0.478	0.064	0	0
**18**	NPDC1	1.126511703	0.94	0.173	0	0
**18**	ANGPT2	1.11852182	0.733	0.093	0	0
**18**	PCAT19	1.115999642	0.906	0.063	0	0
**18**	TGFBR2	1.093138705	0.919	0.175	0	0
**18**	CLEC14A	1.084631727	0.852	0.066	0	0
**18**	IGFBP2	1.077959976	0.91	0.118	0	0
**18**	FAM241A	1.077368612	0.867	0.153	0	0
**18**	CD74	1.063710481	0.974	0.36	0	0
**18**	ECSCR	1.061665993	0.911	0.065	0	0
**18**	FKBP1A	1.059620124	0.983	0.683	0	0
**18**	TMSB10	1.055339955	1	0.973	0	0
**18**	LMCD1	1.036467579	0.864	0.131	0	0
**18**	TIMP3	1.034182254	0.978	0.386	0	0
**18**	EMCN	1.030912046	0.898	0.088	0	0
**19**	AQP5	2.356120258	0.733	0.021	0	0

**19**	SNORC	2.171497307	0.744	0.015	0	0
**19**	KRT19	1.885147375	0.679	0.032	0	0
**19**	GAPDH	1.759623037	1	0.996	0	0
**19**	ZG16B	1.731288429	0.446	0.057	0	0
**19**	CA6	1.621725597	0.517	0.007	0	0
**19**	KRT18	1.599839477	0.645	0.074	0	0
**19**	KRT7	1.55257752	0.604	0.036	0	0
**19**	CLDN10	1.488940925	0.471	0.01	0	0
**19**	KRT8	1.428738655	0.565	0.059	0	0
**19**	SFRP1	1.398337262	0.458	0.11	0	0
**19**	S100A1	1.26310774	0.571	0.014	0	0
**19**	PPP1R1B	1.227462479	0.567	0.018	0	0
**19**	KRT15	1.202912986	0.795	0.176	0	0
**19**	DBI	1.198878122	0.995	0.749	0	0
**19**	NDRG2	1.133997643	0.622	0.229	0	0
**19**	TPD52L1	0.885482715	0.474	0.101	0	0
**19**	ELF3	0.875220185	0.329	0.029	0	0
**19**	STAC2	0.792149817	0.332	0.013	0	0
**19**	DNER	0.78810041	0.29	0.008	0	0
**19**	TESC	0.750827649	0.356	0.038	0	0
**19**	NCALD	0.740395909	0.367	0.037	0	0
**19**	ROPN1B	0.738272906	0.375	0.007	0	0
**19**	EPCAM	0.703031379	0.326	0.039	0	0
**19**	COX7C	1.003199961	0.998	0.951	1.01E-297	2.39E-293
**19**	AKR1C1	0.927242295	0.417	0.106	4.47E-282	1.07E-277
**19**	CALM2	0.823880026	1	0.957	1.40E-278	3.34E-274
**19**	CD24	0.924712012	0.38	0.086	7.27E-278	1.73E-273
**19**	SLC12A2	1.549025428	0.358	0.081	5.68E-274	1.35E-269
**19**	FXYD3	0.840519618	0.983	0.481	8.59E-273	2.05E-268
**19**	CHCHD10	1.119389288	0.668	0.33	3.36E-271	8.00E-267
**19**	COX5B	0.871898273	0.997	0.857	1.15E-265	2.74E-261
**19**	LDHA	1.067273007	0.992	0.936	2.58E-261	6.14E-257
**19**	PDCD4	1.189736444	0.595	0.271	4.09E-250	9.72E-246
**19**	ATP5MC3	0.905575404	0.977	0.702	1.06E-242	2.51E-238
**19**	ATP1B1	1.203222727	0.44	0.137	1.22E-231	2.90E-227
**19**	HINT1	0.728985162	0.998	0.903	7.40E-224	1.76E-219
**19**	PPDPF	0.752157528	0.995	0.874	1.04E-222	2.47E-218
**19**	COX7A2	0.718326076	0.991	0.862	1.06E-177	2.53E-173
**19**	NDUFA1	0.692200238	0.959	0.68	5.10E-174	1.21E-169
**19**	CD59	0.880332696	0.971	0.61	6.09E-173	1.45E-168
**19**	PEBP1	0.747165269	0.99	0.82	2.14E-170	5.08E-166
**19**	ENO1	0.828531887	0.977	0.823	1.99E-165	4.73E-161
**19**	UQCRB	0.70273757	0.979	0.86	7.20E-165	1.71E-160
**19**	ATP1A1	0.867128143	0.976	0.627	6.39E-157	1.52E-152
**19**	SOD2	0.693690863	0.9	0.496	8.22E-143	1.96E-138
**19**	ATP5F1B	0.716558004	0.98	0.814	4.97E-135	1.18E-130
**19**	CLDN4	0.808252803	0.362	0.205	1.86E-53	4.42E-49
**19**	SDC4	1.035481084	0.336	0.218	2.67E-40	6.35E-36
**19**	MT-CO1	0.789542364	0.999	0.995	6.30E-11	1.50E-06
**20**	KRT15	3.078848369	1	0.172	0	0
**20**	KRT14	1.535970999	0.998	0.669	0	0
**20**	DST	1.441361864	0.943	0.324	0	0
**20**	KRT5	1.4090983	1	0.488	0	0
**20**	S100A2	1.316276552	0.966	0.378	0	0
**20**	COL17A1	1.238602458	0.909	0.141	0	0
**20**	POSTN	0.822755389	0.844	0.199	0	0
**20**	SOX15	0.768468408	0.784	0.269	0	0
**20**	HOPX	0.735092205	0.863	0.383	0	0
**20**	ARL4A	0.730829614	0.769	0.288	0	0
**20**	FBXW7	0.721378673	0.75	0.241	0	0
**20**	ALDH3A1	0.706603252	0.636	0.117	0	0
**20**	TGFBI	0.706174594	0.796	0.275	0	0
**20**	CXADR	0.674144147	0.805	0.242	0	0
**20**	SYT8	0.657763798	0.481	0.091	0	0

**20**	NDFIP2	0.534942724	0.724	0.246	0	0
**20**	LAMB3	0.532159416	0.581	0.087	0	0
**20**	FGFR3	0.520288479	0.603	0.165	0	0
**20**	CXCL14	0.759409926	0.993	0.76	1.62E-300	3.86E-296
**20**	ATP1B3	0.723920032	0.94	0.64	7.21E-297	1.72E-292
**20**	ALDH3A2	0.542940747	0.699	0.249	3.38E-281	8.05E-277
**20**	HSPA2	0.723066681	0.804	0.366	2.01E-279	4.79E-275
**20**	SPINT2	0.569331987	0.88	0.389	4.08E-271	9.72E-267
**20**	IMPA2	0.53021361	0.7	0.246	2.01E-269	4.78E-265
**20**	CBR1	0.592748579	0.804	0.356	2.04E-269	4.87E-265
**20**	AQP3	0.718111524	0.952	0.54	6.09E-262	1.45E-257
**20**	C19orf33	0.523766984	0.834	0.34	2.41E-253	5.73E-249
**20**	ERRFI1	0.831815527	0.692	0.279	3.06E-249	7.29E-245
**20**	AREG	1.247852152	0.595	0.209	9.20E-244	2.19E-239
**20**	ADRB2	0.712053675	0.656	0.242	2.14E-242	5.10E-238
**20**	TOB1	0.757491937	0.806	0.437	4.71E-241	1.12E-236
**20**	FAM213A	0.56533439	0.706	0.276	6.69E-240	1.59E-235
**20**	CCL27	0.7687491	0.633	0.229	4.03E-225	9.59E-221
**20**	KLF5	0.708764775	0.85	0.479	1.15E-219	2.74E-215
**20**	PKM	0.546826031	0.959	0.818	1.40E-209	3.32E-205
**20**	PERP	0.56748657	1	0.708	3.06E-206	7.29E-202
**20**	TGIF1	0.606318947	0.745	0.374	9.09E-201	2.16E-196
**20**	SGK1	0.637820513	0.743	0.354	3.62E-197	8.62E-193
**20**	MT1X	0.699415369	0.962	0.748	2.38E-195	5.67E-191
**20**	PMAIP1	0.5523102	0.669	0.287	1.78E-183	4.23E-179
**20**	ANKRD37	0.53028169	0.481	0.17	5.01E-173	1.19E-168
**20**	GPNMB	0.579739105	0.849	0.556	2.76E-168	6.56E-164
**20**	AMD1	0.637172327	0.792	0.571	4.90E-142	1.17E-137
**20**	DNAJA1	0.673584605	0.919	0.829	7.87E-141	1.87E-136
**20**	SLC38A2	0.532221747	0.878	0.686	6.32E-139	1.50E-134
**20**	ZC3H12A	0.529262599	0.725	0.443	1.47E-128	3.49E-124
**20**	MYC	0.566767423	0.863	0.702	3.38E-98	8.04E-94
**20**	CKS2	0.56867293	0.679	0.461	2.90E-91	6.90E-87
**20**	DDX5	0.538389084	0.956	0.935	6.64E-91	1.58E-86
**20**	ATF3	0.543699269	0.767	0.556	7.47E-86	1.78E-81
**21**	TPSB2	3.863621758	0.96	0.007	0	0
**21**	TPSAB1	3.520733161	0.978	0.005	0	0
**21**	CTSG	2.016982158	0.719	0.003	0	0
**21**	HPGD	1.934101421	0.738	0.076	0	0
**21**	FTH1	1.807330596	1	0.951	0	0
**21**	HPGDS	1.725033914	0.674	0.009	0	0
**21**	SELENOK	1.610485838	0.996	0.687	0	0
**21**	ANXA1	1.602556542	1	0.866	0	0
**21**	CD69	1.291495339	0.327	0.041	0	0
**21**	RGS13	1.228706504	0.528	0.002	0	0
**21**	GATA2	1.088911757	0.437	0.051	0	0
**21**	H3F3B	0.890378502	1	0.997	0	0
**21**	CPA3	0.82497721	0.343	0.001	0	0
**21**	MS4A2	0.809021363	0.354	0	0	0
**21**	FTL	0.736062902	1	0.97	0	0
**21**	FCER1G	0.687062937	0.413	0.064	0	0
**21**	IL1RL1	0.608024261	0.253	0.007	0	0
**21**	PTMA	0.705645189	1	0.997	5.05E-292	1.20E-287
**21**	GLUL	1.146304936	0.963	0.504	1.02E-280	2.42E-276
**21**	VWA5A	0.808598764	0.363	0.083	9.30E-272	2.21E-267
**21**	UBB	0.947967392	1	0.969	1.94E-251	4.62E-247
**21**	SRGN	0.544329795	0.602	0.188	6.88E-246	1.64E-241
**21**	EMP3	0.752730094	0.809	0.408	1.65E-206	3.92E-202
**21**	SDCBP	0.712612347	0.975	0.542	2.88E-202	6.85E-198
**21**	SGK1	0.786612393	0.736	0.354	5.22E-197	1.24E-192
**21**	RPL34	0.524524633	1	1	1.42E-186	3.38E-182
**21**	EIF1	0.501735043	1	0.999	7.96E-175	1.90E-170
**21**	SAT1	0.612517742	0.998	0.789	3.56E-173	8.48E-169
**21**	MT-CO2	0.464765839	1	0.995	2.04E-150	4.85E-146

**21**	CLIC1	0.637240924	1	0.88	7.23E-147	1.72E-142
**21**	RPL36AL	0.625871636	0.999	0.944	3.31E-133	7.87E-129
**21**	BIRC3	0.96165045	0.413	0.169	4.25E-131	1.01E-126
**21**	HSPH1	0.659758442	0.965	0.534	2.24E-129	5.34E-125
**21**	PRNP	0.610687825	0.926	0.544	1.58E-125	3.76E-121
**21**	LAPTM5	0.495321473	0.323	0.105	9.93E-121	2.36E-116
**21**	MALAT1	0.495446253	1	0.997	1.87E-119	4.45E-115
**21**	DNAJA1	0.625359017	0.999	0.827	9.22E-115	2.19E-110
**21**	HSPA8	0.639497826	1	0.931	4.99E-104	1.19E-99
**21**	SEC62	0.473449992	0.982	0.654	4.33E-99	1.03E-94
**21**	PRDX1	0.567741619	1	0.901	8.99E-98	2.14E-93
**21**	JUND	0.551298077	0.663	0.428	3.09E-83	7.35E-79
**21**	ELF1	0.789772176	0.415	0.26	1.06E-74	2.51E-70
**21**	SQSTM1	0.470334478	0.99	0.708	2.38E-69	5.67E-65
**21**	HSPE1	0.664740601	0.994	0.789	6.58E-60	1.57E-55
**21**	DNAJB1	0.669507748	0.998	0.843	2.98E-59	7.10E-55
**21**	SH3BGRL3	0.473283441	0.998	0.786	1.02E-56	2.43E-52
**21**	LMNA	0.439330653	1	0.96	4.90E-47	1.17E-42
**21**	PLIN2	0.668345314	0.383	0.304	2.65E-30	6.32E-26
**21**	RGS2	0.470035563	0.296	0.196	5.08E-29	1.21E-24
**21**	DNAJB9	0.540148631	0.342	0.273	7.83E-26	1.86E-21
**22**	GJB6	0.663511109	0.332	0.034	0	0
**22**	MT-CO3	1.067059106	1	0.995	2.26E-254	5.38E-250
**22**	MT-CO2	0.944874903	0.999	0.995	1.14E-237	2.72E-233
**22**	GJB2	0.614097012	0.33	0.066	5.99E-228	1.43E-223
**22**	MT-ND5	0.788267543	0.99	0.949	5.99E-204	1.42E-199
**22**	MT-ND1	0.754915478	0.999	0.994	1.37E-200	3.25E-196
**22**	MT-CYB	0.787000795	0.999	0.995	1.68E-200	4.01E-196
**22**	MT-ATP6	0.787853481	0.998	0.993	1.14E-191	2.72E-187
**22**	MT-ND2	0.749278758	0.999	0.995	4.89E-184	1.16E-179
**22**	MT-CO1	0.893297678	1	0.995	7.54E-177	1.79E-172
**22**	MT-ND4	0.80319778	0.999	0.995	1.14E-170	2.72E-166
**22**	RPS6	0.459855685	1	1	9.92E-168	2.36E-163
**22**	RPL10A	0.453883199	1	0.999	8.53E-151	2.03E-146
**22**	RPS18	0.480570776	1	1	4.90E-140	1.17E-135
**22**	RPL13A	0.401408883	1	1	4.33E-131	1.03E-126
**22**	RPL7	0.394308419	1	1	5.12E-130	1.22E-125
**22**	RPL12	0.466070093	1	0.999	3.24E-129	7.72E-125
**22**	RPS5	0.422524126	1	0.999	2.46E-128	5.86E-124
**22**	MGST1	0.688066774	0.389	0.131	1.56E-127	3.72E-123
**22**	MT-ND3	0.7970054	0.997	0.992	8.11E-127	1.93E-122
**22**	RPL3	0.422808307	1	1	3.65E-126	8.69E-122
**22**	RPS19	0.421667452	1	1	6.22E-119	1.48E-114
**22**	RPL37A	0.36014027	1	1	3.08E-118	7.33E-114
**22**	RPL8	0.355109183	1	1	7.77E-116	1.85E-111
**22**	RPS24	0.366899767	1	0.998	6.05E-112	1.44E-107
**22**	RPL36	0.416107292	1	0.999	2.09E-111	4.97E-107
**22**	RPS3	0.402665677	1	0.999	2.17E-111	5.16E-107
**22**	FAM84A	0.360694766	0.323	0.108	9.71E-104	2.31E-99
**22**	RPLP1	0.407055644	1	1	2.00E-102	4.76E-98
**22**	FXYD3	0.490649336	0.835	0.485	2.03E-101	4.84E-97
**22**	RPL31	0.377140082	1	0.999	1.40E-100	3.34E-96
**22**	KRT15	0.511709038	0.455	0.184	1.41E-99	3.36E-95
**22**	COX4I1	0.410402129	0.998	0.967	5.70E-98	1.36E-93
**22**	RPS21	0.424969705	0.999	0.994	3.76E-97	8.96E-93
**22**	MT1X	0.380850454	0.954	0.749	3.89E-89	9.25E-85
**22**	RPLP0	0.36116346	1	0.999	2.17E-88	5.18E-84
**22**	RPL36A	0.408953839	1	0.988	6.20E-86	1.48E-81
**22**	UQCRH	0.39024379	0.947	0.819	1.98E-85	4.72E-81
**22**	SFRP1	0.387111114	0.311	0.114	1.51E-83	3.58E-79
**22**	ATP1B1	0.491315189	0.343	0.139	1.49E-79	3.56E-75
**22**	RPS2	0.435738692	1	1	2.85E-78	6.77E-74
**22**	RPS17	0.374225531	1	0.996	2.66E-76	6.34E-72
**22**	RPL28	0.401769086	1	0.999	4.56E-76	1.09E-71

**22**	SNHG8	0.401805634	0.891	0.705	1.02E-68	2.44E-64
**22**	DAPL1	0.512289852	0.505	0.271	1.30E-63	3.09E-59
**22**	CHCHD10	0.467396079	0.526	0.333	2.77E-59	6.59E-55
**22**	C19orf33	0.436281031	0.568	0.346	1.95E-56	4.65E-52
**22**	DEFB1	0.769882569	0.357	0.199	1.75E-46	4.16E-42
**22**	ATP1A1	0.592177333	0.748	0.632	3.81E-42	9.08E-38
**22**	ATP1B3	0.63007137	0.761	0.644	5.94E-36	1.41E-31
**23**	RGS5	2.234573773	0.866	0.081	0	0
**23**	NDUFA4L2	1.7223768	0.847	0.233	0	0
**23**	IGFBP7	1.352209397	1	0.644	0	0
**23**	CALD1	1.244220003	0.986	0.576	0	0
**23**	CPE	1.139131088	0.858	0.249	0	0
**23**	TINAGL1	1.087001896	0.788	0.149	0	0
**23**	ADIRF	1.086848901	0.999	0.629	0	0
**23**	CD36	1.063997354	0.493	0.079	0	0
**23**	LHFPL6	1.052802431	0.818	0.259	0	0
**23**	STEAP4	1.034262831	0.631	0.08	0	0
**23**	COX4I2	1.015031127	0.626	0.057	0	0
**23**	IFITM3	1.009670387	0.998	0.759	0	0
**23**	TAGLN	0.959046971	0.918	0.29	0	0
**23**	7-Sep	0.934695419	0.938	0.626	0	0
**23**	MYL9	0.918342919	0.971	0.454	0	0
**23**	NR2F2	0.903441951	0.714	0.154	0	0
**23**	ACTA2	0.900942287	0.787	0.18	0	0
**23**	LGI4	0.780829398	0.569	0.064	0	0
**23**	EPS8	0.755322341	0.643	0.159	0	0
**23**	FAM162B	0.748659163	0.509	0.039	0	0
**23**	4-Sep	0.729162831	0.517	0.075	0	0
**23**	SYNPO2	0.66362574	0.52	0.115	0	0
**23**	HIGD1B	0.646053857	0.376	0.022	0	0
**23**	GPM6B	0.629921439	0.502	0.097	0	0
**23**	RERG	0.609550047	0.512	0.08	0	0
**23**	MYO1B	0.608946118	0.503	0.108	0	0
**23**	NOTCH3	0.742550744	0.613	0.175	2.44E-302	5.80E-298
**23**	FABP4	0.972688034	0.553	0.134	2.11E-301	5.02E-297
**23**	ARHGDIB	0.953630586	0.723	0.264	6.68E-298	1.59E-293
**23**	PRRX1	0.745186166	0.692	0.216	2.53E-296	6.02E-292
**23**	A2M	0.661374941	0.686	0.191	5.02E-294	1.19E-289
**23**	PDGFRB	0.759391029	0.647	0.194	5.93E-288	1.41E-283
**23**	TFPI	0.714012502	0.595	0.167	4.14E-287	9.86E-283
**23**	COL18A1	0.890498252	0.754	0.284	7.50E-287	1.79E-282
**23**	CYGB	0.766911461	0.534	0.139	1.24E-269	2.95E-265
**23**	SPARCL1	0.772891772	0.867	0.344	4.60E-268	1.10E-263
**23**	C20orf27	0.697070549	0.552	0.167	4.92E-261	1.17E-256
**23**	SPARC	0.731461326	0.987	0.594	7.16E-261	1.70E-256
**23**	TIMP3	0.90140937	0.828	0.392	1.20E-257	2.85E-253
**23**	MFGE8	0.847410885	0.797	0.362	3.98E-257	9.48E-253
**23**	GPX3	0.716313942	0.6	0.184	6.80E-241	1.62E-236
**23**	IFITM1	0.745557533	0.707	0.268	1.13E-239	2.69E-235
**23**	COL4A2	0.635439402	0.525	0.164	8.20E-204	1.95E-199
**23**	IFITM2	0.709691219	0.902	0.557	3.93E-199	9.36E-195
**23**	COL4A1	0.609989856	0.48	0.146	1.51E-186	3.59E-182
**23**	LAPTM4A	0.605517131	0.992	0.899	1.18E-185	2.81E-181
**23**	LGALS1	0.625396867	0.997	0.669	6.70E-165	1.59E-160
**23**	MT2A	0.712066707	0.981	0.801	6.72E-165	1.60E-160
**23**	THY1	0.646741873	0.576	0.213	3.02E-150	7.18E-146
**23**	S100A4	0.622817936	0.96	0.673	2.10E-138	4.99E-134
**24**	KRT6A	3.155013462	0.981	0.112	0	0
**24**	KRT16	2.672338562	0.916	0.143	0	0
**24**	KRT6B	2.467449613	0.771	0.046	0	0
**24**	KRT5	2.235254077	0.977	0.491	0	0
**24**	KRT14	2.111785623	0.987	0.671	0	0
**24**	KRT6C	1.819756632	0.646	0.017	0	0
**24**	KRT17	1.761204791	0.75	0.196	0	0

**24**	S100A2	1.744485798	0.931	0.381	0	0
**24**	LY6D	1.455725843	0.975	0.494	0	0
**24**	MT1X	1.405557661	0.986	0.749	0	0
**24**	LGALS7	1.327436062	0.916	0.257	0	0
**24**	S100A14	1.313944636	0.993	0.534	0	0
**24**	CALML3	1.196888747	0.824	0.175	0	0
**24**	TPPP3	1.093434507	0.838	0.326	0	0
**24**	DSP	1.058636786	0.964	0.485	0	0
**24**	C19orf33	1.053232713	0.908	0.341	0	0
**24**	S100A16	0.972516975	0.976	0.596	0	0
**24**	JUP	0.893869016	0.924	0.331	0	0
**24**	PKP1	0.885974326	0.848	0.237	0	0
**24**	TRIM29	0.884282382	0.916	0.273	0	0
**24**	IGFL1	0.821691045	0.346	0.006	0	0
**24**	PKP3	0.765034899	0.843	0.228	0	0
**24**	IMPA2	0.754584634	0.842	0.246	0	0
**24**	LAD1	0.743327367	0.744	0.164	0	0
**24**	DSC3	0.82617956	0.906	0.354	4.60E-304	1.10E-299
**24**	SPINT2	0.755262709	0.918	0.391	2.29E-302	5.45E-298
**24**	RHOV	0.663377417	0.686	0.189	2.09E-300	4.97E-296
**24**	PERP	1.043198421	1	0.709	1.15E-294	2.74E-290
**24**	FXYD3	0.766736818	0.976	0.483	1.32E-277	3.14E-273
**24**	DAPL1	0.6184722	0.812	0.267	3.60E-272	8.57E-268
**24**	FGFBP1	0.649773886	0.667	0.183	1.43E-268	3.40E-264
**24**	MT1G	0.650644012	0.458	0.098	7.22E-267	1.72E-262
**24**	HOPX	0.717315419	0.886	0.385	2.90E-265	6.90E-261
**24**	AQP3	0.849036241	0.984	0.541	1.44E-263	3.44E-259
**24**	LGALS7B	0.855846481	0.978	0.528	1.78E-258	4.24E-254
**24**	CLTB	0.676216805	0.955	0.661	3.48E-256	8.29E-252
**24**	SERPINB5	0.671344133	0.912	0.379	3.48E-253	8.28E-249
**24**	GSTP1	0.666578845	0.992	0.926	4.31E-245	1.02E-240
**24**	CA2	0.624911536	0.643	0.193	2.97E-242	7.06E-238
**24**	FAM213A	0.604647101	0.755	0.277	5.69E-226	1.35E-221
**24**	SFN	0.786917028	1	0.684	1.21E-220	2.89E-216
**24**	S100A8	0.60005979	0.363	0.072	2.55E-220	6.06E-216
**24**	IFI27	0.894844993	0.861	0.472	9.46E-200	2.25E-195
**24**	DMKN	1.011969334	0.919	0.609	2.31E-197	5.49E-193
**24**	FABP5	1.097396845	0.845	0.533	7.67E-190	1.82E-185
**24**	PDLIM1	0.618252249	0.842	0.47	3.00E-179	7.13E-175
**24**	COL17A1	0.600023121	0.497	0.15	1.58E-161	3.76E-157
**24**	MT1E	0.80440509	0.773	0.465	2.75E-144	6.54E-140
**24**	KRT1	0.904407703	0.849	0.581	3.91E-134	9.31E-130
**24**	KRT10	0.973373111	0.928	0.854	2.50E-63	5.95E-59
**25**	S100A8	3.463071706	0.989	0.063	0	0
**25**	S100A9	3.012122806	0.928	0.064	0	0
**25**	S100A7	2.83996266	0.841	0.028	0	0
**25**	SPRR1B	1.792988834	0.442	0.068	0	0
**25**	LGALS7B	1.51827461	0.994	0.528	0	0
**25**	KRT6A	1.382413705	0.601	0.118	0	0
**25**	LY6D	1.37657574	0.95	0.495	0	0
**25**	S100A14	1.153560105	0.978	0.534	0	0
**25**	CSTA	1.117506296	0.783	0.258	0	0
**25**	CCL27	1.102086192	0.802	0.228	0	0
**25**	TMEM45A	1.044506578	0.818	0.292	0	0
**25**	IMPA2	0.774810404	0.792	0.247	0	0
**25**	CALML3	1.060724357	0.659	0.177	4.54E-306	1.08E-301
**25**	TRIM29	0.694036841	0.83	0.275	4.27E-296	1.02E-291
**25**	KRT16	1.598858438	0.588	0.148	3.05E-290	7.27E-286
**25**	SFN	1.138595293	1	0.684	7.75E-289	1.84E-284
**25**	DAPL1	0.726767188	0.789	0.267	4.58E-276	1.09E-271
**25**	LYPD3	1.185238072	0.91	0.453	9.53E-276	2.27E-271
**25**	SPINK5	0.777542322	0.566	0.145	7.93E-266	1.89E-261
**25**	CRABP2	0.920940023	0.753	0.273	1.39E-261	3.30E-257
**25**	PERP	0.996084397	0.999	0.709	1.15E-258	2.73E-254

**25**	HMGA1	0.663904999	0.798	0.299	2.69E-256	6.41E-252
**25**	DEFB1	0.714384943	0.659	0.195	3.87E-255	9.20E-251
**25**	HBEGF	0.870414984	0.696	0.24	3.21E-251	7.64E-247
**25**	TACSTD2	0.981606432	0.954	0.537	7.98E-250	1.90E-245
**25**	PMAIP1	0.729663559	0.769	0.288	5.65E-246	1.35E-241
**25**	SBSN	1.380915236	0.737	0.289	1.40E-244	3.32E-240
**25**	AQP3	0.931457784	0.958	0.542	8.78E-239	2.09E-234
**25**	CA2	0.890031845	0.616	0.193	1.68E-237	4.01E-233
**25**	LGALS7	1.093501301	0.687	0.261	6.75E-229	1.61E-224
**25**	GLTP	0.752042777	0.69	0.254	1.65E-222	3.94E-218
**25**	SERPINB5	0.662496944	0.875	0.379	2.09E-222	4.96E-218
**25**	JUP	0.691553786	0.804	0.333	7.76E-219	1.85E-214
**25**	FGFBP1	0.871938521	0.605	0.184	4.25E-218	1.01E-213
**25**	KRTDAP	1.585126172	0.819	0.44	3.48E-216	8.29E-212
**25**	S100A11	0.657147199	0.999	0.951	8.93E-216	2.12E-211
**25**	S100A16	0.680761864	0.934	0.597	3.31E-207	7.87E-203
**25**	DMKN	1.154694203	0.897	0.609	7.25E-200	1.72E-195
**25**	RPS4Y1	0.718740211	0.654	0.24	4.14E-191	9.85E-187
**25**	CSTB	0.794175188	0.938	0.713	7.68E-190	1.83E-185
**25**	TUBA1C	0.675814839	0.914	0.58	1.98E-188	4.71E-184
**25**	KRT1	1.289301809	0.857	0.581	3.90E-183	9.28E-179
**25**	CALML5	0.930011797	0.54	0.173	7.80E-175	1.86E-170
**25**	KRT10	1.226938119	0.956	0.854	1.36E-173	3.24E-169
**25**	KRT17	0.806892554	0.578	0.199	3.22E-170	7.67E-166
**25**	PHLDA2	1.043402193	0.82	0.509	2.85E-166	6.79E-162
**25**	FABP5	1.19100155	0.817	0.534	2.47E-149	5.89E-145
**25**	AREG	0.786027179	0.539	0.211	4.57E-125	1.09E-120
**25**	S100A2	1.142229482	0.607	0.386	2.13E-71	5.07E-67
**25**	KRT2	0.926359455	0.398	0.33	1.30E-12	3.09E-08
**26**	DCD	6.248746204	0.91	0.154	0	0
**26**	MUCL1	5.384126253	0.92	0.074	0	0
**26**	SCGB2A2	5.259035733	0.972	0.058	0	0
**26**	SCGB1B2P	4.055237009	0.932	0.022	0	0
**26**	SCGB1D2	3.896271685	0.853	0.025	0	0
**26**	PIP	3.648296276	0.707	0.013	0	0
**26**	AZGP1	2.376579253	0.795	0.019	0	0
**26**	KRT19	2.177905186	0.762	0.034	0	0
**26**	KRT7	1.946993173	0.742	0.037	0	0
**26**	AQP5	1.799972522	0.692	0.025	0	0
**26**	SLC12A2	1.619066375	0.623	0.078	0	0
**26**	KRT18	1.575799633	0.703	0.076	0	0
**26**	SNORC	1.405049849	0.333	0.025	0	0
**26**	PRR4	1.272462426	0.269	0.003	0	0
**26**	KRT8	1.270370483	0.619	0.061	0	0
**26**	TSPAN8	1.113067719	0.599	0.066	0	0
**26**	ATP1B1	1.104702505	0.576	0.136	0	0
**26**	ZG16B	1.097302565	0.438	0.059	0	0
**26**	PPP1R1B	1.032536438	0.559	0.021	0	0
**26**	ELF3	0.987478284	0.469	0.029	0	0
**26**	C5orf46	0.966595749	0.527	0.022	0	0
**26**	SERHL2	0.953228367	0.424	0.013	0	0
**26**	LRRC26	0.93564336	0.533	0.01	0	0
**26**	CD24	0.908768785	0.498	0.085	0	0
**26**	CLDN10	0.905345454	0.282	0.015	0	0
**26**	CALML5	0.867491008	0.698	0.171	0	0
**26**	SCGB2A1	0.768350638	0.308	0.004	0	0
**26**	CRACR2B	0.74042383	0.525	0.048	0	0
**26**	CLDN3	0.731513292	0.459	0.01	0	0
**26**	SOX9	0.728564374	0.445	0.064	0	0
**26**	SMIM22	0.727708738	0.481	0.01	0	0
**26**	CITED4	0.714772879	0.479	0.093	0	0
**26**	CLDN7	0.666057091	0.478	0.033	0	0
**26**	CXCL1	1.319461108	0.341	0.07	2.65E-207	6.30E-203
**26**	XBP1	0.847213379	0.89	0.5	1.47E-203	3.49E-199

**26**	FXYD3	0.669088323	0.964	0.484	6.08E-185	1.45E-180
**26**	SDC4	1.215091774	0.545	0.216	1.29E-170	3.06E-166
**26**	MT-CO3	0.939108735	1	0.995	1.13E-167	2.69E-163
**26**	CLDN4	0.838741484	0.565	0.202	2.64E-166	6.29E-162
**26**	SFRP1	1.195073019	0.394	0.113	1.55E-163	3.70E-159
**26**	MT-ND4	0.936921433	1	0.995	2.64E-163	6.27E-159
**26**	ATP1A1	0.73295271	0.952	0.629	4.35E-163	1.03E-158
**26**	MT-CO2	1.004824772	1	0.995	1.64E-154	3.91E-150
**26**	MT-CO1	0.973621054	1	0.995	5.17E-152	1.23E-147
**26**	DBI	0.710095936	0.976	0.75	3.43E-141	8.16E-137
**26**	MT-ND1	0.812724712	1	0.994	2.34E-137	5.57E-133
**26**	MT-CYB	0.720388639	1	0.995	2.08E-112	4.94E-108
**26**	MT-ATP6	0.750102327	1	0.993	2.00E-111	4.77E-107
**26**	CXCL2	0.893015843	0.47	0.205	7.58E-93	1.80E-88
**26**	MT-ND3	0.714853947	1	0.992	2.86E-89	6.80E-85
**27**	HIST1H4C	2.230176724	0.844	0.25	0	0
**27**	KRT5	1.531155818	0.973	0.492	0	0
**27**	STMN1	1.513598936	0.96	0.299	0	0
**27**	HMGB2	1.462925057	0.979	0.452	0	0
**27**	PTTG1	1.447670869	0.949	0.206	0	0
**27**	UBE2C	1.30750392	0.854	0.018	0	0
**27**	TUBA1B	1.264063105	1	0.934	0	0
**27**	NUSAP1	1.181576873	0.906	0.076	0	0
**27**	PCLAF	1.121206108	0.729	0.03	0	0
**27**	TOP2A	1.116926749	0.771	0.016	0	0
**27**	TK1	1.07350078	0.71	0.026	0	0
**27**	HMGB1	1.045089822	1	0.948	0	0
**27**	CDK1	1.042551433	0.804	0.022	0	0
**27**	CENPF	1.018762748	0.716	0.018	0	0
**27**	CKS1B	1.016095596	0.919	0.265	0	0
**27**	CDC20	0.906613693	0.654	0.037	0	0
**27**	SMC4	0.885399698	0.81	0.146	0	0
**27**	BIRC5	0.875405094	0.73	0.017	0	0
**27**	CENPW	0.868285629	0.804	0.07	0	0
**27**	TYMS	0.820912008	0.65	0.037	0	0
**27**	MT1G	0.779203509	0.59	0.097	0	0
**27**	CDKN3	0.774368321	0.629	0.02	0	0
**27**	PHGDH	0.773799815	0.817	0.235	0	0
**27**	MKI67	0.767783404	0.651	0.01	0	0
**27**	PBK	0.759050303	0.666	0.007	0	0
**27**	RRM2	0.730691605	0.534	0.011	0	0
**27**	CCNB1	0.728155395	0.529	0.043	0	0
**27**	CCNB2	0.703880209	0.593	0.012	0	0
**27**	H2AFZ	0.914001291	1	0.827	6.31E-280	1.50E-275
**27**	HMGN2	1.016198948	0.973	0.689	1.03E-277	2.44E-273
**27**	H2AFV	0.679235428	0.859	0.323	3.71E-277	8.83E-273
**27**	HINT1	0.759065825	0.997	0.904	1.09E-274	2.60E-270
**27**	TUBB	1.031432882	0.991	0.845	5.45E-258	1.30E-253
**27**	PTMA	0.725920847	1	0.997	8.90E-252	2.12E-247
**27**	S100A2	0.999615492	0.91	0.383	2.79E-244	6.64E-240
**27**	KRT14	1.310968394	0.966	0.672	1.05E-234	2.49E-230
**27**	UBE2S	0.7206341	0.779	0.282	2.03E-234	4.82E-230
**27**	PLP2	0.718800523	0.976	0.676	2.38E-233	5.66E-229
**27**	S100A14	0.951123539	0.964	0.535	1.10E-226	2.61E-222
**27**	DEK	0.716289879	0.897	0.488	5.54E-221	1.32E-216
**27**	PCNA	0.743863041	0.624	0.197	2.23E-213	5.31E-209
**27**	COL17A1	0.710410187	0.58	0.15	1.88E-205	4.47E-201
**27**	CKS2	0.753482285	0.914	0.46	2.44E-205	5.80E-201
**27**	MT1X	1.063613134	0.967	0.749	5.48E-200	1.30E-195
**27**	SLC25A5	0.719561077	0.979	0.803	9.77E-199	2.33E-194
**27**	TUBA1C	0.732386183	0.941	0.581	1.26E-192	3.00E-188
**27**	MT2A	0.869235235	0.991	0.802	1.88E-191	4.47E-187
**27**	TUBB4B	0.746895617	0.983	0.781	1.56E-174	3.71E-170
**27**	MT1E	0.903087599	0.826	0.466	9.58E-158	2.28E-153

**27**	KRT17	0.674349961	0.563	0.2	4.46E-126	1.06E-121
**28**	KRT17	3.711891716	0.997	0.195	0	0
**28**	S100A2	2.177100707	0.94	0.383	0	0
**28**	KRT6B	2.05260012	0.504	0.052	0	0
**28**	GJB6	1.194929792	0.527	0.033	0	0
**28**	GJB2	0.819943915	0.509	0.066	0	0
**28**	SOSTDC1	0.708111816	0.419	0.043	0	0
**28**	SOX9	0.702099298	0.518	0.064	0	0
**28**	RBP1	0.640828396	0.5	0.075	0	0
**28**	BARX2	0.597521725	0.494	0.06	0	0
**28**	C1QTNF12	0.56631685	0.34	0.021	0	0
**28**	VSNL1	0.414586676	0.398	0.055	0	0
**28**	KRT5	1.457517779	0.991	0.492	3.67E-305	8.74E-301
**28**	CALML3	1.506500959	0.669	0.179	3.39E-281	8.07E-277
**28**	KRT6A	1.465946467	0.534	0.12	1.48E-244	3.51E-240
**28**	S100A14	0.994686434	0.967	0.535	9.65E-221	2.30E-216
**28**	KRT14	0.877226147	0.964	0.672	7.03E-170	1.67E-165
**28**	ABRACL	0.552079498	0.57	0.184	4.39E-164	1.04E-159
**28**	EGR2	0.457148841	0.47	0.127	4.00E-159	9.53E-155
**28**	TRIM29	0.515722556	0.741	0.277	4.83E-154	1.15E-149
**28**	DSG3	0.414504846	0.482	0.135	1.99E-151	4.74E-147
**28**	PHGDH	0.603248237	0.627	0.238	1.15E-143	2.73E-139
**28**	EFNA3	0.411347481	0.549	0.181	3.66E-137	8.70E-133
**28**	CAPNS2	0.446636578	0.496	0.161	1.06E-129	2.52E-125
**28**	SDC1	0.738078767	0.593	0.231	1.73E-123	4.11E-119
**28**	HMGA1	0.518085841	0.696	0.302	2.70E-122	6.42E-118
**28**	PERP	0.579237116	0.999	0.71	1.78E-120	4.23E-116
**28**	LGALS7B	0.839394435	0.882	0.531	8.51E-119	2.03E-114
**28**	SPINT2	0.513950911	0.772	0.394	1.05E-109	2.51E-105
**28**	STMN1	0.584345223	0.66	0.303	4.37E-106	1.04E-101
**28**	KRT16	1.294968658	0.448	0.151	1.86E-104	4.44E-100
**28**	LAMB3	0.427527862	0.337	0.094	2.54E-104	6.04E-100
**28**	DSP	0.467751496	0.87	0.488	3.56E-102	8.48E-98
**28**	ACTN1	0.468227374	0.59	0.27	6.30E-87	1.50E-82
**28**	KRT15	0.86022152	0.481	0.185	7.12E-86	1.69E-81
**28**	DAPL1	0.474657163	0.602	0.271	7.37E-85	1.75E-80
**28**	APOE	0.58786475	0.882	0.661	2.78E-79	6.62E-75
**28**	SERPINB5	0.454900154	0.726	0.383	2.13E-78	5.06E-74
**28**	S100A16	0.564012531	0.819	0.599	1.36E-73	3.24E-69
**28**	TACSTD2	0.575294784	0.831	0.54	5.22E-72	1.24E-67
**28**	FST	0.602899469	0.292	0.096	3.05E-70	7.26E-66
**28**	LGALS7	0.669026109	0.561	0.264	1.35E-69	3.21E-65
**28**	BTG3	0.425568411	0.674	0.435	3.65E-61	8.69E-57
**28**	SFRP1	0.418710034	0.316	0.115	9.64E-59	2.29E-54
**28**	SOX4	0.47279428	0.576	0.404	7.73E-34	1.84E-29
**28**	TMEM45A	0.516976597	0.482	0.298	3.64E-33	8.66E-29
**28**	AREG	0.425813885	0.4	0.214	6.09E-30	1.45E-25
**28**	SPINK5	0.695659648	0.29	0.149	6.39E-28	1.52E-23
**28**	DST	0.471979003	0.49	0.334	3.78E-27	9.00E-23
**28**	GJA1	0.438194746	0.518	0.365	7.45E-24	1.77E-19
**28**	FABP5	0.879677361	0.638	0.537	2.23E-21	5.30E-17
**29**	DES	2.373840879	0.902	0.04	0	0
**29**	ACTG2	2.185801806	0.894	0.054	0	0
**29**	TPM2	2.054769126	0.964	0.224	0	0
**29**	ACTA2	1.946137513	0.953	0.182	0	0
**29**	MYLK	1.9404481	0.842	0.142	0	0
**29**	MYL9	1.836657882	1	0.457	0	0
**29**	TAGLN	1.823354762	0.995	0.292	0	0
**29**	MYH11	1.823284735	0.806	0.078	0	0
**29**	PCP4	1.789577052	0.722	0.017	0	0
**29**	CNN1	1.715094575	0.81	0.057	0	0
**29**	CSRP1	1.577761868	0.837	0.257	0	0
**29**	TPM1	1.455350652	0.912	0.352	0	0
**29**	ACTA1	1.36309124	0.492	0.012	0	0

**29**	PPP1R14A	1.294289395	0.698	0.107	0	0
**29**	ACTC1	0.845420041	0.273	0.013	0	0
**29**	ITGA8	0.773578967	0.382	0.032	0	0
**29**	SYNM	0.766807597	0.373	0.037	0	0
**29**	FLNC	0.741388648	0.304	0.026	0	0
**29**	P2RX1	0.702836397	0.299	0.012	0	0
**29**	SYNPO2	1.203298812	0.564	0.117	2.01E-298	4.77E-294
**29**	CARMN	0.84877877	0.355	0.046	1.49E-289	3.56E-285
**29**	PLN	0.706174657	0.413	0.059	3.07E-280	7.30E-276
**29**	FLNA	1.734881127	0.833	0.36	4.49E-270	1.07E-265
**29**	PDLIM7	1.012647416	0.572	0.152	4.95E-234	1.18E-229
**29**	ACTB	1.090047139	1	0.995	2.72E-231	6.49E-227
**29**	RAMP1	0.752685121	0.404	0.074	1.06E-223	2.53E-219
**29**	SMTN	1.139927393	0.619	0.21	2.00E-206	4.76E-202
**29**	PDLIM3	0.652228385	0.368	0.067	5.03E-200	1.20E-195
**29**	PPP1R12B	0.718392668	0.4	0.089	9.40E-178	2.24E-173
**29**	LMOD1	0.657987263	0.382	0.08	7.54E-176	1.79E-171
**29**	CALD1	0.996218864	0.993	0.578	3.98E-165	9.47E-161
**29**	DSTN	0.85166021	1	0.917	1.09E-151	2.58E-147
**29**	CKB	0.819938771	0.866	0.436	1.13E-151	2.70E-147
**29**	LPP	1.093323174	0.552	0.215	1.44E-144	3.42E-140
**29**	SLMAP	0.810123425	0.446	0.14	3.82E-138	9.09E-134
**29**	CD151	0.723822328	0.825	0.438	5.64E-118	1.34E-113
**29**	MT-ND4	0.992536663	0.995	0.995	1.86E-111	4.43E-107
**29**	MYL6	0.677844285	1	0.993	5.35E-111	1.27E-106
**29**	CFL2	0.632723537	0.353	0.103	6.19E-107	1.47E-102
**29**	FHL1	0.798358298	0.578	0.263	2.87E-106	6.83E-102
**29**	MT-ATP6	0.850009538	0.995	0.993	3.36E-103	7.99E-99
**29**	MT-ND2	0.961137468	0.997	0.995	1.10E-102	2.61E-98
**29**	ITGB1	0.693788044	0.925	0.549	9.44E-98	2.25E-93
**29**	MT-CYB	0.891134662	0.997	0.995	1.01E-95	2.40E-91
**29**	MT-ND3	1.016324688	0.995	0.993	3.16E-95	7.52E-91
**29**	MT-CO2	0.841886069	0.997	0.995	6.58E-82	1.57E-77
**29**	FN1	0.724229209	0.507	0.222	2.62E-81	6.23E-77
**29**	MT-CO3	0.840478305	0.997	0.995	6.48E-79	1.54E-74
**29**	NDUFA4	0.67441362	0.992	0.877	1.20E-74	2.85E-70
**29**	ACTN1	0.757931718	0.49	0.271	1.97E-66	4.70E-62
**30**	FABP4	2.447142919	1	0.132	0	0
**30**	RBP7	1.514226988	0.637	0.087	0	0
**30**	CD36	1.131317586	0.61	0.081	0	0
**30**	CLDN5	0.89669015	0.529	0.087	9.68E-298	2.30E-293
**30**	TMSB4X	0.971097908	1	0.994	8.77E-211	2.09E-206
**30**	IFI27	0.9794039	0.993	0.472	9.38E-210	2.23E-205
**30**	GNG11	1.0810824	0.689	0.264	5.21E-173	1.24E-168
**30**	TMSB10	0.8644489	1	0.973	4.11E-170	9.79E-166
**30**	SPARCL1	0.552812072	0.93	0.346	6.88E-162	1.64E-157
**30**	FABP5	0.701661073	0.988	0.533	6.31E-151	1.50E-146
**30**	EGFL7	0.685706678	0.409	0.101	2.50E-141	5.96E-137
**30**	RAMP2	0.724519939	0.478	0.141	2.12E-127	5.04E-123
**30**	BST2	0.774333366	0.546	0.209	8.59E-117	2.04E-112
**30**	CAV1	0.679021785	0.988	0.656	1.62E-102	3.86E-98
**30**	VWF	0.511729663	0.323	0.08	1.80E-99	4.29E-95
**30**	B2M	0.44654389	1	0.998	6.26E-96	1.49E-91
**30**	PLVAP	0.406955887	0.325	0.082	7.32E-92	1.74E-87
**30**	A2M	0.642510391	0.509	0.196	1.76E-91	4.18E-87
**30**	IGFBP7	0.390588281	1	0.646	7.44E-88	1.77E-83
**30**	IFITM3	0.568383434	1	0.76	1.29E-83	3.07E-79
**30**	PECAM1	0.471660611	0.34	0.097	1.69E-83	4.02E-79
**30**	MYL12A	0.573486616	1	0.837	5.73E-81	1.36E-76
**30**	ADGRL4	0.453086238	0.275	0.074	7.49E-76	1.78E-71
**30**	RAMP3	0.436727572	0.282	0.078	5.21E-74	1.24E-69
**30**	HLA-B	0.486367662	1	0.968	8.67E-74	2.06E-69
**30**	TXNIP	0.455632038	0.921	0.526	1.11E-70	2.64E-66
**30**	CRIP2	0.446039615	0.784	0.428	1.77E-69	4.21E-65

**30**	HLA-E	0.484793373	1	0.882	2.05E-65	4.88E-61
**30**	FKBP1A	0.528163448	0.985	0.687	2.67E-65	6.35E-61
**30**	ID1	0.54354017	0.751	0.404	5.18E-64	1.23E-59
**30**	CCDC85B	0.445079587	0.866	0.518	1.68E-63	4.00E-59
**30**	PCAT19	0.367916514	0.265	0.08	2.06E-58	4.90E-54
**30**	AQP1	0.453749817	0.455	0.2	1.04E-57	2.47E-53
**30**	RHOC	0.433845041	0.881	0.561	1.36E-52	3.24E-48
**30**	IFITM2	0.351225141	0.952	0.558	9.35E-52	2.23E-47
**30**	HLA-C	0.455429073	1	0.961	6.80E-51	1.62E-46
**30**	MYL6	0.377516507	1	0.993	1.67E-47	3.97E-43
**30**	CDC37	0.369024823	0.808	0.519	2.93E-43	6.98E-39
**30**	SLC9A3R2	0.531437482	0.347	0.168	2.73E-41	6.49E-37
**30**	COL4A1	0.479380431	0.323	0.149	8.58E-40	2.04E-35
**30**	ESAM	0.404061398	0.278	0.12	2.18E-36	5.19E-32
**30**	GMFG	0.37864396	0.285	0.133	4.71E-32	1.12E-27
**30**	FIS1	0.314689751	0.773	0.523	1.93E-30	4.59E-26
**30**	SELENOW	0.350973975	0.971	0.74	3.94E-28	9.37E-24
**30**	COL15A1	0.395464143	0.311	0.163	4.40E-27	1.05E-22
**30**	VAMP5	0.465524391	0.405	0.28	1.33E-24	3.17E-20
**30**	ENG	0.339509672	0.282	0.155	1.81E-21	4.32E-17
**30**	COL4A2	0.403388226	0.294	0.168	1.83E-21	4.37E-17
**30**	RGCC	0.345080218	0.373	0.238	2.41E-20	5.75E-16
**30**	MGLL	0.372390125	0.297	0.208	3.52E-13	8.38E-09
**31**	DCT	3.543720778	0.955	0.008	0	0
**31**	TYRP1	3.295146161	0.88	0.009	0	0
**31**	PMEL	2.957021293	0.869	0.015	0	0
**31**	MLANA	2.659096923	0.82	0.008	0	0
**31**	QPCT	1.499653078	0.614	0.114	0	0
**31**	MITF	1.471598202	0.551	0.072	0	0
**31**	CYB561A3	1.278551803	0.598	0.122	0	0
**31**	GPM6B	1.199047798	0.625	0.099	0	0
**31**	MFSD12	1.157183784	0.544	0.052	0	0
**31**	TRPM1	1.13594972	0.535	0.001	0	0
**31**	PCSK2	1.068397165	0.478	0.007	0	0
**31**	BCAN	1.047705878	0.542	0.002	0	0
**31**	PLP1	0.998006921	0.519	0.058	0	0
**31**	TYR	0.997369257	0.504	0.001	0	0
**31**	TUBB2B	0.862300394	0.377	0.043	0	0
**31**	GMPR	0.770958908	0.442	0.033	0	0
**31**	SNCA	0.759719612	0.469	0.062	0	0
**31**	GPR143	0.745809027	0.422	0.002	0	0
**31**	MCOLN3	0.73578809	0.364	0.018	0	0
**31**	KIT	0.721311451	0.409	0.013	0	0
**31**	GPR155	0.772542582	0.425	0.058	1.35E-306	3.21E-302
**31**	EDNRB	0.674292675	0.408	0.072	5.00E-212	1.19E-207
**31**	C4orf48	0.746585472	0.449	0.094	8.74E-196	2.08E-191
**31**	CDC42EP3	0.943950045	0.522	0.132	6.61E-186	1.57E-181
**31**	PHACTR1	0.717738163	0.345	0.063	4.45E-165	1.06E-160
**31**	OSTM1	0.629765514	0.402	0.091	3.18E-159	7.57E-155
**31**	GPNMB	1.013872254	0.961	0.557	3.35E-157	7.98E-153
**31**	SAT1	0.866191185	0.993	0.791	1.12E-144	2.67E-140
**31**	CHCHD6	0.659326355	0.348	0.08	1.49E-133	3.56E-129
**31**	CD59	0.928701363	0.977	0.614	6.15E-132	1.46E-127
**31**	NSG1	0.80057126	0.553	0.205	4.18E-125	9.96E-121
**31**	MT-CO3	0.929320386	0.998	0.995	4.62E-123	1.10E-118
**31**	MT-CYB	0.858148299	1	0.995	1.91E-122	4.54E-118
**31**	APOE	0.78846368	0.982	0.661	4.71E-118	1.12E-113
**31**	HMG20B	0.854602781	0.575	0.256	1.84E-115	4.39E-111
**31**	MT-ND4	0.756030321	1	0.995	6.03E-104	1.43E-99
**31**	RAB32	0.619997711	0.388	0.118	1.23E-101	2.94E-97
**31**	SDCBP	0.684592382	0.93	0.546	3.09E-101	7.35E-97
**31**	FRZB	0.700571798	0.366	0.103	1.38E-98	3.28E-94
**31**	GSTP1	0.948141195	0.993	0.926	1.89E-94	4.50E-90
**31**	TFAP2A	0.65723883	0.472	0.185	7.49E-92	1.78E-87

**31**	MT-ATP6	0.736159467	0.998	0.993	7.98E-92	1.90E-87
**31**	EMP3	0.697872952	0.777	0.412	5.34E-90	1.27E-85
**31**	MT-CO1	0.684340416	1	0.995	2.10E-85	4.99E-81
**31**	MT-ND1	0.658381343	0.998	0.994	1.07E-80	2.55E-76
**31**	MT-ND3	0.825430119	1	0.992	8.62E-75	2.05E-70
**31**	MT-CO2	0.636160001	1	0.995	5.67E-70	1.35E-65
**31**	MT-ND2	0.623181105	1	0.995	3.95E-68	9.40E-64
**31**	ASAH1	0.63699143	0.51	0.291	3.95E-57	9.41E-53
**31**	TTC3	0.646248074	0.451	0.27	6.53E-40	1.56E-35
**32**	GNLY	2.78164674	0.725	0.004	0	0
**32**	NKG7	2.667884612	0.867	0.012	0	0
**32**	XCL1	2.278054512	0.459	0.003	0	0
**32**	XCL2	2.206819527	0.57	0.003	0	0
**32**	CCL5	2.129120846	0.751	0.025	0	0
**32**	GZMB	1.925691361	0.679	0.004	0	0
**32**	CCL4	1.836714415	0.6	0.038	0	0
**32**	SRGN	1.770504441	0.956	0.188	0	0
**32**	KLRB1	1.717719398	0.744	0.016	0	0
**32**	CTSW	1.616881109	0.803	0.014	0	0
**32**	CST7	1.509020107	0.751	0.031	0	0
**32**	CD69	1.351234611	0.613	0.041	0	0
**32**	GZMA	1.306376764	0.585	0.01	0	0
**32**	KLRD1	1.288281262	0.699	0.003	0	0
**32**	CD7	1.264113971	0.662	0.025	0	0
**32**	HCST	1.229832996	0.762	0.079	0	0
**32**	PRF1	1.217326806	0.57	0.003	0	0
**32**	CORO1A	1.20733283	0.681	0.066	0	0
**32**	CXCR4	1.180529478	0.598	0.097	0	0
**32**	CD52	0.988827164	0.487	0.069	0	0
**32**	TYROBP	0.954884495	0.661	0.072	0	0
**32**	FGFBP2	0.938816322	0.393	0.024	0	0
**32**	ITGB2	0.919209266	0.552	0.052	0	0
**32**	DOK2	0.912049021	0.544	0.05	0	0
**32**	KLRC1	0.87439748	0.371	0.001	0	0
**32**	GZMH	0.860527006	0.428	0.003	0	0
**32**	IFNG	0.835273759	0.271	0.007	0	0
**32**	RUNX3	0.83250099	0.518	0.049	0	0
**32**	RAC2	0.80453102	0.544	0.038	0	0
**32**	GPR65	0.79070998	0.482	0.034	0	0
**32**	PTPRC	0.78979645	0.53	0.057	0	0
**32**	FCGR3A	0.788168337	0.328	0.006	0	0
**32**	DUSP2	1.355866927	0.732	0.188	1.56E-284	3.71E-280
**32**	CREM	1.343080336	0.745	0.211	1.54E-260	3.66E-256
**32**	B2M	0.971909131	1	0.998	6.47E-234	1.54E-229
**32**	ISG20	0.788385675	0.485	0.096	5.15E-223	1.22E-218
**32**	ARHGDIB	0.952881641	0.753	0.267	2.23E-199	5.31E-195
**32**	HLA-C	0.868484598	0.998	0.961	3.15E-166	7.49E-162
**32**	HLA-B	0.872563625	1	0.968	1.52E-164	3.61E-160
**32**	HLA-A	0.835177759	1	0.966	8.79E-164	2.09E-159
**32**	IL32	1.009652094	0.526	0.148	1.62E-157	3.86E-153
**32**	PFN1	0.90208893	0.998	0.966	1.77E-156	4.20E-152
**32**	CMC1	1.013545301	0.454	0.122	9.39E-156	2.23E-151
**32**	TMSB4X	0.831137592	1	0.994	1.28E-152	3.05E-148
**32**	CCL3	0.789447456	0.275	0.043	1.92E-145	4.58E-141
**32**	BTG1	1.043186966	0.994	0.904	2.13E-109	5.06E-105
**32**	LITAF	0.791435211	0.638	0.315	1.13E-106	2.68E-102
**32**	REL	1.009960471	0.707	0.435	1.03E-92	2.45E-88
**32**	ID2	0.893564033	0.817	0.601	1.70E-75	4.04E-71
**32**	TNFRSF18	0.822719587	0.365	0.154	2.01E-58	4.79E-54
**33**	MMP7	2.418601245	0.736	0.014	0	0
**33**	CALML5	2.300392977	0.902	0.172	0	0
**33**	KRT6B	2.142065723	0.845	0.05	0	0
**33**	KRT77	1.899764577	0.559	0.015	0	0
**33**	S100P	1.810843326	0.672	0.011	0	0

**33**	CRABP2	1.742712387	0.858	0.274	0	0
**33**	KRT6A	1.611909751	0.803	0.118	0	0
**33**	KRT16	1.535272125	0.71	0.149	0	0
**33**	GJB2	1.357447904	0.572	0.066	0	0
**33**	CLIC3	1.311302769	0.625	0.067	0	0
**33**	WFDC3	1.279203853	0.47	0.007	0	0
**33**	KRT19	1.12782246	0.47	0.041	0	0
**33**	CD24	1.051325281	0.555	0.087	0	0
**33**	GABRP	1.039461652	0.352	0.005	0	0
**33**	HSD11B2	1.037449161	0.439	0.023	0	0
**33**	CNFN	0.982055035	0.446	0.045	0	0
**33**	KRT23	0.972384356	0.496	0.028	0	0
**33**	GJB6	0.949194943	0.445	0.035	0	0
**33**	MIA	0.947258834	0.428	0.015	0	0
**33**	SPTSSB	0.83441039	0.487	0.046	0	0
**33**	SCGB1D2	0.81286839	0.435	0.034	0	0
**33**	FXYD3	1.352657478	0.998	0.486	7.49E-256	1.78E-251
**33**	C19orf33	1.263111888	0.911	0.344	1.07E-250	2.56E-246
**33**	COX6A1	0.969709185	1	0.876	6.27E-172	1.49E-167
**33**	QPCT	0.734840569	0.476	0.116	1.39E-167	3.31E-163
**33**	RHOV	0.824742985	0.596	0.192	2.00E-158	4.75E-154
**33**	COX4I1	0.868214124	1	0.967	2.20E-155	5.24E-151
**33**	CLTB	0.954531088	0.987	0.662	2.18E-152	5.18E-148
**33**	NOV	0.74533392	0.386	0.084	9.77E-152	2.33E-147
**33**	SPINT1	0.991704897	0.528	0.173	6.85E-150	1.63E-145
**33**	COX7A2	0.941593412	0.996	0.863	1.89E-149	4.51E-145
**33**	DEFB1	1.117265432	0.581	0.198	5.54E-149	1.32E-144
**33**	NDUFA4	0.839980003	0.996	0.877	1.51E-145	3.58E-141
**33**	CTSD	0.875261449	0.919	0.499	4.23E-145	1.01E-140
**33**	CD9	0.831408224	1	0.853	6.49E-139	1.54E-134
**33**	COX5B	0.858618274	0.985	0.858	2.61E-136	6.22E-132
**33**	COX6C	0.820989982	0.998	0.923	3.71E-136	8.84E-132
**33**	COX7C	0.770038612	0.998	0.951	2.53E-133	6.03E-129
**33**	COX8A	0.796154339	0.989	0.744	7.07E-131	1.68E-126
**33**	ATP1B1	0.727116955	0.467	0.139	3.65E-122	8.68E-118
**33**	UQCRB	0.80149355	0.985	0.861	1.20E-121	2.85E-117
**33**	LGALS3	0.734729708	1	0.937	2.43E-113	5.79E-109
**33**	NDUFA1	0.722441212	0.959	0.683	6.25E-107	1.49E-102
**33**	COX7B	0.763035454	0.965	0.723	1.80E-104	4.29E-100
**33**	UQCRQ	0.763058289	0.983	0.765	1.16E-100	2.76E-96
**33**	NDUFB2	0.729945167	0.954	0.661	6.39E-100	1.52E-95
**33**	SLC25A5	0.700083808	0.989	0.804	1.71E-99	4.07E-95
**33**	UQCR11	0.733657005	0.982	0.733	4.81E-96	1.14E-91
**33**	CHCHD10	0.706755545	0.574	0.334	2.19E-69	5.20E-65
**33**	CSTA	0.818804092	0.485	0.264	5.12E-47	1.22E-42
**34**	CCL21	3.787632258	0.868	0.021	0	0
**34**	TFF3	2.921617146	0.9	0.005	0	0
**34**	TFPI	1.77641098	0.787	0.168	0	0
**34**	GNG11	1.676360219	0.863	0.263	0	0
**34**	CLDN5	1.666213057	0.796	0.084	0	0
**34**	ANGPT2	1.510682045	0.616	0.102	0	0
**34**	MMRN1	1.454539541	0.653	0.018	0	0
**34**	FABP4	1.316217944	0.839	0.134	0	0
**34**	ECSCR	1.287961032	0.664	0.079	0	0
**34**	RAMP2	1.25391259	0.727	0.139	0	0
**34**	LYVE1	1.244656783	0.529	0.009	0	0
**34**	LAPTM5	1.226876788	0.664	0.104	0	0
**34**	CAVIN2	1.224055039	0.599	0.069	0	0
**34**	PPFIBP1	1.097419642	0.618	0.108	0	0
**34**	EGFL7	1.087899345	0.675	0.098	0	0
**34**	PCAT19	0.84804836	0.577	0.077	0	0
**34**	FXYD6	0.783092084	0.482	0.068	0	0
**34**	KANK3	0.766887161	0.494	0.05	0	0
**34**	PROX1	0.763104455	0.451	0.01	0	0

**34**	PTX3	0.742485843	0.423	0.02	0	0
**34**	PDPN	0.738372116	0.464	0.064	0	0
**34**	TM4SF18	0.703352019	0.484	0.053	0	0
**34**	TIE1	0.70144159	0.475	0.038	0	0
**34**	LMO2	0.925497539	0.464	0.068	1.41E-305	3.36E-301
**34**	SNCG	1.146589384	0.659	0.138	2.45E-296	5.82E-292
**34**	TM4SF1	0.859599852	0.904	0.29	3.97E-248	9.45E-244
**34**	NRP2	0.711756706	0.453	0.082	9.41E-228	2.24E-223
**34**	HYAL2	0.832221662	0.506	0.113	2.41E-205	5.73E-201
**34**	ELK3	0.724899073	0.475	0.105	5.01E-193	1.19E-188
**34**	GYPC	1.048858264	0.677	0.233	2.16E-191	5.15E-187
**34**	IGFBP7	1.194394648	0.998	0.646	6.10E-185	1.45E-180
**34**	TMSB10	0.906636115	1	0.973	1.68E-175	4.00E-171
**34**	S100A10	0.845777213	1	0.896	3.26E-152	7.77E-148
**34**	ARHGAP29	0.719356289	0.56	0.174	9.70E-151	2.31E-146
**34**	ATP5F1E	0.800524636	0.998	0.958	7.59E-146	1.81E-141
**34**	FN1	0.93281648	0.622	0.221	2.36E-136	5.62E-132
**34**	APP	0.7724002	0.866	0.482	9.85E-133	2.34E-128
**34**	FABP5	0.725207636	0.946	0.534	1.63E-130	3.88E-126
**34**	SOX4	1.108268916	0.807	0.402	6.85E-129	1.63E-124
**34**	GNAS	0.839930327	0.937	0.64	7.29E-129	1.74E-124
**34**	CRIP2	0.728643878	0.846	0.427	2.89E-126	6.88E-122
**34**	TIMP3	0.7098244	0.837	0.395	3.61E-121	8.58E-117
**34**	VIM	0.835799029	1	0.711	3.17E-118	7.55E-114
**34**	RAB11A	0.661827357	0.853	0.501	9.39E-117	2.23E-112
**34**	HLA-E	0.722983127	0.996	0.882	4.16E-112	9.91E-108
**34**	LIMS1	0.700431932	0.544	0.22	3.30E-106	7.85E-102
**34**	CD59	0.657360185	0.942	0.614	1.79E-98	4.25E-94
**34**	CD9	0.73173519	0.991	0.853	3.29E-88	7.83E-84
**34**	STMN1	0.669963326	0.605	0.304	4.22E-80	1.00E-75
**34**	EFEMP1	0.840029648	0.508	0.209	1.21E-73	2.89E-69
**35**	GPM6B	2.009057061	0.852	0.097	0	0
**35**	S100B	1.928385347	0.739	0.018	0	0
**35**	PLP1	1.690902968	0.722	0.056	0	0
**35**	CDH19	1.459282141	0.682	0.004	0	0
**35**	SCN7A	1.343229973	0.593	0.008	0	0
**35**	SEMA3B	1.25019197	0.627	0.042	0	0
**35**	MPZ	1.151512118	0.508	0.005	0	0
**35**	NRXN1	1.150922009	0.581	0.001	0	0
**35**	GFRA3	1.142400045	0.504	0.002	0	0
**35**	LGI4	1.10493609	0.562	0.068	0	0
**35**	PCSK2	1.09346059	0.443	0.007	0	0
**35**	NTM	0.785911691	0.384	0.023	0	0
**35**	PMP2	0.777112724	0.324	0.003	0	0
**35**	SOX2	0.731561351	0.381	0.007	0	0
**35**	L1CAM	0.71930895	0.379	0.007	0	0
**35**	PLAT	0.686847306	0.373	0.041	0	0
**35**	COL9A3	0.659826508	0.35	0.026	0	0
**35**	MAL	0.651503841	0.381	0.013	0	0
**35**	VWA1	1.26902954	0.593	0.116	2.36E-304	5.61E-300
**35**	HSPA12A	0.648836485	0.348	0.04	4.41E-285	1.05E-280
**35**	PMP22	1.503825132	0.932	0.375	6.83E-269	1.63E-264
**35**	CADM1	0.810498181	0.434	0.069	3.64E-260	8.66E-256
**35**	CADM3	0.719035805	0.36	0.045	3.21E-259	7.64E-255
**35**	TUBB2B	0.70488406	0.333	0.044	3.22E-230	7.67E-226
**35**	CRYAB	1.781762761	0.951	0.443	9.78E-227	2.33E-222
**35**	MATN2	1.05726672	0.504	0.117	3.41E-206	8.11E-202
**35**	CLU	1.053755305	0.892	0.352	1.60E-184	3.81E-180
**35**	CD9	1.179811225	0.998	0.853	1.31E-181	3.13E-177
**35**	NGFR	0.682540424	0.348	0.059	4.61E-180	1.10E-175
**35**	PRNP	1.135736709	0.93	0.547	8.65E-147	2.06E-142
**35**	FXYD1	1.037195454	0.633	0.245	9.03E-145	2.15E-140
**35**	NDRG2	1.075499701	0.597	0.233	8.53E-140	2.03E-135
**35**	TMEM176B	0.78769266	0.504	0.156	6.42E-127	1.53E-122

**35**	TUBA1A	0.935506928	0.985	0.673	4.26E-115	1.01E-110
**35**	RARRES2	0.727987791	0.542	0.196	5.54E-105	1.32E-100
**35**	CD59	0.726574335	0.958	0.614	2.01E-101	4.78E-97
**35**	S100A10	0.659312797	1	0.896	1.84E-100	4.39E-96
**35**	NRN1	0.725278788	0.462	0.164	2.05E-96	4.89E-92
**35**	CNN3	0.847691092	0.612	0.33	1.46E-87	3.48E-83
**35**	VIM	0.66681138	1	0.711	9.22E-87	2.20E-82
**35**	LTBP4	0.765230649	0.506	0.216	7.71E-86	1.83E-81
**35**	RHOB	0.658495799	0.862	0.519	1.35E-78	3.22E-74
**35**	AP1S2	0.664987873	0.445	0.185	5.35E-75	1.27E-70
**35**	PEBP1	0.660849856	0.992	0.822	3.46E-74	8.24E-70
**35**	CLIC4	0.691633562	0.473	0.216	1.04E-71	2.48E-67
**35**	DKK3	0.804705362	0.439	0.186	1.32E-71	3.14E-67
**35**	PDLIM4	0.658918963	0.46	0.225	8.77E-63	2.09E-58
**35**	S100A4	0.787427809	0.972	0.675	1.43E-59	3.40E-55
**35**	PLPP1	0.661726413	0.413	0.202	1.02E-49	2.43E-45
**35**	FOS	0.647695755	0.985	0.787	1.37E-33	3.25E-29
**36**	COCH	4.587863498	0.98	0.012	0	0
**36**	ASPN	2.093967515	0.919	0.113	0	0
**36**	OGN	1.430011083	0.853	0.07	0	0
**36**	CRABP1	1.413014211	0.777	0.018	0	0
**36**	TNN	1.375056253	0.77	0.013	0	0
**36**	TNMD	1.33576949	0.639	0.032	0	0
**36**	CTSK	1.30938624	0.965	0.212	0	0
**36**	CYP1B1	1.266863792	0.702	0.104	0	0
**36**	FIBIN	1.221337254	0.753	0.057	0	0
**36**	PLXDC1	1.177110388	0.716	0.074	0	0
**36**	FMOD	1.092314116	0.751	0.124	0	0
**36**	PRELP	1.068638136	0.766	0.133	0	0
**36**	PLPP5	1.068631172	0.628	0.075	0	0
**36**	EMID1	0.971339622	0.674	0.05	0	0
**36**	F13A1	0.829924394	0.606	0.056	0	0
**36**	DKK2	0.827060358	0.637	0.071	0	0
**36**	SLITRK6	0.79326617	0.414	0.009	0	0
**36**	LTBP4	1.226312451	0.832	0.214	2.64E-306	6.29E-302
**36**	IGFBP5	1.753557199	0.882	0.237	1.34E-299	3.19E-295
**36**	MMP23B	0.7696756	0.663	0.118	1.31E-287	3.12E-283
**36**	PTGDS	1.282483474	0.923	0.264	1.40E-272	3.34E-268
**36**	TWIST2	1.041087316	0.729	0.152	5.80E-272	1.38E-267
**36**	MEG3	0.910712478	0.794	0.184	5.19E-247	1.23E-242
**36**	FN1	0.938859311	0.829	0.22	1.98E-241	4.70E-237
**36**	DPT	1.200670257	0.748	0.172	1.12E-240	2.67E-236
**36**	HTRA1	1.268294596	0.888	0.276	1.81E-240	4.30E-236
**36**	ANGPTL2	0.98914892	0.685	0.161	5.00E-235	1.19E-230
**36**	MXRA5	0.808333602	0.621	0.133	6.25E-229	1.49E-224
**36**	TCF4	1.171514817	0.875	0.332	1.44E-217	3.43E-213
**36**	MFAP4	1.270502212	0.877	0.276	8.11E-216	1.93E-211
**36**	SPARCL1	1.256182024	0.937	0.347	2.79E-209	6.64E-205
**36**	COL1A2	1.064465458	0.996	0.478	2.52E-204	6.01E-200
**36**	PCOLCE	0.964442223	0.915	0.321	2.48E-198	5.89E-194
**36**	THY1	0.824925623	0.805	0.214	6.43E-198	1.53E-193
**36**	COL1A1	0.853076187	0.991	0.49	5.32E-197	1.27E-192
**36**	ZBTB20	0.902884268	0.814	0.3	6.07E-187	1.45E-182
**36**	PGRMC1	0.977233779	0.775	0.296	2.85E-179	6.78E-175
**36**	TIMP2	0.783409934	0.827	0.287	5.85E-168	1.39E-163
**36**	SOD3	0.956198455	0.93	0.397	7.36E-164	1.75E-159
**36**	SELENOP	0.935136041	0.972	0.634	5.59E-162	1.33E-157
**36**	S100A6	0.914219191	1	0.826	1.15E-157	2.74E-153
**36**	EID1	0.846773542	0.969	0.676	3.26E-157	7.75E-153
**36**	DCN	0.827337175	0.996	0.561	1.83E-154	4.35E-150
**36**	LUM	1.056753279	0.823	0.307	7.58E-154	1.80E-149
**36**	PMP22	0.832707256	0.867	0.376	1.59E-149	3.77E-145
**36**	LGALS1	0.912810977	1	0.672	4.60E-149	1.09E-144
**36**	S100A10	0.905465304	0.998	0.896	4.53E-147	1.08E-142

**36**	CRABP2	0.808207809	0.748	0.276	3.05E-140	7.26E-136
**36**	FBLN1	0.783660549	0.814	0.311	5.63E-136	1.34E-131
**36**	MFGE8	0.817145817	0.735	0.366	5.93E-87	1.41E-82
**37**	DES	4.284391208	1	0.042	0	0
**37**	ACTG2	3.867379452	0.998	0.056	0	0
**37**	TPM2	3.479176231	1	0.226	0	0
**37**	MYLK	3.394203876	1	0.143	0	0
**37**	TAGLN	3.30341389	1	0.294	0	0
**37**	ACTA2	3.14753383	1	0.184	0	0
**37**	PCP4	3.124362747	0.991	0.017	0	0
**37**	MYL9	3.083517273	1	0.458	0	0
**37**	CNN1	3.036054118	1	0.058	0	0
**37**	MYH11	2.955725941	1	0.078	0	0
**37**	TPM1	2.823991083	1	0.353	0	0
**37**	CSRP1	2.696724226	1	0.258	0	0
**37**	ACTA1	2.425390955	0.899	0.01	0	0
**37**	PPP1R14A	2.395470753	0.998	0.106	0	0
**37**	FLNA	2.310710265	0.995	0.36	0	0
**37**	SYNPO2	2.296828007	0.984	0.115	0	0
**37**	LPP	1.984260224	0.982	0.213	0	0
**37**	ACTC1	1.948728633	0.624	0.011	0	0
**37**	SMTN	1.940001005	0.982	0.209	0	0
**37**	PDLIM7	1.86699841	0.995	0.15	0	0
**37**	PLN	1.713894912	0.961	0.056	0	0
**37**	ACTN1	1.68966222	0.982	0.268	0	0
**37**	FHL1	1.662564118	0.982	0.26	0	0
**37**	SYNM	1.647633015	0.904	0.034	0	0
**37**	SLMAP	1.60876428	0.954	0.137	0	0
**37**	RAMP1	1.552676012	0.936	0.071	0	0
**37**	CFL2	1.53186779	0.945	0.099	0	0
**37**	LMOD1	1.47211917	0.954	0.076	0	0
**37**	ITGA8	1.454206015	0.869	0.029	0	0
**37**	PALLD	1.42718913	0.943	0.226	0	0
**37**	NEXN	1.414221586	0.911	0.089	0	0
**37**	ILK	1.387080092	0.952	0.26	0	0
**37**	FLNC	1.377895025	0.849	0.022	0	0
**37**	PPP1R12B	1.373168148	0.936	0.086	0	0
**37**	ALDH1B1	1.37288892	0.826	0.023	0	0
**37**	SCRG1	1.326032728	0.745	0.017	0	0
**37**	SORBS2	1.311658242	0.849	0.086	0	0
**37**	PDLIM3	1.287276682	0.915	0.064	0	0
**37**	SORBS1	1.268101776	0.897	0.045	0	0
**37**	TNS1	1.223795261	0.911	0.137	0	0
**37**	CKB	1.67434781	0.986	0.436	9.98E-306	2.37E-301
**37**	FN1	1.245146484	0.908	0.219	1.05E-303	2.49E-299
**37**	CALD1	1.929357798	0.995	0.579	1.31E-281	3.11E-277
**37**	ACTB	1.724710465	1	0.995	5.66E-270	1.35E-265
**37**	DSTN	1.647221248	1	0.917	3.59E-264	8.56E-260
**37**	MYL6	1.29358052	1	0.993	2.42E-261	5.76E-257
**37**	NDUFA4	1.356294227	1	0.877	6.93E-251	1.65E-246
**37**	CD151	1.295157997	0.966	0.438	1.50E-247	3.58E-243
**37**	ITGB1	1.34635313	0.989	0.55	8.84E-247	2.10E-242
**37**	SELENOM	1.284396491	0.982	0.486	1.79E-219	4.26E-215
**38**	MALAT1	1.75317808	1	0.997	6.10E-217	1.45E-212
**38**	NEAT1	1.61107729	0.995	0.959	4.10E-133	9.76E-129
**38**	LYPD3	0.370556623	0.95	0.456	4.78E-70	1.14E-65
**38**	CSNK1A1	0.58407531	0.913	0.597	2.00E-48	4.75E-44
**38**	CCNL1	0.553732807	0.945	0.638	2.40E-46	5.71E-42
**38**	HNRNPH1	0.543060378	0.926	0.586	4.33E-44	1.03E-39
**38**	XIST	0.794581298	0.685	0.451	3.86E-42	9.19E-38
**38**	CD44	0.699953721	0.975	0.784	5.69E-41	1.35E-36
**38**	SAT1	1.040669043	0.985	0.792	2.31E-40	5.49E-36
**38**	ATP1A1	0.536762799	0.948	0.632	2.28E-37	5.42E-33
**38**	EIF5B	0.580864014	0.811	0.561	1.12E-36	2.67E-32

**38**	CD55	0.443635994	0.797	0.481	3.27E-33	7.77E-29
**38**	INTS6	0.571109989	0.727	0.481	4.11E-33	9.77E-29
**38**	TRA2B	0.493680465	0.734	0.525	4.98E-30	1.18E-25
**38**	REL	0.360602768	0.705	0.436	1.02E-29	2.44E-25
**38**	BAZ1A	0.391433921	0.725	0.456	5.80E-29	1.38E-24
**38**	EMP2	0.366465577	0.888	0.563	7.69E-29	1.83E-24
**38**	HNRNPDL	0.405892881	0.968	0.712	7.06E-28	1.68E-23
**38**	SLC38A2	0.414465993	0.983	0.687	4.97E-27	1.18E-22
**38**	FUS	0.448242606	0.99	0.76	2.82E-26	6.71E-22
**38**	SRSF2	0.593906271	0.99	0.789	4.30E-26	1.02E-21
**38**	SRSF5	0.365831095	0.918	0.64	9.22E-26	2.19E-21
**38**	DNAJB6	0.418766482	0.99	0.725	1.36E-25	3.24E-21
**38**	CLDN4	0.568485908	0.377	0.207	4.07E-23	9.69E-19
**38**	LMNA	0.376738957	0.995	0.961	1.13E-22	2.70E-18
**38**	LUC7L3	0.417416478	0.615	0.451	4.25E-21	1.01E-16
**38**	KLF6	0.363562293	0.973	0.708	7.45E-21	1.77E-16
**38**	TAF1D	0.567456766	0.496	0.386	6.25E-20	1.49E-15
**38**	PNISR	0.40133791	0.648	0.489	6.64E-20	1.58E-15
**38**	AC020916.1	0.678832942	0.375	0.251	8.68E-19	2.07E-14
**38**	HES1	0.518836164	0.963	0.598	1.34E-16	3.18E-12
**38**	LDLR	0.486587773	0.278	0.175	6.59E-14	1.57E-09
**38**	CCNL2	0.38656187	0.268	0.173	2.87E-12	6.83E-08
**38**	EHF	0.436306056	0.28	0.182	7.80E-12	1.86E-07
**38**	SRRM2	0.380764003	0.553	0.469	5.49E-10	1.31E-05
**38**	EPHA2	0.389430908	0.293	0.213	3.94E-09	9.38E-05
**38**	NXF1	0.520932851	0.347	0.296	8.29E-09	0.000197379
**38**	IER2	0.374057387	0.995	0.809	3.53E-08	0.000839224
**38**	THUMPD3-AS1	0.497189123	0.32	0.27	7.51E-08	0.001787626
**38**	IVNS1ABP	0.374774642	0.275	0.208	8.71E-08	0.002072757
**38**	KCNQ1OT1	0.437881491	0.305	0.25	4.45E-07	0.010598143
**38**	MACF1	0.37765524	0.27	0.22	1.48E-06	0.035219248
**38**	JUN	0.374136961	0.998	0.86	1.98E-06	0.047043082
**38**	ABHD5	0.457923509	0.345	0.317	6.91E-06	0.164347258
**38**	DUSP1	0.377707457	0.988	0.841	7.42E-06	0.176535164
**38**	MAP2K3	0.425103833	0.337	0.331	0.000139874	1
**38**	VMP1	0.493668322	0.347	0.348	0.000167731	1
**38**	TLE4	0.419330602	0.29	0.273	0.000230919	1
**38**	ACADVL	0.409044842	0.32	0.342	0.004146335	1
**38**	EFNA1	0.364001799	0.285	0.282	0.007983029	1
**39**	CLDN5	2.630217418	0.991	0.086	0	0
**39**	SLC9A3R2	2.29259568	0.962	0.165	0	0
**39**	RAMP2	1.955831653	0.984	0.14	0	0
**39**	A2M	1.609904264	0.962	0.195	0	0
**39**	PECAM1	1.449323668	0.927	0.095	0	0
**39**	CLEC14A	1.425563384	0.826	0.08	0	0
**39**	ICAM2	1.384880839	0.87	0.067	0	0
**39**	PODXL	1.268184416	0.794	0.036	0	0
**39**	ECSCR	1.224592929	0.848	0.08	0	0
**39**	EGFL7	1.223752058	0.848	0.1	0	0
**39**	ESAM	1.206237454	0.858	0.118	0	0
**39**	SOX17	1.199327851	0.728	0.044	0	0
**39**	RNASE1	1.114342912	0.896	0.104	0	0
**39**	GIMAP7	1.107593264	0.772	0.079	0	0
**39**	SEMA3G	1.096175769	0.747	0.016	0	0
**39**	CDH5	1.058336175	0.775	0.064	0	0
**39**	GJA4	1.018641607	0.671	0.071	0	0
**39**	IGFBP3	1.693770524	0.756	0.117	3.39E-305	8.06E-301
**39**	HYAL2	1.079494498	0.741	0.113	3.54E-298	8.42E-294
**39**	EPAS1	1.38584343	0.915	0.203	4.98E-285	1.18E-280
**39**	DEPP1	1.141236104	0.684	0.096	7.64E-283	1.82E-278
**39**	BST2	1.350738056	0.905	0.209	1.93E-261	4.59E-257
**39**	SRGN	1.080707903	0.953	0.191	2.58E-255	6.14E-251
**39**	TM4SF1	1.78297963	0.991	0.292	2.29E-246	5.45E-242
**39**	KCTD12	1.462421455	0.839	0.193	1.48E-244	3.53E-240

**39**	GNG11	1.573635353	0.962	0.265	1.71E-242	4.07E-238
**39**	BCAM	1.179491202	0.915	0.209	5.53E-241	1.32E-236
**39**	NPDC1	1.043174641	0.826	0.187	3.39E-220	8.06E-216
**39**	IFI27	2.001714003	1	0.475	1.02E-206	2.44E-202
**39**	KLF2	1.19379305	0.839	0.215	5.45E-206	1.30E-201
**39**	SRP14	1.747867135	1	0.95	1.75E-202	4.16E-198
**39**	IFI6	1.237448036	0.842	0.221	7.27E-201	1.73E-196
**39**	PLPP1	1.224578459	0.797	0.201	1.57E-198	3.75E-194
**39**	CRIP2	1.418469967	0.968	0.428	1.04E-194	2.49E-190
**39**	FN1	1.426290706	0.82	0.221	1.37E-173	3.27E-169
**39**	STOM	1.157282516	0.886	0.324	1.19E-171	2.84E-167
**39**	ISG15	1.070035035	0.801	0.213	2.75E-171	6.54E-167
**39**	IFITM1	1.126918909	0.858	0.272	6.14E-167	1.46E-162
**39**	CAV1	1.381706788	0.994	0.657	5.61E-163	1.34E-158
**39**	ADAM15	1.011551149	0.778	0.229	8.28E-162	1.97E-157
**39**	IFITM3	1.243077944	1	0.761	6.97E-149	1.66E-144
**39**	STMN1	1.12990012	0.851	0.304	1.42E-142	3.39E-138
**39**	TMSB10	1.10105891	1	0.973	6.13E-141	1.46E-136
**39**	HLA-E	1.029238396	0.997	0.883	2.01E-136	4.80E-132
**39**	ID1	1.364675409	0.88	0.405	8.73E-136	2.08E-131
**39**	SPARCL1	1.026575227	0.94	0.349	4.58E-133	1.09E-128
**39**	TIMP3	1.019226416	0.877	0.396	4.44E-107	1.06E-102
**39**	TXNIP	1.024922384	0.896	0.528	8.64E-98	2.06E-93
**39**	ADAMTS1	1.009633011	0.696	0.244	5.96E-94	1.42E-89
**39**	SAT1	1.274865615	0.965	0.792	3.10E-71	7.38E-67
**40**	LYZ	1.165548531	0.581	0.05	0	0
**40**	HLA-DPB1	1.56746326	0.898	0.191	1.95E-274	4.65E-270
**40**	HLA-DRA	1.338752443	0.984	0.238	8.08E-257	1.92E-252
**40**	HLA-DPA1	1.268813144	0.812	0.173	6.02E-222	1.43E-217
**40**	HLA-DQA1	0.916345225	0.591	0.098	3.54E-192	8.42E-188
**40**	CD74	1.341749734	1	0.371	3.41E-182	8.11E-178
**40**	LST1	0.914433495	0.415	0.052	2.84E-181	6.75E-177
**40**	FCER1A	0.648176414	0.313	0.034	4.60E-156	1.09E-151
**40**	HLA-DQB1	0.962055929	0.578	0.136	5.24E-129	1.25E-124
**40**	HLA-DRB1	0.905910925	0.703	0.204	3.70E-119	8.80E-115
**40**	TYROBP	0.654495039	0.422	0.076	2.82E-109	6.71E-105
**40**	AIF1	0.77615561	0.355	0.059	5.64E-107	1.34E-102
**40**	TMSB4X	0.9983058	1	0.994	2.57E-103	6.12E-99
**40**	CST3	1.090796716	0.997	0.797	1.62E-94	3.87E-90
**40**	FCER1G	0.592859044	0.326	0.069	4.06E-70	9.65E-66
**40**	TMSB10	0.736686975	1	0.973	6.76E-69	1.61E-64
**40**	ACTB	0.731514155	1	0.995	4.79E-52	1.14E-47
**40**	RPL28	0.463647545	1	0.999	3.42E-48	8.14E-44
**40**	FTH1	0.521719789	1	0.951	4.35E-43	1.04E-38
**40**	FTL	0.571576889	1	0.97	4.43E-38	1.05E-33
**40**	PFN1	0.517411592	1	0.966	1.22E-34	2.91E-30
**40**	SAT1	0.490623522	1	0.792	1.56E-32	3.72E-28
**40**	S100A4	0.355355643	0.994	0.676	8.91E-30	2.12E-25
**40**	NAMPT	0.301592155	0.869	0.465	9.72E-29	2.31E-24
**40**	RPS23	0.330957762	1	0.999	6.94E-28	1.65E-23
**40**	EMP3	0.279098885	0.78	0.413	9.90E-27	2.36E-22
**40**	RPL27A	0.33020365	1	1	1.44E-25	3.42E-21
**40**	TPM3	0.315038582	0.802	0.449	3.97E-25	9.45E-21
**40**	RPS29	0.331619323	1	0.999	9.27E-25	2.21E-20
**40**	ATP5F1E	0.468165735	1	0.958	1.76E-24	4.20E-20
**40**	AP2S1	0.314386203	0.869	0.504	1.08E-23	2.57E-19
**40**	HIGD2A	0.271971046	0.888	0.528	7.32E-21	1.74E-16
**40**	RPS11	0.368279535	1	0.999	1.36E-20	3.24E-16
**40**	RPS20	0.341146563	1	0.999	2.55E-20	6.08E-16
**40**	RPS24	0.268283318	1	0.998	4.53E-20	1.08E-15
**40**	ARPC3	0.416637737	0.971	0.688	8.79E-20	2.09E-15
**40**	HLA-DMA	0.426590773	0.284	0.126	9.44E-20	2.25E-15
**40**	ARPC5	0.260980562	0.805	0.477	2.50E-19	5.95E-15
**40**	RPS13	0.30914329	1	0.999	4.13E-19	9.82E-15

**40**	S100A10	0.322675847	1	0.897	2.36E-18	5.62E-14
**40**	GNG5	0.2773642	0.939	0.591	4.37E-18	1.04E-13
**40**	RPL19	0.268978352	1	1	4.58E-17	1.09E-12
**40**	RPL21	0.280439609	1	1	8.39E-14	2.00E-09
**40**	SH3BGRL3	0.389669653	0.997	0.789	9.95E-14	2.37E-09
**40**	TUBA1B	0.454347333	0.994	0.934	4.89E-13	1.16E-08
**40**	COTL1	0.507549575	0.31	0.186	7.07E-13	1.68E-08
**40**	ARPC2	0.323601965	0.994	0.878	9.58E-13	2.28E-08
**40**	UBA52	0.275134977	1	0.998	4.34E-11	1.03E-06
**40**	OAZ1	0.319397332	1	0.983	3.57E-09	8.49E-05
**40**	YBX1	0.289433119	1	0.982	4.11E-06	0.09785014
**41**	ANGPTL7	1.917961601	0.408	0.004	0	0
**41**	FGFBP2	1.115660692	0.525	0.025	0	0
**41**	ENTPD2	0.577128204	0.317	0.015	0	0
**41**	ABCA8	0.911104946	0.673	0.094	5.92E-253	1.41E-248
**41**	APOD	3.722124619	1	0.368	4.21E-239	1.00E-234
**41**	PODNL1	0.60164266	0.419	0.039	5.04E-238	1.20E-233
**41**	GPC3	1.018382908	0.665	0.095	8.51E-237	2.03E-232
**41**	CYP1B1	1.189987992	0.616	0.106	1.04E-185	2.47E-181
**41**	VIT	0.582299364	0.437	0.055	7.24E-179	1.72E-174
**41**	LTBP4	1.195298821	0.81	0.216	7.56E-179	1.80E-174
**41**	CFH	1.132676845	0.852	0.22	4.77E-159	1.13E-154
**41**	C2orf40	0.94209414	0.433	0.061	3.16E-156	7.53E-152
**41**	LUM	1.33169353	0.933	0.308	9.76E-155	2.32E-150
**41**	PTGDS	1.747022439	0.838	0.267	5.93E-141	1.41E-136
**41**	IGFBP6	1.187525659	0.683	0.161	7.30E-139	1.74E-134
**41**	GSN	1.351350402	1	0.742	4.68E-125	1.11E-120
**41**	PDGFRL	0.61135019	0.553	0.122	4.82E-109	1.15E-104
**41**	DCN	0.992297013	1	0.562	5.90E-109	1.40E-104
**41**	RARRES2	0.679885571	0.718	0.197	6.91E-108	1.64E-103
**41**	MEG3	0.908662777	0.651	0.187	4.49E-94	1.07E-89
**41**	AKAP12	0.574974432	0.5	0.12	1.99E-91	4.73E-87
**41**	IGFBP7	1.017415663	0.996	0.648	1.08E-87	2.57E-83
**41**	NGFR	0.645441191	0.331	0.061	1.34E-84	3.19E-80
**41**	CST3	0.723056448	1	0.797	1.02E-75	2.42E-71
**41**	C1S	0.585409052	0.87	0.352	2.28E-75	5.44E-71
**41**	SERPING1	0.694466116	0.88	0.427	2.20E-73	5.23E-69
**41**	PLAC9	0.751702092	0.817	0.365	6.25E-73	1.49E-68
**41**	SERPINF1	0.840478852	0.835	0.388	1.82E-70	4.34E-66
**41**	TIMP1	0.726265881	0.944	0.588	3.60E-67	8.57E-63
**41**	TCF4	0.667387134	0.75	0.335	8.07E-66	1.92E-61
**41**	LAPTM4A	0.689686867	0.996	0.9	1.64E-64	3.91E-60
**41**	LGALS1	0.638507314	1	0.673	2.09E-63	4.98E-59
**41**	S100A6	0.624184882	1	0.827	1.70E-59	4.06E-55
**41**	DKK3	0.540418177	0.535	0.186	5.68E-59	1.35E-54
**41**	CNN3	0.622056326	0.725	0.331	5.78E-59	1.37E-54
**41**	EID1	0.564777233	0.954	0.677	3.57E-58	8.50E-54
**41**	CD63	0.56531074	0.996	0.849	7.86E-56	1.87E-51
**41**	SPARCL1	0.58577384	0.782	0.35	1.70E-55	4.05E-51
**41**	ZBTB20	0.594133081	0.658	0.303	1.97E-54	4.68E-50
**41**	LHFPL6	0.658908401	0.63	0.266	2.27E-52	5.40E-48
**41**	FN1	0.797374777	0.57	0.223	1.34E-50	3.19E-46
**41**	SPTBN1	0.544386368	0.602	0.278	5.49E-46	1.31E-41
**41**	CEBPD	0.90681611	0.958	0.821	1.37E-34	3.26E-30
**41**	CCL2	0.738869813	0.739	0.506	2.76E-25	6.57E-21
**41**	HES1	0.636188541	0.796	0.6	9.69E-23	2.31E-18
**41**	CYR61	0.567053193	0.722	0.526	1.32E-22	3.14E-18
**41**	TXNIP	0.550874845	0.68	0.529	7.19E-22	1.71E-17
**41**	JUN	0.727094856	0.954	0.861	1.93E-21	4.60E-17
**41**	ID3	0.702068905	0.81	0.666	1.81E-20	4.30E-16
**41**	EGR1	0.614423456	0.715	0.605	3.11E-17	7.39E-13
**42**	IGHM	1.699141327	0.76	0.004	0	0
**42**	CD37	1.594294943	0.909	0.07	0	0
**42**	CD79A	1.348593148	0.635	0.008	0	0

**42**	MS4A1	1.227547232	0.558	0.001	0	0
**42**	LTB	1.209008924	0.731	0.043	0	0
**42**	CCR7	1.152600193	0.519	0.028	0	0
**42**	LY9	0.986334089	0.457	0.01	0	0
**42**	IGKC	0.882239349	0.75	0.052	0	0
**42**	SPIB	0.798779183	0.476	0.008	0	0
**42**	JCHAIN	0.746904806	0.308	0.01	0	0
**42**	RUBCNL	0.659340863	0.423	0.017	0	0
**42**	LAPTM5	1.354754596	0.827	0.107	8.86E-260	2.11E-255
**42**	CD69	1.367685664	0.524	0.045	4.79E-241	1.14E-236
**42**	CD52	1.012808261	0.663	0.07	3.84E-235	9.15E-231
**42**	RHOH	0.713761913	0.471	0.037	1.72E-232	4.09E-228
**42**	CXCR4	1.687804215	0.755	0.1	1.38E-230	3.28E-226
**42**	HLA-DQA1	0.998022068	0.774	0.098	3.63E-228	8.63E-224
**42**	HLA-DQB1	1.105404027	0.88	0.136	9.79E-225	2.33E-220
**42**	CORO1A	0.778892952	0.639	0.07	2.75E-219	6.54E-215
**42**	IRF8	1.007365627	0.519	0.049	3.79E-218	9.02E-214
**42**	CD83	1.335279687	0.784	0.123	8.97E-205	2.13E-200
**42**	HLA-DPB1	1.012057459	0.942	0.192	1.48E-184	3.52E-180
**42**	CD48	0.741005822	0.476	0.05	1.29E-173	3.08E-169
**42**	HLA-DRA	1.37111135	0.981	0.24	1.29E-173	3.08E-169
**42**	HLA-DPA1	0.894848202	0.904	0.174	6.20E-172	1.48E-167
**42**	CYTIP	0.724834944	0.519	0.061	6.04E-162	1.44E-157
**42**	LIMD2	0.649408663	0.476	0.058	6.22E-150	1.48E-145
**42**	GPR183	1.493655834	0.591	0.088	2.95E-143	7.01E-139
**42**	HLA-DRB1	1.048637383	0.885	0.204	4.94E-139	1.18E-134
**42**	CD74	1.589901878	1	0.372	6.35E-136	1.51E-131
**42**	STK4	0.84015579	0.644	0.131	3.29E-119	7.84E-115
**42**	RPS27	0.891519052	1	1	2.71E-102	6.45E-98
**42**	PHACTR1	0.6836596	0.423	0.065	4.14E-101	9.86E-97
**42**	NFKBID	0.857227756	0.562	0.121	7.98E-97	1.90E-92
**42**	RPS29	0.802042497	1	0.999	5.43E-89	1.29E-84
**42**	RPL21	0.712276234	1	1	1.48E-88	3.51E-84
**42**	RPLP2	0.714895566	1	1	2.13E-88	5.07E-84
**42**	CREM	1.122629502	0.688	0.214	1.09E-76	2.59E-72
**42**	RPL23A	0.63209855	1	1	1.84E-74	4.38E-70
**42**	REL	1.042134273	0.856	0.436	8.55E-69	2.04E-64
**42**	RPL39	0.643032453	1	0.999	2.18E-68	5.18E-64
**42**	CYBA	0.922173306	0.774	0.339	5.82E-60	1.39E-55
**42**	EZR	0.952167091	0.909	0.537	3.26E-58	7.77E-54
**42**	BIRC3	0.762097307	0.524	0.173	4.55E-47	1.08E-42
**42**	HERPUD1	1.25535563	0.875	0.676	1.13E-45	2.69E-41
**42**	SRGN	0.713030693	0.562	0.194	9.90E-41	2.36E-36
**42**	HSPE1	0.767171149	0.918	0.793	2.44E-29	5.81E-25
**42**	NR4A2	0.694867368	0.534	0.267	4.10E-24	9.76E-20
**42**	IRF7	0.932144458	0.293	0.123	3.48E-18	8.28E-14
**42**	RGS2	1.160257626	0.332	0.197	1.73E-09	4.12E-05
**43**	CCL19	3.295404518	1	0.054	0	0
**43**	CCL21	2.172874125	0.826	0.027	0	0
**43**	STEAP4	1.063857652	0.669	0.087	3.63E-170	8.63E-166
**43**	LGI4	0.745365022	0.593	0.071	6.51E-158	1.55E-153
**43**	GGT5	1.014590347	0.773	0.132	7.96E-148	1.89E-143
**43**	RERG	0.757752666	0.622	0.085	2.13E-146	5.07E-142
**43**	SYNPO2	0.917036464	0.703	0.12	4.16E-131	9.91E-127
**43**	NR2F2	1.101259692	0.773	0.161	9.69E-124	2.31E-119
**43**	GPX3	1.077021213	0.82	0.189	1.51E-112	3.59E-108
**43**	TINAGL1	1.022110179	0.756	0.158	9.00E-110	2.14E-105
**43**	ACTA2	1.349502306	0.814	0.188	4.05E-106	9.63E-102
**43**	ANGPT2	0.681699547	0.622	0.106	8.57E-105	2.04E-100
**43**	EPS8	0.788314149	0.738	0.165	1.46E-104	3.49E-100
**43**	FGF7	0.927165108	0.698	0.145	1.67E-104	3.98E-100
**43**	TAGLN	1.544495814	0.924	0.298	3.05E-102	7.27E-98
**43**	FILIP1L	0.890587656	0.68	0.159	5.73E-91	1.36E-86
**43**	ISYNA1	0.780725358	0.558	0.104	1.77E-90	4.22E-86

**43**	RARRES2	0.873501917	0.779	0.198	4.47E-88	1.06E-83
**43**	CPE	0.970001477	0.86	0.257	6.46E-86	1.54E-81
**43**	LHFPL6	0.886719458	0.837	0.267	7.18E-83	1.71E-78
**43**	IGFBP5	1.596468375	0.785	0.24	2.37E-81	5.64E-77
**43**	DEPP1	0.823529882	0.512	0.098	1.81E-76	4.31E-72
**43**	CALD1	1.320147685	0.965	0.582	2.73E-75	6.49E-71
**43**	C2orf40	1.094682095	0.39	0.062	5.08E-74	1.21E-69
**43**	PRRX1	0.840273724	0.75	0.222	8.55E-74	2.04E-69
**43**	MYL9	1.035846261	0.948	0.461	2.35E-73	5.60E-69
**43**	IGFBP7	1.37405633	0.994	0.648	9.70E-73	2.31E-68
**43**	TIMP3	1.244190994	0.878	0.398	3.32E-72	7.90E-68
**43**	RGS5	0.852002686	0.5	0.093	6.93E-72	1.65E-67
**43**	TFPI	0.735915244	0.669	0.172	8.47E-72	2.02E-67
**43**	IFITM3	1.060908002	1	0.762	4.02E-70	9.57E-66
**43**	ID4	0.996048038	0.762	0.255	7.11E-69	1.69E-64
**43**	FHL2	0.744500275	0.64	0.18	2.87E-66	6.84E-62
**43**	CTSC	0.895164251	0.721	0.237	3.68E-66	8.75E-62
**43**	IFITM1	0.820206828	0.797	0.274	7.39E-66	1.76E-61
**43**	RGS16	1.545432195	0.797	0.316	1.23E-60	2.92E-56
**43**	C11orf96	0.716383798	0.68	0.196	4.19E-58	9.97E-54
**43**	SELENOM	0.734511573	0.942	0.488	1.09E-51	2.59E-47
**43**	CCL8	0.733911887	0.291	0.047	5.40E-51	1.29E-46
**43**	ADIRF	0.760996151	0.983	0.634	1.09E-50	2.59E-46
**43**	TGFBI	1.054472696	0.721	0.283	4.13E-50	9.83E-46
**43**	SERPING1	0.792192871	0.872	0.428	1.71E-49	4.07E-45
**43**	C1S	0.670600925	0.866	0.353	2.33E-48	5.55E-44
**43**	IFITM2	0.77474756	0.919	0.561	4.32E-46	1.03E-41
**43**	S100A4	0.825083728	0.988	0.677	9.52E-45	2.27E-40
**43**	CCL2	1.095219335	0.89	0.506	2.25E-43	5.35E-39
**43**	TPM1	0.737803269	0.744	0.356	6.38E-40	1.52E-35
**43**	7-Sep	0.721821715	0.866	0.631	3.78E-39	9.00E-35
**43**	NDUFA4L2	0.895656854	0.593	0.242	6.73E-34	1.60E-29
**43**	CYR61	0.745764061	0.744	0.526	1.50E-19	3.58E-15
**44**	IGKC	7.284640366	0.756	0.053	0	0
**44**	IGLC3	7.077897969	0.774	0.025	0	0
**44**	IGLC2	6.67108764	0.72	0.019	0	0
**44**	IGHG1	6.559663056	0.915	0.013	0	0
**44**	IGHG3	6.440959484	0.933	0.019	0	0
**44**	JCHAIN	5.149108722	0.841	0.009	0	0
**44**	IGHA1	4.962746319	0.311	0.009	0	0
**44**	IGHG4	4.480336182	0.86	0.007	0	0
**44**	IGHG2	4.087463095	0.787	0.001	0	0
**44**	IGHM	3.887047528	0.476	0.006	0	0
**44**	MZB1	2.428646243	0.982	0.003	0	0
**44**	CD79A	2.373515533	0.896	0.008	0	0
**44**	DERL3	1.933076481	0.963	0.004	0	0
**44**	IGHGP	1.438219899	0.543	0	0	0
**44**	CD27	1.264651233	0.841	0.01	0	0
**44**	LY9	0.894689039	0.591	0.01	0	0
**44**	SPAG4	0.860294839	0.707	0.003	0	0
**44**	IGLV6-57	0.810662758	0.463	0	0	0
**44**	EVI2B	0.693496626	0.665	0.043	0	0
**44**	CYTIP	0.939156948	0.756	0.061	2.12E-293	5.06E-289
**44**	ANKRD28	1.099514533	0.805	0.108	9.38E-199	2.23E-194
**44**	FKBP11	1.476041105	0.902	0.158	6.18E-198	1.47E-193
**44**	SEC11C	1.316942721	0.89	0.153	1.15E-194	2.73E-190
**44**	PDE4B	0.751843054	0.671	0.072	2.50E-194	5.95E-190
**44**	PIM2	1.058167863	0.726	0.097	6.65E-181	1.58E-176
**44**	CYBA	2.277767658	1	0.339	4.01E-147	9.56E-143
**44**	ISG20	0.795567193	0.689	0.098	1.06E-146	2.52E-142
**44**	SERPINB9	0.760623314	0.628	0.086	1.40E-135	3.33E-131
**44**	AL121944.1	0.873818565	0.598	0.085	1.49E-131	3.55E-127
**44**	SDF2L1	0.909662924	0.805	0.194	7.93E-109	1.89E-104
**44**	SSR4	1.964042413	0.988	0.737	1.35E-108	3.21E-104

**44**	CXCR4	0.958703646	0.634	0.101	5.74E-108	1.37E-103
**44**	HERPUD1	2.306042038	0.976	0.676	2.03E-99	4.83E-95
**44**	TNFRSF18	0.679075642	0.72	0.155	3.49E-95	8.30E-91
**44**	ITM2C	0.868938872	0.695	0.152	5.04E-92	1.20E-87
**44**	DNAJB9	1.011743693	0.841	0.273	4.96E-89	1.18E-84
**44**	DUSP5	1.430905148	0.732	0.2	1.34E-86	3.19E-82
**44**	PRDX4	0.862241227	0.878	0.292	9.12E-86	2.17E-81
**44**	XBP1	1.268521656	0.97	0.504	8.10E-85	1.93E-80
**44**	ERLEC1	0.688920801	0.689	0.178	2.98E-78	7.10E-74
**44**	CYTOR	0.867092306	0.72	0.197	4.38E-72	1.04E-67
**44**	RGS1	0.91110704	0.372	0.056	1.24E-68	2.95E-64
**44**	SPCS2	0.747965999	0.902	0.54	1.30E-57	3.09E-53
**44**	PELI1	0.784713127	0.646	0.2	1.14E-56	2.71E-52
**44**	RGCC	0.902628716	0.72	0.238	2.74E-51	6.51E-47
**44**	BIRC3	0.774443152	0.561	0.173	2.14E-42	5.10E-38
**44**	ERN1	0.938340243	0.378	0.089	8.85E-42	2.11E-37
**44**	TSC22D3	1.017337507	0.768	0.437	2.19E-36	5.21E-32
**44**	SAT1	1.20442031	0.909	0.793	5.84E-26	1.39E-21
**44**	DDIT4	0.885893036	0.707	0.409	2.27E-25	5.40E-21
**45**	HSPA6	2.643212532	1	0.135	3.22E-178	7.66E-174
**45**	KRT15	1.61781985	0.95	0.187	8.08E-97	1.92E-92
**45**	COL17A1	1.032243087	0.861	0.154	1.07E-87	2.55E-83
**45**	SYT8	0.728791038	0.604	0.098	1.94E-66	4.62E-62
**45**	S100A2	1.060597868	1	0.388	1.91E-53	4.55E-49
**45**	HSPA1A	1.482191853	1	0.932	2.12E-53	5.05E-49
**45**	ATF3	1.553422991	1	0.559	3.38E-53	8.04E-49
**45**	HSPA1B	1.447599329	1	0.871	5.78E-52	1.38E-47
**45**	KRT5	1.35479513	1	0.497	9.85E-50	2.34E-45
**45**	KRT14	1.632202323	1	0.675	4.00E-49	9.53E-45
**45**	JUN	1.40587256	1	0.861	1.62E-47	3.87E-43
**45**	DST	1.020424401	0.911	0.335	3.23E-46	7.70E-42
**45**	DNAJB1	1.295068714	1	0.846	6.04E-46	1.44E-41
**45**	HSPB1	1.196986473	1	0.971	1.56E-44	3.70E-40
**45**	HSP90AA1	1.123930174	1	0.971	5.02E-42	1.20E-37
**45**	SOX15	0.7505537	0.851	0.278	1.05E-41	2.50E-37
**45**	UBC	0.972208369	1	0.992	1.30E-41	3.11E-37
**45**	DNAJB4	0.832045963	0.762	0.246	2.41E-40	5.74E-36
**45**	ZFAND2A	0.972081221	0.713	0.227	1.03E-38	2.45E-34
**45**	FOS	1.395012665	0.97	0.788	2.47E-38	5.88E-34
**45**	HSPH1	1.142101975	0.921	0.542	2.71E-37	6.45E-33
**45**	AC020916.1	0.717274029	0.752	0.251	8.24E-37	1.96E-32
**45**	DNAJA1	1.077022414	1	0.83	4.98E-35	1.19E-30
**45**	EGR1	1.202314294	0.911	0.605	1.21E-34	2.87E-30
**45**	TOB1	0.898265555	0.861	0.443	1.66E-33	3.94E-29
**45**	SERPINH1	1.259920241	0.891	0.476	1.93E-33	4.60E-29
**45**	ANKRD37	0.858163527	0.594	0.175	9.00E-33	2.14E-28
**45**	IFRD1	0.80767752	0.842	0.366	7.19E-32	1.71E-27
**45**	FOSB	1.015265117	0.941	0.659	1.71E-31	4.08E-27
**45**	HSPA8	0.926230381	1	0.932	2.34E-30	5.57E-26
**45**	PMAIP1	0.716274505	0.772	0.294	1.30E-29	3.09E-25
**45**	ZFP36	0.916415781	1	0.898	6.94E-29	1.65E-24
**45**	ARL4A	0.801237864	0.733	0.297	5.77E-27	1.37E-22
**45**	RND3	0.818050209	0.792	0.388	1.09E-26	2.59E-22
**45**	TRA2B	0.709565053	0.861	0.526	4.88E-26	1.16E-21
**45**	DUSP1	1.287165511	0.931	0.842	4.09E-25	9.75E-21
**45**	IER2	0.776329399	0.98	0.81	4.68E-25	1.11E-20
**45**	SERTAD1	0.713921611	0.891	0.529	8.35E-25	1.99E-20
**45**	BAG3	0.821662963	0.832	0.47	2.81E-24	6.69E-20
**45**	CYR61	1.322130676	0.832	0.526	2.68E-23	6.38E-19
**45**	NR4A1	0.881685195	0.832	0.494	3.42E-23	8.14E-19
**45**	MYC	1.027966036	0.931	0.704	2.82E-22	6.71E-18
**45**	SLC38A2	0.753974207	0.901	0.689	1.77E-21	4.21E-17
**45**	HES1	1.065892592	0.861	0.6	7.99E-21	1.90E-16
**45**	INTS6	0.705595418	0.802	0.483	7.77E-19	1.85E-14

**45**	BTG2	0.813114775	0.832	0.565	1.02E-18	2.43E-14
**45**	SGK1	0.718607101	0.683	0.361	1.65E-14	3.93E-10
**45**	ANXA1	0.762671019	0.97	0.869	5.50E-13	1.31E-08
**45**	UBB	0.774253393	0.911	0.97	1.61E-11	3.82E-07
**45**	CRYAB	0.750858904	0.653	0.448	1.51E-08	0.00035879
**46**	MPZ	4.226660735	1	0.009	0	0
**46**	S100B	2.39034972	0.941	0.024	0	0
**46**	PMP2	1.748414652	0.662	0.005	0	0
**46**	MIA	1.370275558	0.691	0.018	0	0
**46**	MLIP	1.369169082	0.706	0.006	0	0
**46**	BCAS1	1.292370784	0.676	0.004	0	0
**46**	MT3	1.290437831	0.632	0.002	0	0
**46**	KIF19	1.032377853	0.544	0.005	0	0
**46**	NCMAP	0.978241902	0.588	0.001	0	0
**46**	AATK	0.925451542	0.559	0.005	0	0
**46**	PLEKHB1	0.88330954	0.471	0.009	0	0
**46**	CLDN19	0.829209593	0.485	0.001	0	0
**46**	SOX2	1.204197762	0.471	0.01	5.20E-298	1.24E-293
**46**	PRX	2.156219003	0.897	0.046	8.86E-263	2.11E-258
**46**	MAL	0.920729132	0.515	0.015	1.23E-238	2.93E-234
**46**	FGFBP2	1.240301667	0.676	0.027	2.30E-236	5.48E-232
**46**	PLP1	2.173794942	0.853	0.062	3.17E-178	7.55E-174
**46**	MBP	3.155745765	0.956	0.118	6.96E-129	1.66E-124
**46**	GPM6B	1.640670513	0.912	0.103	2.56E-123	6.10E-119
**46**	SEMA3B	1.091043436	0.632	0.047	1.83E-120	4.36E-116
**46**	LPL	0.865942907	0.5	0.035	1.89E-98	4.50E-94
**46**	GLDN	1.185749909	0.603	0.058	6.21E-90	1.48E-85
**46**	PLLP	1.345460519	0.75	0.101	3.31E-85	7.88E-81
**46**	FXYD6	1.216061688	0.647	0.071	8.61E-85	2.05E-80
**46**	SECISBP2L	1.229609789	0.676	0.083	1.08E-81	2.57E-77
**46**	PLEKHA4	1.507437196	0.706	0.122	3.86E-62	9.18E-58
**46**	PMP22	3.042025242	1	0.379	1.58E-59	3.77E-55
**46**	GATM	1.498594734	0.779	0.16	6.07E-59	1.44E-54
**46**	SVIP	1.170107284	0.721	0.136	5.98E-57	1.42E-52
**46**	CNP	1.252507145	0.735	0.16	4.41E-52	1.05E-47
**46**	CRYAB	2.215443686	1	0.447	1.22E-49	2.91E-45
**46**	ITM2C	1.142733442	0.691	0.153	5.23E-45	1.24E-40
**46**	PLA2G16	1.057896037	0.603	0.121	2.58E-42	6.14E-38
**46**	LIMCH1	0.994794896	0.559	0.103	5.11E-42	1.22E-37
**46**	TSC22D4	1.346582838	0.706	0.193	3.41E-40	8.11E-36
**46**	RHOBTB3	0.878437166	0.559	0.137	2.83E-30	6.74E-26
**46**	PEBP1	0.973403283	1	0.823	3.91E-28	9.30E-24
**46**	CD9	1.291361402	1	0.854	5.62E-28	1.34E-23
**46**	TAX1BP3	1.086394413	0.794	0.363	5.90E-27	1.40E-22
**46**	SPARC	0.868766388	1	0.6	2.03E-25	4.83E-21
**46**	RGCC	0.972020019	0.706	0.239	3.50E-25	8.34E-21
**46**	CALM2	0.88044374	1	0.958	7.42E-24	1.77E-19
**46**	VIM	1.02095201	1	0.714	6.09E-22	1.45E-17
**46**	FTH1	0.837653446	1	0.952	2.29E-21	5.44E-17
**46**	TUBA1A	0.896038179	0.985	0.676	3.33E-21	7.93E-17
**46**	FXYD1	0.82571262	0.662	0.248	3.66E-21	8.71E-17
**46**	SMTN	0.821648311	0.588	0.214	1.08E-20	2.57E-16
**46**	DYNLRB1	0.900111751	0.794	0.485	2.35E-18	5.59E-14
**46**	CUEDC2	1.056013495	0.529	0.203	9.28E-18	2.21E-13
**46**	RCAN1	1.098178081	0.368	0.141	2.10E-09	5.00E-05
**47**	RBP4	2.180682672	0.82	0.009	0	0
**47**	CIDEC	1.986512352	0.738	0.002	0	0
**47**	GPD1	1.867394656	0.689	0.002	0	0
**47**	PLIN1	1.856073022	0.721	0.001	0	0
**47**	PLIN4	1.778787563	0.607	0.004	0	0
**47**	THRSP	1.394872064	0.492	0.001	0	0
**47**	ADIPOQ	1.326813916	0.475	0	0	0
**47**	GPAM	1.284283997	0.492	0.011	5.64E-275	1.34E-270
**47**	PPP1R1A	0.968262833	0.492	0.011	6.22E-273	1.48E-268

**47**	LIPE	1.306692129	0.492	0.014	9.66E-220	2.30E-215
**47**	AQP7	0.893934888	0.361	0.01	3.64E-159	8.66E-155
**47**	SCD	1.853945858	0.459	0.019	2.82E-141	6.70E-137
**47**	G0S2	2.420391726	0.951	0.093	1.14E-130	2.72E-126
**47**	S100B	1.230202633	0.492	0.024	2.36E-123	5.63E-119
**47**	FABP4	2.418266898	1	0.14	1.64E-99	3.91E-95
**47**	DGAT2	1.588544918	0.525	0.036	9.54E-99	2.27E-94
**47**	LPL	1.233904549	0.459	0.035	6.58E-75	1.57E-70
**47**	MGST1	2.142174432	0.787	0.135	3.12E-65	7.42E-61
**47**	PLA2G16	1.585452772	0.705	0.121	1.03E-56	2.46E-52
**47**	CD36	1.518678318	0.623	0.085	1.47E-54	3.50E-50
**47**	ADH1B	1.372138654	0.639	0.121	4.40E-40	1.05E-35
**47**	AKR1C2	1.322127489	0.557	0.111	1.85E-35	4.41E-31
**47**	PNPLA2	1.745818544	0.689	0.204	5.11E-33	1.22E-28
**47**	HSPB6	0.824138151	0.459	0.078	5.31E-32	1.26E-27
**47**	AKR1C1	1.184630778	0.541	0.112	6.69E-32	1.59E-27
**47**	AGPAT2	1.574916737	0.672	0.201	1.29E-31	3.08E-27
**47**	CYB5A	1.311588369	0.934	0.434	2.18E-30	5.20E-26
**47**	CRYAB	1.658248639	0.984	0.447	9.63E-30	2.29E-25
**47**	ADIRF	1.422668262	1	0.635	7.03E-28	1.67E-23
**47**	GPX4	1.340977573	1	0.835	1.01E-26	2.40E-22
**47**	RARRES2	1.328945062	0.656	0.199	2.13E-26	5.07E-22
**47**	FASN	1.268397544	0.361	0.061	9.52E-26	2.27E-21
**47**	LGALS1	1.009280301	1	0.674	5.16E-21	1.23E-16
**47**	ALDH2	1.044710984	0.885	0.425	3.13E-20	7.45E-16
**47**	ACSL1	0.890952984	0.41	0.106	4.72E-18	1.12E-13
**47**	SNCG	0.941542669	0.492	0.143	2.05E-17	4.87E-13
**47**	FTL	0.91599389	1	0.971	5.02E-17	1.20E-12
**47**	ELMOD3	0.817363678	0.279	0.056	1.34E-15	3.18E-11
**47**	SLC25A1	0.766677652	0.393	0.117	7.56E-15	1.80E-10
**47**	FHL1	1.025278319	0.607	0.266	1.21E-14	2.87E-10
**47**	MT-ND4	0.780716374	1	0.995	1.72E-12	4.10E-08
**47**	MT-ND3	0.982621805	1	0.993	3.57E-12	8.50E-08
**47**	MGST3	0.811932156	1	0.756	1.09E-11	2.59E-07
**47**	MT-ND2	0.918399556	1	0.995	6.88E-11	1.64E-06
**47**	MDH1	1.222555986	0.557	0.336	1.40E-10	3.33E-06
**47**	CAV2	0.831440339	0.557	0.327	2.78E-10	6.62E-06
**47**	MT-CYB	0.761478882	1	0.995	2.48E-09	5.90E-05
**47**	AKR1C3	0.78362187	0.361	0.148	3.91E-08	0.000930173
**47**	LDHB	1.046826568	0.492	0.344	4.05E-06	0.09645537
**47**	ASPH	0.830479467	0.377	0.236	3.79E-05	0.900944089
**48**	LOR	7.184271559	1	0.017	0	0
**48**	FLG	5.70314629	1	0.016	0	0
**48**	LCE1B	5.44717131	0.792	0.004	0	0
**48**	LCE2B	5.336523023	0.917	0.002	0	0
**48**	C1orf68	5.200627537	1	0.016	0	0
**48**	SPRR2G	5.126262139	0.792	0.004	0	0
**48**	CRCT1	5.057580629	1	0.003	0	0
**48**	ASPRV1	5.049936943	1	0.007	0	0
**48**	CST6	5.034479043	1	0.013	0	0
**48**	FLG2	4.729901338	1	0.003	0	0
**48**	LCE2C	4.719254981	0.792	0.001	0	0
**48**	SPRR2E	4.718538008	1	0.009	0	0
**48**	LCE1E	4.716047683	0.75	0.002	0	0
**48**	LCE2D	4.42470632	0.833	0.001	0	0
**48**	PSORS1C2	4.091830801	1	0.004	0	0
**48**	LCE1A	4.066620924	0.708	0	0	0
**48**	LCE2A	4.040805821	0.708	0.001	0	0
**48**	KPRP	3.977919637	0.958	0.002	0	0
**48**	IL37	3.96775823	0.958	0.001	0	0
**48**	LCE5A	3.846790972	0.5	0.001	0	0
**48**	LCE6A	3.635404555	0.917	0.001	0	0
**48**	SPRR5	3.136592883	0.833	0.001	0	0
**48**	CDSN	3.125778914	1	0.004	0	0

**48**	LCE3D	2.943941489	0.875	0.001	0	0
**48**	NCCRP1	2.807406945	0.958	0.015	0	0
**48**	WFDC12	2.690727348	0.792	0	0	0
**48**	LINC00302	2.67109308	0.792	0	0	0
**48**	AL365226.2	2.652709324	0.75	0.003	0	0
**48**	PSAPL1	2.429463138	1	0.001	0	0
**48**	BTC	2.422263327	0.958	0.008	0	0
**48**	SPRR2B	2.334859181	0.542	0	0	0
**48**	ARG1	2.310889166	0.833	0.001	0	0
**48**	KLK5	3.440467738	1	0.023	1.01E-227	2.40E-223
**48**	LCE1C	5.888259076	0.833	0.017	1.39E-213	3.30E-209
**48**	FAM25A	4.314492311	1	0.029	2.62E-184	6.24E-180
**48**	C5orf46	4.14798495	1	0.029	1.10E-182	2.61E-178
**48**	AZGP1	4.010573732	1	0.03	2.16E-180	5.13E-176
**48**	CYSRT1	2.363732767	0.958	0.028	8.00E-175	1.90E-170
**48**	KRT23	3.373710833	1	0.032	2.48E-165	5.90E-161
**48**	CTSV	2.521978968	0.917	0.057	2.67E-82	6.35E-78
**48**	CNFN	2.856105506	0.833	0.049	2.89E-77	6.88E-73
**48**	SPINK5	2.337342699	0.958	0.151	1.03E-36	2.44E-32
**48**	GLRX	2.820294499	0.958	0.16	1.61E-36	3.83E-32
**48**	CALML5	3.554873655	0.958	0.178	2.44E-31	5.80E-27
**48**	CTNNBIP1	3.291630802	1	0.281	9.16E-27	2.18E-22
**48**	SBSN	3.649505228	1	0.296	9.50E-26	2.26E-21
**48**	CSTA	2.664230127	0.958	0.265	1.15E-23	2.73E-19
**48**	BNIP3L	2.273515132	0.958	0.288	2.67E-23	6.36E-19
**48**	KRTDAP	2.971909471	1	0.445	8.48E-20	2.02E-15
**48**	HOPX	2.449168803	0.833	0.392	6.59E-11	1.57E-06
