# Supplementary material for: Tofacitinib blocks IFN-regulated biomarker genes in skin fibroblasts and keratinocytes in a systemic sclerosis trial
Source: JCI Insight. 2022 Sep 8;7(17):e159566. doi: 10.1172/jci.insight.159566 (PMC9536259; doi:10.1172/jci.insight.159566)
Supplement: ICMJE disclosure forms [file jciinsight-7-159566-s207.pdf]

ICMJE DISCLOSURE FORM

Date: 2/25/2022

Your Name: Dinesh Khanna

Manuscript Title: **Tofacitinib blocks interferon-regulated biomarker genes in dermal and adventitial skin fibroblasts and keratinocytes in early systemic sclerosis: Results from a Phase I/II randomized controlled trial**

Manuscript Number (if known): **159566-INS-CMED-1**

In the interest of transparency, we ask you to disclose all relationships/activities/interests listed below that are related to the content of your manuscript. "Related" means any relation with for-profit or not-for-profit third parties whose interests may be affected by the content of the manuscript. Disclosure represents a commitment to transparency and does not necessarily indicate a bias. If you are in doubt about whether to list a relationship/activity/interest, it is preferable that you do so.

The author's relationships/activities/interests should be defined broadly. For example, if your manuscript pertains to the epidemiology of hypertension, you should declare all relationships with manufacturers of antihypertensive medication, even if that medication is not mentioned in the manuscript.

In item #1 below, report all support for the work reported in this manuscript without time limit. For all other items, the time frame for disclosure is the past 36 months.

		**Name all entities with whom you have this relationship or indicate none (add rows as needed)**	**Specifications/Comments (e.g., if payments were made to you or to your institution)**
**Time frame: Since the initial planning of the work**			
1	All support for the present manuscript (e.g., funding, provision of study materials, medical writing, article processing charges, etc.) **No time limit for this item.**	Pfizer	
			Click the tab key to add additional rows.
**Time frame: past 36 months**			
**2**	Grants or contracts from any entity (if not indicated in item #1 above).	☒ **None**	
**3**	Royalties or licenses	☒ **None**	

		Name all entities with whom you have this relationship or indicate none (add rows as needed)	Specifications/Comments (e.g., if payments were made to you or to your institution)
**4**	Consulting fees	☐ **None**	
		Acceleron	
		Actelion	
		Amgen	
		Bayer	
		Boehringer Ingelheim	
		Chemomab	
		CSL Behring	
		Genentech/Roche	
		Horizon	
		Paracrine Cell Therapy	
		Mitsubishi Tanabe Pharma	
		Prometheus	
		Theraly	
**5**	Payment or honoraria for lectures, presentations, speakers bureaus, manuscript writing or educational events	☒ **None**	
**6**	Payment for expert testimony	☒ **None**	
**7**	Support for attending meetings and/or travel	☒ **None**	
**8**	Patents planned, issued or pending	☒ **None**	
**9**	Participation on a Data Safety Monitoring Board or Advisory Board	☒ **None**	

		Name all entities with whom you have this relationship or indicate none (add rows as needed)	Specifications/Comments (e.g., if payments were made to you or to your institution)
**10**	Leadership or fiduciary role in other board, society, committee or advocacy group, paid or unpaid	☐ **None**	
		Eicos Sciences, Inc – a subsidiary of CiviBioPharma	Chief Medical Officer
**11**	Stock or stock options	☐ **None**	
		Eicos Science, Inc	Stock options
**12**	Receipt of equipment, materials, drugs, medical writing, gifts or other services	☒ **None**	
**13**	Other financial or non-financial interests	☒ **None**	

Please place an "X" next to the following statement to indicate your agreement:

☒ I certify that I have answered every question and have not altered the wording of any of the questions on this form.

ICMJE DISCLOSURE FORM

**Date:**	2/24/2022
**Your Name:**	David A Fox
**Manuscript Title:**	**Tofacitinib blocks interferon-regulated biomarker genes in dermal and adventitial skin fibroblasts and keratinocytes in early systemic sclerosis: Results from a Phase I/II randomized controlled trial**
**Manuscript Number (if known):**	**159566-INS-CMED-1**

In the interest of transparency, we ask you to disclose all relationships/activities/interests listed below that are related to the content of your manuscript. "Related" means any relation with for-profit or not-for-profit third parties whose interests may be affected by the content of the manuscript. Disclosure represents a commitment to transparency and does not necessarily indicate a bias. If you are in doubt about whether to list a relationship/activity/interest, it is preferable that you do so.

The author's relationships/activities/interests should be defined broadly. For example, if your manuscript pertains to the epidemiology of hypertension, you should declare all relationships with manufacturers of antihypertensive medication, even if that medication is not mentioned in the manuscript.

In item #1 below, report all support for the work reported in this manuscript without time limit. For all other items, the time frame for disclosure is the past 36 months.

		**Name all entities with whom you have this relationship or indicate none (add rows as needed)**	**Specifications/Comments (e.g., if payments were made to you or to your institution)**
**Time frame: Since the initial planning of the work**			
1	All support for the present manuscript (e.g., funding, provision of study materials, medical writing, article processing charges, etc.) **No time limit for this item.**	☐ None	
		Pfizer funded this study through a grant on which I am a co-investigator	University of Michigan
			Click the tab key to add additional rows.
**Time frame: past 36 months**			
**2**	Grants or contracts from any entity (if not indicated in item #1 above).	☐ **None**	
		NIH/NIAID ACE Program - CoPI	University of Michigan
		NIH/NIAID/ITN - Coinvestigator	U Michigan subcontract - Benaroya Institute
		NIH/NIAMS – R56	U Michigan
		Scleroderma Foundation	U Michigan
**3**	Royalties or licenses	☒ **None**	

		Name all entities with whom you have this relationship or indicate none (add rows as needed)	Specifications/Comments (e.g., if payments were made to you or to your institution)
**4**	Consulting fees	☒ **None**	
**5**	Payment or honoraria for lectures, presentations, speakers bureaus, manuscript writing or educational events	☒ **None**	
**6**	Payment for expert testimony	☒ **None**	
**7**	Support for attending meetings and/or travel	☒ **None**	
**8**	Patents planned, issued or pending	☒ **None**	
**9**	Participation on a Data Safety Monitoring Board or Advisory Board	☒ **None**	
**10**	Leadership or fiduciary role in other board, society, committee or advocacy group, paid or unpaid	☒ **None**	

		Name all entities with whom you have this relationship or indicate none (add rows as needed)	Specifications/Comments (e.g., if payments were made to you or to your institution)
**11**	Stock or stock options	☒ **None**	
**12**	Receipt of equipment, materials, drugs, medical writing, gifts or other services	☒ **None**	
**13**	Other financial or non-financial interests	☒ **None**	

Please place an "X" next to the following statement to indicate your agreement:

☒ I certify that I have answered every question and have not altered the wording of any of the questions on this form.

ICMJE DISCLOSURE FORM

**Date:**	2/24/2022
**Your Name:**	Johann E. Gudjonsson
**Manuscript Title:**	**Tofacitinib blocks interferon-regulated biomarker genes in dermal and adventitial skin fibroblasts and keratinocytes in early systemic sclerosis: Results from a Phase I/II randomized controlled trial**
**Manuscript Number (if known):**	**159566-INS-CMED-1**

In the interest of transparency, we ask you to disclose all relationships/activities/interests listed below that are related to the content of your manuscript. "Related" means any relation with for-profit or not-for-profit third parties whose interests may be affected by the content of the manuscript. Disclosure represents a commitment to transparency and does not necessarily indicate a bias. If you are in doubt about whether to list a relationship/activity/interest, it is preferable that you do so.

The author's relationships/activities/interests should be defined broadly. For example, if your manuscript pertains to the epidemiology of hypertension, you should declare all relationships with manufacturers of antihypertensive medication, even if that medication is not mentioned in the manuscript.

In item #1 below, report all support for the work reported in this manuscript without time limit. For all other items, the time frame for disclosure is the past 36 months.

		Name all entities with whom you have this relationship or indicate none (add rows as needed)	Specifications/Comments (e.g., if payments were made to you or to your institution)
Time frame: Since the initial planning of the work			
1	All support for the present manuscript (e.g., funding, provision of study materials, medical writing, article processing charges, etc.) **No time limit for this item.**	☒ None	
			Click the tab key to add additional rows.
Time frame: past 36 months			
**2**	Grants or contracts from any entity (if not indicated in item #1 above).	☒ **None**	
**3**	Royalties or licenses	☒ **None**	

		Name all entities with whom you have this relationship or indicate none (add rows as needed)	Specifications/Comments (e.g., if payments were made to you or to your institution)
**4**	Consulting fees	☐ **None**	
		Almirall	Advisory Board / Research grant
		Novartis	Advisory Board
		Janssen	Advisory Board / Research grant
		Boehringer Ingelheim	Advisory Board
**5**	Payment or honoraria for lectures, presentations, speakers bureaus, manuscript writing or educational events	☐ **None**	
		Boehringer Ingeleheim	
**6**	Payment for expert testimony	☒ **None**	
**7**	Support for attending meetings and/or travel	☒ **None**	
**8**	Patents planned, issued or pending	☒ **None**	
**9**	Participation on a Data Safety Monitoring Board or Advisory Board	☒ **None**	
**10**	Leadership or fiduciary role in other board, society, committee or advocacy group, paid or unpaid	☐ **None**	
		International Psoriasis Council	
		Editorial Board of Journal of Investigative Dermatology, Editoral Board of Journal of Allergy and Clinical Immunology, Editorial Board of JCI Insight.	

		Name all entities with whom you have this relationship or indicate none (add rows as needed)	Specifications/Comments (e.g., if payments were made to you or to your institution)
**11**	Stock or stock options	☒ **None**	
**12**	Receipt of equipment, materials, drugs, medical writing, gifts or other services	☒ **None**	
**13**	Other financial or non-financial interests	☒ **None**	

Please place an "X" next to the following statement to indicate your agreement:

☒ I certify that I have answered every question and have not altered the wording of any of the questions on this form.

ICMJE DISCLOSURE FORM

Date: 2/24/2022

Your Name: Suiyuan Huang

Manuscript Title: **Tofacitinib blocks interferon-regulated biomarker genes in dermal and adventitial skin fibroblasts and keratinocytes in early systemic sclerosis: Results from a Phase I/II randomized controlled trial**

Manuscript Number (if known): **159566-INS-CMED-1**

In the interest of transparency, we ask you to disclose all relationships/activities/interests listed below that are related to the content of your manuscript. "Related" means any relation with for-profit or not-for-profit third parties whose interests may be affected by the content of the manuscript. Disclosure represents a commitment to transparency and does not necessarily indicate a bias. If you are in doubt about whether to list a relationship/activity/interest, it is preferable that you do so.

The author's relationships/activities/interests should be defined broadly. For example, if your manuscript pertains to the epidemiology of hypertension, you should declare all relationships with manufacturers of antihypertensive medication, even if that medication is not mentioned in the manuscript.

In item #1 below, report all support for the work reported in this manuscript without time limit. For all other items, the time frame for disclosure is the past 36 months.

		Name all entities with whom you have this relationship or indicate none (add rows as needed)	Specifications/Comments (e.g., if payments were made to you or to your institution)
**Time frame: Since the initial planning of the work**			
1	All support for the present manuscript (e.g., funding, provision of study materials, medical writing, article processing charges, etc.) **No time limit for this item.**	☒ None	
			Click the tab key to add additional rows.
**Time frame: past 36 months**			
**2**	Grants or contracts from any entity (if not indicated in item #1 above).	☒ **None**	
**3**	Royalties or licenses	☒ **None**	

		Name all entities with whom you have this relationship or indicate none (add rows as needed)	Specifications/Comments (e.g., if payments were made to you or to your institution)
**4**	Consulting fees	☒ **None**	
**5**	Payment or honoraria for lectures, presentations, speakers bureaus, manuscript writing or educational events	☒ **None**	
**6**	Payment for expert testimony	☒ **None**	
**7**	Support for attending meetings and/or travel	☒ **None**	
**8**	Patents planned, issued or pending	☒ **None**	
**9**	Participation on a Data Safety Monitoring Board or Advisory Board	☒ **None**	
**10**	Leadership or fiduciary role in other board, society, committee or advocacy group, paid or unpaid	☒ **None**	

		Name all entities with whom you have this relationship or indicate none (add rows as needed)	Specifications/Comments (e.g., if payments were made to you or to your institution)
**11**	Stock or stock options	☒ **None**	
**12**	Receipt of equipment, materials, drugs, medical writing, gifts or other services	☒ **None**	
**13**	Other financial or non-financial interests	☒ **None**	

Please place an "X" next to the following statement to indicate your agreement:

☒ I certify that I have answered every question and have not altered the wording of any of the questions on this form.

ICMJE DISCLOSURE FORM

Date: 2/24/2022

Your Name: J. Michelle Kahlenberg

Manuscript Title: **Tofacitinib blocks interferon-regulated biomarker genes in dermal and adventitial skin fibroblasts and keratinocytes in early systemic sclerosis: Results from a Phase I/II randomized controlled trial**

Manuscript Number (if known): **159566-INS-CMED-1**

In the interest of transparency, we ask you to disclose all relationships/activities/interests listed below that are related to the content of your manuscript. "Related" means any relation with for-profit or not-for-profit third parties whose interests may be affected by the content of the manuscript. Disclosure represents a commitment to transparency and does not necessarily indicate a bias. If you are in doubt about whether to list a relationship/activity/interest, it is preferable that you do so.

The author's relationships/activities/interests should be defined broadly. For example, if your manuscript pertains to the epidemiology of hypertension, you should declare all relationships with manufacturers of antihypertensive medication, even if that medication is not mentioned in the manuscript.

In item #1 below, report all support for the work reported in this manuscript without time limit. For all other items, the time frame for disclosure is the past 36 months.

		**Name all entities with whom you have this relationship or indicate none (add rows as needed)**	**Specifications/Comments (e.g., if payments were made to you or to your institution)**
Time frame: Since the initial planning of the work			
1	All support for the present manuscript (e.g., funding, provision of study materials, medical writing, article processing charges, etc.) **No time limit for this item.**	☒ None	
			Click the tab key to add additional rows.
Time frame: past 36 months			
**2**	Grants or contracts from any entity (if not indicated in item #1 above).	☐ **None**	
		Q32 Bio	institution
		Janssen	institution
		Bristol Myers Squibb	institution
		Ventus Therapeutics	institution
**3**	Royalties or licenses	☒ **None**	

		Name all entities with whom you have this relationship or indicate none (add rows as needed)	Specifications/Comments (e.g., if payments were made to you or to your institution)
**4**	Consulting fees	☐ **None**	
		Glaxo Smith Kline	self
		AstraZeneca	Self
		Lupus Therapeutics	Self
		Aurinia Pharmaceuticals	self
		Eli Lilly	Self
		Bristol Myers Squibb	Self
		Vera Therapeutics	Self
		Gilead	self
**5**	Payment or honoraria for lectures, presentations, speakers bureaus, manuscript writing or educational events	☒ **None**	
**6**	Payment for expert testimony	☒ **None**	
**7**	Support for attending meetings and/or travel	☒ **None**	
**8**	Patents planned, issued or pending	☐ **None**	
		USPTO 62/342,690	No payments received
		USPTO 62/613,104	No payments received
**9**	Participation on a Data Safety Monitoring Board or Advisory Board	☒ **None**	
**10**	Leadership or fiduciary role in other board, society, committee or	☐ **None**	
		Rheumatology Research Foundation Scientific Advisory Board	No payments received

		Name all entities with whom you have this relationship or indicate none (add rows as needed)	Specifications/Comments (e.g., if payments were made to you or to your institution)
	advocacy group, paid or unpaid	Arthritis National Research Foundation Scientific Advisory Board	Non payments received
		Associate editor for Arthritis and Rheumatology	Payments to self
**11**	Stock or stock options	☒ **None**	
**12**	Receipt of equipment, materials, drugs, medical writing, gifts or other services	☒ **None**	
**13**	Other financial or non-financial interests	☒ **None**	

Please place an "X" next to the following statement to indicate your agreement:

☒ I certify that I have answered every question and have not altered the wording of any of the questions on this form.

ICMJE DISCLOSURE FORM

Date:____February 22, 2022____
Your Name:____Robert Lafyatis_______________

Manuscript Title:__ Tofacitinib blocks interferon-regulated biomarker genes in dermal and adventitial skin fibroblasts and keratinocytes in early systemic sclerosis: Results from a Phase I/II randomized controlled trial

Manuscript number (if known):__ 159566-INS-CMED-1

In the interest of transparency, we ask you to disclose all relationships/activities/interests listed below that are related to the content of your manuscript. "Related" means any relation with for-profit or not-for-profit third parties whose interests may be affected by the content of the manuscript. Disclosure represents a commitment to transparency and does not necessarily indicate a bias. If you are in doubt about whether to list a relationship/activity/interest, it is preferable that you do so.

The following questions apply to the author's relationships/activities/interests as they relate to the current manuscript only.

The author's relationships/activities/interests should be defined broadly. For example, if your manuscript pertains to the epidemiology of hypertension, you should declare all relationships with manufacturers of antihypertensive medication, even if that medication is not mentioned in the manuscript.

In item #1 below, report all support for the work reported in this manuscript without time limit. For all other items, the time frame for disclosure is the past 36 months.

		**Name all entities with whom you have this relationship or indicate none (add rows as needed)**	**Specifications/Comments (e.g., if payments were made to you or to your institution)**
		**Time frame: Since the initial planning of the work**	
1	All support for the present manuscript (e.g., funding, provision of study materials, medical writing, article processing charges, etc.) **No time limit for this item.**	National Institute of Arthritis and Musculoskeletal and Skin Diseases: 2P50 AR060780	
		**Time frame: past 36 months**	

2	Grants or contracts from any entity (if not indicated in item #1 above).	Corbus, Formation, Moderna, Regeneron, Astra Zeneca, Pfizer	
3	Royalties or licenses	____None	
4	Consulting fees	Pfizer, Bristol Myers Squibb, Boehringer-Ingleheim, Formation, Sanofi, Boehringer-Mannheim, Merck and Genentech/Roche, Biogen	
5	Payment or honoraria for lectures, presentations, speakers bureaus, manuscript writing or educational events	Cleveland Clinic	
6	Payment for expert testimony	____None	
7	Support for attending meetings and/or travel	____None	
8	Patents planned, issued or pending	____None	
9	Participation on a Data Safety Monitoring Board or Advisory Board	____None	
10	Leadership or fiduciary role in other board, society, committee or advocacy group, paid or unpaid	____None	
11	Stock or stock options	____None	
12	Receipt of equipment, materials, drugs, medical writing, gifts or other services	____None	
13	Other financial or non-financial interests	____None	

Please place an "X" next to the following statement to indicate your agreement:

__X_ I certify that I have answered every question and have not altered the wording of any of the questions on this form.

ICMJE DISCLOSURE FORM

Date: 2/24/2022

Your Name: Vivek Nagaraja

Manuscript Title: **Tofacitinib blocks interferon-regulated biomarker genes in dermal and adventitial skin fibroblasts and keratinocytes in early systemic sclerosis: Results from a Phase I/II randomized controlled trial**

Manuscript Number (if known): **159566-INS-CMED-1**

In the interest of transparency, we ask you to disclose all relationships/activities/interests listed below that are related to the content of your manuscript. "Related" means any relation with for-profit or not-for-profit third parties whose interests may be affected by the content of the manuscript. Disclosure represents a commitment to transparency and does not necessarily indicate a bias. If you are in doubt about whether to list a relationship/activity/interest, it is preferable that you do so.

The author's relationships/activities/interests should be defined broadly. For example, if your manuscript pertains to the epidemiology of hypertension, you should declare all relationships with manufacturers of antihypertensive medication, even if that medication is not mentioned in the manuscript.

In item #1 below, report all support for the work reported in this manuscript without time limit. For all other items, the time frame for disclosure is the past 36 months.

		**Name all entities with whom you have this relationship or indicate none (add rows as needed)**	**Specifications/Comments (e.g., if payments were made to you or to your institution)**
**Time frame: Since the initial planning of the work**			
1	All support for the present manuscript (e.g., funding, provision of study materials, medical writing, article processing charges, etc.) **No time limit for this item.**	☒ None	
			Click the tab key to add additional rows.
**Time frame: past 36 months**			
**2**	Grants or contracts from any entity (if not indicated in item #1 above).	☒ **None**	
**3**	Royalties or licenses	☒ **None**	

		Name all entities with whom you have this relationship or indicate none (add rows as needed)	Specifications/Comments (e.g., if payments were made to you or to your institution)
**4**	Consulting fees	☒ **None**	
**5**	Payment or honoraria for lectures, presentations, speakers bureaus, manuscript writing or educational events	☒ **None**	
**6**	Payment for expert testimony	☒ **None**	
**7**	Support for attending meetings and/or travel	☒ **None**	
**8**	Patents planned, issued or pending	☒ **None**	
**9**	Participation on a Data Safety Monitoring Board or Advisory Board	☒ **None**	
**10**	Leadership or fiduciary role in other board, society, committee or advocacy group, paid or unpaid	☒ **None**	

		Name all entities with whom you have this relationship or indicate none (add rows as needed)	Specifications/Comments (e.g., if payments were made to you or to your institution)
**11**	Stock or stock options	☒ **None**	
**12**	Receipt of equipment, materials, drugs, medical writing, gifts or other services	☒ **None**	
**13**	Other financial or non-financial interests	☒ **None**	

Please place an "X" next to the following statement to indicate your agreement:

☒ I certify that I have answered every question and have not altered the wording of any of the questions on this form.

ICMJE DISCLOSURE FORM

Date: 2/25/2022

Your Name: Puja P. Khanna

Manuscript Title: **Tofacitinib blocks interferon-regulated biomarker genes in dermal and adventitial skin fibroblasts and keratinocytes in early systemic sclerosis: Results from a Phase I/II randomized controlled trial**

Manuscript Number (if known): **159566-INS-CMED-1**

In the interest of transparency, we ask you to disclose all relationships/activities/interests listed below that are related to the content of your manuscript. "Related" means any relation with for-profit or not-for-profit third parties whose interests may be affected by the content of the manuscript. Disclosure represents a commitment to transparency and does not necessarily indicate a bias. If you are in doubt about whether to list a relationship/activity/interest, it is preferable that you do so.

The author's relationships/activities/interests should be defined broadly. For example, if your manuscript pertains to the epidemiology of hypertension, you should declare all relationships with manufacturers of antihypertensive medication, even if that medication is not mentioned in the manuscript.

In item #1 below, report all support for the work reported in this manuscript without time limit. For all other items, the time frame for disclosure is the past 36 months.

		**Name all entities with whom you have this relationship or indicate none (add rows as needed)**	**Specifications/Comments (e.g., if payments were made to you or to your institution)**
Time frame: Since the initial planning of the work			
1	All support for the present manuscript (e.g., funding, provision of study materials, medical writing, article processing charges, etc.) **No time limit for this item.**	☒ None	
			Click the tab key to add additional rows.
Time frame: past 36 months			
**2**	Grants or contracts from any entity (if not indicated in item #1 above).	☒ **None**	
**3**	Royalties or licenses	☒ **None**	

		Name all entities with whom you have this relationship or indicate none (add rows as needed)	Specifications/Comments (e.g., if payments were made to you or to your institution)
**4**	Consulting fees	☐ **None**	
		Horizon	
**5**	Payment or honoraria for lectures, presentations, speakers bureaus, manuscript writing or educational events	☒ **None**	
**6**	Payment for expert testimony	☒ **None**	
**7**	Support for attending meetings and/or travel	☒ **None**	
**8**	Patents planned, issued or pending	☒ **None**	
**9**	Participation on a Data Safety Monitoring Board or Advisory Board	☒ **None**	
**10**	Leadership or fiduciary role in other board, society, committee or advocacy group, paid or unpaid	☒ **None**	

		Name all entities with whom you have this relationship or indicate none (add rows as needed)	Specifications/Comments (e.g., if payments were made to you or to your institution)
**11**	Stock or stock options	☒ **None**	
**12**	Receipt of equipment, materials, drugs, medical writing, gifts or other services	☒ **None**	
**13**	Other financial or non-financial interests	☒ **None**	

Please place an "X" next to the following statement to indicate your agreement:

☒ I certify that I have answered every question and have not altered the wording of any of the questions on this form.

ICMJE DISCLOSURE FORM

**Date:**	2/25/2022
**Your Name:**	Cristina Padilla, MD
**Manuscript Title:**	**Tofacitinib blocks interferon-regulated biomarker genes in dermal and adventitial skin fibroblasts and keratinocytes in early systemic sclerosis: Results from a Phase I/II randomized controlled trial**
**Manuscript Number (if known):**	**159566-INS-CMED-1**

In the interest of transparency, we ask you to disclose all relationships/activities/interests listed below that are related to the content of your manuscript. "Related" means any relation with for-profit or not-for-profit third parties whose interests may be affected by the content of the manuscript. Disclosure represents a commitment to transparency and does not necessarily indicate a bias. If you are in doubt about whether to list a relationship/activity/interest, it is preferable that you do so.

The author's relationships/activities/interests should be defined broadly. For example, if your manuscript pertains to the epidemiology of hypertension, you should declare all relationships with manufacturers of antihypertensive medication, even if that medication is not mentioned in the manuscript.

In item #1 below, report all support for the work reported in this manuscript without time limit. For all other items, the time frame for disclosure is the past 36 months.

		**Name all entities with whom you have this relationship or indicate none (add rows as needed)**	**Specifications/Comments (e.g., if payments were made to you or to your institution)**
Time frame: Since the initial planning of the work			
1	All support for the present manuscript (e.g., funding, provision of study materials, medical writing, article processing charges, etc.) **No time limit for this item.**	☒ None	
			Click the tab key to add additional rows.
Time frame: past 36 months			
**2**	Grants or contracts from any entity (if not indicated in item #1 above).	☒ **None**	
**3**	Royalties or licenses	☒ **None**	

		Name all entities with whom you have this relationship or indicate none (add rows as needed)	Specifications/Comments (e.g., if payments were made to you or to your institution)
**4**	Consulting fees	☒ **None**	
**5**	Payment or honoraria for lectures, presentations, speakers bureaus, manuscript writing or educational events	☒ **None**	
**6**	Payment for expert testimony	☒ **None**	
**7**	Support for attending meetings and/or travel	☒ **None**	
**8**	Patents planned, issued or pending	☒ **None**	
**9**	Participation on a Data Safety Monitoring Board or Advisory Board	☒ **None**	
**10**	Leadership or fiduciary role in other board, society, committee or advocacy group, paid or unpaid	☒ **None**	

		Name all entities with whom you have this relationship or indicate none (add rows as needed)	Specifications/Comments (e.g., if payments were made to you or to your institution)
**11**	Stock or stock options	☒ **None**	
**12**	Receipt of equipment, materials, drugs, medical writing, gifts or other services	☒ **None**	
**13**	Other financial or non-financial interests	☒ **None**	

Please place an "X" next to the following statement to indicate your agreement:

☒ I certify that I have answered every question and have not altered the wording of any of the questions on this form.

ICMJE DISCLOSURE FORM

**Date:**	2/24/2022
**Your Name:**	Tracy Tabib
**Manuscript Title:**	**Tofacitinib blocks interferon-regulated biomarker genes in dermal and adventitial skin fibroblasts and keratinocytes in early systemic sclerosis: Results from a Phase I/II randomized controlled trial**
**Manuscript Number (if known):**	**159566-INS-CMED-1**

In the interest of transparency, we ask you to disclose all relationships/activities/interests listed below that are related to the content of your manuscript. "Related" means any relation with for-profit or not-for-profit third parties whose interests may be affected by the content of the manuscript. Disclosure represents a commitment to transparency and does not necessarily indicate a bias. If you are in doubt about whether to list a relationship/activity/interest, it is preferable that you do so.

The author's relationships/activities/interests should be defined broadly. For example, if your manuscript pertains to the epidemiology of hypertension, you should declare all relationships with manufacturers of antihypertensive medication, even if that medication is not mentioned in the manuscript.

In item #1 below, report all support for the work reported in this manuscript without time limit. For all other items, the time frame for disclosure is the past 36 months.

		**Name all entities with whom you have this relationship or indicate none (add rows as needed)**	**Specifications/Comments (e.g., if payments were made to you or to your institution)**
Time frame: Since the initial planning of the work			
1	All support for the present manuscript (e.g., funding, provision of study materials, medical writing, article processing charges, etc.) **No time limit for this item.**	☒ None	
			Click the tab key to add additional rows.
Time frame: past 36 months			
**2**	Grants or contracts from any entity (if not indicated in item #1 above).	☒ **None**	
**3**	Royalties or licenses	☒ **None**	

		Name all entities with whom you have this relationship or indicate none (add rows as needed)	Specifications/Comments (e.g., if payments were made to you or to your institution)
**4**	Consulting fees	☒ **None**	
**5**	Payment or honoraria for lectures, presentations, speakers bureaus, manuscript writing or educational events	☒ **None**	
**6**	Payment for expert testimony	☒ **None**	
**7**	Support for attending meetings and/or travel	☒ **None**	
**8**	Patents planned, issued or pending	☒ **None**	
**9**	Participation on a Data Safety Monitoring Board or Advisory Board	☒ **None**	
**10**	Leadership or fiduciary role in other board, society, committee or advocacy group, paid or unpaid	☒ **None**	

		Name all entities with whom you have this relationship or indicate none (add rows as needed)	Specifications/Comments (e.g., if payments were made to you or to your institution)
**11**	Stock or stock options	☒ **None**	
**12**	Receipt of equipment, materials, drugs, medical writing, gifts or other services	☒ **None**	
**13**	Other financial or non-financial interests	☒ **None**	

Please place an "X" next to the following statement to indicate your agreement:

☒ I certify that I have answered every question and have not altered the wording of any of the questions on this form.

ICMJE DISCLOSURE FORM

**Date:**	2/24/2022
**Your Name:**	Lam C Tsoi
**Manuscript Title:**	**Tofacitinib blocks interferon-regulated biomarker genes in dermal and adventitial skin fibroblasts and keratinocytes in early systemic sclerosis: Results from a Phase I/II randomized controlled trial**
**Manuscript Number (if known):**	**159566-INS-CMED-1**

In the interest of transparency, we ask you to disclose all relationships/activities/interests listed below that are related to the content of your manuscript. "Related" means any relation with for-profit or not-for-profit third parties whose interests may be affected by the content of the manuscript. Disclosure represents a commitment to transparency and does not necessarily indicate a bias. If you are in doubt about whether to list a relationship/activity/interest, it is preferable that you do so.

The author's relationships/activities/interests should be defined broadly. For example, if your manuscript pertains to the epidemiology of hypertension, you should declare all relationships with manufacturers of antihypertensive medication, even if that medication is not mentioned in the manuscript.

In item #1 below, report all support for the work reported in this manuscript without time limit. For all other items, the time frame for disclosure is the past 36 months.

		Name all entities with whom you have this relationship or indicate none (add rows as needed)	Specifications/Comments (e.g., if payments were made to you or to your institution)
**Time frame: Since the initial planning of the work**			
1	All support for the present manuscript (e.g., funding, provision of study materials, medical writing, article processing charges, etc.) **No time limit for this item.**	☐ None	
		**NIH-NIAMS P30 AR075043**	
		**NIH/NIAMS K01AR072129**	
			Click the tab key to add additional rows.
**Time frame: past 36 months**			
**2**	Grants or contracts from any entity (if not indicated in item #1 above).	☐ **None**	
		**Michigan Medicine-Peking University Health Science Center Discovery award**	
**3**	Royalties or licenses	☒ **None**	

		Name all entities with whom you have this relationship or indicate none (add rows as needed)	Specifications/Comments (e.g., if payments were made to you or to your institution)
**4**	Consulting fees	☒ **None**	
**5**	Payment or honoraria for lectures, presentations, speakers bureaus, manuscript writing or educational events	☒ **None**	
**6**	Payment for expert testimony	☒ **None**	
**7**	Support for attending meetings and/or travel	☒ **None**	
**8**	Patents planned, issued or pending	☒ **None**	
**9**	Participation on a Data Safety Monitoring Board or Advisory Board	☒ **None**	
**10**	Leadership or fiduciary role in other board, society, committee or advocacy group, paid or unpaid	☒ **None**	

		Name all entities with whom you have this relationship or indicate none (add rows as needed)	Specifications/Comments (e.g., if payments were made to you or to your institution)
**11**	Stock or stock options	☒ **None**	
**12**	Receipt of equipment, materials, drugs, medical writing, gifts or other services	☒ **None**	
**13**	Other financial or non-financial interests	☐ **None**	
		LCT has received research support from **Janssen**	

Please place an "X" next to the following statement to indicate your agreement:

☒ I certify that I have answered every question and have not altered the wording of any of the questions on this form.

ICMJE DISCLOSURE FORM

Date: 2/24/2022

Your Name: Amber Young

Manuscript Title: **Tofacitinib blocks interferon-regulated biomarker genes in dermal and adventitial skin fibroblasts and keratinocytes in early systemic sclerosis: Results from a Phase I/II randomized controlled trial**

Manuscript Number (if known): **159566-INS-CMED-1**

In the interest of transparency, we ask you to disclose all relationships/activities/interests listed below that are related to the content of your manuscript. "Related" means any relation with for-profit or not-for-profit third parties whose interests may be affected by the content of the manuscript. Disclosure represents a commitment to transparency and does not necessarily indicate a bias. If you are in doubt about whether to list a relationship/activity/interest, it is preferable that you do so.

The author's relationships/activities/interests should be defined broadly. For example, if your manuscript pertains to the epidemiology of hypertension, you should declare all relationships with manufacturers of antihypertensive medication, even if that medication is not mentioned in the manuscript.

In item #1 below, report all support for the work reported in this manuscript without time limit. For all other items, the time frame for disclosure is the past 36 months.

		Name all entities with whom you have this relationship or indicate none (add rows as needed)	Specifications/Comments (e.g., if payments were made to you or to your institution)
**Time frame: Since the initial planning of the work**			
1	All support for the present manuscript (e.g., funding, provision of study materials, medical writing, article processing charges, etc.) **No time limit for this item.**	☒ None	
			Click the tab key to add additional rows.
**Time frame: past 36 months**			
**2**	Grants or contracts from any entity (if not indicated in item #1 above).	☒ **None**	
**3**	Royalties or licenses	☒ **None**	

		Name all entities with whom you have this relationship or indicate none (add rows as needed)	Specifications/Comments (e.g., if payments were made to you or to your institution)
**4**	Consulting fees	☒ **None**	
**5**	Payment or honoraria for lectures, presentations, speakers bureaus, manuscript writing or educational events	☒ **None**	
**6**	Payment for expert testimony	☒ **None**	
**7**	Support for attending meetings and/or travel	☒ **None**	
**8**	Patents planned, issued or pending	☒ **None**	
**9**	Participation on a Data Safety Monitoring Board or Advisory Board	☒ **None**	
**10**	Leadership or fiduciary role in other board, society, committee or advocacy group, paid or unpaid	☒ **None**	

		Name all entities with whom you have this relationship or indicate none (add rows as needed)	Specifications/Comments (e.g., if payments were made to you or to your institution)
**11**	Stock or stock options	☒ **None**	
**12**	Receipt of equipment, materials, drugs, medical writing, gifts or other services	☒ **None**	
**13**	Other financial or non-financial interests	☒ **None**	

Please place an "X" next to the following statement to indicate your agreement:

☒ I certify that I have answered every question and have not altered the wording of any of the questions on this form.

CONSORT 2010 checklist of information to include when reporting a randomised trial*

Section/Topic	Item No	Checklist item	Reported on page No
**Title and abstract**			
	1a	Identification as a randomised trial in the title	1
	1b	Structured summary of trial design, methods, results, and conclusions (for specific guidance see CONSORT for abstracts)	3
**Introduction**			
Background and objectives	2a	Scientific background and explanation of rationale	4-5
	2b	Specific objectives or hypotheses	5
**Methods**			
Trial design	3a	Description of trial design (such as parallel, factorial) including allocation ratio	5-6
	3b	Important changes to methods after trial commencement (such as eligibility criteria), with reasons	N/A
Participants	4a	Eligibility criteria for participants	6
	4b	Settings and locations where the data were collected	
Interventions	5	The interventions for each group with sufficient details to allow replication, including how and when they were actually administered	6, Supp pg 61
Outcomes	6a	Completely defined pre-specified primary and secondary outcome measures, including how and when they were assessed	7
	6b	Any changes to trial outcomes after the trial commenced, with reasons	N/A
Sample size	7a	How sample size was determined	
	7b	When applicable, explanation of any interim analyses and stopping guidelines	N/A
Randomisation:			
Sequence generation	8a	Method used to generate the random allocation sequence	6-7
	8b	Type of randomisation; details of any restriction (such as blocking and block size)	6-7
Allocation concealment mechanism	9	Mechanism used to implement the random allocation sequence (such as sequentially numbered containers), describing any steps taken to conceal the sequence until interventions were assigned	6-7
Implementation	10	Who generated the random allocation sequence, who enrolled participants, and who assigned participants to interventions	7
Blinding	11a	If done, who was blinded after assignment to interventions (for example, participants, care providers, those	

		assessing outcomes) and how	7
	11b	If relevant, description of the similarity of interventions	N/A
Statistical methods	12a	Statistical methods used to compare groups for primary and secondary outcomes	7, supp pg 62
	12b	Methods for additional analyses, such as subgroup analyses and adjusted analyses	N/A
**Results**			
Participant flow (a diagram is strongly recommended)	13a	For each group, the numbers of participants who were randomly assigned, received intended treatment, and were analysed for the primary outcome	8
	13b	For each group, losses and exclusions after randomisation, together with reasons	8
Recruitment	14a	Dates defining the periods of recruitment and follow-up	8
	14b	Why the trial ended or was stopped	
Baseline data	15	A table showing baseline demographic and clinical characteristics for each group	Supp Table 1
Numbers analysed	16	For each group, number of participants (denominator) included in each analysis and whether the analysis was by original assigned groups	8-9
Outcomes and estimation	17a	For each primary and secondary outcome, results for each group, and the estimated effect size and its precision (such as 95% confidence interval)	8-9
	17b	For binary outcomes, presentation of both absolute and relative effect sizes is recommended	N/A
Ancillary analyses	18	Results of any other analyses performed, including subgroup analyses and adjusted analyses, distinguishing pre-specified from exploratory	N/A
Harms	19	All important harms or unintended effects in each group (for specific guidance see CONSORT for harms)	8-9
**Discussion**			
Limitations	20	Trial limitations, addressing sources of potential bias, imprecision, and, if relevant, multiplicity of analyses	
Generalisability	21	Generalisability (external validity, applicability) of the trial findings	18-19
Interpretation	22	Interpretation consistent with results, balancing benefits and harms, and considering other relevant evidence	15-19
**Other information**			
Registration	23	Registration number and name of trial registry	6
Protocol	24	Where the full trial protocol can be accessed, if available	6
Funding	25	Sources of funding and other support (such as supply of drugs), role of funders	2

*We strongly recommend reading this statement in conjunction with the CONSORT 2010 Explanation and Elaboration for important clarifications on all the items. If relevant, we also recommend reading CONSORT extensions for cluster randomised trials, non-inferiority and equivalence trials, non-pharmacological treatments, herbal interventions, and pragmatic trials. Additional extensions are forthcoming: for those and for up to date references relevant to this checklist, see www.consort-statement.org.

EVALUATION OF TOFACITINIB IN EARLY DIFFUSE CUTANEOUS SYSTEMIC SCLEROSIS: A PHASE I/II TWO-CENTER SAFETY AND TOLERABILITY STUDY

Principal Investigator:
Dinesh Khanna, MD, MS
Professor of Medicine
Director, Scleroderma Program
University of Michigan Rheumatology Division
300 N. Ingalls building, Suite 7C27
Ann Arbor, MI 48109-5422
p.734-763-3182
f.734-763-5761
khannad@med.umich.edu

Protocol Version 4.0
2 July 2018

Table of Contents

1 BACKGROUND AND RATIONALE ... 6
1.1 Indication ... 6
1.2 Background ... 6
1.2.1 Tofactinib ... 6
1.2.2 Exploratory Mechanistic Endpoints ... 7
1.2.3 Role of T cells in the Pathogenesis of SSc... 7
1.2.4 Effects of JAK inhibition on T Cells and Fibroblasts ... 9
1.2.5 Clinical Safety of Tofacitinib ... 10
1.3 Overall Risk/Benefit Assessment ... 13
1.4 Study Rationale ... 13
1.5 Summary of Rationale for Proposed Study ... 13
1.6 Dose Rationale ... 13
2 STUDY OUTCOMES ... 13
2.1 Primary Outcomes ... 13
2.2 Secondary Outcomes ... 13
2.3 Exploratory Outcomes ... 14
3 REGULATORY OBLIGATIONS ... 14
3.1 Good Clinical Practice (GCP)... 14
3.2 Institutional Review Board (IRB) ... 15
3.3 Informed Consent ... 15
3.4 Confidentiality ... 15
4 INVESTIGATIONAL PLAN ... 15
4.1 Study Design and Duration ... 15
4.2 Study Schema ... 16
4.3 Study Population... 16
4.4 Inclusion Criteria ... 16
4.5 Exclusion Criteria ... 16
4.6 Reproductive Status ... 19
4.7 Other considerations for potential subjects ... 20
4.7.1 Vaccine and Exposure to Infection Guidelines ... 20
4.7.2 Elective Surgery... 21
4.8 Discontinuation of subjects from Treatment ... 21
5 STUDY TREATMENT ... 22
5.1 Treatment ... 22
5.1.1 Identification ... 22
5.2 Method of Assigning Subjects to Treatment... 23
5.3 Blinding and Unblinding ... 22
5.4 Concomitant Medications... 23
5.5 Prohibited Medications... 23
5.5.1 Other Restrictions & Precautions ... 23
6 STUDY ASSESSMENTS AND PROCEDURES ... 24

6.1 Schedule of Events ... 27
7 ADVERSE EVENT REPORTING ... 30
7.1 Adverse Events ... 30
7.2 Serious Adverse Events ... 30
7.3 Non-Serious Adverse Events ... 31
7.4 Assignment of Adverse Events ... 31
7.5 Adverse Events – Collection and Reporting ... 31
7.5.1 Adverse Events of Special Interest ... 32
7.6 Serious Adverse Event-Collection and Reporting ... 32
7.7 Non-Serious Adverse Event Collection and Reporting ... 33
7.8 Laboratory Monitoring ... 33
8 STATISTICAL CONSIDERATIONS ... 33
8.1 Sample Size Considerations ... 34
8.2 Statistical Analyses ... 34
8.2.1 Safety Outcomes ... 34
8.2.2 Efficacy (Activity) and Exploratory Outcomes ... 34
9 STUDY MANAGEMENT ... 35
9.1 Compliance with protocol ... 35
9.2 Record Retention ... 35
9.3 Study Drug Record ... 36
9.4 Study Drug Destruction ... 36
9.5 Study Monitoring ... 36
10 RESEARCH OF HUMAN SPECIMENS, SAMPLES OR DATA ... 37
10.1 Use of Stored Samples and Data ... 37
10.2 Disposition of Stored Samples and Data ... 37
11 REFERENCES ... 37

Study Synopsis

**Protocol Title:**	Evaluation of tofacitinib in early diffuse cutaneous systemic sclerosis (dcSSc): A phase I/II two center safety and tolerability study
**Clinical Phase:**	Phase I/II
**Sponsor:**	This is a Clinical and Research Collaborative study and Dinesh Khanna, MD, MS will be the sponsor. Pfizer will be supplying the drug/placebo and study funding (including drug distribution at 2 sites).
**Accrual Objective:**	15 subjects (10 active, 5 placebo)
**Accrual Rate:**	Enroll over 12 month time period
**Study Duration:**	Up to 65-day screening period, 24 weeks of double blind follow up, followed by 24 weeks of open label, with a 30 day follow up telephone call after permanent discontinuation of medication.
**Research Centers:**	2: University of Michigan and University of Pittsburgh
**Research Hypothesis:**	Tofacitinib is safe and is well-tolerated in patients with early dcSSc
**Study Schema:**	5 mg BID of tofacitinib vs placebo for 24 weeks with an open label extension receiving tofacitnib 5mg, BID for 24 weeks.
**Primary Outcome:**	
**Primary Outcome Measure:**	The primary endpoint will be the proportion of participants who experience Grade 3 or higher adverse events that occur at or before Week 24.
**Secondary Outcomes:**	• To assess the efficacy of tofacitinib in improving skin thickness in early dcSSc<br>• To assess the efficacy of tofacitinib in improving skin and peripheral whole blood biomarkers in early dcSSc<br>• To correlate skin and peripheral whole blood biomarkers with clinical efficacy assessments
**Secondary Outcome Measures:**	• Number of Grade 3 or higher adverse events that occur at or before Week 12<br>• Number of Grade 3 (severe) or higher adverse events that occur at or before weeks: 12, 36, and 48<br>• Number of Grade 2 (moderate) or higher adverse events that occur at or before weeks: 12, 24, 36, and 48<br>• Number of AE's of special interest (AESI) at weeks: 12, 24, 36, and 48<br>• Change in modified Rodnan Skin Score (mRSS) at weeks: 12, 24, 36, and 48<br>• Provisional American College of Rheumatology Combined Response Index in Systemic Sclerosis at Weeks 12 , 24, and 48.

**Exploratory Outcomes:**	Additionally, to assess the efficacy of treatment using 5mg of tofacitinib versus placebo. These endpoints will include any change in the following scores at the following time points highlighted below:<br><br>• Proportion of subjects with mRSS improvement of 20%, 40%, and 60% at weeks: 12 and 24<br>• Comparison of average, most representative, and maximum skin score methodology for mRSS.<br>• Physician's global assessment on a Likert scale at weeks: 12, 24, 36 and 48<br>• Patient's global assessment on a Likert scale at weeks: 12, 24, 36 and 48<br>• Health-related quality of life (HRQOL) using PROMIS-29 2.0 at weeks: 12, 24, 36 and 48<br>• Physical function as assessed by the scleroderma health assessment questionnaire-disability index (SHAQ-DI) at weeks: 12, 24, 36 and 48<br>• Gastrointestinal symptoms as assessed by UCLA SCTC GIT 2.0 at weeks: 12, 24, 36 and 48<br>• Scleroderma-related skin symptoms assessed by PRO-SRSS at weeks: 12, 24, 36 and 48<br>• Percent predicted FVC at weeks: 12, 24, and 48<br>• Change in left ventricular ejection fraction at week 24<br>• Change in tricuspid regurgitation jet at week 24
**Correlative Studies:**	Skin and biomarkers
**Study Design**	This is a phase I/II placebo controlled study to evaluate the safety, and tolerability of tofacitinib, along with any possible efficacy descriptors, as a treatment for dcSSc. The study will enroll 15 Subjects from 18 to 70 [inclusive] years of age with dcSSc with disease duration of $\leq$ 60 months [from 1st non-Raynaud's phenomenon signs or symptom]. Subjects will be randomized to tofacitinib vs placebo in a 2:1 ratio at 5 mg twice a day for 24 weeks. Subjects will also be offered to participate in an open label phase during which they will receive tofacitinib 5 mg twice a day for 24 weeks. This study will be conducted over a period of approximately 2 years. Two sites are expected to participate in this study.

1. BACKGROUND AND RATIONALE

The safety and effectiveness of tofacitinib for the treatment of rheumatoid arthritis (RA) has been demonstrated in adult subjects. The Sponsor is conducting an investigational program to determine the safety, tolerability, and any possible descriptive indicators of efficacy using tofacitinib in subjects ≥18 and ≤70 years of age with early diffuse cutaneous systemic sclerosis (dcSSc).

1.1 Indication

Tofacitinib was approved on 06 November 2012 in the United States at a dose of 5 mg twice daily (BID) for the treatment of adults with moderately to severely active RA who have had an inadequate response or intolerance to MTX (Methotrexate). As of 05 November 2015, tofacitinib 5 mg BID is approved as 2nd line therapy for the treatment of adults with moderate to severe RA in 50 countries and marketed in 43 countries worldwide including the United States, Canada, Switzerland, Australia and Japan. Tofacitinib 10 mg twice daily (BID) is also approved for the treatment of RA in 3 countries (Switzerland, Russia, and Botswana). In this protocol, we are using the FDA approved dosing of 5 mg BID. It may be used in combination with methotrexate or other non-biologic disease modifying anti-rheumatic drugs (DMARDs).

1.2 Background

1.2.1 Tofactinib

Tofacitinib is a potent selective inhibitor of the Janus Kinase (JAK) family of kinases with a high degree of selectivity against other kinases in the human genome. In kinase assays, tofacitinib inhibits JAK1, JAK2, JAK3 and, to a lesser, extent Tyrosine Kinase 2 (TyK2). In cellular settings where JAK kinases signal in pairs, tofacitinib preferentially inhibits signaling by heterodimeric receptors associated with JAK3 and/or JAK1 with functional selectivity over receptors that signal via pairs of JAK2. Inhibition of JAK1 and JAK3 by tofacitinib blocks signaling through the common gamma chain containing receptors for several cytokines, including interleukins (IL) IL-2, -4,-7,-9, -15 and -21. These cytokines are integral to lymphocyte activation, proliferation and function, and inhibition of their signaling may thus result in modulation of multiple aspects of the immune response. In addition, inhibition of JAK1 will result in attenuation of signaling by additional pro-inflammatory cytokines, such as IL-6 and interferon (IFN)γ. At higher exposures inhibition of erythropoietin signaling could occur via inhibition of JAK2 signaling.

The safety and effectiveness of tofacitinib for the treatment of RA has been demonstrated in adult subjects. Both 5 mg BID and 10 mg BID dose regimens have been studied in adults and demonstrated efficacy. A greater number of adverse events were reported at the 10 mg BID dose level. The sponsor is conducting a safety phase 1/II trial for subjects aged 18 - 70 for treatment of dcSSc. Tyrosine kinase inhibitors have been proposed as logical targets for novel treatment strategies in SSc[1], but not much attention has yet been given to the possible use of JAK inhibitors in SSc. However, the various considerations noted above regarding the pathogenesis of SSc and the target effects of tofacitinib provide a compelling case for *in vitro*, animal model and human

clinical testing of this agent in SSc. The ability of tofacitinib to suppress both Th2 differentiation and the pro-fibrotic effects of Th2 cytokines, while sparing Treg function is a combination of effects that may make this drug especially suitable for SSc

1.2.2 Exploratory Mechanistic Endpoints

In the human CD4+ T cells, inhibiting JAK1 expression completely suppressed IL-6–mediated STAT1 phosphorylation[2] and showed that tofacitinib inhibits IL-6 production and blocks IL 6 signaling. This is supported by a recent phase 2 randomized controlled trial of the interleukin-6 receptor-α inhibitor, tocilizumab patients with SSc.[3] This global, double-blind, placebo-controlled study enrolled adult patients with progressive SSc of ≤5 years' duration from first non-Raynaud sign or symptom. Patients were randomly assigned (1:1) to weekly subcutaneous tocilizumab 162 mg or placebo for 48 weeks. The primary efficacy endpoint was the difference in mean change from baseline in modified Rodnan skin score (mRSS) at week 24. 87 patients received tocilizumab (n=43) or placebo (n=44). The primary endpoint showed a treatment difference of –2·70 mRSS units (95% CI: –5·85, 0·45) in favor of tocilizumab at week 24 but did not meet statistical significance (p=0·0915). At week 48, the treatment difference was –3·55 mRSS units (95% CI: −7·23, 0·12), favoring tocilizumab over placebo (p=0·0579). Exploratory analysis of lung function showed that fewer patients in the tocilizumab arm had a decline in percent predicted forced vital capacity than in the placebo arm by comparison of the cumulative distribution (week 48, p=0·0373). Tocilizumab downregulated the expression of myeloid-associated genes in the skin and decreased circulating levels of CCL18, a chemokine associated with fibrosis and progression of SSc-associated lung disease. Rates of adverse events/serious adverse events were not different between tocilizumab (42/43 [97·7%]/14/43 [32·6%]) and placebo (40/44 [90·9%]/15/44 [34·1%]).

1.2.3 Role of T Cells in the Pathogenesis of SSc

CD4+ T cells are central to the pathogenesis of a range of autoimmune diseases, both through their role in activating B cell differentiation and autoantibody production, and through secretion of cytokines. T cell subsets can also protect against or attenuate autoimmunity through a variety of regulatory mechanisms. The CD4+ T cell population contains distinct subsets classified according to their program of cytokine secretion, such as the Th1, Th2 and Th17 cells, regulatory T cells (Tregs), follicular-helper T cells, and additional subsets including bi-functional subsets that overlap between more than one Th population. Activation of all subsets of CD4+ T cells requires delivery of at least 2 signals to the T cell, one through recognition of antigen-MHC and the second through co-stimulation, primarily by binding of the CD28 ligands B7.1 and B7.2 (CD80 and CD86) to CD28 on the T cell membrane. Differentiation into T effector subsets is controlled by cytokines secreted by antigen-presenting cells, such as IL-12, IL-23, IL-6 TGF-beta and others, most of which signal through JAK-STAT pathways[4].

Substantial evidence supports the concept that T cells play a key role in the pathogenesis of SSc, including cutaneous disease and at least some of the visceral complications. Skin biopsies obtained from SSc patients early in their disease

demonstrate a perivascular, mononuclear cell infiltrate comprised of T cells and macrophages[5-7]. T cells are the dominant population of lymphocytes in the skin, and are activated[6]. T cell infiltration correlate with the skin thickening, suggesting a relation between inflammation and fibrosis[8]. The expression of inducible costimulator (ICOS), expressed on activated T cells, is elevated in patients with early dcSSc[9]. T cells transferred from bleomycin-treated mice (a mouse model of SSc) to healthy animals induces skin thickening[10]. Also, the T lymphocytes in SSc tissue overexpress TNF receptor II and that these cells, when costimulated with TNF-α, trigger collagen production by releasing profibrotic cytokines[11].

Controversy and ambiguity exists, however, regarding which Th subset may be pathogenic in SSc, with the focus largely on the Th2 and Th17 cells[12, 13]. Th2 cells, defined by their production of IL-4, have been implicated in SSc because some of their cytokine products, such as IL-13, are pro-fibrotic[14-16]. IL-4 itself also upregulates expression type I collagen mRNA[17], as well as mRNA for the enzyme lysyl hydroxylase 2, which contributes to collagen cross-linking[18]. Moreover, Th2 cells are found in excess in the blood of patients with SSc, and in both cutaneous and pulmonary disease. Th2 cells in bronchoalveolar lavage fluid in SSc associated interstitial lung disease declined during treatment with imatinib[19, 20]. Th1 mechanisms appear to be anti-fibrotic[12, 13]. In SSc Treg cells that localize to the skin acquire properties of Th2 cells and produce IL-4 and IL-13[21].

The more recently described Th17 subset is also expanded in SSc blood and skin, and has been suspected to be pathogenic[22-26], although Th17 cytokines are not viewed as pro-fibrotic. One report suggests that Th22 cells, as well as Th2 and Th17, are expanded in patients with interstitial lung disease and SSc[27]. Important new evidence suggests that although prevalent in SSc skin, the Th17 cells might actually be protective against skin fibrosis[28]. Unlike IL-4+ cells, the IL-17A+ cells are in proximity to myofibroblasts in SSc skin, but in vitro IL-17A does not induce but rather inhibits myofibroblast differentiation, and instead increases collagenase expression. An inverse correlation was found between the density of IL-17A+ cells and the extent of skin thickness.

The genome-wide association studies in SSc[29] have led to the discovery of over 30 genes and gene regions, including both human leukocyte antigen (HLA) and non-HLA genes, identifying as SSc susceptibility loci. Most of these genes are associated with lymphocyte activation and signaling (e.g. *TNIP1*[30]), innate immunity (e.g. *IRF8*[31]), transcription factors (e.g. *STAT4*[32]) and cytokine receptors (e.g. *IL2RA*[33]). T cells from peripheral blood of women with SSc are activated, as judged by over-expression of CD40-ligand due to DNA demethylation[34] and that the level of DNA methyltransferase 1 is significantly decreased in patients with SSc[35]. Recently cytosine-phosphate-guanosine demethylation within the CD40L gene on the inactive X chromosome has been documented to contribute to CD40L overexpression in CD4+ T lymphocytes from female SSc patients[34] and could explain the female dominance in this condition.

In summary, available data suggests that Th2 cells are pathogenic and Th17 cells protective in SSc[12, 13]. However, there are large knowledge gaps related in part to small sample sizes of most prior studies, lack of longitudinal data (other than serial measurements of bronchoalveolar lavage T cells and IL-4-producing cells in a small cohort of patients who received imatinib in an open label fashion for scleroderma lung disease[20]), limited data concurrently obtained from skin and blood, and lack of data prospectively acquired during a clinical trial of an agent that is expected to affect T cells.

1.2.4 Effects of JAK inhibition on T Cells and Fibroblasts

JAK-STAT signaling is essential for the action of numerous cytokines that are either produced by T cells or that act on T cells. These include cytokines that utilize the so-called common gamma chain in their receptors (IL-2, -4, -7, -9, -15, -21), those that signal through gp130 (e.g. IL-6), cytokines that use dimeric receptors (e.g. IL-12, -23), the interferons, IL-10, growth factors and others[4]. Inherited deficiencies in JAKs lead to significant immune deficiencies with T cell dysfunction, and STAT deficiencies have been described that alter immune regulation.

Tofacitinib has effects on both numbers and function of various T and NK cell subsets[36-38]. Tofacitinib suppresses production of cytokines following T cell activation by anti-CD3, including IL-2, -4, -17 -22 and gamma-interferon, but not IL-2[39]. In this study, IL-4 production was especially sensitive to low concentrations of tofacitinib. In RA effects on Th1 and Th17 cells may be most important[40, 41]. On the other hand, Th2-driven processes are especially sensitive to JAK inhibition[42]. Interestingly, the function of Tregs seems to be relatively resistant to tofacitinib compared to other Teffector subsets[43]. Tofacitinib suppresses the differentiation of Th1, Th2 and Th17 cells through heterogeneous and distinct mechanisms. It also suppresses the *in vivo* response to LPS, lowering levels of TNF and IL-6 while raising the level of IL-10[2]. Its effect on T cell responses may be due in part to suppression of the stimulatory capacity (and of CD80/86 expression) of dendritic cells[44]. In contrast, the numbers of myeloid-derived suppressor cells were increased by tofacitinib in a mouse arthritis model[45].

Amelioration of inflammatory arthritis by JAK inhibition may in part reflect effects on synovial fibroblasts. JAK-3 is heavily phosphorylated in RA synovium and in synovial fibroblasts[46]. Although TNF does not use a JAK-STAT signaling pathway, TNF induction of chemokine secretion was blocked by tofacitinib, a phenomenon that was attributed to an autocrine loop involving JAK-STAT dependent type I interferon signaling critical to the response to TNF[47]. Tofacitinib also suppresses production of RANK-ligand (critical for osteoclast activation in RA) by both T cells and synovial cells[48].

The pro-fibrotic effects of TGF-beta, a critically-important cytokine in SSc, were recently reported to occur in part through a JAK-2 dependent pathway, which was inhibited by TG101209[49]. Tofacitinib was not used in these experiments. However, tofacitinib did suppress an animal model of graft-versus-host disease, an immune driven process that in some respects resembles SSc[50].

1.2.5 Clinical Safety of Tofacitinib

The following data includes two Phase 2 and five Phase 3 double-blind, controlled, multicenter trials. In these trials, patients were randomized to doses of tofacitinib (XELJANZ)5 mg twice daily (292 patients) and 10 mg twice daily (306 patients) monotherapy, XELJANZ 5 mg twice daily (1044 patients) and 10 mg twice daily (1043 patients) in combination with DMARDs (including methotrexate) and placebo (809 patients). All seven protocols included provisions for patients taking placebo to receive treatment with XELJANZ at Month 3 or Month 6 either by patient response (based on uncontrolled disease activity) or by design, so that adverse events cannot always be unambiguously attributed to a given treatment

The long-term safety population includes all patients who participated in a double-blind, controlled trial (including earlier development phase studies) and then participated in one of two long-term safety studies. The design of the long-term safety studies allowed for modification of XELJANZ doses according to clinical judgment. This limits the interpretation of the long-term safety data with respect to dose.

The most common serious adverse reactions were serious infections.

Overall Infections

In the seven controlled trials, during the 0 to 3 months' exposure, the overall frequency of infections was 20% in the 5 mg twice daily group, respectively, and 18% in the placebo group.

The most commonly reported infections with XELJANZ were upper respiratory tract infections, nasopharyngitis, and urinary tract infections (4%, 3%, and 2% of patients, respectively).

Serious Infections

In the seven controlled trials, during the 0 to 3 months' exposure, serious infections were reported in 1 patient (0.5 events per 100 patient-years) who received placebo and 11 patients (1.7 events per 100 patient-years) who received XELJANZ 5 mg or 10 mg twice daily.

In the seven controlled trials, during the 0 to 12 months' exposure, serious infections were reported in 34 patients (2.7 events per 100 patient-years) who received 5 mg twice daily of XELJANZ.

The most common serious infections included pneumonia, cellulitis, herpes zoster, and urinary tract infection.

Tuberculosis

In the seven controlled trials, during the 0 to 3 months' exposure, tuberculosis was not reported in patients who received placebo, 5 mg twice daily of XELJANZ, or 10 mg twice daily of XELJANZ.

In the seven controlled trials, during the 0 to 12 months' exposure, tuberculosis was reported in 0 patients who received 5 mg twice daily of XELJANZ and 6 patients (0.5 events per 100 patient-years) who received 10 mg twice daily of XELJANZ. Cases of disseminated tuberculosis were also reported. The median XELJANZ exposure prior to diagnosis of tuberculosis was 10 months (range from 152 to 960 days)

Opportunistic Infections (excluding tuberculosis)

In the seven controlled trials, during the 0 to 3 months' exposure, opportunistic infections were not reported in patients who received placebo, 5 mg twice daily of XELJANZ, or 10 mg twice daily of XELJANZ.

In the seven controlled trials, during the 0 to 12 months' exposure, opportunistic infections were reported in 4 patients (0.3 events per 100 patient-years) who received 5 mg twice daily of XELJANZ.

The median XELJANZ exposure prior to diagnosis of an opportunistic infection was 8 months (range from 41 to 698 days).

Malignancy

In the seven controlled trials, during the 0 to 3 months' exposure, malignancies excluding NMSC were reported in 0 patients who received placebo and 2 patients (0.3 events per 100 patient-years) who received either XELJANZ 5 mg or 10 mg twice daily.

In the seven controlled trials, during the 0 to 12 months' exposure, malignancies excluding NMSC were reported in 5 patients (0.4 events per 100 patient-years) who received 5 mg twice daily of XELJANZ.

The most common types of malignancy, including malignancies observed during the long-term extension, were lung and breast cancer, followed by gastric, colorectal, renal cell, prostate cancer, lymphoma, and malignant melanoma.

Laboratory Abnormalities

Lymphopenia: In the controlled clinical trials, confirmed decreases in absolute lymphocyte counts below 500 cells/mm3 occurred in 0.04% of patients for the 5 mg twice daily and 10 mg twice daily XELJANZ groups combined during the first 3 months of exposure.

Confirmed lymphocyte counts less than 500 cells/mm3 were associated with an increased incidence of treated and serious infections.

Neutropenia: In the controlled clinical trials, confirmed decreases in ANC below 1000 cells/mm3 occurred in 0.07% of patients for the 5 mg twice XELJANZ groups. There were no confirmed decreases in ANC below 500 cells/mm3 observed in any treatment group.

There was no clear relationship between neutropenia and the occurrence of serious infections.

Liver Enzyme Elevations: Confirmed increases in liver enzymes greater than 3 times the upper limit of normal (3× ULN) were observed in patients treated with XELJANZ. In patients experiencing liver enzyme elevation, modification of treatment regimen, such as reduction in the dose of concomitant DMARD, interruption of XELJANZ, or reduction in XELJANZ dose, resulted in decrease or normalization of liver enzymes.

In the controlled monotherapy trials (0–3 months), no differences in the incidence of ALT or AST elevations were observed between the placebo, and XELJANZ 5 mg, and 10 mg twice daily groups.

In the controlled background DMARD trials (0–3 months), ALT elevations greater than 3× ULN were observed in 1.0%, 1.3% and 1.2% of patients receiving placebo, 5 mg, and 10 mg twice daily, respectively. In these trials, AST elevations greater than 3× ULN were observed in 0.6%, 0.5% and 0.4% of patients receiving placebo, 5 mg, and 10 mg twice daily, respectively.

One case of drug-induced liver injury was reported in a patient treated with XELJANZ 10 mg twice daily for approximately 2.5 months. The patient developed symptomatic elevations of AST and ALT greater than 3× ULN and bilirubin elevations greater than 2× ULN, which required hospitalizations and a liver biopsy.

Lipid Elevations: In the controlled clinical trials, dose-related elevations in lipid parameters (total cholesterol, LDL cholesterol, HDL cholesterol, triglycerides) were observed at one month of exposure and remained stable thereafter. Changes in lipid parameters during the first 3 months of exposure in the controlled clinical trials are summarized below:

- Mean LDL cholesterol increased by 15% in the XELJANZ 5 mg twice daily arm
- Mean HDL cholesterol increased by 10% in the XELJANZ 5 mg twice daily arm
- Mean LDL/HDL ratios were essentially unchanged in XELJANZ-treated patients.

In a controlled clinical trial, elevations in LDL cholesterol and ApoB decreased to pretreatment levels in response to statin therapy.

In the long-term safety population, elevations in lipid parameters remained consistent with what was seen in the controlled clinical trials.

Serum Creatinine Elevations: In the controlled clinical trials, dose-related elevations in serum creatinine were observed with XELJANZ treatment. The mean increase in serum creatinine was <0.1 mg/dL in the 12-month pooled safety analysis; however, with increasing duration of exposure in the long-term extensions, up to 2% of patients were discontinued from XELJANZ treatment due to the protocol-specified discontinuation criterion of an increase in creatinine by more than 50% of baseline. The clinical significance of the observed serum creatinine elevations is unknown.

Other Adverse Reactions:

At least 2% or more of patients experiences the following adverse reactions: Nasopharyngitis, Diarrhea, Upper Respiratory Infection, Headache, Hypertension

1.3 Overall Risk/Benefit Assessment

This phase I/II study will assess the safety, tolerability and efficacy tofacitinib in patients with dcSSc. SSc is one of the most fatal rheumatic diseases, and is associated with substantial morbidity and many detrimental effects on health-related quality of life. There are no FDA approved drugs for SSc.

1.4 Study Rationale

This study will evaluate tofacitinib treatment in subjects 18 to 70 years of age with dcSSc in placebo controlled trial. This phase I/II study is intended to provide safety, and tolerability data sin participants with early dcSSc when dosed to target exposures similar to that used in adult participant with RA.

This study will evaluate safety and tolerability, along with descriptive efficacy and pharmacokinetics of tofacitinib as treatment for early dcSSc. After 24 weeks of double blind treatment, participants will then be able to continue into the open label phase during which they will receive tofacitinib 5 mg twice a day for 24 weeks. The primary objective of this study will be to demonstrate acceptable safety and tolerability of tofacitinib in early dcSSc.

1.5 Summary of Rationale for the Proposed Study

Tyrosine kinase inhibitors have been proposed as logical targets for novel treatment strategies in SSc[1], but not much attention has yet been given to the possible use of JAK inhibitors in SSc. However, the various considerations noted above regarding the pathogenesis of SSc and the target effects of tofacitinib provide a compelling case for *in vitro*, animal model and human clinical testing of this agent in SSc. The ability of tofacitinib to suppress both Th2 differentiation and the pro-fibrotic effects of Th2 cytokines, while sparing Treg function is a combination of effects that may make this drug especially suitable for SSc.

1.6 Dose Rationale

The doses of tofacitinib to be evaluated within this study are selected based on the regulatory approval for RA. We plan to administer tofacitinib at 5 mg twice daily (BID), an approved dose of tofacitinib in adult RA patients in most countries.

2. STUDY OUTCOMES

2.1 Primary Outcomes

The **primary study endpoint** will be the proportion of participants who experience Grade 3 (severe) or higher adverse events that occur at or before Week 24.

2.2 Secondary Outcomes

The **secondary study endpoints** will include:

- Number of Grade 3(severe) or higher adverse events that occur at or before Weeks 12, 36, and 48

- Number of Grade 2 (moderate) or higher adverse events that occur at or before Weeks 12, 24, 36, and 48
- Number of AE's of special interest (AESI) at Weeks 12, 24, 36, and 48
- Change in modified Rodnan Skin Score (mRSS) at Weeks 12, 24, 36, and 48
- Provisional American College of Rheumatology Combined Response Index in Systemic Sclerosis at Weeks 12 , 24, and 48.

2.3 Exploratory Outcomes

To assess the efficacy of treatment using 5mg of tofacitinib versus placebo. These **exploratory study endpoints** will include any change in the following scores at the following time points highlighted below.

- Proportion of subjects with mRSS improvement of 20%, 40%, and 60% at weeks: 12 and 24
- Comparison of average, most representative, and maximum skin score methodology for mRSS
- Physician's global assessment on a Likert scale at weeks: 12, 24,36 and 48
- Patient's global assessment on a Likert scale at weeks: 12, 24,36 and 48
- Health-related quality of life (HRQOL) using PROMIS-29 2.0 at weeks: 12, 24,36 and 48
- Physical function as assessed by the scleroderma health assessment questionnaire-disability index (SHAQ-DI) at weeks: 12, 24,36 and 48
- Gastrointestinal symptoms as assessed by UCLA SCTC GIT 2.0 at weeks: 12, 24,36 and 48
- Scleroderma-related skin symptoms assessed by PRO-SRSS at weeks: 12, 24, 36 and 48
- Percent predicted FVC at weeks: 12, 24, and 48
- Change in left ventricular ejection fraction at week 24
- Change in tricuspid regurgitation jet at week 24

3. REGULATORY OBLIGATIONS

3.1 Good Clinical Practice

This study will be conducted in accordance with Good Clinical Practice (GCP), as defined by the International Conference on Harmonization (ICH) and in accordance with the ethical principles underlying European Union Directive 2001/20/EC and the United States Code of Federal Regulations, Title 21, Part 50 (21CFR50).

The study will be conducted in compliance with the protocol. The protocol, any amendments, and the participant informed consent will receive Institutional Review Board/Independent Ethics Committee (IRB/IEC) approval/favorable opinion before initiation of the study.

Study personnel involved in conducting this study will be qualified by education, training, and experience to perform their respective tasks. This study will not use the services of

study personnel where sanctions have been invoked or where there has been scientific misconduct or fraud (e.g., loss of medical licensure; debarment).

3.2 Institutional Review Board

The investigator must have written and dated approval/favorable opinion from the IRB for the protocol, consent form, participant recruitment materials/process (e.g., advertisements), along with any other written information that is planned to be provided to participants, prior the initiation of the study.

The investigator should provide the IRB/IEC with reports, updates, and other information (e.g., expedited safety reports, amendments, and administrative letters) according to regulatory requirements or local institution procedures.

3.3 Informed Consent

The investigator, or a person designated by the investigator, will obtain written informed consent from each subject or the subject's legally acceptable representative, before any study-specific activity is performed, unless a waiver of informed consent has been granted by an IRB/EC. The investigator will retain the original of each subject's signed consent document.

The rights, safety, and well-being of the study participants are the most important considerations and should prevail over interests of science and society.

3.4 Confidentiality

The Investigator must ensure that the subject's confidentiality is maintained. On the case report forms or other documents submitted to the Sponsor or designee, subjects should be identified by unique initials and a subject study number only. Documents that are not for submission to the Sponsor or designee (e.g., signed informed consent/assent forms) should be kept in strict confidence by the Investigator.

4. INVESTIGATIONAL PLAN

4.1 Study Design and Duration

This is a phase I/II placebo controlled study to evaluate the efficacy, safety, and tolerability, and pharmacokinetics of tofacitinib as a treatment for early dcSSc. The study will enroll approximately 15 Subjects from 18 to 70 [inclusive] years of age with active dcSSc. Subjects will be randomized to tofacitinib vs. placebo in a 2:1 ratio at 5 mg twice a day for 24 weeks. Subjects will be able to continue into the open label phase during which they will receive tofacitinib 5 mg twice a day for 24 weeks. There will be also be a follow up phone call which will occur 30 days post completing/discontinuing study medication. This study will be conducted over a period of approximately 2 years. Two sites are expected to participate in this study.

4.2 Study Schema

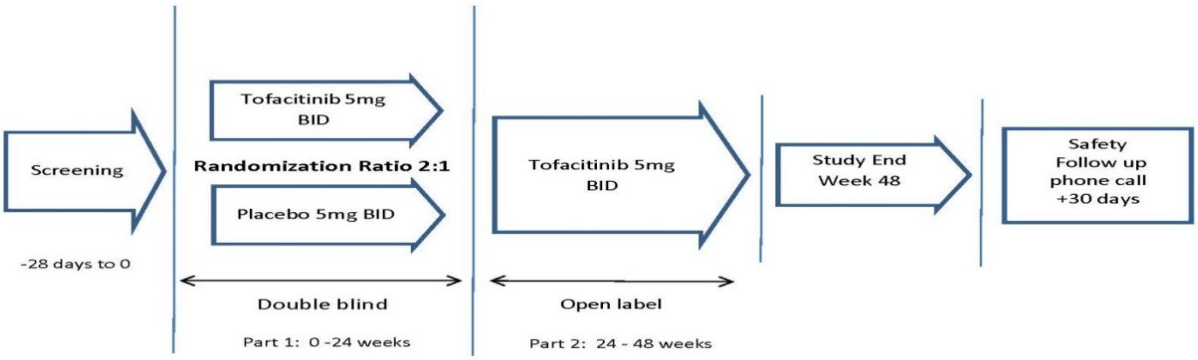

4.3 Study Population

This study can fulfill its objectives only if appropriate subjects are enrolled. The following eligibility criteria are designed to select subjects for whom participation in the study is considered appropriate. All relevant medical and nonmedical conditions should be taken into consideration when deciding whether a particular subject is suitable for this protocol. Subjects will be able to participate with this study drug in combination with methotrexate or other non-biologic immunosuppressive agents.

4.4 Inclusion Criteria

Subjects must meet all of the following inclusion criteria to be eligible for enrollment in the study:

1. Diagnosis of SSc, as classified using the 2013 American College of Rheumatology/ European Union League Against Rheumatism classification of SSc[51].
2. dcSSc as defined by 2001 LeRoy and Medsger[52]
3. Disease duration ≤ 60 months (defined as time from the first non−Raynaud phenomenon manifestation)
4. mRSS units ≥ 10 and ≤ 45 at screening.
5. Agreement to receive varicella-zoster vaccination or have received vaccination prior to screening.
6. Oral corticosteroids (≤ 10 mg/day of prednisone or equivalent) are permitted if the patient is on a stable dose regimen for ≥ 2 weeks prior to and including the baseline visit.
7. PDFE-5 inhibitors for Raynaud's and digital ulcers are permitted to use as oral monotherapy
8. Age ≥ 18 years and ≤ 70 years
9. Ability to provide informed consent.

4.5 Exclusion Criteria

Subjects with any of the following characteristics/conditions will <u>not</u> be included in the study:

1. Rheumatic disease other than dcSSc; it is acceptable to include patients with fibromyalgia, Sjogren syndrome, and scleroderma-associated myopathy
2. Limited cutaneous SSc or sine scleroderma
3. Significant trauma or major surgery (including joint surgery) within 8 weeks prior to baseline.
4. Any infected ulcer at screening
5. Subjects with any serious bacterial infection within the last 3 months, unless treated and resolved with antibiotics, or any chronic bacterial infection (e.g., chronic pyelonephritis, osteomyelitis, or bronchiectasis)
6. Oral corticosteroids >10 mg/day of prednisone or equivalent.
7. Treatment with anti-CD20 6 months prior to baseline and/or B cell counts less than the lower limit of normal
8. Hydroxychloroquine >400 mg/day, methotrexate >25 mg/week, D-Penicillamine >1000mg/day or mycophenolate mofetil > 2 grams/day prior to baseline. **Subjects can be on combination therapy of hydroxychloroquine and methotrexate or hydroxychloroquine and mycophenolate mofetil and must have been on a stable dose for at least 1month prior to baseline visit.
9. Prior history of treatment in the 3 months prior to baseline with biological DMARDs potent immunosuppressants such as cyclosporine and azathioprine
10. Treatment with etanercept within ≤ 2 weeks of baseline: infliximab, certolizumab, golimumab, abatacept, tociluzimab, or adalimumab within ≤ 8 weeks of baseline; and anakinra within ≤ 1 week prior to the baseline visit.
11. Intravenous corticosteroids within 2 weeks prior to baseline visit.
12. Treatment with any investigational agent ≤ 4 weeks prior to baseline (or 5 half-lives of the investigational drug, whichever is longer)
13. Other investigational or marketed biologics with immunomodulatory properties within 3 months prior to baseline.
14. Any prior treatment with cell-depleting therapies other than anti-CD20 such as CAMPATH, anti-CD4, anti-CD5, anti-CD3, anti-CD19
15. Any prior treatment with chlorambucil, bone marrow transplantation, or total lymphoid irradiation
16. Vaccinated or exposed to a live/attenuated vaccine (other than Zostavax/Shingrix®) ≤ 6 weeks prior to baseline; or is expected to be vaccinated or to have household exposure to these vaccines during treatment or during the 6 weeks following discontinuation of study medication. (**See additional inclusion for obtaining Zostavax/Shingrix® prior to entering the study)
17. Pulmonary disease with FVC ≤ 50% of predicted, or DLCO (uncorrected for hemoglobin) ≤ 40% of predicted
18. History of pulmonary arterial hypertension (PAH) with mean PAP> 30 mmHg on right heart catheterization requiring subcutaneous or intravenous prostacyclin or dual use of oral PAH therapies
19. Subjects at risk for tuberculosis (TB).
    A. Specifically excluded from this study will be participants with a history of active TB within the last 3 years, even if it was treated; a history of active TB greater than

3 years ago, unless there is documentation that the prior anti-TB treatment was appropriate in duration and type; current clinical, radiographic, or laboratory evidence of active TB; (TB results within 30 days of screening will be accepted and will not to be repeated.

B. Latent TB at or within 30 days of screening,

history of or current positive purified protein derivative tuberculin skin test (PPD) (> 5mm induration, regardless of Bacille Calmette Guerin [BCG] vaccine and/or QuantiFERON Gold, a negative chest x-ray, and no symptoms or risk factors), unless one month of prophylaxis has been completed prior to inclusion

- An indeterminate QuantiFERON® unless followed by a subsequent negative PPD or negative QuantiFERON® or a consultation with and clearance by local infectious disease (ID) department is required.

20. Positive for hepatitis B surface antigen at or within 30 days of screening
21. Positive for hepatitis C antigen at or within 30 days of screening
22. Current or recent history of uncontrolled clinically significant renal, hepatic, hematologic, gastrointestinal, metabolic, endocrine, pulmonary, cardiac or neurologic disease.
23. History of HIV (as determined by medical records or patient reported).
24. History of diverticulitis or chronic, ulcerative lower GI disease such as Crohns disease, ulcerative colitis, or other symptomatic, lower GI conditions that might predispose a patient to perforations.
25. Pregnant or breastfeeding female subjects; land female subjects of childbearing potential who are unwilling or unable to use a highly effective method of contraception as outlined in the protocol for the duration of the study and for at least 28 days after discontinuation of study drug.
26. Severe acute or chronic medical or psychiatric condition or laboratory abnormality that may increase risk associated with study participation and in the judgment of the investigator would make the subject inappropriate for entry into this study.
27. History of SSc Renal Crisis within the 6 months prior to baseline.
28. Any of the following lab results at screening:[a]

- Hemoglobin <9 g/dL or Hematocrit <30%
- White Blood Cell count <3.0 x 10^9/L;
- Absolute Neutrophil count <1.2 x 10^9/L;
- White Blood Cell count <3.0 x 10^9/L;
- Absolute Neutrophil count <1.2 x 10^9/L;
- Platelet count <100 x 10^9/L;
- Absolute Lymphocyte count <0.75 x 10^9/L.
- ALT or AST > 1.5 × the upper limit of normal (ULN) of normal at screening or any uncontrolled clinically significant laboratory abnormality that would affect interpretation of study data or the patient's participation in the study
- Total bilirubin > ULN at Screening.
- Estimated glomerular filtration rate [GFR] <40mL/min/1.73 m^2

29. History of recurrent (more than one episode) herpes zoster or disseminated (at least one episode) herpes zoster, or disseminated (at least one episode) herpes simplex
30. History of any lymphoproliferative disorder, such as Epstein Barr Virus (EBV) related lymphoproliferative disorder, history of lymphoma, leukemia, or signs and symptoms suggestive of current lymphatic disease.
31. History of any malignancy in the last 5 years with the exception of adequately treated or excised basal cell or squamous cell or cervical cancer in situ.
32. History of alcohol or substance abuse, unless in full remission for greater than 6 months prior to first dose of study drug.

[a] Please note thresholds for lab parameters indicated throughout this protocol can differ from the published, current package insert due to a new disease and concomitant immunosuppressive therapies.

4.6 Reproductive Status

Definition of Women of Child-Bearing Potential (WOCBP). WOCBP comprises women who have experienced menarche and who have not undergone successful surgical sterilization (hysterectomy, bilateral tubal ligation, or bilateral oophorectomy) or who are not post-menopausal. WOCBP therefore includes women using the following methods to prevent pregnancy: Oral contraceptives, other hormonal contraceptives (vaginal products, skin patches, or implanted or injectable products), or mechanical products such as intrauterine devices or barrier methods (diaphragm, condoms, spermicides); women who are practicing abstinence; and women who have a partner who is sterile (e.g., due to vasectomy).

The following women are defined as post-menopausal:

- Women who have had amenorrhea for ≥12 consecutive months (without another cause)
- Women who have irregular menstrual periods and a documented serum FSH level > 35 mLU/mL as part of her medical history.
- Women who are taking hormone replacement therapy (HRT).

WOCBP must be using an acceptable method of contraception to avoid pregnancy throughout the study and for at least 28 days after the last dose of study drug in such a manner that the risk of pregnancy is minimized.
Acceptable methods of contraception are listed below.

- Hormonal methods: combined (estrogen and progestogen containing) hormonal contraception associated with inhibition of ovulation (oral, intravaginal or transdermal); progestogen-only hormonal contraception associated with inhibition of ovulation (oral, injectable or implantable)
- Barrier and mechanical methods: intrauterine device (IUD); intrauterine hormone-releasing system (IUS), male or female condom with or without spermicide; cap, diaphragm or sponge with spermicide
- Bilateral tubal occlusion

- Vasectomized partner (provided that partner is the sole sexual partner of the WOCBP trial participant and that the vasectomized partner has received medical assessment of the surgical success of the vasectomy)
- True abstinence (where abstinence is a lifestyle choice made by the subject and not only for the duration of the trial)

Periodic abstinence (for example, calendar, ovulation, symptom-thermal and post ovulation methods), abstinence for the duration of the trial and the withdrawal method are not considered acceptable forms of contraception.

WOCBP must have a negative serum or urine pregnancy test result (minimum sensitivity 25 IU/L or equivalent units of HCG) within 0 to 48 hours before the first dose of study drug. Women must not be breast-feeding.

4.7 Other Considerations for potential subjects

4.7.1 Vaccine and Exposure to Infections Guidelines

Subject Specific Recommendations

It is recommended that all subjects should be up-to-date with respect to standard of care vaccinations or SSc guidelines. Vaccination of subjects with live components is prohibited within the 6 weeks prior to first dose of study drug and throughout the study.

Guidance Regarding Household Contact Vaccine-Related Exposure

Current routine household contact with children and others who have been vaccinated with live vaccine components may pose a risk during treatment and for 6 weeks following completion of the study. Some of these vaccines include varicella ("chickenpox") vaccine oral polio vaccine, and the inhaled flu vaccine. Following vaccination with live component vaccines, the virus may be shed in bodily fluids, including stool, and there is a potential risk that the virus may be transmitted. General guidelines for immunosuppressed subjects suggest that exposure (through routine contact) should be avoided following vaccination (of others) with these vaccines for the stated time period:

- Varicella or attenuated typhoid fever vaccination for 4 weeks following vaccination.
- Oral polio vaccination for 6 weeks following vaccination.
- Attenuated rotavirus vaccine for 10 days following vaccination.
- FluMist® (inhaled flu vaccine) for 1 week following vaccination.

Subjects should avoid exposure to infected persons and contact the Investigator promptly should they develop signs or symptoms of infection.

Due to the increased risk of re-activating varicella-zoster virus, participants will be receiving zoster vaccinations before entering the study. Participants who already have documented zoster vaccination prior to screening will be able to continue on with randomizing once entry criteria are confirmed.

All participants who have not received Zostavax ® prior will follow the timeline indicated below considering whether or not they are on background immunosuppressive therapy. Participants currently on background therapy (i.e.: methotrexate, mycophenolate, D-Penicillamine) will be asked to temporarily hold these medications for 14 days, receive Zostavax®, wait another 14 to re-start the background medication and then 28 days later can continue on to randomization. If the participant is on mycophenolate mofetil at > 2grams/day (if on Mycophenolic acid prior, must be re-started ≤1440 mg/day) at the screening visit, the mycophenolate will need to be started at ≤2 grams/day. Participants on stable, acceptable criteria dosages of hydroxychloroquine as background therapy are not required to interrupt their background therapy and can follow the same timeline indicated for those not on background immunosuppressive therapy. Participants not on background immunosuppressive therapy will only have to wait for 14 days post vaccination to be randomized.

Possible screening timelines:

1. Screening → Participant had zostavax →continue to randomization after criteria is confirmed

2. Screening→Participant is not on background therapy (or is on stable hydroxychloroquine) but requires zostavax→ confirm criteria and participant obtains zostavax→randomization may occur 14 days post vaccination

3. Screening→Participant is on a background therapy that requires interruption for vaccination→ Confirm eligibility, temporarily hold the medications for 14 days→ Receive the zostavax→ Re-start at eligible doses (ensuring ≤ 2 grams mycophenolate/ methotrexate ≤25)14 days post vaccination→ ensure stable dosing by waiting an additional 28 days→ Randomization (up to 65 day window).

Participants can also enter the study having received the Shingrix vaccine. If both doses of Shingrix have been received the participant may start screening and may randomize as long as 4 weeks have passed since receiving the 2nd dose. Alternatively, a participant can begin screening with only one dose if they will be receiving the 2nd dose and can complete the 4 weeks waiting period prior to randomization and still staying within the window.

The screening window will be up to 65 days to ensure these steps are all completed.

4.7.2 Elective Surgery

During the course of this trial, no elective surgery should be scheduled without first consulting with the site investigator.

Subjects who do require surgery should temporarily discontinue study medication for one week prior to the surgical procedure and remain off study medication after the surgical procedure until sutures/staples are removed. If absorbing sutures or chemical closure methods are utilized, study medication can be resumed when the operative site is sufficiently healed and risk of infection is minimal.

4.8 Discontinuation of Subjects from Treatment

Subjects MUST discontinue investigational product (and non-investigational product at the discretion of the investigator) for any of the following reasons:

- Withdrawal of informed consent (participant's decision to withdraw for any reason).
- Any clinical adverse event, laboratory abnormality, or intercurrent illness which, in the opinion of the investigator, indicates that continued participation in the study is not in the best interest of the participant.
- Pregnancy
- Instruct WOCBP to contact the investigator or study staff immediately if they suspect they might be pregnant (e.g., missed or late menstrual period) at any time during study participation. Institutional policy and local regulations should determine the frequency of on-study pregnancy tests for WOCBP enrolled in the study.
- Loss of ability to freely provide consent through imprisonment or involuntary incarceration for treatment of either a psychiatric or physical (e.g., infectious disease) illness.
- Anaphylaxis or serious allergic reaction

All participants who discontinue should comply with returning to the clinic for an Early Termination visit 30 days post last dose of study drug as outlined in Section 6. For subjects whom cannot comply with returning to the clinic, then a follow up phone call should occur 30 days post last dose. The only exception to this requirement is when a participant withdraws consent for all study procedures or loses the ability to consent freely (i.e., is imprisoned or involuntarily incarcerated for the treatment of either a psychiatric or physical illness). If a participant withdraws before completing the study, the reason for withdrawal must be documented appropriately.

5. STUDY TREATMENT

5.1 Treatment

Definition of Investigational Product: A pharmaceutical form of an active substance or placebo being tested or used as a reference in a clinical study, including products already with a marketing authorization but used or assembled (formulated or packaged) in a way different from the authorized form, or used for an unauthorized indication, or when used to gain further information about the authorized form. In this protocol, the investigational product is tofacitinib.

Definition of Non-Investigational Product: Other medications used in the study as support or escape medication for preventative, diagnostic, or therapeutic reasons as components of a given standard of care. In this protocol, the non-investigational products are medications used for the management of their comorbidities.

5.1.1 Identification

Product Description and Dosage	Route of Administration	Potency	Appearance	Storage Conditions (per label)
**Tofacitinib**	Oral	5mg tablets	White, round, immediate-release film-coated tablets	Store at 20°C to 25°C (68°F to 77°F
**Placebo for tofacitinib**	Oral	5mg tablets	White, round, immediate-release film-coated tablets	Store at 20°C to 25°C (68°F to 77°F

5.2 Method of Assigning Subjects to Treatment

Patients will be randomized after all screening assessments have been completed and the investigator has verified that eligibility criteria have been met. At the time of randomization, patients will be assigned a unique randomization number; no subject may begin treatment prior to randomization. Eligible subjects will be randomized to tofacitinib or placebo in a 2:1 manner. The statistician will prepare the randomization schedule, using computer-generated block randomization with the block size(s) only known by the statistician.

5.3 Blinding and Unblinding

- This is a double-blind study. The study staff (with the exception of the study pharmacist) and the patient are blinded to the treatment assignment.
- Blinding is critical to the integrity of this clinical study. However, in the event of a medical emergency or pregnancy in a participant, in which knowledge of the investigational product is critical to the participant's management, the blind for that participant may be broken.
- Before breaking the blind of an individual participant's treatment, the investigator should have determined that the information is necessary, i.e., that it will alter the participant's immediate management. A discussion with the protocol chairs is encouraged prior to proceeding with unblinding. The investigator holds sole responsibility for the decision to unblind in case of emergency. In many cases, particularly when the emergency is not investigational product-related, the problem may be properly managed by assuming that the participant is receiving active product without the need for unblinding.

5.4 Concomitant Medications

All concomitant medication taken during the study must be recorded with generic name of the medication, indication, daily dose, and start and stop dates of administration. A subject who is receiving an allowed concomitant medication for any reason must be on a locally-approved medication and dose that is considered standard-of-care for the treated indication. Medications taken after informed consent is obtained but before the first dose of study medication will be documented as prior medications. Medications taken after the first dose of study drug has been

administered will be documented as concomitant medications.

5.5 Prohibited Medications

After enrollment, subjects are allowed to continue on their stable background SSc therapy, which can include nonsteroidal anti-inflammatory drugs (NSAIDs), cyclooxygenase-2 (COX-2) inhibitors, allowed DMARDs (methotrexate – see Eligibility), and Corticosteroids.

Use of tofacitinib in combination with biologic disease-modifying antirheumatic drugs (DMARDs) or potent immunosuppressants such as azazathioprine and cyclosporine is not allowed.

5.5.1 Other Restrictions and Precautions

Serious infections leading to hospitalization or death, including tuberculosis and bacterial, invasive fungal, viral, and other opportunistic infections, have occurred in patients receiving tofacitinib.

- Avoid use of **tofacitinib** during an active serious infection, including localized infections.
- **Gastrointestinal Perforations** – Use with caution in patients that may be at increased risk.
- **Laboratory Monitoring** – Recommended due to potential changes in lymphocytes, neutrophils, hemoglobin, liver enzymes and lipids.
- **Immunizations** – Live vaccines

6. STUDY ASSESSMENTS AND PROCEDURES

Study-related procedures and outcome measures that will be performed as part of this protocol are listed below. Specific times at which each test will be performed are summarized in the schedule of evaluations table (section 7.1).

- **Complete medical history -**Medical history will be performed as part of screening activities and standard medical care.** Other medical records such as HRCT reports and images, laboratory work, and other tests done as part of clinical care may be accessed for future research data purposes.
- **Physical examination -** A standard complete physical examination will be performed, with the addition of the relevant assessments listed below (See Physician Assessments)
- **Vital signs -** Vital signs will include: pulse, blood pressure, temperature (Cº), height (cm), and weight (kg) (height and weight only collected at screening).
- **Assessment of Signs and Symptoms**

  Physician Assessments
  - **Modified Rodnan skin score (mRSS):** is a validated physical examination method for estimating skin induration. It is correlated with biopsy measures of skin thickness and reflects prognosis and visceral involvement, especially in early disease. It is scored on a 0 (normal) to 3+ (severe induration) ordinal scales over 17 body areas, with a maximum score of 51 and is used to categorize severity of SSc. mRSS will be assessed 3 different ways including: average, maximum

scare and representative area. It has been extensively used as primary/ secondary outcome in RCTs.
Collection: ***ALL STUDY VISITS with the exception of SAFETY FU phone call***

- **Physician's Global Assessment for overall disease:** This assessment represents the physician's assessment of the patient's current disease activity on a 0-10 Likert scale. "On a scale of 0-10, how was your patient's overall health in the last week? 0 = Excellent; 10 = Extremely Poor". **Investigators will be asked to review their previous score before answering the physician global assessment at each visit.**

	**WEIGHT**
**Patient skin perception** ***(any progression felt by the patient in the last month)***	**1.5**
**Digital ulcers** ***(ongoing ischemic DU)***	**1.5**
**Modified Rodnan skin score:**<br>**any value > 18:**<br><br>**for mRSS score equal or lower to 18:**	**1.5**<br><br>**mRSS score x 0.084**
**Tendon friction rubs: yes/no**	**2.25**
**C-reactive protein > 1 mg/dl: yes/no**	**2.25**
**DLCO < 70% of the predicted value: Yes/no**	**1.0**

Collection: ***ALL STUDY VISITS with the exception SAFETY FU phone call***

- **Combined Response Index in Systemic Sclerosis (CRISS)**: CRISS is a composite measure for early dcSSc. It is determined in a 2-step process that assesses the probability of deterioration (step 1) and of improvement (step 2), where each probability ranges from 0.0 to 1.0. The first step assesses whether the patient has had a significant decline in renal or cardiopulmonary involvement. The second step assesses the probability of improvement by incorporating changes in the modified Rodnan skin score, percent predicted forced vital capacity (FVC), patient and physician global assessments, and SHAQ-DI over 1 year. **Assessed at 12, 24, and 48 weeks.**
- **European Activity Index**
  This index assesses disease activity utilizing 6 assessments: patient perception of skin involvement, mRSS, digital ulcers, tendon friction rubs, C-Reactive protein values and predicted DLCO over 1 year. **Assessed at baseline, 24 and 48 weeks.**

Patient reported Outcomes

- **PROMIS-29 Profile v2.0 measure:** The National Institutes of Health (NIH) Patient-Reported Outcomes Measurement Information System (PROMIS®) Roadmap initiative (www.nihpromise.org) is a cooperative research program designed to develop, evaluate, and standardize item banks to measure patient-reported outcomes (PROs) across different medical conditions as well as the US population. PROMIS-29 Profile v2.0 measure contains 29 items, which includes four items each from physical function, anxiety, depression, fatigue, sleep disturbance, pain interference, and satisfaction with social roles domains, and a single item on pain intensity. With the exception of physical function which does not include a time frame, all item banks reference the past 7 days. It is part of the NHLBI-funded RCT of cyclophosphamide vs. mycophenolate mofetil in SSc-associated interstitial lung disease (SSc-ILD).
  Collection: *Baseline, Week 12, 24,36,48*

- **Patient's Global Assessment for overall disease:** This assessment represents the patient's assessment of the patient's global scleroderma on a 0-10 Likert scale. "On a scale of 0-10, how was your overall health in the last week? 0=Excellent; 10=Extremely Poor. **Participants will be asked to review their previous score before answering the patient global assessment at each visit. Collection: *Baseline, Week 12, 24,36,48***

- **SHAQ-DI:** The SHAQ-DI is a disease-targeted, musculoskeletal-targeted measure intended for assessing functional ability in arthritis. A self-administered 20-question instrument assesses a patient's level of functional ability and includes questions that involve both upper and lower extremities. The SHAQ-DI score ranges from 0 (no disability) to 3 (severe disability). It has a 7-day recall period and has been extensively used in SSc. 5 visual analog scales are included in the scleroderma-HAQ assessing burden of digital ulcers, Raynaud's, gastrointestinal involvement, breathing, and overall disease.
  Collection: *Baseline, Week 12, 24,36,48*

- **UCLA SCTC GIT 2.0:** This validated instrument assesses scleroderma-related gastrointestinal symptoms. It has 7 scales and a final composite score http://uclascleroderma.researchcore.org.
  Collection: *Baseline, Week 12, 24,36,48*

- **PRO for Scleroderma-related Skin Symptoms (PRO-SRSS):** This instrument focuses on seven skin symptoms: skin tightness, skin thickening, skin sensitivity, skin color, itchiness, pain from skin tightness, and skin puffiness; the baseline version has the additional symptom, skin ulcers. The PRO-SRSS has a 7-day recall. **Collection: *Baseline, week 12, 24,36 and 48.***

- **Patient-*reported worsening of the skin disease***

- **Pulmonary function tests (PFTs)**
  - **1. Spirometry:** Carried out by either certified pulmonary function technologists (National Board of Respiratory Care) or experienced staff that meets American Thoracic Society (ATS) recommendations. All spirometry equipment and procedures will conform to the most recently published standards of the ATS/ERS Task Force. Forced expiratory maneuvers will be performed at least in triplicate with the minimal requirement that three maneuvers are "acceptable" and that two of these maneuvers meet end-of-test and repeatability criteria for FVC and FEV1.
    Collection: *Screening, week 12, 24 and 48*
  - **2. Single-breath diffusing capacity for carbon monoxide (DLCO):** performed in accordance with recently published ATS/ERS guidelines using equipment and testing techniques that meet ATS/ERS requirements. At least 2 acceptable tests that meet repeatability criteria will be performed and the mean DLCO value (uncorrected for hemoglobin) from acceptable measurements will be reported.
    Collection: *Screening, 24, and 48.*

- **Echocardiogram with Doppler**
  Echocardiograms will be performed to assess ejection fraction and TR jet to determine pulmonary arterial hypertension. This assessment will also be used to determine systolic or diastolic dysfunction as well as arterial or ventricular enlargement.
  Collection: *Screening/Prior to Randomization: Week 24*

- **Skin Biopsy**
  We will perform two 3-mm skin biopsies. The skin biopsies are required for participation. They will be shipped to University of Pittsburgh. Skin tissue from patients with diffuse cutaneous SSc (dcSSc) before and after toficitinib or placebo will be collected. Single-cell RNA-sequencing analysis will be performed.
  Collection: *Baseline, Week 6*

- **Blood samples:** 40 ml of blood will be obtained from all study participants at baseline, Week 6, and Week 24 and shipped from participating centers to the University of Michigan. The analysis will include proteomics analysis, RNA analysis, flow cytometry and auto-antibody measurements.
  Collection: *Baseline, Week 6, Week 24*

6.1 Schedule of Events

The schedule of events table provides an overview of the protocol visits and procedures. Section 6 of the protocol provides detailed information on each procedure and assessment required for compliance with the protocol.

The investigator may schedule visits (unplanned visits) in addition to those listed on the schedule of events in order to conduct evaluations or assessments required to protect the well-being of the subject.

	Randomized Treatment Period						Open-Label Treatment Period				Safety FU
**Study Visit Date[a]**	**Screening**	**Baseline**	**Week 6**	**Week 12**	**Phone Call Week 18**	**Week 24[m]**	**Week 30**	**Week 36**	**Phone Call Week 42**	**Week 48**	**End of Study Phone Call +30d**
**Study Drug Dispensed**											
Tofacitinib or placebo		X	X	X							
Open-label tofacitinib						X		X			
**General**											
Informed Consent	X										
Eligibility Review	X										
Medical History	X										
Demographics	X										
Vaccine[b]	X										
**Clinical Assessments**											
Physical Exam	X		X	X		X	X	X		X	
Vital Signs[c]	X	X	X	X		X	X	X		X	
Assessment of Symptoms	X	X	X	X		X	X	X		X	
mRSS[d]	X	X	X	X		X	X	X		X	
CRISS				X		X				X	
European Activity Index	X					X				X	
Physician Assessments[e]	X	X	X	X		X	X	X		X	
Patient Reported Outcomes[f]		X		X		X		X		X	
Adverse Events		X	X	X	X	X	X	X	X	X	X
Pulmonary Function Tests[g]	X			X**FVC only		X				X	
Echocardiogram w/ doppler	X					X					
**Laboratory Assessments[h]**											
PPD/Quantiferon[i]	X										
ESR	X		X	X		X	X	X		X	
CRP	X					X				X	
Hepatitis B and C [j]	X										
CBC w/ diff	X	X	X	X		X	X	X		X	
Lipid Panel [n]	X		X				X	X			
Comp Panel	X	X	X	X		X	X	X		X	
Pregnancy Test (Urine)[k]	X	X	X	X		X	X	X		X	
**Research Assessments**											
Biomarkers		X	X			X					

Skin biopsy[l]		X	X								

[a] Screening window will be -65 to 0 days / Remaining Visit windows will be ± 10 days
[b] Follow zostervax or shingrix dosing scheduleprovided on page 20 of this protocol.
[c] Blood pressure, Heart Rate, and Temperature. Height and Weight will also be collected at Screening visit.
[d] mRSS will be assessed as one of the inclusion criterion
[e] Physician Assessments include: Physician's global assessment, Tendon Friction Rub, Swollen and Tender Joint Count, Joint Contractures, and Digital Ulcer assessments.
[f] Patient Reported Outcomes include: patient's global assessment, PROMIS-29 2.0, SHAQ-DI, PRO-SRSS and UCLA GIT 2.0
[g] FVC and DCLO (Assessments performed within two 2 weeks prior to the screening date are allowable and do not need to be repeated.)
[h] All laboratory assessments will be performed at local laboratory
[i] Negative results from two weeks prior to the screening visit will be accepted and will not have to be assessed at screening.
[j] For women of child bearing potential
[k] 40 mL for proposed blood studies described in the protocol
[l] Two 3-mm skin biopsies will be collected
[m] Should a subject terminate early the subject will be brought in to complete all assessments included at the Week 24 visit. Study drug however would not be provided but returned at this time.
[n] All lipid draws require 4 hour fasting.

7. ADVERSE EVENT REPORTING

7.1 Adverse Events

An *Adverse Event (AE)* is defined as

- Any new untoward medical occurrence or worsening of a pre-existing medical condition in a patient or clinical investigation participant administered an investigational (medicinal) product and that does not necessarily have a causal relationship with this treatment. An AE can therefore be any unfavorable and unintended sign (including an abnormal laboratory finding, for example), symptom, or disease temporally associated with the use of investigational product, whether or not considered related to the investigational product.
- Adverse events can be spontaneously reported or elicited during open-ended questioning, examination, or evaluation of a participant. (In order to prevent reporting bias, participants should not be questioned regarding the specific occurrence of one or more AEs.)

7.2 Serious Adverse Events

A *Serious Adverse Event (SAE)* is any untoward medical occurrence that at any dose:

- Results in death
- Is life-threatening (defined as an event in which the participant was at risk of death at the time of the event; it does not refer to an event which hypothetically might have caused death if it were more severe)
- Requires inpatient hospitalization or causes prolongation of existing hospitalization (see note below for exceptions)
- Results in persistent or significant disability/incapacity
- Is a congenital anomaly/birth defect
- Is an important medical event, defined as a medical event that may not be immediately life-threatening or result in death or hospitalization but, based on appropriate medical and scientific judgment, may jeopardize the participant or may require intervention (e.g., medical, surgical) to prevent one of the other serious outcomes listed above.
- Suspected transmission of an infectious agent (e.g., any organism, virus or infectious particle, pathogenic or non-pathogenic) via the study drug is an SAE.

If any SAE occurs the investigator will report it to the Sponsor within 24 hours of their being made aware of the SAE. Although pregnancy, overdose and cancer are not always serious by regulatory definition, these events must be handled as SAEs.

NOTE: The following hospitalizations are not considered SAEs in this study:

- A visit to the emergency room or other hospital department lasting less than 24 hours that does not result in admission (unless considered an “important medical event” or a life-threatening event)
- Elective surgery planned before signing consent
- Admissions as per protocol for a planned medical/surgical procedure

- Routine health assessment requiring admission for baseline/trending of health status (e.g., routine colonoscopy)
- Medical/surgical admission for purpose other than remedying ill health state that was planned before study entry. Appropriate documentation is required in these cases.
- Admission encountered for another life circumstance that carries no bearing on health status and requires no medical/surgical intervention (e.g., lack of housing, economic inadequacy, caregiver respite, family circumstances, administrative).

7.3 Non-Serious Adverse Events

Non-serious adverse events are all adverse events that are not classified as SAEs.

7.4 Assignment of Adverse Events

All adverse events, including those that are serious, will be graded by the investigator based on CTCAE v 4.03 as follows:

- Grade 1: Mild; asymptomatic or mild symptoms; clinical or diagnostic observations only; intervention not indicated.
- Grade 2: Moderate; minimal, local or non-invasive intervention indicated; limiting age-appropriate instrumental ADL (activities of daily living)
- Grade 3: Severe or medically significant but not immediately life threatening; hospitalization indicated; disabling, limiting self-care ADL
- Grade 4: Life threatening consequences; urgent intervention indicated
- Grade 5: Death related to AE

The following categories and definitions of causal relationship to investigational product as determined by a physician should be used:

- Related: There is a reasonable causal relationship to investigational product administration and the adverse event.
- Not Related: There is not a reasonable causal relationship between the investigational product administration and the adverse event.

The expression “reasonable causal relationship” is meant to convey in general that there are facts (e.g., evidence such as de-challenge/re-challenge) or other arguments to suggest a positive causal relationship.

7.5 Adverse Events – Collection and Reporting

Adverse events can be spontaneously reported or elicited during open-ended questioning, examination, or evaluation of a participant. To prevent reporting bias, participants should not be questioned regarding the specific occurrence of one or more adverse events.

If known, the diagnosis of the underlying illness or disorder should be recorded, rather than its individual symptoms. The following information should be captured for all AEs: onset, duration, intensity, seriousness, relationship to investigational product, action taken, and treatment required. If treatment for the event was administered, it should be recorded in the medical record.

7.5.1 Adverse Events of Special Interest

Included in the secondary outcome analysis are the adverse events of special interest (AESI). The adverse events of interest include but are not limited to:

Gastrointestinal perforations	Patients presenting with new onset abdominal symptoms should be evaluated promptly for early identification of gastrointestinal perforations and report SAE if event meets criteria.
Herpes Zoster	Though infection Herpes is a separate event of interest for this study.
Malignancy	Solid cancers, lymphoma, and non-melanoma skin cancer.
Serious Infections	Serious infections due to bacterial, mycobacterial, invasive fungal, viral, or other opportunistic pathogens. Examples of these would include: pneumonia, cellulitis, urinary tract infection, and diverticulitis, tuberculosis and other mycobacterial infections, cryptococcosis, esophageal candidiasis, pneumocystosis, multidermatomal, cytomegalovirus, and BK virus.
Lab value abnormalities compared to screening at any time point and confirmed by repeat testing	1. Lymphocyte count less than 500 cells/mm3<br>2. ANC less than 500 cells/mm3<br>3. AST/ALT >3 ULN<br>4. Hy's law= ALT > 3x ULN + bilirubin > 2x ULN<br>5. A drop of Hb by >2 gm/dL<br>6. Hb $\leq$ 8 gm/dL<br>7. Increase in LDL or HDL >50%<br>8. Increase in serum creatinine >50%

7.6 Serious Adverse Event Collection and Reporting

Following the participant's written consent to participate in the study, all AE/SAEs, whether related or not related to study drug, must be collected, including those thought to be associated with protocol-specified procedures. All SAEs must be collected that occur within 30 days of discontinuation of dosing. If applicable, SAEs must be collected that relate to any later protocol-specified procedure (e.g., a follow-up skin biopsy).

The investigator should report any SAE occurring after these time periods that is believed to be related to study drug or protocol-specified procedure.
An SAE report should be completed for any event where doubt exists regarding its status of seriousness.

If the investigator believes that an SAE is not related to study drug, but is potentially related to the conditions of the study (such as withdrawal of previous therapy, or a complication of a study procedure), the relationship should be specified in the narrative section of the SAE Report Form.

7.7 Non-Serious Adverse Event Collection and Reporting

The collection of non-serious adverse event (NSAE) information should begin at initiation of study drug. NSAE information should also be collected from the start of a placebo lead-in period or other observational period intended to establish a baseline status for the participants. NSAEs should be followed to resolution or stabilization, or reported as SAEs if they become serious.

Follow-up is also required for NSAEs that cause interruption or discontinuation of study drug, or those that are present at the end of study treatment as appropriate. All identified NSAEs must be documented appropriately and reported per the sites individual local guidelines.

7.8 Laboratory Monitoring

A lab monitor will provide oversight and will review subject labs in a blinded manner. The following lab values would result in dose reduction/discontinuation of study drug:

1. Lymphocyte count less than 500 cells/mm^3 and confirmed by repeat testing: discontinue drug.
2. Neutropenia. ANC 500–1000 cells/mm^3: For persistent decreases in this range, interrupt dosing until ANC is greater than 1000 cells/mm^3. When greater than 1000 cells/mm^3, resume dose.
3. ANC less than 500 cells/mm^3: discontinue drug
4. Low Hemoglobin. Greater than 2 g/dL decrease or less than 8.0 g/dL: discontinue drug until values have normalized.

8 STATISTICAL CONSIDERATIONS

This study is a two-center, double-blind, randomized, placebo-controlled phase 1/2 clinical trial in which the safety and tolerability of tofacitinib will be assessed in patients with early dcSSc, preliminary efficacy (activity) will be estimated, and exploratory mechanistic research assessments will be performed. Fifteen subjects will be enrolled into the trial, which involves a 24-week course of 5 mg BID tofacitinib or placebo, followed by a 24-week open-label extension period of 5 mg BID tofacitinib.

This section describes the planned analyses; however, a statistical analysis plan (SAP) will be written that provides detailed descriptions of the analyses to be conducted. The SAP will be finalized prior to unblinding of the data.

Continuous variables will be summarized using descriptive statistics including n, mean, median, standard deviation, range (e.g., minimum and maximum). Qualitative variables will be

summarized using counts and percentages. Summaries will be provided by treatment group and overall. Unless otherwise specified, statistical analyses will be performed using SAS Version 9 or higher. Where appropriate, statistical tests will be conducted at the 0.1 significance level using two-tailed tests and p-values will be reported. Given the study objectives and size of this study, the statistical power of any comparisons is limited and p-values will be interpreted as hypothesis generating and not definitive. No adjustment for multiplicity will be made.

8.1 Sample Size Considerations

The planned sample size of 15 evaluable early dcScc subjects is based on practical considerations, rather than a desired power to detect for a pre-specified treatment difference. With the proposed sample of 15 subjects for the primary efficacy analyses (10 tofacitinib and 5 placebo), there would be 80% power to detect large treatment differences. For example, with a two-sided Type I error of 10% with the planned sample size, there is at least 80% power to detect a treatment difference differences in the proportion of participants who experience a Grade 3 or higher adverse event of 20% vs 86%, 10% vs 79%, or 5% vs 74% (placebo vs tofacitinib, respectively). For continuous efficacy (activity) outcomes, the proposed sample size provides 80% power (with a two-sided 10% Type I error) to detect an effect size of 1.37, or greater.

8.2 Statistical Analyses

8.2.1 Safety Outcomes

All safety analyses will be performed on the Safety Population, defined as all subjects who were randomized and received at least one dose of the study drug. Subjects will be analyzed by the treatment received.

Safety measures including AEs, clinical laboratory tests, vital signs, ECG, physical exams, and concomitant medication usage will be summarized descriptively. For quantitative variables, descriptive statistics including number of observations, mean, median, standard deviation and range will be given for the values themselves as well as for change from baseline by treatment group at each study visit. Qualitative variables will be summarized using counts and percentages by treatment group at each study visit.

The primary endpoint is the proportion of participants who experience a Grade 3 or higher adverse event at or before Week 24 and will be compared between the two treatment groups using an exact Fisher's exact test. Exact 90% confidence intervals will be calculated to estimate the incidence of the adverse events (both primary and secondary endpoints) for each treatment group. Similar methods will be used for the secondary safety endpoints. Treatment comparisons for quantitative safety outcomes will be made using two-sample t-tests or their nonparametric counterparts (e.g., Wilcoxon rank sum test). The number (counts) of adverse events will also be compared between treatments for select adverse events (e.g., Grade 3 or higher adverse events at or before Week 24, adverse events of special interest).

8.2.2 Efficacy (Activity) and Exploratory Outcomes

The main population for efficacy and exploratory will be the modified intention-to-treat population (MITT), defined as all subjects randomized, receiving at least one dose of treatment, and having at least one post-baseline efficacy assessment. Subjects will be analyzed by assigned treatment. No adjustment for multiplicity will be made.

Similar methods (described in Section 8.2.1) to compare treatment differences in quantitative and qualitative efficacy and exploratory outcomes will be employed.

Secondary analyses will be performed to assess the robustness of the conclusions and may include the use of a secondary analysis set (e.g., the Per Protocol population (PP) consisting of all subjects in the MITT population who did not have a major protocol violation), alternative methods to deal with missing data, and alternative methods to deal with potential violations of distribution assumptions for the primary analyses. Such sensitivity analyses will be outlined in the SAP for the study.

Additional exploratory analyses may be performed and will be defined and outlined in the SAP for the study.

9 STUDY MANAGEMENT

9.1 Compliance with protocol

The study shall be conducted as described in this approved protocol. The investigator should not implement any deviation or change to the protocol without prior review and documented approval/favorable opinion from the IRB of an amendment, except where necessary to eliminate an immediate hazard(s) to study participants.

If a deviation or change to a protocol is implemented to eliminate an immediate hazard(s) prior to obtaining IRB/ approval/favorable opinion, as soon as possible the deviation or change will be submitted to:

- IRB for review and approval
- Pfizer
- Regulatory Authority(ies), if required by local regulations

If an amendment substantially alters the study design or increases the potential risk to the participant: (1) the consent form must be revised and submitted to the IRB(s) for review and approval/favorable opinion; (2) the revised form must be used to obtain consent from participants currently enrolled in the study if they are affected by the amendment; and (3) the new form must be used to obtain consent from new participants prior to enrollment.

If the revision is an administrative letter, investigators must inform their IRB(s).

9.2 Record Retention

Source documents are original documents, data, and records from which the subject's case report form data are obtained. These include but are not limited to hospital records, clinical and office charts, laboratory and pharmacy records, diaries, imaging, and correspondence. All original source documents supporting entries in the case report forms must be maintained and be readily available.

The Investigator and the study center staff are responsible for maintaining a comprehensive and centralized filing system of all study-related (essential) documentation in accordance with Section 8 of the ICH Guidelines (E6), suitable for inspection at any time by representatives from the Sponsor or designee and/or applicable regulatory authorities. The clinical site's regulatory document binder essential elements should include:

- Subject files containing completed case report forms (eCRFs), informed consents/assents, and supporting copies of source documentation
- Study files containing the protocol with all amendments, Package Insert, copies of pre-study documentation and all correspondence to and from the IEC/IRB and the Sponsor or designee.
- If drug supplies are maintained at the study center, documentation for proof of receipt, study drug accountability records, return of study drug for destruction, final study drug product reconciliation statement, and all drug-related correspondence.

The investigator must retain all study records and source documents for the maximum period required by applicable regulations and guidelines, or institution procedures, whichever is longer.

9.3 Study Drug Record

It is the responsibility of the investigator to ensure that a current disposition record of investigational product is maintained at each study site where study drug is inventoried and dispensed. Records or logs must comply with applicable regulations and guidelines.

9.4 Study Drug Destruction

Study drugs are to be destroyed on site upon sponsor approval, it is the investigator's responsibility to ensure that arrangements have been made for disposal, and that procedures for proper disposal have been established according to applicable regulations, guidelines, and institutional procedures. Appropriate records of the disposal must be maintained.

9.5 Study Monitoring

Throughout the course of the study, data will be monitored for accuracy and completeness and study procedures will be monitored for adherence to the protocol and Good Clinical Practices (GCP). Clinical data monitoring will be conducted utilizing experienced clinical monitors. The monitoring plan includes routine remote monitoring with 1 annual site visit.

During the on-site visits, the CRFs will be reviewed for completeness and adherence to the protocol, accuracy, consistency of data, and adherence to local regulations on the conduct of clinical research.

A Medical Safety Monitor, a physician with clinical trials experience and expertise in scleroderma who is independent of the conduct of the study, will review all SAEs.

10 RESEARCH USE OF HUMAN SPECIMENS, SAMPLES, OR DATA

10.1 Use of Stored Samples and Data

Samples and data collection under this protocol may be used to study effect of tofacitinib on skin and blood gene expression and effect on T-cell biology. No genetic testing will be performed.

10.2 Disposition of Stored Samples and Data

Access to stored samples will be limited using a locked freezer. Samples and data will be stored using codes assigned by the investigators. Data will be kept in password-protected computers. Only investigators will have access to the samples and data.

- Samples and data acquired will be kept at University of Michigan.
- Skin biopsies will be housed at University of Pittsburgh

At the completion of the protocol (termination), samples and data will be reserved for future, unspecified research if the subject has consented to analysis of their specimens outside of this protocol. If the subject doesn't consent to long term storage, the samples will be destroyed at the end of the study.

Additionally, subjects may decide at any point during the study not to have their samples stored. In this case, the principal investigator will destroy all known remaining samples and report what was done to both the subject and to the IRB. This decision will not affect the subject's participation in this protocol.

11 REFERENCES

1. Distler O, Cozzio A. Systemic sclerosis and localized scleroderma--current concepts and novel targets for therapy. Semin Immunopathol. 2016;38(1):87-95. doi: 10.1007/s00281-015-0551-z. PubMed PMID: 26577237.
2. Ghoreschi K, Jesson MI, Li X, Lee JL, Ghosh S, Alsup JW, Warner JD, Tanaka M, Steward-Tharp SM, Gadina M, Thomas CJ, Minnerly JC, Storer CE, LaBranche TP, Radi ZA, Dowty ME, Head RD, Meyer DM, Kishore N, O'Shea JJ. Modulation of innate and adaptive immune responses by tofacitinib (CP-690,550). Journal of immunology. 2011;186(7):4234-43. doi: 10.4049/jimmunol.1003668. PubMed PMID: 21383241; PMCID: 3108067.
3. Khanna D, Denton CP, Jahreis A, van Laar JM, Frech TM, Anderson ME, Baron M, Chung L, Fierlbeck G, Lakshminarayanan S, Allanore Y, Pope JE, Riemekasten G, Steen V, Muller-Ladner U, Lafyatis R, Stifano G, Spotswood H, Chen-Harris H, Dziadek S, Morimoto A, Sornasse T, Siegel J, Furst DE. Safety and efficacy of subcutaneous

tocilizumab in adults with systemic sclerosis (faSScinate): a phase 2, randomised, controlled trial. Lancet. 2016;387(10038):2630-40. doi: 10.1016/S0140-6736(16)00232-4. PubMed PMID: 27156934.

4. Schwartz DM, Bonelli M, Gadina M, O'Shea JJ. Type I/II cytokines, JAKs, and new strategies for treating autoimmune diseases. Nature reviews Rheumatology. 2016;12(1):25-36. doi: 10.1038/nrrheum.2015.167. PubMed PMID: 26633291; PMCID: 4688091.
5. Hugle T, Hogan V, White KE, van Laar JM. Mast cells are a source of transforming growth factor beta in systemic sclerosis. Arthritis and rheumatism. 2011;63(3):795-9. doi: 10.1002/art.30190. PubMed PMID: 21360509.
6. Kalogerou A, Gelou E, Mountantonakis S, Settas L, Zafiriou E, Sakkas L. Early T cell activation in the skin from patients with systemic sclerosis. Annals of the rheumatic diseases. 2005;64(8):1233-5. doi: 10.1136/ard.2004.027094. PubMed PMID: 16014686; PMCID: 1755597.
7. Roumm AD, Whiteside TL, Medsger TA, Jr., Rodnan GP. Lymphocytes in the skin of patients with progressive systemic sclerosis. Quantification, subtyping, and clinical correlations. Arthritis and rheumatism. 1984;27(6):645-53. PubMed PMID: 6375682.
8. Fleischmajer R, Perlish JS, Reeves JR. Cellular infiltrates in scleroderma skin. Arthritis and rheumatism. 1977;20(4):975-84. PubMed PMID: 861067.
9. Hasegawa M, Fujimoto M, Matsushita T, Hamaguchi Y, Takehara K. Augmented ICOS expression in patients with early diffuse cutaneous systemic sclerosis. Rheumatology. 2013;52(2):242-51. doi: 10.1093/rheumatology/kes258. PubMed PMID: 23024058.
10. Phelps RG, Daian C, Shibata S, Fleischmajer R, Bona CA. Induction of skin fibrosis and autoantibodies by infusion of immunocompetent cells from tight skin mice into C57BL/6 Pa/Pa mice. Journal of autoimmunity. 1993;6(6):701-18. doi: 10.1006/jaut.1993.1059. PubMed PMID: 8155252.
11. Hugle T, O'Reilly S, Simpson R, Kraaij MD, Bigley V, Collin M, Krippner-Heidenreich A, van Laar JM. Tumor necrosis factor-costimulated T lymphocytes from patients with systemic sclerosis trigger collagen production in fibroblasts. Arthritis and rheumatism. 2013;65(2):481-91. doi: 10.1002/art.37738. PubMed PMID: 23045159.
12. Abraham DJ, Varga J. Scleroderma: from cell and molecular mechanisms to disease models. Trends in immunology. 2005;26(11):587-95. doi: 10.1016/j.it.2005.09.004. PubMed PMID: 16168711.
13. Varga J, Abraham D. Systemic sclerosis: a prototypic multisystem fibrotic disorder. The Journal of clinical investigation. 2007;117(3):557-67. doi: 10.1172/JCI31139. PubMed PMID: 17332883; PMCID: 1804347.
14. Greenblatt MB, Sargent JL, Farina G, Tsang K, Lafyatis R, Glimcher LH, Whitfield ML, Aliprantis AO. Interspecies comparison of human and murine scleroderma reveals IL-13 and CCL2 as disease subset-specific targets. The American journal of pathology. 2012;180(3):1080-94. doi: 10.1016/j.ajpath.2011.11.024. PubMed PMID: 22245215; PMCID: 3349888.
15. Kaviratne M, Hesse M, Leusink M, Cheever AW, Davies SJ, McKerrow JH, Wakefield LM, Letterio JJ, Wynn TA. IL-13 activates a mechanism of tissue fibrosis that is completely TGF-beta independent. Journal of immunology. 2004;173(6):4020-9. PubMed PMID: 15356151.
16. Wynn TA. Fibrotic disease and the T(H)1/T(H)2 paradigm. Nature reviews Immunology. 2004;4(8):583-94. doi: 10.1038/nri1412. PubMed PMID: 15286725; PMCID: 2702150.
17. McGaha TL, Le M, Kodera T, Stoica C, Zhu J, Paul WE, Bona CA. Molecular mechanisms of interleukin-4-induced up-regulation of type I collagen gene expression in

murine fibroblasts. Arthritis and rheumatism. 2003;48(8):2275-84. doi: 10.1002/art.11089. PubMed PMID: 12905482.
18. Brinckmann J, Kim S, Wu J, Reinhardt DP, Batmunkh C, Metzen E, Notbohm H, Bank RA, Krieg T, Hunzelmann N. Interleukin 4 and prolonged hypoxia induce a higher gene expression of lysyl hydroxylase 2 and an altered cross-link pattern: important pathogenetic steps in early and late stage of systemic scleroderma? Matrix biology : journal of the International Society for Matrix Biology. 2005;24(7):459-68. doi: 10.1016/j.matbio.2005.07.002. PubMed PMID: 16139999.
19. Khanna D, Saggar R, Mayes MD, Abtin F, Clements PJ, Maranian P, Assassi S, Saggar R, Singh RR, Furst DE. A one-year, phase I/IIa, open-label pilot trial of imatinib mesylate in the treatment of systemic sclerosis-associated active interstitial lung disease. Arthritis and rheumatism. 2011;63(11):3540-6. doi: 10.1002/art.30548. PubMed PMID: 21769849; PMCID: 3205223.
20. Divekar AA, Khanna D, Abtin F, Maranian P, Saggar R, Saggar R, Furst DE, Singh RR. Treatment with imatinib results in reduced IL-4-producing T cells, but increased CD4(+) T cells in the broncho-alveolar lavage of patients with systemic sclerosis. Clinical immunology. 2011;141(3):293-303. doi: 10.1016/j.clim.2011.08.010. PubMed PMID: 22015344; PMCID: 3221795.
21. MacDonald KG, Dawson NA, Huang Q, Dunne JV, Levings MK, Broady R. Regulatory T cells produce profibrotic cytokines in the skin of patients with systemic sclerosis. The Journal of allergy and clinical immunology. 2015;135(4):946- e9. doi: 10.1016/j.jaci.2014.12.1932. PubMed PMID: 25678090.
22. Brembilla NC, Chizzolini C. T cell abnormalities in systemic sclerosis with a focus on Th17 cells. European cytokine network. 2012;23(4):128-39. doi: 10.1684/ecn.2013.0325. PubMed PMID: 23360781.
23. Fenoglio D, Battaglia F, Parodi A, Stringara S, Negrini S, Panico N, Rizzi M, Kalli F, Conteduca G, Ghio M, De Palma R, Indiveri F, Filaci G. Alteration of Th17 and Treg cell subpopulations co-exist in patients affected with systemic sclerosis. Clinical immunology. 2011;139(3):249-57. doi: 10.1016/j.clim.2011.01.013. PubMed PMID: 21419712.
24. Fenoglio D, Bernuzzi F, Battaglia F, Parodi A, Kalli F, Negrini S, De Palma R, Invernizzi P, Filaci G. Th17 and regulatory T lymphocytes in primary biliary cirrhosis and systemic sclerosis as models of autoimmune fibrotic diseases. Autoimmunity reviews. 2012;12(2):300-4. doi: 10.1016/j.autrev.2012.05.004. PubMed PMID: 22634708.
25. Murata M, Fujimoto M, Matsushita T, Hamaguchi Y, Hasegawa M, Takehara K, Komura K, Sato S. Clinical association of serum interleukin-17 levels in systemic sclerosis: is systemic sclerosis a Th17 disease? Journal of dermatological science. 2008;50(3):240-2. doi: 10.1016/j.jdermsci.2008.01.001. PubMed PMID: 18329249.
26. Papp G, Horvath IF, Barath S, Gyimesi E, Vegh J, Szodoray P, Zeher M. Immunomodulatory effects of extracorporeal photochemotherapy in systemic sclerosis. Clinical immunology. 2012;142(2):150-9. doi: 10.1016/j.clim.2011.09.014. PubMed PMID: 22036269.
27. Truchetet ME, Brembilla NC, Montanari E, Allanore Y, Chizzolini C. Increased frequency of circulating Th22 in addition to Th17 and Th2 lymphocytes in systemic sclerosis: association with interstitial lung disease. Arthritis research & therapy. 2011;13(5):R166. doi: 10.1186/ar3486. PubMed PMID: 21996293; PMCID: 3308100.
28. Truchetet ME, Brembilla NC, Montanari E, Lonati P, Raschi E, Zeni S, Fontao L, Meroni PL, Chizzolini C. Interleukin-17A+ cell counts are increased in systemic sclerosis skin and their number is inversely correlated with the extent of skin involvement. Arthritis and rheumatism. 2013;65(5):1347-56. doi: 10.1002/art.37860. PubMed PMID: 23335253.

29. Martin JE, Bossini-Castillo L, Martin J. Unraveling the genetic component of systemic sclerosis. Human genetics. 2012;131(7):1023-37. doi: 10.1007/s00439-011-1137-z. PubMed PMID: 22218928.
30. Allanore Y, Saad M, Dieude P, Avouac J, Distler JH, Amouyel P, Matucci-Cerinic M, Riemekasten G, Airo P, Melchers I, Hachulla E, Cusi D, Wichmann HE, Wipff J, Lambert JC, Hunzelmann N, Tiev K, Caramaschi P, Diot E, Kowal-Bielecka O, Valentini G, Mouthon L, Czirjak L, Damjanov N, Salvi E, Conti C, Muller M, Muller-Ladner U, Riccieri V, Ruiz B, Cracowski JL, Letenneur L, Dupuy AM, Meyer O, Kahan A, Munnich A, Boileau C, Martinez M. Genome-wide scan identifies TNIP1, PSORS1C1, and RHOB as novel risk loci for systemic sclerosis. PLoS genetics. 2011;7(7):e1002091. doi: 10.1371/journal.pgen.1002091. PubMed PMID: 21750679; PMCID: 3131285.
31. Gorlova O, Martin JE, Rueda B, Koeleman BP, Ying J, Teruel M, Diaz-Gallo LM, Broen JC, Vonk MC, Simeon CP, Alizadeh BZ, Coenen MJ, Voskuyl AE, Schuerwegh AJ, van Riel PL, Vanthuyne M, van 't Slot R, Italiaander A, Ophoff RA, Hunzelmann N, Fonollosa V, Ortego-Centeno N, Gonzalez-Gay MA, Garcia-Hernandez FJ, Gonzalez-Escribano MF, Airo P, van Laar J, Worthington J, Hesselstrand R, Smith V, de Keyser F, Houssiau F, Chee MM, Madhok R, Shiels PG, Westhovens R, Kreuter A, de Baere E, Witte T, Padyukov L, Nordin A, Scorza R, Lunardi C, Lie BA, Hoffmann-Vold AM, Palm O, Garcia de la Pena P, Carreira P, Spanish Scleroderma G, Varga J, Hinchcliff M, Lee AT, Gourh P, Amos CI, Wigley FM, Hummers LK, Nelson JL, Riemekasten G, Herrick A, Beretta L, Fonseca C, Denton CP, Gregersen PK, Agarwal S, Assassi S, Tan FK, Arnett FC, Radstake TR, Mayes MD, Martin J. Identification of novel genetic markers associated with clinical phenotypes of systemic sclerosis through a genome-wide association strategy. PLoS genetics. 2011;7(7):e1002178. doi: 10.1371/journal.pgen.1002178. PubMed PMID: 21779181; PMCID: 3136437.
32. Radstake TR, Gorlova O, Rueda B, Martin JE, Alizadeh BZ, Palomino-Morales R, Coenen MJ, Vonk MC, Voskuyl AE, Schuerwegh AJ, Broen JC, van Riel PL, van 't Slot R, Italiaander A, Ophoff RA, Riemekasten G, Hunzelmann N, Simeon CP, Ortego-Centeno N, Gonzalez-Gay MA, Gonzalez-Escribano MF, Spanish Scleroderma G, Airo P, van Laar J, Herrick A, Worthington J, Hesselstrand R, Smith V, de Keyser F, Houssiau F, Chee MM, Madhok R, Shiels P, Westhovens R, Kreuter A, Kiener H, de Baere E, Witte T, Padykov L, Klareskog L, Beretta L, Scorza R, Lie BA, Hoffmann-Vold AM, Carreira P, Varga J, Hinchcliff M, Gregersen PK, Lee AT, Ying J, Han Y, Weng SF, Amos CI, Wigley FM, Hummers L, Nelson JL, Agarwal SK, Assassi S, Gourh P, Tan FK, Koeleman BP, Arnett FC, Martin J, Mayes MD. Genome-wide association study of systemic sclerosis identifies CD247 as a new susceptibility locus. Nature genetics. 2010;42(5):426-9. doi: 10.1038/ng.565. PubMed PMID: 20383147; PMCID: 2861917.
33. Martin JE, Carmona FD, Broen JC, Simeon CP, Vonk MC, Carreira P, Rios-Fernandez R, Espinosa G, Vicente-Rabaneda E, Tolosa C, Garcia-Hernandez FJ, Castellvi I, Fonollosa V, Gonzalez-Gay MA, Saez-Comet L, Portales RG, de la Pena PG, Fernandez-Castro M, Diaz B, Martinez-Estupinan L, Coenen M, Voskuyl AE, Schuerwegh AJ, Vanthuyne M, Houssiau F, Smith V, de Keyser F, De Langhe E, Riemekasten G, Witte T, Hunzelmann N, Kreuter A, Palm O, Chee MM, van Laar JM, Denton C, Herrick A, Worthington J, Koeleman BP, Radstake TR, Fonseca C, Martin J, Spanish Scleroderma G. The autoimmune disease-associated IL2RA locus is involved in the clinical manifestations of systemic sclerosis. Genes and immunity. 2012;13(2):191-6. doi: 10.1038/gene.2011.72. PubMed PMID: 22012429.
34. Lian X, Xiao R, Hu X, Kanekura T, Jiang H, Li Y, Wang Y, Yang Y, Zhao M, Lu Q. DNA demethylation of CD40l in CD4+ T cells from women with systemic sclerosis: a possible

explanation for female susceptibility. Arthritis and rheumatism. 2012;64(7):2338-45. doi: 10.1002/art.34376. PubMed PMID: 22231486.
35. Lei W, Luo Y, Lei W, Luo Y, Yan K, Zhao S, Li Y, Qiu X, Zhou Y, Long H, Zhao M, Liang Y, Su Y, Lu Q. Abnormal DNA methylation in CD4+ T cells from patients with systemic lupus erythematosus, systemic sclerosis, and dermatomyositis. Scandinavian journal of rheumatology. 2009;38(5):369-74. doi: 10.1080/03009740902758875. PubMed PMID: 19444718.
36. Conklyn M, Andresen C, Changelian P, Kudlacz E. The JAK3 inhibitor CP-690550 selectively reduces NK and CD8+ cell numbers in cynomolgus monkey blood following chronic oral dosing. Journal of leukocyte biology. 2004;76(6):1248-55. doi: 10.1189/jlb.0504282. PubMed PMID: 15371489.
37. Paniagua R, Si MS, Flores MG, Rousvoal G, Zhang S, Aalami O, Campbell A, Changelian PS, Reitz BA, Borie DC. Effects of JAK3 inhibition with CP-690,550 on immune cell populations and their functions in nonhuman primate recipients of kidney allografts. Transplantation. 2005;80(9):1283-92. PubMed PMID: 16314797.
38. van Gurp EA, Schoordijk-Verschoor W, Klepper M, Korevaar SS, Chan G, Weimar W, Baan CC. The effect of the JAK inhibitor CP-690,550 on peripheral immune parameters in stable kidney allograft patients. Transplantation. 2009;87(1):79-86. doi: 10.1097/TP.0b013e31818bbea7. PubMed PMID: 19136895.
39. Migita K, Miyashita T, Izumi Y, Koga T, Komori A, Maeda Y, Jiuchi Y, Aiba Y, Yamasaki S, Kawakami A, Nakamura M, Ishibashi H. Inhibitory effects of the JAK inhibitor CP690,550 on human CD4(+) T lymphocyte cytokine production. BMC immunology. 2011;12:51. doi: 10.1186/1471-2172-12-51. PubMed PMID: 21884580; PMCID: 3179939.
40. Tanaka Y, Maeshima K, Yamaoka K. In vitro and in vivo analysis of a JAK inhibitor in rheumatoid arthritis. Annals of the rheumatic diseases. 2012;71 Suppl 2:i70-4. doi: 10.1136/annrheumdis-2011-200595. PubMed PMID: 22460142.
41. Maeshima K, Yamaoka K, Kubo S, Nakano K, Iwata S, Saito K, Ohishi M, Miyahara H, Tanaka S, Ishii K, Yoshimatsu H, Tanaka Y. The JAK inhibitor tofacitinib regulates synovitis through inhibition of interferon-gamma and interleukin-17 production by human CD4+ T cells. Arthritis and rheumatism. 2012;64(6):1790-8. doi: 10.1002/art.34329. PubMed PMID: 22147632.
42. Kudlacz E, Conklyn M, Andresen C, Whitney-Pickett C, Changelian P. The JAK-3 inhibitor CP-690550 is a potent anti-inflammatory agent in a murine model of pulmonary eosinophilia. European journal of pharmacology. 2008;582(1-3):154-61. doi: 10.1016/j.ejphar.2007.12.024. PubMed PMID: 18242596.
43. Sewgobind VD, Quaedackers ME, van der Laan LJ, Kraaijeveld R, Korevaar SS, Chan G, Weimar W, Baan CC. The Jak inhibitor CP-690,550 preserves the function of CD4CD25FoxP3 regulatory T cells and inhibits effector T cells. American journal of transplantation : official journal of the American Society of Transplantation and the American Society of Transplant Surgeons. 2010;10(8):1785-95. doi: 10.1111/j.1600-6143.2010.03200.x. PubMed PMID: 20626385.
44. Kubo S, Yamaoka K, Kondo M, Yamagata K, Zhao J, Iwata S, Tanaka Y. The JAK inhibitor, tofacitinib, reduces the T cell stimulatory capacity of human monocyte-derived dendritic cells. Annals of the rheumatic diseases. 2014;73(12):2192-8. doi: 10.1136/annrheumdis-2013-203756. PubMed PMID: 24013646.
45. Nishimura K, Saegusa J, Matsuki F, Akashi K, Kageyama G, Morinobu A. Tofacitinib facilitates the expansion of myeloid-derived suppressor cells and ameliorates arthritis in SKG mice. Arthritis & rheumatology. 2015;67(4):893-902. doi: 10.1002/art.39007. PubMed PMID: 25545152.

46. Migita K, Izumi Y, Torigoshi T, Satomura K, Izumi M, Nishino Y, Jiuchi Y, Nakamura M, Kozuru H, Nonaka F, Eguchi K, Kawakami A, Motokawa S. Inhibition of Janus kinase/signal transducer and activator of transcription (JAK/STAT) signalling pathway in rheumatoid synovial fibroblasts using small molecule compounds. Clinical and experimental immunology. 2013;174(3):356-63. doi: 10.1111/cei.12190. PubMed PMID: 23968543; PMCID: 3826302.
47. Rosengren S, Corr M, Firestein GS, Boyle DL. The JAK inhibitor CP-690,550 (tofacitinib) inhibits TNF-induced chemokine expression in fibroblast-like synoviocytes: autocrine role of type I interferon. Annals of the rheumatic diseases. 2012;71(3):440-7. doi: 10.1136/ard.2011.150284. PubMed PMID: 22121136.
48. LaBranche TP, Jesson MI, Radi ZA, Storer CE, Guzova JA, Bonar SL, Thompson JM, Happa FA, Stewart ZS, Zhan Y, Bollinger CS, Bansal PN, Wellen JW, Wilkie DP, Bailey SA, Symanowicz PT, Hegen M, Head RD, Kishore N, Mbalaviele G, Meyer DM. JAK inhibition with tofacitinib suppresses arthritic joint structural damage through decreased RANKL production. Arthritis and rheumatism. 2012;64(11):3531-42. doi: 10.1002/art.34649. PubMed PMID: 22899318.
49. Dees C, Tomcik M, Palumbo-Zerr K, Distler A, Beyer C, Lang V, Horn A, Zerr P, Zwerina J, Gelse K, Distler O, Schett G, Distler JH. JAK-2 as a novel mediator of the profibrotic effects of transforming growth factor beta in systemic sclerosis. Arthritis and rheumatism. 2012;64(9):3006-15. doi: 10.1002/art.34500. PubMed PMID: 22549363.
50. Okiyama N, Furumoto Y, Villarroel VA, Linton JT, Tsai WL, Gutermuth J, Ghoreschi K, Gadina M, O'Shea JJ, Katz SI. Reversal of CD8 T-cell-mediated mucocutaneous graft-versus-host-like disease by the JAK inhibitor tofacitinib. The Journal of investigative dermatology. 2014;134(4):992-1000. doi: 10.1038/jid.2013.476. PubMed PMID: 24213371; PMCID: 3961527.
51. van den Hoogen F, Khanna D, Fransen J, Johnson SR, Baron M, Tyndall A, Matucci-Cerinic M, Naden RP, Medsger TA, Jr., Carreira PE, Riemekasten G, Clements PJ, Denton CP, Distler O, Allanore Y, Furst DE, Gabrielli A, Mayes MD, van Laar JM, Seibold JR, Czirjak L, Steen VD, Inanc M, Kowal-Bielecka O, Muller-Ladner U, Valentini G, Veale DJ, Vonk MC, Walker UA, Chung L, Collier DH, Ellen Csuka M, Fessler BJ, Guiducci S, Herrick A, Hsu VM, Jimenez S, Kahaleh B, Merkel PA, Sierakowski S, Silver RM, Simms RW, Varga J, Pope JE. 2013 classification criteria for systemic sclerosis: an American college of rheumatology/European league against rheumatism collaborative initiative. Ann Rheum Dis. 2013;72(11):1747-55. doi: 10.1136/annrheumdis-2013-204424. PubMed PMID: 24092682.
52. LeRoy EC, Medsger TA, Jr. Criteria for the classification of early systemic sclerosis. The Journal of rheumatology. 2001;28(7):1573-6. Epub 2001/07/27. PubMed PMID: 11469464.
